# Reduced expression of SOX7 in ovarian cancer: a novel tumor suppressor through the Wnt/β-catenin signaling pathway

**DOI:** 10.1186/s13048-014-0087-1

**Published:** 2014-09-05

**Authors:** Huidi Liu, Zi-Qiao Yan, Bailiang Li, Si-Yuan Yin, Qiang Sun, Jun-Jie Kou, Dan Ye, Kelsey Ferns, Hong-Yu Liu, Shu-Lin Liu

**Affiliations:** Genomics Research Centre, Harbin Medical University, Harbin, 150081 China; HMU-UCFM Centre for Infection and Genomics, Harbin, China; Pathology Department, The First Hospital of Qiqihaer City, Qiqihaer, 161006 China; Department of Microbiology, Immunology and Infectious Diseases, University of Calgary, Calgary, Canada; Current address: Department of Biology, University of British Columbia, Vancouver, Canada

**Keywords:** SOX7, Tumor suppressor, Ovarian cancer, Wnt/β-catenin signaling pathway

## Abstract

**Background:**

Products of the SOX gene family play important roles in the life process. One of the members, SOX7, is associated with the development of a variety of cancers as a tumor suppression factor, but its relevance with ovarian cancer was unclear. In this study, we investigated the involvement of SOX7 in the progression and prognosis of epithelial ovarian cancer (EOC) and the involved mechanisms.

**Methods:**

Expression profiles in two independent microarray data sets were analyzed for SOX7 between malignant and normal tissues. The expression levels of SOX7 in EOC, borderline ovarian tumors and normal ovarian tissues were measured by immunohistochemistry. We also measured levels of COX2 and cyclin-D1 to examine their possible involvement in the same signal transduction pathway as SOX7.

**Results:**

The expression of SOX7 was significantly reduced in ovarian cancer tissues compared with normal controls, strongly indicating that SOX7 might be a negative regulator in the Wnt/β-catenin pathway in ovarian cancer. By immunohistochemistry staining, the protein expression of SOX7 showed a consistent trend with that of the gene expression microarray analysis. By contrast, the protein expression level of COX2 and cyclin-D1 increased as the tumor malignancy progressed, suggesting that SOX7 may function through the Wnt/β-catenin signaling pathway as a tumor suppressor. In comparison between the protein expression levels of SOX7 with pathological features of the cancer, we found that SOX7 was down-regulated mainly in serous cystadenocarcinoma and advanced stages of the cancers.

**Conclusions:**

The expression of SOX7 correlates with tumor progression as a tumor suppressor, possibly through the Wnt/β-catenin signaling pathway in ovarian cancers, suggesting that SOX7 may be a promising prognostic marker.

**Electronic supplementary material:**

The online version of this article (doi:10.1186/s13048-014-0087-1) contains supplementary material, which is available to authorized users.

## Background

Ovarian cancer is one of the three most common cancers in female with high morbidity and mortality rates [1]. The disease is also among the most lethal cancers in females, primarily because of its aggressive metastasis within the peritoneal cavity. It is hardly detectable at the early stage, largely due to the lack of specific symptoms and reliable screening [2,3]. Among all types, epithelial ovarian cancer (EOC) is the most common, accounting for 85% to 90% of ovarian cancers [4], and this type is highly lethal. This situation calls for the investigation of pathogenesis of EOC and the identification of molecular markers for early diagnosis or for use as targets in treatment.

SOX7, a member of subfamily SOXF along with SOX17 and SOX18, has been identified as a developmental regulator in hematopoiesis and cardiogenesis [5–9]. The human SOX7 gene is located at the chromosomal region of 8p23.1 and is approximately 7.7 kbps in length [10]; a frequently methylated CpG island on the promoter region of the SOX7 gene plays a role in regulating SOX7 gene expression. SOX7 is frequently down-regulated in many human cancers, such as prostate, colon, lung, and breast cancers, and its reduced expression often correlates with poor prognoses [11–13]. It is reported that SOX7 can directly bind β-catenin and negatively regulate its activity [14,15].

The Wnt/β-catenin signal transduction pathway is widely acknowledged as playing a dominating role in human diseases, especially in the occurrence and development of cancer [16]. Although the molecular mechanism by which SOX7 produces its tumor suppressive effects has yet to be fully determined, SOX7 has been shown to interact with β-catenin and inhibit cell proliferation mediated by the Wnt signaling pathways. Evidence is accumulating to show that SOX7 may disrupt the transcriptional function of the β-catenin-TCF/LEF complex and inhibit the activity of Wnt target genes including cyclin D1, c-Myc and COX-2 [15,17]. Additionally, recent studies indicate that SOX7 may act as a tumor suppressor [11,13,14]. Ectopic SOX7 expression is reported to have a regulating function against cell growth and promote apoptosis; meanwhile, SOX7 gene knockdown may lead to neoplastic transformation [14,18]. As SOX7 negatively regulates the Wnt/β-catenin signaling pathway by impeding the transcriptional machinery of β-catenin/TCF/LEF-1, inactivation of SOX7 may be associated with the pathogenesis of cancers, e.g., endometrial cancer and prostate cancer [14,15]. Additionally, SOX7 may be involved in aspirin-mediated growth inhibition of COX2 colorectal cancer cells, in which aspirin may up-regulate the expression of SOX7 by activating the MAPK [17]. There are also reports indicating that the decreased expression of SOX7 is an important feature of lung adenocarcinoma [12]. Based on whole genome analysis to measure the expression of SOX7 on a series of non-small cell lung cancers (NSCLC), investigators have shown that SOX7 is a novel tumor suppressor gene silenced in the majority of NSCLC [19]. Numerous studies also suggest that promoter methylation might be an important mechanism of SOX7 down-regulation in breast cancer and MDS [11,13]. On the other hand, reports are also available to show that SOX7 mRNA is significantly up-regulated in cancer, such as in pancreatic, gastric, and esophageal cancer cell lines, as well as in primary gastric cancer [20]. To date, nevertheless, the contributions and molecular mechanisms of SOX7 in ovarian cancer are largely unknown.

In this study, we investigated the changes and possible roles of SOX7 in ovarian cancer using the microarray gene expression techniques and validated the results with clinical tissues. Both kinds of data showed that the expression of SOX7 was significantly reduced in ovarian cancer and the levels of expression were correlated with tumour progression. This effect might be mediated by the down-regulation of Wnt target genes cyclinD1 and COX2.

## Methods

### Chemicals and antibodies

Peroxidase-labeled rabbit-anti human IgG SOX7 (sc-20093) was purchased from Santa cruz biotechnology. Peroxidase-labeled rabbit-anti human IgG COX2 (BA3708) and rabbit-anti human IgG cyclinD1 (BA0770) used in immunohistochemistry were purchased from BOSTER Bio, Wuhan, China. Histostain-Plus Kit (SP-9001) and 3, 3′-diaminobenzidine tetrahydrochoride Substrate Kit (ZLI-9032) used in immunohistochemistry were purchased from ZSGB Bio, Beijing, China.

### Datasets and preprocessing

Two sets of normalized microarray gene expression data with accession numbers GSE12470 and GSE27651 were downloaded from the Gene Expression Omnibus data repository (GEO, http://www.ncbi.nlm.nih.gov/geo/). Probe sets that did not match any known Gene ID or that matched multiple Gene IDs were abandoned. For each of the samples, the expression values of the probe sets matched to the same Entrez Gene ID were averaged as the expression value of that Entrez Gene ID. Genes in GSE27651, the expression of which significantly correlated (Pearson correlation) with that of SOX7, were defined as coexpression genes with SOX7. The pathway information of Wnt/β-catenin was documented in Kyoto Encyclopedia of Genes and Genomes (KEGG) website.

### Functional enrichment analysis

The functional enrichment analysis was performed using the Database for Annotation, Visualization and Integrated Discovery (DAVID) v6.7 [21,22] and the biological processes (BPs) within Gene Ontology (GO) were checked for overrepresented function entities.

### Clinical specimens

Clinical specimens were collected from the Department of Pathology of the Third Affiliated Hospital of Harbin Medical University from January to June 2008. All patients were informed of the purpose of the study and gave written informed consents. This work was reviewed by the Harbin Medical University Ethics Committee and was approved, consistent with the 1975 Declaration of Helsinki. The stage and histological grades of all the cases were determined according to the criteria of FIGO. None of the patients received any chemotherapy or radiotherapy before operation. Clinical information of patients was obtained from medical records and pathology reports.

### Immunohistochemistry

The samples were fixed in 10% neutral buffered formalin and subsequently embedded in paraffin. The paraffin-embedded tissues were cut at 3 μm, deparaffinized with xylene and rehydrated for further peroxidase (DAB) immunohistochemistry staining. After trypsinization, the tissue slides were blocked with peroxidase and then were incubated overnight with the primary antibodies against respective target proteins (SOX7 Ab., Santa Cruz #sc-20093; Rabbit Anti-COX2, ZSGB-BIO, Rabbit Anti-CyclinD1, ZSGB-BIO) at a dilution of 1:100 at 4°C. After washing, peroxidase labeled polymer and substrate-chromogen were applied to visualize the staining of the target proteins. Finally, the slides were counterstained with hematoxylin.

### Standard for evaluation

Immunostaining was scored by two independent experienced pathologists, who were blinded to the clinicopathological data and clinical outcomes of the patients. The scores of the two pathologists were compared and any discrepant scores were trained through re-examining the staining by both pathologists to achieve a consensus score. Tumor specimens were scored in a semi-quantitative manner in consideration of the homogenicity of the staining of the target proteins. The percentage scoring of immunoreactive tumor cells was as follows: 0 (0%), 1 (1-10%), 2 (11-50%) and 3 (>50%). The staining intensity was externally scored and stratified as follows: 0 (negative), 1 (weak), 2 (moderate) and 3 (strong). A final immunoreactivity score (IRS) was obtained for each of the cases by multiplying the percentage and the intensity score. Protein expression levels were further analyzed by classifying IRS values as low (based on an IRS value less than 4) or high (based on an IRS value greater than 4).

### Statistical analysis

The differentially expressed genes of ovarian cancers vs. normal controls in GSE12470 were calculated by student-t test and the false discovery rate was controlled using the Benjamini–Hochberg procedure [23]. The gene expression level of SOX7 was compared in groups of different malignant states in GSE27651 using one-way analysis of variance (one-way ANOVA). The difference between each pair of the groups was tested by multiple comparison tests and the statistical significance level was given by a student-t test.

For the immunohistochemistry experiment, SPSS version 17.0 was used. Statistical analysis was performed with Fisher’s exact test, chi square test and Spearman’s Rank correlation analysis. Differences with *p* < 0.05 were considered statistically significant.

## Results

### Expression levels of SOX7 in ovarian cancer and normal tissues

**Figure 1:**
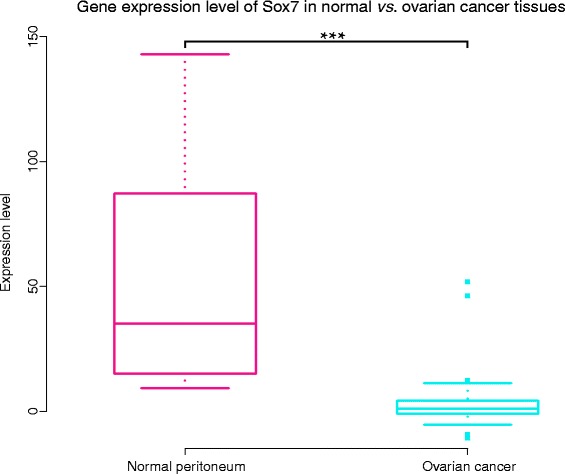
**Down-regulated SOX7 in ovarian cancer.** Box plot analysis of SOX7 mRNA expression levels among ovarian cancer samples and normal peritoneum samples. A significant correlation was found between ovarian cancer and reduced SOX7 mRNA levels compared with normal control. (Normal peritoneum mean: 53.96 vs. ovarian cancer mean: 3.50 FDR =7.4e-07).

To investigate the correlation between SOX7 and the state of ovarian cancer, we compared the SOX7 gene expression profiles of forty-three serous ovarian cancers and ten peritoneum controls in GSE12470, with the false discovery rate (FDR) being controlled. We found that the expression of SOX7 was reduced significantly in ovarian cancer tissues compared with normal controls (ovarian cancer mean: 3.50 *vs.* normal peritoneum mean: 53.96, FDR =7.4e-07; Figure 1).

### Correlation of reduced SOX7 expression with tumor progression

**Figure 2:**
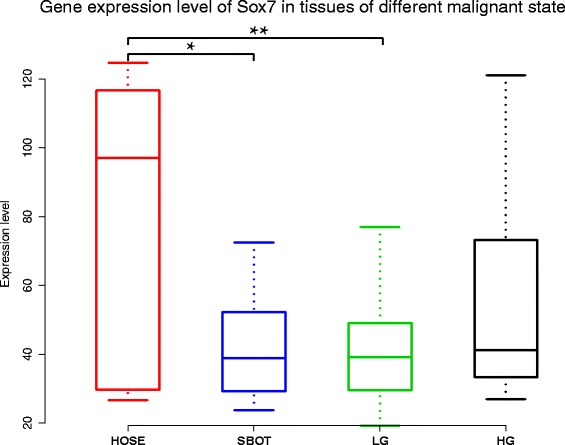
**One-way ANOVA analysis of SOX7 mRNA expression levels among HOSE, SBOT, LG and HG.** A significant correlation was found in HOSE relative to SBOT (*p* = 0.033) and HOSE compared with LG (*p* = 0.007).

We assessed the expression of SOX7 in ovarian tissues of different tumor progression states. We used the gene expression data set GSE27651, which profiled six human ovarian surface epithelia (HOSE), eight serous borderline ovarian tumors (SBOT), thirteen low-grade serous ovarian carcinomas (LG), and twenty-two high-grade serous ovarian carcinomas (HG). As highly malignant cells are believed to arise from ovarian carcinoma of low malignancy, the expression value of SOX7 should be different among tissues of different malignancy. Differences of SOX7 gene expression were observed among the four groups (*p* =0. 012) by one-way analysis of variance (one-way ANOVA). Multiple comparisons showed significant down-regulation of SOX7 mRNA expression compared to HOSE in both SBOT (SBOT mean: 42.07 *vs.* HOSE mean: 81.98, student-t *p* = 0.033) and LG (LG mean: 40.85 vs. HOSE mean: 81.98, student-t *p* = 0.007). The amount of SOX7 expression in HG in comparison with HOSE was similar, although the difference was not statistically significant (Figure 2).

### SOX7 as a negative regulator in Wnt/β-catenin pathway in ovarian cancer

**Figure 3:**
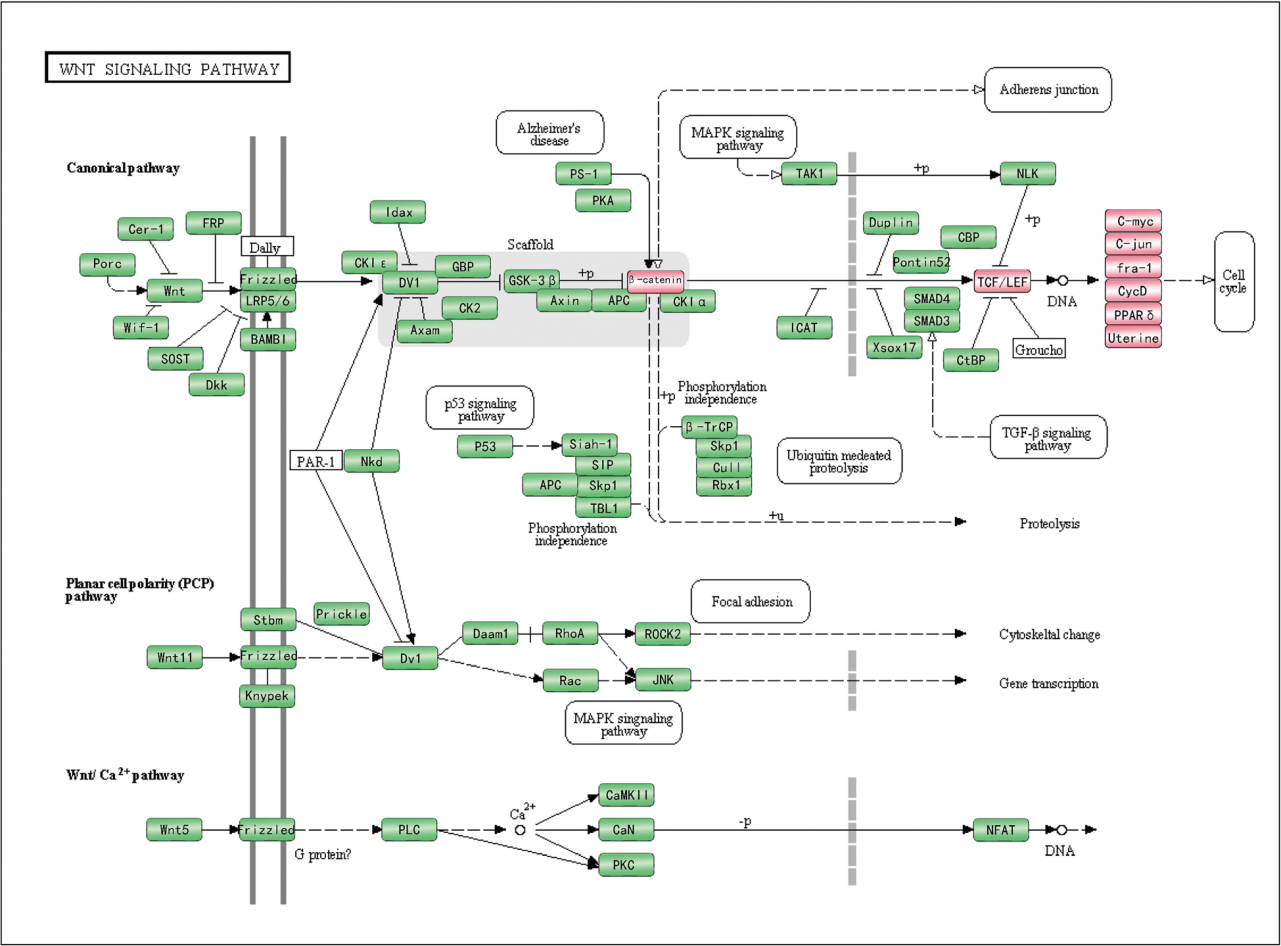
**Downstream genes chosen from KEGG annotated in Wnt/β-catenin pathway.** Downstream genes in the Wnt/β-catenin pathway are denoted in red. The figure is created based on KEGG pathway. Only a part of the pathway is shown for clarity.

It has been reported that SOX7, a member of the SOX family, functions as a transcriptional factor. To investigate the mechanism by which SOX7 is involved in the oncogenesis and progression of ovarian cancers, we analyzed genes that were co-expressed with SOX7 and short-listed 7933 genes by Pearson correlation (*FDR* < 0.01) in GSE27651 (see Additional file 1: Table S1). Using DAVID with a 5% FDR control, we found that the 7933 genes were significantly overrepresented in eleven GO-biological processes involved mainly in transcription activities (Table 1). As SOX7 has been reported to block the transcription of Wnt/β-catenin signaling pathway in various cancers, we postulated that it might have the same function in ovarian cancer. We chose thirteen genes from KEGG that were annotated in the Wnt/β-catenin pathway as downstream or pivotal hub genes (Figure 3) and calculated the Pearson correlation coefficients of expression levels between the 13 genes and SOX7 (Table 2). We found that the expression of six of the 13 selected genes was significantly correlated with that of SOX7, all negatively (binomial distribution *p =* 0.0156). Additionally, a marginal correlation of cyclinD1 with SOX7 was observed (*p =* 0.0555). Interestingly, as many as eleven of the 13 selected genes (except PPARD and TCF7L1) were negatively correlated with SOX7 (binomial distribution, *p =* 0.0112), suggesting that SOX7 could be a negative regulator of the Wnt/β-catenin pathway.

**Table 1:** **Biological processes enriched with genes dysregulated to a larger extent in ovarian cancer**

**Accession**	**Term**	***P*** **-Values**	**FDR**
GO:0006414	translational elongation	4.692062542984977E-29	9.047961108371685E-26
GO:0006412	translation	2.04912911734554E-22	3.951447873919316E-19
GO:0006396	RNA processing	2.7244884076177707E-10	5.253780188674284E-7
GO:0022613	ribonucleoprotein complex biogenesis	1.3552603187281535E-9	2.6134227004703803E-6
GO:0016071	mRNA metabolic process	3.895055800131527E-8	7.51104669238778E-5
GO:0006397	mRNA processing	5.7556740309817435E-8	1.1098975527534805E-4
GO:0000375	RNA splicing, via transesterification reactions	3.5239047199996375E-7	6.795316527141715E-4
GO:0000377	RNA splicing, via transesterification reactions with bulged adenosine as nucleophile	3.5239047199996375E-7	6.795316527141715E-4
GO:0000398	nuclear mRNA splicing, via spliceosome	3.5239047199996375E-7	6.795316527141715E-4
GO:0042254	ribosome biogenesis	5.134416394199803E-7	9.900929787476365E-4
GO:0008380	RNA splicing	1.103477760861063E-6	0.0021278751

**Table 2:** **The Pearson correlation coefficients between the expression levels of 13 genes and SOX7**

**Gene name**	**r**	***p***
**CCN-D1**	−0.2753514639	0.0555052128
**CCND2**	−0.2083737906	0.1507783665
CCND3*	−0.3789749206	0.0072460704
CTNNB1	−0.2298744109	0.1120735508
JUN*	−0.328410818	0.0212317742
**MMP7**	−0.1436013727	0.3249317121
MYC*	−0.3794897149	0.0071609446
**PPARD(NR1C2)**	0.0113166546	0.9384851238
TCF7*	−0.4721329855	0.0006148219
TCF7L2*	−0.5079689459	0.00019461
**FOSL1**	0.1976706188	0.1733712192
LEF1*	−0.4230961749	0.0024547299
TCF7L1	0.014360968	0.9219826098

Bold front denotes downstream genes in wnt signal pathway list in KEGG.

*Presents significant correlation.

### Expression of SOX7, cyclin-D1 and COX2 proteins in normal ovarian tissues, borderline ovarian tumors and ovarian cancer

**Figure 4:**
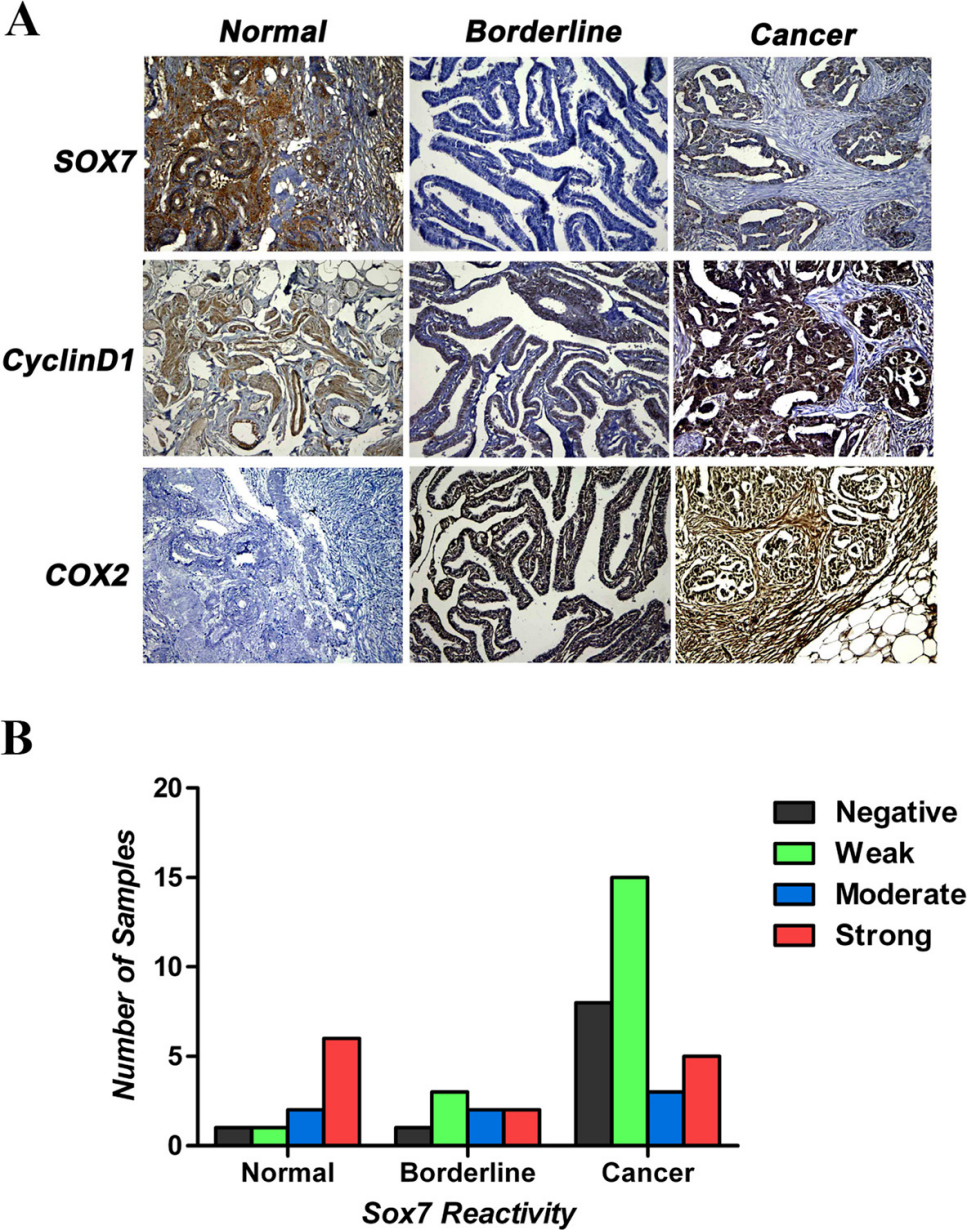
**Immunohistochemical staining for SOX7, cyclinD1 and COX2 in normal ovarian tissues, borderline ovarian tumors and ovarian cancer. A**. SOX7 strong positive staining in normal ovarian tissues and weak positive staining in borderline ovarian tumor and ovarian cancer. CyclinD1 weak positive staining in normal ovarian tissues and borderline ovarian tumor, strong positive staining in ovarian cancer. COX2 weak positive staining in normal ovarian tissues, strong positive staining in borderline ovarian tumor and ovarian cancer. **B**. SOX7 localizes to the nucleus in in every SOX7-positive ovarian tissue section. Representative of section (20 × magnification) immunohistochemical stains of normal ovarian tissues, borderline ovarian tumors and ovarian cancer with anti-SOX7 antibody shown, from left to right, negative (a), weak (b), moderate (c) and strong stain (d).

Eight of the ten analyzed normal ovarian tissue sections were positive for SOX7 immunoreactivity, with moderate to strong staining intensity (Figure 4A), but the differences among the levels of staining intensity were not statistically significant. A minority of the ovarian cancer tissue sections had positive SOX7 staining intensity, but negative or weak SOX7 staining intensity was seen in most of the malignant ovarian tissues (23/31; Figure 4B). SOX7 immunoreactivity was nucleonic in every SOX7-positive ovarian tissue section. COX2 and cyclin-D1 are the target genes in the β-catenin signal pathway. The immunoreactivity in COX2 and cyclin-D1 was crosscurrent compared to SOX7 in our research, which is consistent with the previous reports in other tissues (Figure 4A). For simultaneous comparison, each type of ovarian tissues used for photographing was from the same patient.

### Relations between SOX7 and clinical or pathological characteristics in patients with epithelial ovarian carcinoma

We analyzed the correlation between SOX7 expression and clinical or pathological characteristics of the tissues. We observed that SOX7 was down-regulated mainly in serous cystadenocarcinoma (19/31) but did not find significant differences among the pathological types (i.e., serous cystadenocarcinoma, mucinous cystadenocarcinoma, and endometrioid carcinoma). On the other hand, we found that the down-regulation of SOX7 was significantly associated with the advanced stages (III-IV; 14/31, *p* = 0.037), although no relationship was observed between SOX7 expression and other clinical features, such as pathology grade or age (Table 3). Correlation analysis showed that SOX7 expression was negatively correlated with COX2 (*r*_*s*_ = −0.618, *p* < 0.001) and cyclinD1 (*r*_*s*_ = −0.583, *p* < 0.001), two specific downstream targets in the Wnt-β-catenin pathway. The negative correlation of SOX7 with COX2 and cyclinD1 indicates a trend of inverse expression pattern of SOX7 to COX2 and cyclin D1.

**Table 3:** **Correlations of the SOX7 protein expression with the clinicopathological features of ovarian cancer and COX2/cyclinD1**

**Characteristic**	**Total**	**SOX7 expression**		***P***
		**Over-expression**	**Low-expression**	
**All cases**	31			
**Pathology type**				NS
** Serous**	26	7	19	
** Mucinous**	2	0	2	
** Endometrioid**	3	1	2	
**FIGO stage**				0.037
** Early (I-II)**	16	7	9	
** Advance (III-IV)**	15	1	14	
**Pathology grade**				NS
** Low (1)**	4	1	3	
** High (2 + 3)**	27	7	20	
**Age (years)**				NS
** <40**	0	0	0	
** 40-60**	21	5	16	
** > 60**	10	3	7	
**Cyclin D1**				r_s_ = −0.583, *p* < 0.001
** Over-expression**	26	8	18	
** Low-expression**	5	0	5	
**COX2**				r_s_ = −0.618, *p* < 0.001
** Over-expression**	27	5	22	
** Low-expression**	4	3	1	

## Discussion

Ovarian cancer remains to be a leading cause of death from gynecological malignancies. It is a huge challenge of current basic and clinical research to seek novel molecular markers for more accurate and efficient use in early diagnosis, treatment or prognosis of ovarian cancer. In this study, we chose ovarian cancer to work on primarily due to the fact that this disease is so devastating in females and that to date relatively little has been done on SOX7 in ovarian cancer. Our results obtained from different platforms indicate that the expression levels of SOX7 were significantly reduced in all types of ovarian cancers studied here, though at different extents. LG is the most malignant among ovarian cancer and may progress from SBOT, while HG, much less malignant than LG, likely develops from other kinds of precursors, such as normal epithelium of ovary or distal fallopian tube [24]. HG is a well-differentiated neoplasm and closely resembles normal tissues in many ways, so its prognosis is generally much better than LG [25]. As a result, the gene expression levels of SOX7 showed opposite tendencies to malignancy degrees. Based on the findings in this study, we propose that SOX7 is a key factor during ovarian cancer progression and is a useful prognostic marker.

Of great significance, we found that SOX7 was negatively correlated with Cyclin D1 and could be a negative regulator of the Wnt/β-catenin pathway. Cyclin D1, which controls the cell cycle, is the target gene of β-catenin and plays an important role in ovarian cancer. It is known that the Wnt/β-catenin signaling pathway is activated in epithelial ovarian cancer [26] and strongly involved in ovarian cancer development [27]. As SOX7 has been reported to block the transcriptional function of the Wnt/β-catenin signaling pathway and inhibit the activity of Wnt target genes including cyclin D1, c-Myc and COX-2 [15,17], we wondered whether there might be a correlation between SOX7 and these supposed target genes. Our results demonstrated that the expression levels of SOX7 and its targets, COX-2 and cyclin D1, have an inverse relationship, further supporting our hypothesis that SOX7 is a negative regulator in the Wnt/β-catenin signaling pathway in ovarian cancer. Although our data showed a marginal correlation of cyclin D1 with SOX7 (*p* = 0.0555) at the mRNA level, the correlation at the protein level was strong, so both had similar trends.

SOX7, a member of the SOX-F subfamily encoding transcription factors that have a pivotal role in cardiovascular development [8],has been implicated as a tumor suppressor in a variety of human cancers, e.g., colorectal, prostate, breast, liver and lung cancers [13,14,19,28]. Additionally, SOX7 may have potential usage as an independent prognostic marker in prostate and lung cancers as well as MDS patients [11,12,29]. Several lines of evidence indicate SOX7 as a negative regulator of the Wnt/β-catenin signaling pathway [14,15]. Of great interest, a recent report based on whole genomic copy number analysis demonstrates SOX7 as a novel tumor suppressor, which is silenced in the majority of non-small cell lung cancer (NSCLC) samples [19]. Molecules modulating SOX7 expression, such as miR-184 in hepatocellular carcinoma (HCC), have also been reported [28]. Another study published recently also showed that SOX7 could suppress HCC *in vivo* and *in vitro* [30]*.* There are also contradicting reports to demonstrate that SOX7 mRNA was significantly up-regulated in several human cancer cells [20]. These results have prompted us to hypothesize that SOX7 might be a true tumor suppressor but behaves differently in different cancers.

## Conclusions

Our work reported here suggests, for the first time, that SOX7 may play an important role as a tumor suppressor in ovarian cancer progression. Our results also revealed SOX7 as a negative regulator in the Wnt/β-catenin signaling pathway in ovarian cancer. Although further studies are needed to elucidate the underlining mechanisms of interactions between SOX7 and the Wnt/β-catenin signaling pathway, our result demonstrates the suppressive function of SOX7 in the carcinogenic process of ovarian cancer.

## Additional file

Additional file 1: Table S1. Short-listed 7933 genes were co-expressed with SOX7 by Pearson correlation (*FDR*< 0.01) in GSE27651.

## Abbreviations

EOC: Epithelial ovarian cancer
NSCLC: Non-small cell lung cancers
GEO: Gene Expression Omnibus
KEGG: Kyoto Encyclopedia of Genes and Genomes
DAVID: Database for Annotation, Visualization and Integrated Discovery
BPs: Biological processes
GO: Gene Ontology
FDR: False discovery rate
HOSE: Human ovarian surface epithelia
SBOT: Serous borderline ovarian tumors
LG: Low-grade serous ovarian carcinomas
HG: High-grade serous ovarian carcinomas
HCC: Hepatocellular carcinoma
