# Supplementary material for: Reduced expression of SOX7 in ovarian cancer: a novel tumor suppressor through the Wnt/β-catenin signaling pathway
Source: J Ovarian Res. 2014 Sep 5;7:87. doi: 10.1186/s13048-014-0087-1 (PMC4172779; doi:10.1186/s13048-014-0087-1)
Supplement: Additional file 1: Table S1. — Short-listed 7933 genes were co-expressed with SOX7 by Pearson correlation (FDR< 0.01) in GSE27651. [file 13048_2014_87_MOESM1_ESM.pdf]

Short-listed 7933 genes were co-expressed with SOX7 by Pearson correlation (*FDR* < 0.01) in GSE27651

Number	Gene	EntrezGene	Fdr	Correlation Coefficient
1	NAT2	10	0.000513216	0.521953398
2	AAMP	14	0.002578854	-0.463809284
3	AANAT	15	4.50E-07	0.687615139
4	ABCA2	20	0.00033142	0.535889973
5	ABCB7	22	0.000125177	-0.56417787
6	ABCF1	23	4.98E-08	0.72395679
7	ABCA4	24	5.65E-07	0.683662042
8	ABL1	25	1.88E-05	-0.612299095
9	ABO	28	0.003272509	0.454277393
10	ACAA1	30	6.94E-07	-0.679867807
11	ACACA	31	1.25E-05	0.621637458
12	ACADL	33	5.51E-06	0.639549184
13	ACADM	34	0.000898826	-0.5029226
14	ACADS	35	0.008911061	-0.410342016
15	ACADVL	37	0.001810363	-0.477308146
16	ACCN1	40	9.07E-11	0.811074864
17	ACCN2	41	2.58E-09	0.766461128
18	ACR	49	3.66E-05	0.59631723
19	ACO2	50	0.000720254	-0.510553049
20	ACRV1	56	8.94E-10	0.780831535
21	ACTB	60	0.000354905	-0.53368132
22	ACTG1	71	1.73E-05	-0.614198145
23	ACTG2	72	5.86E-09	0.755341564
24	ACTN1	87	0.000245481	-0.544983
25	ACTN2	88	2.08E-07	0.700988954
26	ACVR1B	91	0.004391957	-0.441953464
27	ACVR2A	92	5.94E-05	0.584068417
28	ACYP1	97	0.004149916	-0.444414307
29	ADAM10	102	0.00390117	-0.446948211
30	ADAR	103	8.17E-06	-0.631159613
31	ADARB1	104	6.95E-05	0.580046518
32	ADCY1	107	0.001171582	0.493387984
33	ADCY3	109	0.003990357	0.446037688
34	ADCY5	111	8.27E-05	0.575487931
35	ADCY6	112	1.25E-08	0.744756905
36	ADCY7	113	9.70E-11	0.810155776
37	ADD1	118	0.000141826	-0.560749048
38	ADD2	119	0.000764707	0.508567761
39	ADD3	120	0.000371602	-0.532206773

40	ADH1A	124	9.17E-07	0.674746156
41	ADH5	128	0.001159337	-0.493762811
42	ADORA2B	136	9.48E-10	0.779939701
43	ADPRH	141	2.63E-07	0.696942065
44	ADRA1D	146	3.13E-06	0.651155021
45	ADRA2A	150	0.003146724	0.455894529
46	ADRB2	154	0.000297284	0.539200803
47	ADRB3	155	1.15E-10	0.807913909
48	ADRBK1	156	1.32E-08	0.744086115
49	ADSL	158	0.000233606	-0.546436971
50	AP2A2	161	0.00466349	-0.439475302
51	AP1G1	164	0.000819717	0.506067723
52	AGT	183	0.002888855	0.45929497
53	AGTR2	186	5.30E-06	0.640352693
54	AGXT	189	0.004311475	0.442762568
55	AHCY	191	0.000918994	-0.502070596
56	AIF1	199	2.44E-09	0.767258041
57	AK2	204	0.001712933	-0.479431991
58	AKT1	207	0.00214153	-0.47097635
59	ALAS2	212	4.72E-08	0.724821937
60	ALB	213	0.000198494	0.551185222
61	ALDH9A1	223	0.000371591	-0.532213656
62	ABCD2	225	5.48E-05	0.586202008
63	ALDOA	226	0.000352167	-0.533938831
64	ALDOAP2	228	0.008023184	0.415381494
65	ALDOC	230	0.000678036	0.512630639
66	ALPL	249	3.62E-10	0.792503241
67	AMBN	258	4.44E-10	0.789722556
68	AMBP	259	4.02E-07	0.689669065
69	AMHR2	269	0.001411956	0.486598009
70	AMPD2	271	1.25E-05	-0.621688363
71	AMPH	273	0.000343759	0.534746474
72	ANK1	286	2.58E-09	0.766435438
73	SLC25A4	291	0.005353002	-0.433503724
74	SLC25A6	293	2.69E-05	-0.603806658
75	ANXA2P1	303	1.78E-08	0.739744162
76	ANXA6	309	5.22E-11	0.819767729
77	ANXA11	311	0.000322483	-0.536738094
78	AOC2	314	0.007320269	0.419659022
79	APBA1	320	1.09E-09	0.778008813
80	APBB2	323	9.38E-10	0.780100745
81	APC	324	5.84E-08	0.721513546
82	AIRE	326	0.002622404	0.463155035
83	APEH	327	0.000196193	-0.55151034
84	APEX1	328	0.008774718	-0.411085087

85	BIRC2	329	2.05E-05	-0.610263728
86	APLP1	333	0.003577408	-0.4506282
87	APLP2	334	0.003010668	-0.457650899
88	APOA2	336	3.80E-05	0.595402693
89	APOA4	337	4.83E-09	0.758044146
90	APOB	338	7.94E-07	0.677416267
91	APOC2	344	2.29E-10	0.798503432
92	APOC3	345	1.57E-05	0.616579624
93	APOD	347	2.47E-05	0.605815327
94	APOH	350	4.17E-10	0.790661297
95	APP	351	0.00068128	-0.512468385
96	APRT	353	0.006988612	-0.421784084
97	KLK3	354	2.05E-09	0.769558955
98	SHROOM2	357	0.007352678	-0.419410869
99	AQP2	359	0.003207647	0.455090563
100	AQP4	361	0.000239935	0.545657863
101	ARAF	369	0.000179089	-0.554146415
102	TRIM23	373	0.00024451	0.545095849
103	ARF1	375	0.000709213	-0.511092267
104	ARF3	377	0.000917216	-0.50213681
105	ARF4	378	0.001226803	-0.491777183
106	ARF6	382	0.000803459	-0.506829358
107	ARG1	383	5.94E-08	0.721205749
108	ARHGAP1	392	1.09E-05	-0.624750622
109	ARHGAP6	395	7.72E-10	0.782673705
110	RHOH	399	1.90E-08	0.738724459
111	ARL3	403	0.008241216	-0.4140694
112	ARNT	405	2.80E-06	0.653426825
113	ARR3	407	0.000575379	0.518193809
114	ARRB1	408	1.48E-11	0.840739966
115	ARSB	411	2.02E-05	0.610666581
116	ARSF	416	4.73E-06	0.642719552
117	ART1	417	5.24E-07	0.684988308
118	ART4	420	3.16E-08	0.730937731
119	ASCL1	429	1.47E-10	0.804827095
120	ASGR2	433	4.53E-10	0.789438504
121	ASIP	434	3.95E-09	0.760880086
122	ASL	435	0.004604767	-0.439996975
123	Unknown	438	2.22E-07	0.699859965
124	ASPA	443	0.000137826	0.56154436
125	ASS1	445	0.000157499	-0.557804794
126	ATF1	466	0.007243475	-0.42017957
127	ATF4	468	1.72E-05	-0.614447659
128	ATIC	471	0.000121462	-0.565021822
129	ATM	472	0.000204	0.550405005

130	RERE	473	0.000852094	-0.504765437
131	ATOH1	474	9.59E-10	0.779770451
132	ATP12A	479	1.28E-05	0.621106395
133	ATP1B3	483	0.0059645	-0.428746429
134	ATP2A3	489	0.000352592	0.533893876
135	ATP2B3	492	0.000201591	0.5507427
136	ATP4A	495	1.11E-09	0.7777615
137	ATP5A1	498	0.000365916	-0.532682668
138	ALDH7A1	501	0.004036836	-0.445539765
139	ATP5B	506	0.005240647	-0.434460621
140	ATP5C1	509	0.000707815	-0.511184966
141	ATP5D	513	0.005765078	-0.430327723
142	ATP5E	514	1.24E-05	-0.621897122
143	ATP5F1	515	0.003161139	-0.455693313
144	ATP5G1	516	0.000110789	-0.567638369
145	ATP5G2	517	4.73E-06	-0.642716703
146	ATP5I	521	0.003292861	-0.45401705
147	ATP5J	522	6.52E-05	-0.581655818
148	ATP6V0C	527	0.003784285	-0.448282508
149	ATP6V1E1	529	0.004846323	-0.437847537
150	ATP6V0B	533	0.003550669	-0.450923804
151	ATP6V1G2	534	1.01E-07	0.712790677
152	ATP6AP1	537	0.009953468	-0.405031031
153	ATP5O	539	1.27E-05	-0.621398423
154	ATR	545	0.000170876	-0.555478944
155	ATRX	546	9.66E-05	-0.571356234
156	AUH	549	0.000552061	-0.519563483
157	AUP1	550	0.006228015	-0.426894807
158	AVPR1A	552	1.46E-07	0.706970978
159	AVPR1B	553	2.12E-06	0.65898287
160	BAGE	574	2.48E-11	0.831704855
161	BAI1	575	0.0064943	-0.425096713
162	BAI3	577	3.90E-06	0.646713853
163	BAK1	578	4.33E-10	0.790065804
164	BBS1	582	0.001546962	0.483206518
165	BBS2	583	0.001497208	-0.48443261
166	BCAT2	587	0.000412513	-0.528955013
167	BCKDHA	593	1.64E-06	0.663921296
168	BCKDHB	594	2.50E-09	0.76682406
169	BCL2L1	598	0.002578619	-0.463818459
170	BCL6	604	0.004268046	-0.443190132
171	BCL8	606	2.18E-06	0.658358779
172	TNFRSF17	608	0.009412735	0.40767012
173	HCN2	610	1.12E-05	-0.624048812
174	BDKRB1	623	0.000115162	0.566526482

175	CFB	629	0.001992426	-0.473663191
176	PRDM1	639	9.29E-06	0.628299178
177	BLM	641	0.009381196	0.407855221
178	BMP3	651	0.0018637	0.476167362
179	BMP7	655	0.004962826	0.436773223
180	BMP8B	656	9.49E-10	0.779908993
181	BMPR1B	658	0.002226992	-0.469516474
182	BMX	660	4.28E-08	0.726292654
183	BPI	671	2.79E-10	0.795816885
184	BRCA1	672	0.001103145	0.495533105
185	BRAF	673	3.85E-08	0.727890562
186	BRCA2	675	0.007307286	0.419732662
187	BRDT	676	0.006309629	0.426330049
188	ZFP36L2	678	0.002148031	-0.470852464
189	BRS3	680	4.57E-06	0.643457627
190	KLF9	687	0.000189123	0.552589945
191	KLF5	688	0.000474406	-0.524518917
192	BTF3	689	0.000772296	-0.508180828
193	BTN1A1	696	3.91E-09	0.761005819
194	C3AR1	719	0.009326073	0.408121205
195	C4BPB	725	6.17E-05	0.583050525
196	CAPN5	726	0.000492557	-0.523296613
197	C5	727	2.98E-06	0.65211989
198	C8B	732	2.91E-05	0.601888927
199	OSGIN2	734	1.86E-05	0.61254226
200	C11orf2	738	1.40E-05	-0.619118123
201	MRPL49	740	0.0076713	-0.417437768
202	ZNHIT2	741	1.01E-07	0.71289592
203	C11orf10	746	5.29E-06	-0.640399691
204	C16orf3	750	1.25E-10	0.806712605
205	PTTG1IP	754	9.52E-05	-0.571767512
206	TMEM50B	757	0.003087759	-0.456688172
207	MPPED1	758	6.91E-05	0.580168726
208	CA1	759	3.03E-11	0.827351446
209	CA5A	763	0.001346186	0.488449527
210	CACNA1F	778	1.13E-11	0.85181174
211	CACNA1S	779	1.44E-09	0.774271314
212	DDR1	780	4.24E-06	-0.644968493
213	CACNB2	783	0.003653117	0.449725002
214	CACNB3	784	0.003268597	-0.454329714
215	CACNB4	785	8.78E-08	0.715035166
216	CACNG1	786	9.66E-07	0.673769278
217	S100G	795	0.001620315	0.481515357
218	CALCA	796	0.007961714	0.415733357
219	CALCB	797	0.000564593	0.518805228

220	CALCR	799	0.006875145	0.422525764
221	CALM1	801	0.008036948	-0.415299058
222	CALM2	805	0.001261288	-0.490769741
223	CALML3	810	7.59E-08	0.717320161
224	CAMK2A	815	0.000384573	0.531147415
225	CAMLG	819	5.86E-05	-0.584406124
226	CAMP	820	0.001407242	-0.486748067
227	CAPN2	824	0.002945515	-0.458526693
228	CAPZA1	829	2.53E-05	-0.605301638
229	CARS	833	5.91E-06	-0.638083931
230	CASP3	836	0.00433672	-0.442510077
231	CASP10	843	3.07E-06	0.651514084
232	CASQ1	844	4.00E-07	0.689759742
233	CASQ2	845	1.13E-09	0.777432924
234	CASR	846	1.30E-11	0.847529663
235	CAT	847	0.000605481	-0.516519587
236	CAV3	859	2.41E-09	0.767417249
237	CBFA2T3	863	2.29E-05	0.607728327
238	CBFB	865	0.002803402	-0.460445274
239	CBLN1	869	1.41E-10	0.805339268
240	CBR1	873	0.00679483	-0.42305205
241	CCK	885	0.005944332	0.428914948
242	CCKAR	886	3.99E-06	0.646246988
243	CCKBR	887	2.48E-06	0.655805448
244	KRIT1	889	0.004907678	-0.437277054
245	CCNG2	901	8.89E-05	-0.573612464
246	CCNT1	904	1.70E-10	0.802508181
247	CCNT2	905	0.000610386	-0.516240408
248	CCT6A	908	0.000104683	-0.569229248
249	CD1A	909	1.59E-07	0.705510154
250	CD1B	910	1.78E-09	0.771398247
251	CD2	914	6.63E-08	0.719437747
252	CD3E	916	1.44E-09	0.774305762
253	CD3G	917	0.00588508	0.429358406
254	CD247	919	3.34E-05	0.598539449
255	CD4	920	1.89E-09	0.770649374
256	MS4A1	931	4.04E-07	0.689519045
257	CD28	940	0.00433188	0.442555578
258	CD80	941	2.26E-10	0.798816228
259	CD86	942	6.70E-08	0.719270653
260	TNFSF8	944	5.34E-11	0.819236511
261	CD33	945	0.004574277	0.440266113
262	CD36	948	0.000396831	0.530186798
263	SCARB2	950	8.73E-05	-0.57407862
264	CD37	951	4.54E-05	0.590960601

265	ENTPD2	954	2.69E-11	0.829775361
266	ENTPD6	955	5.71E-07	0.683461601
267	ENTPD5	957	4.05E-11	0.823054715
268	CD40LG	959	6.42E-08	0.719927253
269	CD47	961	0.002757868	-0.461090854
270	CD53	963	1.05E-09	0.778505607
271	CD59	966	0.00139464	-0.487087146
272	CD63	967	0.002098173	-0.471760859
273	CD68	968	2.58E-05	0.604841834
274	CD72	971	0.00028189	0.540806227
275	CD74	972	0.008709631	-0.411405184
276	CD151	977	0.003129414	-0.456122963
277	CDA	978	1.38E-05	0.619457631
278	7-Sep	989	0.000986334	-0.499539677
279	CDC34	997	0.004448609	-0.441411553
280	CDC42	998	0.006536433	-0.424804311
281	CDH1	999	0.000772296	-0.508177945
282	CDH4	1002	2.02E-08	0.737885797
283	CDH7	1005	4.09E-11	0.822942767
284	CDH9	1007	0.000825286	-0.505837845
285	CDH10	1008	2.33E-05	0.607319377
286	CDH17	1015	3.91E-07	0.69014225
287	CDK2	1017	2.27E-06	0.657503874
288	CDK8	1024	0.000401231	-0.529822765
289	CDKN1B	1027	0.002453489	-0.465825141
290	CDKN2D	1032	0.001543183	0.483327136
291	CDSN	1041	2.06E-11	0.835063798
292	CDX1	1044	7.98E-05	0.576449972
293	CDX2	1045	1.15E-05	0.62360836
294	CDX4	1046	0.001133176	0.494595853
295	CELP	1057	1.64E-07	0.704998198
296	CETN1	1068	5.26E-08	0.723101173
297	CETP	1071	3.18E-10	0.794207112
298	CFL1	1072	0.002230963	-0.469429986
299	CFTR	1080	0.007358482	0.419370058
300	CGA	1081	1.62E-10	0.803405976
301	CEACAM3	1084	1.68E-05	0.614961411
302	CEACAM8	1088	4.51E-05	0.591140532
303	CHAT	1103	4.97E-06	0.641697789
304	RCC1	1104	0.007258204	-0.420028699
305	CHD2	1106	0.00139785	-0.486998017
306	CHD4	1108	0.000439499	-0.52694009
307	FOXN3	1112	4.73E-08	0.724763361
308	CHIT1	1118	0.002274954	0.468671746
309	CHKA	1119	0.009856772	-0.405500457

310	CHRM2	1129	4.90E-09	0.757819974
311	LYST	1130	0.001674556	0.480249051
312	CHRM5	1133	1.20E-10	0.807294174
313	CHRNA1	1134	6.47E-09	0.754006276
314	CHRNA2	1135	0.000260829	0.543138342
315	CHRNA3	1136	1.06E-10	0.808899514
316	CHRNA4	1137	0.001265498	0.490632288
317	CHRNA5	1138	1.05E-10	0.809181381
318	CHRNB3	1142	2.82E-08	0.732630871
319	CHRNB4	1143	2.89E-06	0.652753435
320	CHRND	1144	1.08E-08	0.746902653
321	CHUK	1147	1.23E-05	0.622055273
322	CIRBP	1153	0.007329731	-0.419588467
323	TBCB	1155	0.000703484	-0.511375875
324	CKM	1158	0.000103273	0.569603058
325	ERCC8	1161	0.000832787	0.505528621
326	AP2S1	1175	0.002721539	-0.46163833
327	AP3S1	1176	0.005342015	-0.433590217
328	CLCN3	1182	0.001905342	-0.475337954
329	CLCN5	1184	0.000465577	0.525140034
330	CLU	1191	0.008965302	-0.410033894
331	CLIC1	1192	0.008416687	-0.413046634
332	CLK3	1198	0.000907551	0.502540913
333	CLNS1A	1207	1.72E-05	-0.614453635
334	CLTA	1211	0.009093825	-0.409320115
335	CLTB	1212	1.72E-05	0.614401791
336	CLTC	1213	0.003167515	-0.455602034
337	CCR3	1232	0.001001241	0.499005296
338	CCR5	1234	0.000562229	0.518942708
339	CCR8	1237	8.12E-09	0.75080501
340	CCBP2	1238	1.05E-06	0.672185432
341	CMKLR1	1240	1.50E-10	0.804490393
342	LTB4R	1241	0.000144416	0.56025263
343	CNGA1	1259	8.94E-08	0.714748715
344	CNGA3	1261	8.79E-06	0.62955651
345	PLK3	1263	0.002495217	0.465122405
346	CNR1	1268	8.87E-07	0.675341208
347	CNR2	1269	3.03E-09	0.764422469
348	COL4A2	1284	1.89E-06	0.661180964
349	COL4A3	1285	4.05E-07	0.68948394
350	COL4A4	1286	0.009321524	0.408145784
351	COL9A2	1298	8.19E-07	0.676873895
352	COL11A2	1302	3.77E-11	0.824352009
353	COL15A1	1306	0.000767778	0.508391505
354	COMT	1312	0.002757868	-0.461083749

355	COPA	1314	0.00595928	-0.428810288
356	COPB1	1315	8.15E-06	-0.63121269
357	Unknown	1325	0.000257941	0.543450932
358	COX4I1	1327	0.001011716	-0.498632517
359	COX5B	1329	4.61E-05	-0.590509161
360	COX6B1	1340	0.000333676	-0.535657688
361	COX6C	1345	0.000913279	-0.502317662
362	COX7A2	1347	0.001928709	-0.474885378
363	COX7B	1349	0.00145813	-0.485442881
364	COX7C	1350	0.000171635	-0.555353713
365	COX8A	1351	0.00717469	-0.420619445
366	CPA2	1358	0.00813132	0.414703434
367	CPA3	1359	2.41E-08	0.735160084
368	CPB2	1361	0.000343513	0.534772882
369	CPD	1362	0.00024704	0.544780091
370	CPM	1368	0.000350088	0.534130191
371	CPN1	1369	4.65E-09	0.758591977
372	CPN2	1370	0.001372157	0.487704552
373	CR1	1378	2.96E-06	0.652318719
374	CR2	1380	1.98E-05	0.611099715
375	CREB1	1385	0.002077367	-0.472135185
376	ATF2	1386	0.000343759	-0.534738225
377	CREBBP	1387	0.002726008	-0.4615609
378	CRH	1392	5.96E-05	0.583982092
379	CRHBP	1393	2.92E-07	0.695186363
380	CRHR2	1395	4.73E-09	0.758311528
381	CRKL	1399	0.002871319	-0.459543423
382	CRMP1	1400	2.12E-06	0.658903259
383	CRYBA4	1413	5.08E-08	0.723669039
384	CRYBB1	1414	1.49E-10	0.804636973
385	CRYBB3	1417	1.05E-10	0.809126502
386	CRYGA	1418	5.02E-12	0.863225926
387	CRYGB	1419	2.21E-10	0.799139391
388	CRYGS	1427	0.00363614	0.449921715
389	CS	1431	0.000582009	-0.517830665
390	CSF1	1435	1.11E-06	0.671205744
391	CSF3R	1441	5.71E-05	0.585126701
392	CSH1	1442	0.008850185	0.410664634
393	CSH2	1443	1.58E-05	0.616304081
394	CSHL1	1444	2.36E-07	0.698786154
395	CSK	1445	4.48E-09	0.759158505
396	CSN1S1	1446	3.87E-05	0.594899256
397	CSN2	1447	0.001600511	0.481958494
398	CSN3	1448	2.08E-08	0.737441574
399	CSNK1A1	1452	0.000307041	-0.538204966

400	CSNK1D	1453	1.75E-06	-0.662604034
401	CSNK1G2	1455	0.00056596	-0.518712532
402	CSNK1G3	1456	0.002757868	-0.461085252
403	CSNK2A2	1459	0.003810747	-0.44798248
404	CSNK2B	1460	0.001591642	-0.482158189
405	SLC25A10	1468	0.00124594	-0.491200962
406	CST3	1471	0.008240788	-0.414076581
407	CST5	1473	1.05E-10	0.809093373
408	CSTF3	1479	0.000415162	-0.528753969
409	CTBP1	1487	1.48E-05	-0.61783652
410	CTBP2	1488	7.27E-05	-0.578856219
411	CTLA4	1493	1.29E-09	0.775668474
412	CTNNA1	1495	0.000935714	-0.501398877
413	CTRL	1506	7.65E-06	0.632627805
414	CTSG	1511	1.30E-05	0.620819411
415	CTSH	1512	0.009263577	-0.408470025
416	Unknown	1518	7.21E-06	0.633859614
417	CTSS	1520	0.000699749	0.511566292
418	CTSW	1521	3.93E-08	0.727591186
419	CTSZ	1522	2.12E-10	0.799630425
420	CUX1	1523	0.006750202	-0.423360755
421	CXADR	1525	3.32E-05	-0.598748122
422	TEX28	1527	5.45E-10	0.787121094
423	CYBB	1536	3.82E-07	0.690573646
424	CYC1	1537	0.00679483	-0.423055853
425	CYLC1	1538	7.52E-10	0.783062921
426	CYLC2	1539	0.006127589	0.427601541
427	CYP1A1	1543	5.64E-05	0.585421393
428	CYP1A2	1544	7.07E-07	0.679489528
429	CYP2A6	1548	9.77E-10	0.779484345
430	CYP2B6	1555	2.37E-07	0.698734854
431	CYP2B7P1	1556	5.99E-11	0.817317861
432	CYP2C19	1557	0.000302861	0.538591139
433	CYP2C9	1559	2.21E-10	0.799079051
434	CYP2C18	1562	6.40E-11	0.81579876
435	CYP2E1	1571	8.10E-11	0.812632707
436	CYP2J2	1573	0.000830604	0.505623959
437	CYP3A4	1576	2.04E-05	0.610401149
438	CYP4A11	1579	6.70E-08	0.71928404
439	CYP11B1	1584	1.78E-08	0.739778864
440	CYP17A1	1586	7.90E-09	0.751214155
441	ADAM3A	1587	2.45E-07	0.698140058
442	CYP19A1	1588	0.005313175	0.433853334
443	CYP21A2	1589	6.62E-10	0.784627723
444	CYP27B1	1594	3.23E-08	0.730559621

445	CYP51A1	1595	4.06E-06	0.645879627
446	DAB1	1600	2.62E-11	0.830274482
447	DAG1	1605	3.46E-05	-0.59769029
448	DGKA	1606	0.003270579	0.45430327
449	DGKB	1607	0.000522448	0.521348058
450	DGKG	1608	5.92E-07	0.682787232
451	DGKQ	1609	0.00300601	-0.457709549
452	DAO	1610	0.000149577	0.559272403
453	DARS	1615	0.000192192	-0.552107655
454	DAXX	1616	5.91E-08	0.72131383
455	DBH	1621	9.25E-07	0.674594019
456	DBI	1622	0.000177094	-0.554475707
457	DBT	1629	0.002492926	-0.465174934
458	DCC	1630	2.81E-05	0.602675431
459	DCI	1632	0.000193715	-0.551886931
460	DCTD	1635	0.000105246	-0.569041677
461	DCT	1638	5.96E-05	0.583954629
462	DDB1	1642	0.000117771	-0.565900807
463	AKR1C2	1646	0.005541189	0.432018157
464	DDIT3	1649	0.000492234	0.523326984
465	DDOST	1650	6.73E-05	-0.580830155
466	DDT	1652	0.004902882	-0.437323348
467	DDX3X	1654	0.005222912	-0.434601408
468	DDX5	1655	2.15E-05	-0.609221944
469	DDX6	1656	9.12E-06	-0.628672072
470	DHX8	1659	1.40E-05	0.619034611
471	DDX10	1662	0.008108768	-0.414858454
472	DECR1	1666	0.001869953	-0.476041938
473	DEFB4	1673	6.02E-11	0.817133407
474	DFFB	1677	0.006951253	-0.422015406
475	COCH	1690	2.13E-05	0.609423038
476	DHCR24	1718	0.000346813	0.534442807
477	LOC1720	1720	0.000406926	0.529385785
478	DHODH	1723	4.03E-10	0.791070478
479	DHPS	1725	0.000656845	-0.513698932
480	CYB5R3	1727	0.001367532	-0.487849564
481	DKC1	1736	0.001150844	-0.49402888
482	DLG2	1740	0.00193483	0.474770931
483	DLX3	1747	1.19E-05	0.622859903
484	DLX4	1748	5.68E-08	0.721929826
485	DLX6	1750	0.000111782	0.567363429
486	DNM1	1759	0.000697312	0.511679741
487	DMWD	1762	2.48E-09	0.767005342
488	DNAH6	1768	9.09E-08	0.71444975
489	DNAH8	1769	4.83E-05	0.589309479

490	DNASE1L1	1774	4.89E-08	0.724292739
491	DNASE1L3	1776	3.22E-08	0.730629737
492	DYNC1H1	1778	0.000375389	-0.531905114
493	DYNC1I2	1781	0.000871208	-0.503971372
494	DYNC1LI2	1783	0.000468739	-0.524926662
495	DNM2	1785	4.77E-10	0.788774192
496	DOCK1	1793	0.000210867	0.549449464
497	DOCK2	1794	1.07E-10	0.808771227
498	DOK1	1796	0.004897427	-0.437369327
499	DOM3Z	1797	2.22E-05	0.608485506
500	DPAGT1	1798	0.000770368	-0.508269023
501	DPH2	1802	0.002593196	-0.463595424
502	DPT	1805	0.001438898	0.485934917
503	SLC26A3	1811	0.00015756	0.557788743
504	DRD5	1816	5.77E-11	0.817967271
505	DRG2	1819	2.55E-08	0.734202079
506	DRP2	1821	4.33E-05	0.592101359
507	DSC1	1823	4.31E-10	0.790121205
508	RCAN1	1827	2.43E-08	0.734962615
509	DSG3	1830	1.24E-10	0.806898433
510	DSPP	1834	0.00037035	-0.532311189
511	DTX1	1840	0.000239942	0.545649936
512	DUSP6	1848	0.003830033	-0.447739654
513	DUT	1854	0.000128429	-0.563461692
514	DVL1	1855	0.003829154	-0.447754002
515	DVL2	1856	0.001467766	-0.485189916
516	DYRK1A	1859	0.003506256	-0.451456373
517	E2F4	1874	2.00E-10	0.800400746
518	E4F1	1877	0.002878508	-0.45943939
519	EBF1	1879	0.008158352	0.41454261
520	EBI2	1880	2.35E-07	0.698917993
521	ECHS1	1892	0.000192181	-0.55211607
522	EDA	1896	6.98E-10	0.783960598
523	EDG3	1903	1.41E-07	0.707528879
524	PHC2	1912	0.000837503	-0.50533764
525	EEF1A1	1915	0.000817421	-0.506162522
526	EEF1B2	1933	0.000181735	-0.553721965
527	EEF1D	1936	3.12E-06	-0.651188468
528	EEF1G	1937	0.000478177	-0.52426636
529	EEF2	1938	0.000136005	-0.561913263
530	EFNA1	1942	0.000466813	-0.525059879
531	EFNA3	1944	3.89E-07	0.690252291
532	CELSR2	1952	0.001308557	-0.489444316
533	EGFR	1956	2.14E-08	0.736942552
534	EIF2S1	1965	2.81E-06	0.6533456

535	EIF2B1	1967	0.000274675	-0.541602123
536	EIF2S3	1968	0.002471232	-0.465545025
537	EPHA2	1969	0.005792765	-0.430101594
538	EIF4A1	1973	0.000493394	-0.523224943
539	EIF4A2	1974	0.000332638	-0.535783908
540	EIF4B	1975	0.005221335	-0.434617796
541	EIF4G1	1981	0.008682404	-0.41155454
542	EIF4G2	1982	0.00018593	-0.55306355
543	EIF5	1983	0.001545894	-0.48324347
544	ELAVL2	1993	2.46E-09	0.767119755
545	ELAVL4	1996	2.83E-10	0.795604292
546	ELF4	2000	0.000489711	-0.523495396
547	ELF5	2001	0.000111782	0.567365351
548	ELK1	2002	6.61E-05	0.58131162
549	MARK2	2011	0.001111577	0.495259945
550	EMX1	2016	6.08E-07	0.682326886
551	CTTN	2017	9.88E-05	-0.570779205
552	EN1	2019	5.17E-11	0.820026745
553	ENSA	2029	0.000819756	-0.506060565
554	EP300	2033	0.000302056	-0.538699102
555	EPB41	2035	5.77E-05	-0.584836277
556	EPHA1	2041	3.13E-09	0.763961891
557	EPHA7	2045	2.25E-05	0.608103599
558	EPHA8	2046	1.44E-09	0.774313823
559	EPHB2	2048	7.55E-09	0.751893578
560	EPHX2	2053	0.001827603	-0.476934714
561	EPRS	2058	0.000489072	-0.523544083
562	EPS15	2060	0.000965556	-0.500274893
563	NR2F6	2063	0.005334206	-0.433660051
564	ERBB3	2065	0.002385194	-0.46695668
565	EYA4	2070	0.003403783	0.452649522
566	ERCC3	2071	0.000167441	-0.556063027
567	ERH	2079	0.000363367	-0.532885705
568	ERN1	2081	2.81E-08	0.732717021
569	FBL	2091	0.000683793	-0.512343712
570	ESR1	2099	0.000236973	-0.54600706
571	ESRRA	2101	1.32E-06	0.668011772
572	ETF1	2107	0.001515356	-0.483983466
573	ETFA	2108	0.007228698	-0.420278963
574	ETFB	2109	0.001431666	-0.486105101
575	ETV1	2115	1.76E-07	0.703847331
576	ETV5	2119	0.003023156	-0.457492285
577	ETV6	2120	0.008551076	-0.412274007
578	EVI2B	2124	8.31E-06	0.630777531
579	EWSR1	2130	2.53E-06	-0.655385261

580	EXT2	2132	0.000611812	-0.516161496
581	EYA1	2138	1.11E-06	0.671274606
582	EZH1	2145	1.82E-09	0.771106976
583	F5	2153	0.000665078	0.513249782
584	F7	2155	2.64E-10	0.79658778
585	F9	2158	0.005693921	0.430838358
586	F11	2160	7.76E-07	0.67782993
587	F12	2161	9.83E-08	0.713285436
588	F13B	2165	0.005810155	0.429968608
589	FAAH	2166	0.001039742	-0.497619572
590	FANCC	2176	3.52E-10	0.792901967
591	FANCD2	2177	0.000244368	0.545120882
592	ACSL3	2181	0.001008891	-0.498728516
593	BPTF	2186	0.006192337	-0.427137616
594	FANCF	2188	0.002093429	-0.471842627
595	FAT	2195	0.009153005	-0.409021109
596	FAU	2197	4.57E-06	-0.643431975
597	FCAR	2204	0.003868364	0.447317248
598	MS4A2	2206	0.003682828	0.44939199
599	FCER1G	2207	2.21E-10	0.79909665
600	FCER2	2208	1.78E-06	0.662262381
601	FKTN	2218	7.78E-08	0.716959587
602	FDFT1	2222	0.00388335	-0.44715339
603	FDPS	2224	2.99E-05	-0.601248102
604	FECH	2235	0.008837722	-0.410740377
605	FGA	2243	2.02E-09	0.769777365
606	FGD1	2245	5.58E-11	0.818349364
607	FGF10	2255	0.000128731	0.563391054
608	FGF11	2256	1.15E-05	0.62363084
609	FGFR1	2260	0.005206971	-0.434735125
610	FGR	2268	6.54E-11	0.815542802
611	FH	2271	0.000519114	-0.52158276
612	FIGF	2277	0.000494799	0.52312702
613	FKBP1A	2280	0.004471887	-0.441191252
614	FKBP2	2286	0.001837339	-0.476740262
615	FKBP3	2287	0.000361623	-0.53307268
616	FOXF1	2294	6.04E-09	0.754941979
617	FOXF2	2295	9.07E-11	0.811150222
618	FOXD4	2298	0.001264589	0.490665905
619	FOXI1	2299	0.007035297	-0.421496307
620	FOXE3	2301	2.28E-06	0.65742917
621	FOXC2	2303	1.70E-09	0.772038946
622	FOXO1	2308	5.24E-08	0.723180721
623	FOXO3	2309	2.18E-05	-0.608886415
624	FLI1	2313	2.82E-10	0.795635433

625	MLANA	2315	0.002914984	0.458932556
626	FLNB	2317	0.000720718	-0.510512113
627	FLT3	2322	1.26E-08	0.744639438
628	FMO2	2327	1.55E-10	0.804034436
629	FMO4	2329	0.00036806	0.532505857
630	AFF2	2334	8.11E-11	0.812597383
631	FNTA	2339	0.000283119	-0.540667829
632	FOLR2	2350	0.003168789	0.455582734
633	FPR1	2357	0.007129778	0.420917975
634	FPRL1	2358	3.79E-08	0.728104367
635	FPRL2	2359	1.05E-05	0.625592126
636	FXN	2395	1.58E-05	0.616317391
637	FRAP1	2475	9.48E-05	0.571887058
638	FRG1	2483	0.00095474	-0.500649274
639	FSHR	2492	1.18E-08	0.745543983
640	FUCA2	2519	0.002726579	-0.461548356
641	FUT2	2524	0.002997626	-0.45781639
642	FUT3	2525	6.12E-11	0.816717559
643	FUT4	2526	7.62E-09	0.751752785
644	FUT6	2528	2.26E-10	0.798736765
645	FUT7	2529	0.006522621	0.424895058
646	G6PC	2538	5.13E-10	0.787859852
647	XRCC6	2547	2.51E-05	-0.605517528
648	GAB1	2549	2.68E-08	0.733470167
649	GABBR1	2550	3.87E-05	0.594914678
650	GABRA1	2554	5.01E-08	0.723872144
651	GABRA2	2555	8.44E-08	0.715681871
652	GABRA3	2556	1.72E-05	0.614337666
653	GABRA4	2557	1.42E-05	0.618774237
654	GABRA6	2559	1.28E-07	0.709047276
655	GABRB2	2561	0.004770057	0.438489463
656	GABRB3	2562	0.000229882	0.546912798
657	GABRG2	2566	8.60E-05	0.574483307
658	GABRG3	2567	0.001032796	0.497880468
659	GABRR2	2570	0.001206418	0.492341406
660	GAD1	2571	0.000108561	0.568203923
661	GAD2	2572	2.14E-11	0.834423082
662	GAK	2580	0.000997636	-0.499137462
663	GALE	2582	0.00296182	-0.458298874
664	B4GALNT1	2583	1.11E-05	0.624448523
665	GALNS	2588	0.000815516	0.506260619
666	GALNT1	2589	0.007196303	-0.420477778
667	GALNT2	2590	0.000636212	-0.514775068
668	GALNT3	2591	0.001705482	-0.479599994
669	GANC	2595	9.60E-11	0.810302391

670	GAP43	2596	0.005198458	0.434824475
671	LRRC32	2615	0.006102597	-0.427770032
672	GARS	2617	0.002045325	-0.472710071
673	GAS6	2621	0.00012286	-0.564701919
674	GAS8	2622	9.79E-07	0.673528053
675	GATA4	2626	0.000278302	0.541198233
676	Unknown	2630	0.000227278	-0.547255258
677	GBAS	2631	0.000247199	-0.544748115
678	GBE1	2632	0.005483943	-0.432456211
679	GBP2	2634	0.000111782	0.567366446
680	GC	2638	7.37E-09	0.75225001
681	GCHFR	2644	3.30E-05	-0.598875402
682	NR6A1	2649	3.14E-11	0.826912852
683	GDF9	2661	1.89E-11	0.836161962
684	GDI2	2665	0.004355821	-0.442316796
685	GFAP	2670	4.04E-07	0.689546489
686	GFI1	2672	1.28E-05	0.621153602
687	GFRA3	2676	2.27E-10	0.798695072
688	GGT1	2678	5.41E-11	0.819067348
689	GGTLA1	2687	9.14E-10	0.780484103
690	GH1	2688	8.49E-08	0.715570169
691	GH2	2689	0.003765489	0.448490667
692	GHRH	2691	2.08E-06	0.659314512
693	GHRHR	2692	0.002778012	0.46077165
694	GHSR	2693	6.17E-05	0.583049102
695	GJB3	2707	1.69E-10	0.80277833
696	GJB5	2709	0.001696546	0.479791145
697	GK2	2712	5.01E-11	0.820442508
698	GPC3	2719	1.45E-08	0.742655425
699	GLB1	2720	0.00406501	-0.4452437
700	GCLC	2729	1.17E-07	0.710438644
701	GLP1R	2740	6.90E-09	0.753137595
702	GLRA1	2741	3.91E-05	0.59467194
703	GLRA2	2742	5.59E-05	0.585708356
704	GLUL	2752	0.003829154	-0.447753118
705	GMFB	2764	3.06E-07	0.694317536
706	GML	2765	2.10E-08	0.737228759
707	GNA11	2767	0.004853628	-0.437771431
708	GNAI2	2771	1.57E-05	0.616455139
709	GNAO1	2775	7.53E-09	0.751949147
710	GNAQ	2776	0.00353755	-0.451085845
711	GNAS	2778	0.00049332	-0.523235115
712	GNAT2	2780	0.004217852	0.443740752
713	GNB1	2782	0.003817073	-0.447913063
714	GNG5	2787	0.003550186	-0.450933933

715	GNG7	2788	0.00599227	0.428545645
716	GNG10	2790	0.000779761	-0.507836114
717	GNGT2	2793	1.55E-11	0.839666334
718	GNRHR	2798	0.000761576	0.508705987
719	GNS	2799	0.006529934	-0.4248473
720	GOLGA1	2800	7.41E-05	0.578351244
721	GOLGA4	2803	1.33E-05	-0.620326528
722	GOLGB1	2804	0.000354403	-0.533742264
723	GP1BA	2811	7.28E-11	0.814078176
724	GP2	2813	5.40E-07	0.684443759
725	GPD2	2820	0.005922723	-0.429076744
726	GPI	2821	0.009050674	-0.409555213
727	GPR4	2828	7.93E-09	0.751126697
728	XCR1	2829	0.000836015	0.505402056
729	GPR6	2830	2.63E-07	0.696945521
730	NPBWR2	2832	3.17E-10	0.794243368
731	CXCR3	2833	2.41E-08	0.73510227
732	PRLHR	2834	0.003287068	0.454085463
733	GPR12	2835	0.000250719	0.54429203
734	UTS2R	2837	0.008922793	0.410268521
735	GPR17	2840	0.000856336	0.504594666
736	GPR21	2844	0.001298663	0.489708072
737	GPR26	2849	0.007491313	0.418546581
738	GPER	2852	4.46E-09	0.75924854
739	GPR34	2857	0.009487732	0.40731699
740	MLNR	2862	0.00464007	0.439679312
741	GRK4	2868	0.000114431	0.566707795
742	GRK5	2869	5.18E-07	0.685202805
743	GPS1	2873	2.18E-05	-0.608900778
744	GPX1	2876	0.000553232	-0.519491274
745	GPX2	2877	1.55E-05	0.616789039
746	GPX4	2879	0.004387168	-0.441998096
747	GPX5	2880	2.40E-07	0.698503837
748	GRB2	2885	2.12E-06	-0.658960504
749	GRB7	2886	0.00024674	-0.544830066
750	GRIA4	2893	0.001729077	0.479041299
751	GRID1	2894	3.68E-05	0.596179076
752	GRIK1	2897	1.36E-05	0.619766552
753	GRIN1	2902	2.60E-09	0.766303877
754	GRIN2B	2904	1.68E-06	0.66333094
755	GRIN2D	2906	2.60E-06	0.654862208
756	GRM1	2911	0.006005482	0.428431622
757	GRM2	2912	4.84E-06	0.64224577
758	GRM3	2913	0.00110294	0.495544453
759	GRM4	2914	0.006769139	-0.423238266

760	GRM6	2916	3.23E-06	0.650490641
761	GRM8	2918	2.66E-07	0.696765107
762	GRPR	2925	6.40E-10	0.7850539
763	GRSF1	2926	0.001756168	-0.478445817
764	GSK3B	2932	0.000832361	-0.505555399
765	GSPT1	2935	0.003494204	-0.451593098
766	GSS	2937	0.002279546	-0.468588262
767	GSTA1	2938	1.72E-09	0.771850618
768	GSTM4	2948	4.41E-07	0.687984706
769	GSTM5	2949	0.000655318	0.513801604
770	GSTZ1	2954	0.003077205	-0.456822455
771	GTF2B	2959	0.00644232	-0.425464054
772	GTF2I	2969	0.001366877	-0.4878702
773	GTF3A	2971	0.000260862	-0.543122707
774	BRF1	2972	4.52E-10	0.789502795
775	GTF3C1	2975	0.000146434	-0.559886405
776	GTF3C2	2976	0.000101313	-0.570093385
777	GUCA1A	2978	1.35E-07	0.708122186
778	GUK1	2987	0.002490401	-0.465219916
779	GUSB	2990	1.78E-05	-0.613635077
780	GYPA	2993	0.000337691	0.535303416
781	Unknown	2996	6.09E-05	0.583425183
782	GYS1	2997	1.34E-05	0.62006673
783	GYS2	2998	3.83E-09	0.761308071
784	GZMM	3004	3.74E-05	0.595808082
785	H1F0	3005	0.00296182	-0.458294022
786	HIST1H1B	3009	0.000790524	-0.507367422
787	HIST1H1T	3010	1.25E-08	0.744759203
788	H3F3B	3021	6.18E-05	-0.58302046
789	HAGH	3029	0.005342015	-0.433591013
790	HADHA	3030	0.004054053	-0.44534538
791	HADHB	3032	8.96E-05	-0.573410774
792	HADH	3033	0.001643011	-0.480981199
793	HARS	3035	0.007405783	-0.419086381
794	HBBP1	3044	2.46E-05	0.605913698
795	HBE1	3046	3.28E-07	0.693128964
796	HCCS	3052	0.008967197	-0.410016925
797	SERPIND1	3053	1.00E-05	0.626595876
798	HCFC1	3054	4.07E-06	0.645834502
799	HCRT	3060	1.15E-11	0.849299181
800	HD	3064	6.32E-08	0.720170616
801	HDAC1	3065	7.24E-05	-0.578970485
802	HDAC2	3066	0.000530483	-0.520832515
803	HDLBP	3069	2.31E-05	-0.60752175
804	NCKAP1L	3071	0.009760681	0.40597074

805	HEXA	3073	6.32E-05	-0.582474069
806	HEXB	3074	0.003235442	-0.454742893
807	HFE	3077	9.12E-10	0.780522679
808	HIP1	3092	0.000440843	-0.526818732
809	HINT1	3094	0.0059645	-0.428746108
810	HK1	3098	0.003109391	-0.45638809
811	HLA-DPB2	3116	1.11E-08	0.746443741
812	HLCS	3141	0.008823245	-0.410841149
813	HMBS	3145	0.000439499	0.526939593
814	HMGB1	3146	3.42E-05	-0.598023418
815	HMGB2	3148	0.007298638	-0.419783502
816	HMGN1	3150	7.13E-05	-0.579344803
817	HMGN2	3151	0.001942179	-0.474625138
818	HMGCL	3155	1.55E-06	0.664965723
819	HMGCS1	3157	0.008358249	-0.41339746
820	HMGCS2	3158	2.79E-10	0.795826248
821	HMOX1	3162	0.000185565	0.553126294
822	HMOX2	3163	0.000281421	0.540864844
823	HMX2	3167	0.002817435	0.460258689
824	HNF4A	3172	2.05E-08	0.737634943
825	SLC29A2	3177	0.003347379	0.453329026
826	HNRNPA1	3178	0.00069653	-0.511718309
827	HNRNPA2B1	3181	2.54E-05	-0.605138799
828	HNRPAB	3182	0.002861602	-0.459667885
829	HNRNPC	3183	0.000592384	-0.517247678
830	HNRPD	3184	6.44E-05	-0.58196295
831	HNRPH1	3187	0.00044058	-0.526847319
832	HNRPH3	3189	1.44E-05	-0.618485957
833	HNRPK	3190	0.000141402	-0.560842668
834	TLX1	3195	0.009692888	0.406316856
835	HOXA1	3198	5.06E-05	0.588173865
836	HOXA2	3199	0.001839404	0.476687266
837	HOXA6	3203	1.87E-07	0.702769381
838	HOXA11	3207	0.0074727	0.418676542
839	HOXB1	3211	1.44E-09	0.774287145
840	HOXB3	3213	0.0097492	-0.406033297
841	HOXC9	3225	0.001575936	0.48250799
842	HOXC10	3226	9.69E-07	0.673709797
843	HOXC11	3227	2.05E-09	0.769469198
844	HOXC12	3228	6.31E-08	0.720230775
845	HOXD9	3235	3.22E-06	0.65058622
846	HOXD13	3239	6.83E-09	0.753283713
847	HPD	3242	4.54E-09	0.758991985
848	HPX	3263	0.001764567	0.478258571
849	HRAS	3265	0.008631659	-0.41184242

850	HRBL	3268	6.19E-06	0.63712199
851	HRG	3273	0.0059645	0.428753975
852	HRH2	3274	0.000129058	0.563315652
853	HSD3B2	3284	4.01E-07	0.689683022
854	HSD17B4	3295	0.00061782	-0.515809443
855	HSF1	3297	0.005429978	-0.432875394
856	HSF2	3298	0.002766018	-0.460962303
857	DNAJA1	3301	0.00296182	-0.458297216
858	HSPA1L	3305	2.21E-08	0.736389018
859	HSPA4	3308	0.001261475	-0.490759485
860	HSPA5	3309	0.007979892	-0.415628874
861	HSPA8	3312	0.001329614	-0.488887849
862	HSPA9	3313	0.005212957	-0.434682257
863	HSPB1	3315	0.006631863	-0.424154322
864	HSP90AA1	3320	0.002108035	-0.471578865
865	HSPD1	3329	0.001267881	-0.490568095
866	HSPE1	3336	0.008165118	-0.414499852
867	NDST1	3340	0.000153648	0.558509799
868	HTN1	3346	2.34E-05	0.607142211
869	HTN3	3347	0.001235976	0.491517908
870	HTR1A	3350	3.77E-11	0.824251294
871	HTR1B	3351	1.87E-10	0.801315063
872	HTR1F	3355	0.00095385	0.500685372
873	HTR2B	3357	3.57E-05	0.596903685
874	HTR5A	3361	0.000969822	0.500101734
875	HUS1	3364	5.99E-11	0.817416157
876	IAPP	3375	1.71E-07	0.704321642
877	IARS	3376	6.47E-05	-0.581866267
878	ICA1	3382	1.76E-07	0.703856725
879	IRF8	3394	7.84E-08	0.716827457
880	ICT1	3396	0.00076564	-0.508514371
881	ID4	3400	0.001989053	-0.47374426
882	IDE	3416	0.002482008	0.465355198
883	IFI16	3428	0.004807283	-0.438184932
884	IFNA1	3439	9.49E-05	0.571838609
885	IFNA2	3440	7.05E-07	0.679561564
886	IFNA4	3441	0.005450664	0.432708221
887	IFNA5	3442	0.003039063	0.457295442
888	IFNA10	3446	4.32E-06	0.644555609
889	IFNA14	3448	2.11E-08	0.737184211
890	IFNA16	3449	0.00166612	0.48046121
891	IFNA17	3451	2.63E-07	0.696915807
892	IFNA21	3452	3.38E-06	0.649584917
893	IFNAR2	3455	0.002029749	-0.472983129
894	IFNB1	3456	4.10E-09	0.760351633

895	IFNG	3458	3.58E-05	0.596843166
896	IFNGR1	3459	0.005858996	-0.429576969
897	IFRD1	3475	0.009141092	-0.409083168
898	IGBP1	3476	0.005838584	-0.429738954
899	IGF1R	3480	0.007689475	-0.417314894
900	IGFBP7	3490	0.009820222	-0.405670272
901	Unknown	3495	5.31E-11	0.819404153
902	Unknown	3497	3.44E-06	0.649229494
903	IGJ	3512	0.002753563	0.461164664
904	IGSF1	3547	1.91E-10	0.801035202
905	IHH	3549	4.76E-10	0.788836478
906	IK	3550	0.000942052	-0.5011212
907	IKBKB	3551	0.008539672	-0.412336872
908	IL1B	3553	2.12E-05	0.609596314
909	IL1R1	3554	0.002894431	-0.459212737
910	IL2RA	3559	1.55E-10	0.804098449
911	IL2RB	3560	2.26E-10	0.798763097
912	IL2RG	3561	3.49E-10	0.793004243
913	IL3RA	3563	7.65E-06	0.632624199
914	IL4	3565	1.60E-09	0.772837565
915	IL5RA	3568	8.77E-07	0.675561557
916	IL9	3578	4.63E-06	0.643169267
917	IL9R	3581	3.80E-10	0.791842993
918	IL10RB	3588	0.001767698	-0.478190262
919	IL11	3589	5.28E-11	0.819522123
920	IL12B	3593	0.006504632	0.425026646
921	IL13RA1	3597	0.001815227	0.477199136
922	IL15RA	3601	1.54E-10	0.80416129
923	IL16	3603	4.98E-10	0.788285135
924	IL17A	3605	2.63E-05	0.604361969
925	FOXK2	3607	0.001060275	-0.496927349
926	ILF3	3609	0.00300252	-0.457754901
927	ILK	3611	0.002849911	-0.459824043
928	IMPA1	3612	0.006004879	-0.428440608
929	IMPDH2	3615	0.000250585	-0.544312191
930	IMPG1	3617	0.000276551	0.541384068
931	ING2	3622	0.007216178	-0.420356071
932	INHBC	3626	2.78E-05	0.602979348
933	INPPL1	3636	0.000107137	-0.568572857
934	INSM1	3642	0.000340192	0.535072918
935	EIF3E	3646	0.002623167	-0.46312539
936	PDX1	3651	1.25E-05	0.621628961
937	IRAK1	3654	0.004215279	-0.443779968
938	ITGA6	3655	0.007408351	-0.419067809
939	IREB2	3658	0.00134504	-0.488487443

940	IRF2	3660	0.002438934	-0.46605455
941	IRF3	3661	0.000319963	-0.536967886
942	IRF5	3663	3.86E-09	0.761204592
943	IRF7	3665	0.004909552	-0.437257818
944	ITGAD	3681	5.47E-06	0.639724353
945	ITGAE	3682	0.001471151	-0.485094702
946	ITGAM	3684	0.001946647	0.474539071
947	ITGAX	3687	1.44E-10	0.805075808
948	EIF6	3692	0.001199381	-0.492567546
949	ITGB5	3693	0.000565282	-0.518752691
950	ITIH2	3698	2.18E-08	0.736648226
951	ITK	3702	3.06E-05	0.600719845
952	STT3A	3703	1.12E-10	0.808274257
953	ITPA	3704	1.46E-05	-0.618093358
954	ITPK1	3705	0.0009278	-0.50170597
955	ITPR2	3709	0.008108768	0.414861583
956	ITPR3	3710	0.00135838	-0.488096662
957	IVL	3713	1.30E-11	0.846705244
958	JAG2	3714	0.007868522	-0.416290469
959	JUND	3727	0.000936858	-0.501334691
960	JUP	3728	0.000914347	-0.502275145
961	KARS	3735	0.000806926	-0.506684455
962	KCNA5	3741	7.15E-11	0.814260554
963	KCNA6	3742	0.001364852	0.487922331
964	KCNB1	3745	0.001067631	0.496677651
965	KCNG1	3755	0.004950862	0.436878272
966	KCNH1	3756	0.005158247	-0.43513926
967	KCNJ1	3758	8.26E-11	0.812174282
968	KCNJ3	3760	7.01E-09	0.752925727
969	KCNJ9	3765	3.76E-07	0.690877152
970	KCNJ11	3767	5.72E-10	0.786427615
971	KCNJ13	3769	1.71E-05	0.614517769
972	KCNJ15	3772	1.13E-11	0.853544555
973	KCNK2	3776	8.97E-10	0.780766523
974	KCNK3	3777	0.001982804	0.473871149
975	KCNMB1	3779	7.48E-11	0.813744804
976	KCNN1	3780	2.61E-07	0.697088631
977	KCNQ3	3786	1.12E-06	0.671012952
978	KCNS1	3787	0.000236794	0.546038951
979	KCNS2	3788	7.88E-05	0.576788306
980	KEL	3792	4.47E-11	0.821708598
981	KHK	3795	0.001351061	0.488325021
982	KIF3C	3797	0.000444857	0.526542433
983	KIF5A	3798	5.86E-09	0.75533187
984	KIR2DL1	3802	5.83E-09	0.755459427

985	Unknown	3803	1.60E-10	0.803579986
986	Unknown	3804	2.35E-07	0.698864506
987	KIR2DL4	3805	0.000823421	0.505916601
988	Unknown	3806	0.000122256	0.564836113
989	Unknown	3808	6.85E-05	0.580410302
990	KIR2DS4	3809	3.95E-07	0.689977251
991	Unknown	3810	1.13E-05	0.623993979
992	KIR3DL1	3811	1.11E-07	0.711237441
993	KLK2	3817	2.79E-11	0.829020392
994	KLRB1	3820	0.000289962	0.539957451
995	KLRD1	3824	0.000214526	0.548947642
996	KNG1	3827	1.20E-08	0.745379354
997	KLC1	3831	1.26E-05	-0.621506865
998	KIFC1	3833	0.001673595	0.480273186
999	KIF25	3834	2.31E-08	0.735772944
1000	KIF22	3835	0.008348424	-0.41345923
1001	KPNB1	3837	0.000212178	-0.549282686
1002	TNPO1	3842	0.007930866	-0.415910598
1003	RANBP5	3843	0.0003419	-0.534906697
1004	KRTAP5-9	3846	0.000337742	0.535293185
1005	KRT1	3848	0.000843574	0.505088116
1006	KRT2	3849	1.80E-07	0.703431478
1007	Unknown	3854	1.33E-11	0.843152856
1008	KRT8	3856	0.002733455	-0.461449772
1009	KRT10	3858	0.000283567	-0.540613455
1010	KRT12	3859	0.00013352	0.562403188
1011	KRT18	3875	0.001851349	-0.476433429
1012	KRT19	3880	0.005135732	-0.435321283
1013	KRT33A	3883	0.001109313	0.495339255
1014	KRT33B	3884	6.11E-05	0.583324038
1015	KRT34	3885	2.46E-08	0.734731382
1016	KRT81	3887	8.96E-06	0.629089634
1017	KRT82	3888	0.003658827	0.449654533
1018	KRT83	3889	7.35E-10	0.78334311
1019	KRT85	3891	2.85E-06	0.653075374
1020	LAG3	3902	0.004623246	0.439837602
1021	LALBA	3906	3.21E-08	0.730678296
1022	LAMA5	3911	0.005156979	-0.435153568
1023	LAMB2	3913	0.001028394	-0.498050254
1024	LAMP1	3916	0.000711138	-0.51098525
1025	RPSA	3921	2.37E-07	-0.698720245
1026	LASP1	3927	0.002784826	-0.460682764
1027	LCP1	3936	5.02E-12	0.863895447
1028	LCP2	3937	7.91E-06	0.631884481
1029	LDHC	3948	3.66E-08	0.728634635

1030	LECT2	3950	4.01E-05	0.594068439
1031	LEP	3952	0.00800038	0.415513289
1032	LGALS3BP	3959	3.18E-06	-0.650822974
1033	LIG4	3981	0.002398314	-0.466718472
1034	ABLIM1	3983	0.000560248	-0.519055191
1035	LIMK1	3984	6.65E-05	0.581142508
1036	LIMK2	3985	0.003716709	-0.449013028
1037	LIPC	3990	1.86E-05	0.612633639
1038	LLGL2	3993	0.000481956	-0.523991628
1039	LLGL1	3996	9.55E-05	0.571661869
1040	LMAN1	3998	0.001739963	-0.478805761
1041	LMX1B	4010	7.11E-06	0.634140245
1042	LOR	4014	1.05E-10	0.809007944
1043	LPA	4018	0.000186549	0.552972308
1044	LPO	4025	0.004217852	0.44374279
1045	LRMP	4033	2.02E-10	0.800284676
1046	LRCH4	4034	0.008373334	-0.413313599
1047	LRP1	4035	7.82E-10	0.78253093
1048	LRPAP1	4043	0.000288595	-0.540100743
1049	LSS	4047	0.00210099	-0.471711146
1050	LTBP2	4053	0.000147967	0.559581484
1051	LTBP3	4054	0.000160107	-0.557333978
1052	LY6E	4061	0.009400202	-0.407748035
1053	CD180	4064	3.66E-09	0.76196571
1054	LYL1	4066	0.000518216	0.521637683
1055	SH2D1A	4068	1.44E-07	0.707139214
1056	MAB21L1	4081	1.33E-06	0.667922623
1057	MXD1	4084	5.50E-08	0.722419631
1058	SMAD1	4086	0.001534774	-0.483534328
1059	SMAD2	4087	0.002891259	-0.459261089
1060	SMAD4	4089	0.004964826	-0.436753201
1061	SMAD6	4091	0.006655549	-0.423992583
1062	SMAD7	4092	0.004569283	-0.440316273
1063	Unknown	4099	0.000361663	0.533062328
1064	MAGEA10	4109	3.84E-06	0.64704268
1065	MAGEB2	4113	3.67E-07	0.691300818
1066	MAN2C1	4123	9.68E-10	0.779647571
1067	MANBA	4126	1.48E-05	-0.617807484
1068	MAP2	4133	0.000492234	0.523331029
1069	MAP6	4135	0.00724622	0.42014941
1070	MAS1	4142	6.40E-10	0.785066758
1071	MAT1A	4143	1.69E-10	0.802715277
1072	MAT2A	4144	8.60E-06	-0.630055027
1073	MATN1	4146	0.003035469	0.457346984
1074	MAX	4149	0.003421181	-0.452439245

1075	MAZ	4150	0.007993745	0.415555998
1076	MBD1	4152	0.00722909	-0.420271404
1077	MBNL1	4154	0.005841981	-0.429701502
1078	MC1R	4157	0.003713618	0.449050165
1079	MC2R	4158	1.99E-10	0.800495214
1080	MC5R	4161	1.91E-07	0.702463971
1081	MCF2	4168	9.22E-10	0.78035259
1082	MCL1	4170	0.000318174	-0.537135304
1083	CD46	4179	0.000350256	-0.534098912
1084	SMCP	4184	4.02E-11	0.823270079
1085	MDFI	4188	7.99E-09	0.751012233
1086	MDH2	4191	3.01E-05	-0.601122497
1087	ME2	4200	1.17E-07	0.710467298
1088	MEIS1	4211	0.001024216	-0.498191105
1089	MAP3K3	4215	0.001298663	-0.489711922
1090	MAP3K4	4216	0.009386992	-0.407815251
1091	MEN1	4221	0.008829656	0.410802138
1092	MEP1A	4224	1.15E-08	0.745990767
1093	MGAT1	4245	0.000408823	0.529227274
1094	MGAT2	4247	0.000273936	0.541679772
1095	MGAT5	4249	1.30E-05	0.620869205
1096	MGST2	4258	0.000345795	-0.534550577
1097	CIITA	4261	2.61E-07	0.697108089
1098	CD99	4267	0.001865375	-0.476132662
1099	MIF	4282	9.13E-05	-0.572872
1100	ATXN3	4287	5.58E-06	0.63928836
1101	MLH1	4292	4.05E-06	-0.64593083
1102	MAP3K9	4293	1.54E-09	0.773371143
1103	MLN	4295	0.002570988	0.463945667
1104	AFF1	4299	0.00145813	-0.485443766
1105	MLLT6	4302	0.005960541	-0.428797127
1106	MMP19	4327	8.07E-11	0.812690942
1107	ALDH6A1	4329	1.48E-06	0.665818947
1108	MNAT1	4331	1.28E-10	0.806410505
1109	MNT	4335	0.002651975	-0.462690464
1110	MOCS1	4337	0.008453788	-0.412811985
1111	MOCS2	4338	0.0017965	0.477579196
1112	MOS	4342	1.66E-10	0.802979379
1113	MOV10	4343	0.002750791	-0.461204344
1114	MPG	4350	0.006477599	-0.425220474
1115	MPI	4351	0.001021459	-0.498290119
1116	MPL	4352	4.40E-07	0.688040536
1117	MPP1	4354	0.000444707	0.526563833
1118	MPP2	4355	3.02E-10	0.794857111
1119	MPST	4357	0.008127391	-0.414732643

1120	MPV17	4358	1.45E-09	0.774142948
1121	MPZ	4359	1.93E-10	0.800868127
1122	ABCC1	4363	0.009815317	-0.40571071
1123	MSN	4478	0.002882885	-0.459371174
1124	MSR1	4481	8.26E-11	0.812198301
1125	MSRA	4482	1.12E-08	0.746259516
1126	MT1G	4495	0.006149948	0.427433044
1127	MTAP	4507	0.007283611	-0.419878501
1128	Unknown	4512	0.008145645	-0.414619086
1129	Unknown	4513	0.001043045	-0.497505931
1130	Unknown	4514	0.007511447	-0.418411519
1131	MTHFR	4524	2.55E-08	0.734192646
1132	MTIF2	4528	0.000302861	-0.538591054
1133	MTM1	4534	4.98E-09	0.757568163
1134	MTRR	4552	0.000288867	-0.540064557
1135	MUC3A	4584	1.78E-05	0.613669052
1136	MUC5AC	4586	1.31E-09	0.775508929
1137	MUC7	4589	9.53E-05	0.571734543
1138	MUC8	4590	2.75E-07	0.696196733
1139	TRIM37	4591	0.002548866	-0.464291596
1140	MUSK	4593	7.35E-10	0.783323748
1141	MUT	4594	0.002685526	-0.462189485
1142	MUTYH	4595	4.08E-05	0.59358926
1143	MYBPC1	4604	8.07E-06	0.631420932
1144	MYF6	4618	2.31E-11	0.832498212
1145	Unknown	4620	4.81E-05	0.589442227
1146	Unknown	4622	5.15E-08	0.723446414
1147	Unknown	4626	1.03E-09	0.778800211
1148	MYL1	4632	5.14E-06	0.641015229
1149	MYL5	4636	5.05E-10	0.788114422
1150	MYL6	4637	0.001194678	-0.492700335
1151	MYO1A	4640	3.51E-09	0.762466877
1152	Unknown	4645	0.001665938	-0.480470249
1153	MYO6	4646	0.005650141	-0.431209862
1154	MYO7A	4647	2.41E-05	0.606473378
1155	MYOC	4653	1.92E-08	0.738603953
1156	MYOG	4656	3.35E-09	0.763092997
1157	MYT1	4661	5.69E-07	0.683535529
1158	NAB1	4664	0.001780588	-0.477916733
1159	NACA	4666	3.89E-05	-0.594792793
1160	NAGA	4668	3.96E-06	0.646392544
1161	NAGLU	4669	0.005681241	-0.430942432
1162	HNRPM	4670	0.000453294	-0.525958813
1163	NAP1L1	4673	0.00782823	-0.416516217
1164	NARS	4677	8.74E-05	-0.574015523

1165	NCAM2	4685	1.03E-05	0.625960585
1166	NCF4	4689	1.12E-08	0.746324799
1167	NCL	4691	0.000267898	-0.542326234
1168	NDUFA1	4694	0.000119382	-0.565510133
1169	NDUFA2	4695	0.000242191	-0.545374668
1170	NDUFA3	4696	0.007243511	-0.420174187
1171	NDUFA4	4697	0.000617806	-0.515816
1172	NDUFA5	4698	0.001394714	-0.487079863
1173	NDUFA6	4700	0.000525614	-0.521149241
1174	NEB	4703	1.58E-06	0.664572587
1175	NDUFAB1	4706	3.73E-05	-0.595842672
1176	NDUFB2	4708	0.00093832	-0.501275088
1177	NDUFB3	4709	0.000486094	-0.523733644
1178	NDUFB4	4710	0.000185481	-0.553150288
1179	NDUFB5	4711	0.006642158	-0.424079665
1180	NDUFB7	4713	0.006660625	-0.423947936
1181	NDUFB10	4716	0.000594691	-0.517106652
1182	NDUFC1	4717	5.29E-05	-0.587082735
1183	NDUFC2	4718	0.001486691	-0.484683909
1184	NDUFS2	4720	0.000817078	-0.506180753
1185	NDUFS3	4722	3.27E-06	-0.650241997
1186	NDUFV1	4723	4.16E-06	-0.645347566
1187	NDUFS6	4726	0.00122958	-0.491690876
1188	NDUFS8	4728	0.001295397	-0.489809115
1189	NDUFV2	4729	0.000106819	-0.568668041
1190	DRG1	4733	3.23E-05	-0.599420007
1191	2-Sep	4735	0.009742754	-0.406067106
1192	RPL10A	4736	4.34E-06	-0.644465171
1193	NEFM	4741	4.12E-05	0.593337526
1194	NEFL	4747	2.28E-07	0.699384202
1195	NEO1	4756	0.001914058	-0.475167164
1196	NEU2	4759	0.003644233	0.44982739
1197	NEUROD1	4760	0.000616658	0.515906133
1198	NEUROD2	4761	1.62E-10	0.803397452
1199	NEUROG1	4762	0.006724696	-0.423533606
1200	NFATC2	4773	2.59E-05	0.604681713
1201	NFE2L1	4779	0.001206067	-0.492356037
1202	NFE2L2	4780	0.000298219	-0.539096546
1203	NFIB	4781	0.007724063	-0.417109554
1204	NFKB1	4790	1.88E-08	0.738904613
1205	NFKBIB	4793	0.001159615	-0.493749786
1206	NFKBIL2	4796	4.46E-07	0.687765569
1207	NFRKB	4798	3.43E-09	0.762772526
1208	NHLH1	4807	2.87E-11	0.828433668
1209	NHP2L1	4809	0.000302517	-0.538647623

1210	NKX2-2	4821	1.73E-05	0.61418659
1211	NKX3-1	4824	0.002202651	0.469908307
1212	NNAT	4826	7.92E-10	0.782341194
1213	NMB	4828	1.62E-07	0.705214445
1214	NMBR	4829	0.0012112	0.492213287
1215	NME3	4832	0.003913555	-0.446803665
1216	NMT1	4836	0.004767969	-0.438515968
1217	NODAL	4838	7.07E-08	0.718439989
1218	NOL1	4839	0.005534813	-0.432071423
1219	NONO	4841	0.001663157	-0.480540494
1220	NOS1	4842	1.60E-06	0.664296785
1221	CNOT2	4848	0.00904057	-0.409645583
1222	CNOT3	4849	0.004836942	-0.437923551
1223	CNOT4	4850	0.00147191	-0.485072791
1224	NOTCH2	4853	0.000149021	-0.559378324
1225	NOTCH4	4855	2.39E-06	0.656520406
1226	NOVA2	4858	0.0009278	-0.501711398
1227	NPM1	4869	0.000833623	-0.505493016
1228	NPY2R	4887	1.83E-07	0.703120367
1229	NPY6R	4888	0.000886472	0.503360029
1230	NPY5R	4889	0.001661341	0.480581095
1231	NRAP	4892	0.003594914	0.450392417
1232	NRF1	4899	0.006102597	-0.4277658
1233	NRGN	4900	2.80E-09	0.765387501
1234	NRL	4901	3.59E-10	0.792627896
1235	NSF	4905	0.00489164	-0.437420786
1236	NTRK3	4916	1.13E-11	0.851461723
1237	NTSR1	4923	1.57E-11	0.839410473
1238	NUCB1	4924	0.007336777	-0.419520308
1239	NUP98	4928	0.000391059	-0.530644175
1240	OAS3	4940	5.88E-11	0.817725456
1241	TBC1D25	4943	9.42E-11	0.81059999
1242	OAZ1	4946	0.000386835	-0.530966699
1243	OCLN	4950	0.005491482	-0.432384259
1244	ODF1	4956	1.23E-10	0.806943373
1245	ODF2	4957	0.009045521	-0.40959985
1246	OGG1	4968	0.000838042	-0.505306954
1247	OMG	4974	0.001265248	0.490644072
1248	OMP	4975	0.005075451	0.435839524
1249	OPA1	4976	2.49E-08	0.734553979
1250	OPCML	4978	0.000262128	0.542988583
1251	OPRM1	4988	1.93E-10	0.800907874
1252	OR1D2	4991	4.01E-06	0.646139683
1253	ORC4L	5000	0.000182172	-0.5536482
1254	ORC5L	5001	0.001121441	-0.494949178

1255	SLC22A18AS	5003	0.000141672	0.560788759
1256	OTC	5009	4.04E-06	0.645993633
1257	OXA1L	5018	9.00E-05	-0.573267736
1258	OXT	5020	0.008400125	0.413137501
1259	P2RX1	5023	8.99E-07	0.67511234
1260	P2RX3	5024	3.68E-05	0.596152502
1261	P2RX5	5026	4.18E-09	0.760078196
1262	P2RY4	5030	2.02E-05	0.610706708
1263	P4HB	5034	2.09E-05	-0.609838417
1264	PA2G4	5036	0.0008854	-0.503413471
1265	PEBP1	5037	0.000167261	-0.556102746
1266	PABPC3	5042	6.66E-06	-0.635559265
1267	PAFAH1B1	5048	0.000230527	-0.546827281
1268	PAFAH1B2	5049	0.000122077	-0.564878479
1269	PAFAH2	5051	0.000190837	0.552339775
1270	SERPINB2	5055	0.000108094	0.56832821
1271	PAK2	5062	0.005271363	-0.43420818
1272	PAK3	5063	2.48E-09	0.766981899
1273	PARK2	5071	4.57E-09	0.7588334
1274	PAX1	5075	0.000216634	0.548656663
1275	PAX3	5077	2.31E-05	0.607463754
1276	PAX5	5079	4.13E-10	0.790810122
1277	PAX6	5080	0.004765977	0.438541754
1278	PAX7	5081	1.75E-05	0.61401943
1279	PAX9	5083	5.56E-11	0.81852914
1280	PBX2	5089	0.000597673	0.51693988
1281	PCNT	5116	1.54E-06	0.665118902
1282	CHMP1A	5119	0.005284327	-0.434112237
1283	PCSK1	5122	2.58E-06	0.654984887
1284	PCYT1A	5130	0.004512446	-0.440838274
1285	PDC	5132	7.00E-05	0.579827198
1286	PDCD1	5133	1.41E-06	0.666708298
1287	PDCD2	5134	0.008133438	-0.414688093
1288	PDE1C	5137	2.59E-11	0.830949379
1289	PDE2A	5138	0.000720718	0.510499216
1290	PDE3B	5140	0.001275505	0.490347702
1291	PDE4B	5142	0.003635649	0.449937164
1292	PDE4D	5144	0.005681232	0.430947728
1293	PDE6A	5145	8.16E-11	0.812417826
1294	PDGFA	5154	0.000788629	-0.507444141
1295	PDGFB	5155	1.30E-11	0.846392141
1296	PDE6B	5158	0.007012029	-0.421629879
1297	PDHA1	5160	0.000249614	-0.544446572
1298	PDHA2	5161	0.008491571	0.412600485
1299	PDHB	5162	0.000905031	-0.502664577

1300	ENPP1	5167	5.64E-11	0.818187795
1301	ENPP3	5169	2.16E-05	0.609143324
1302	PDPK1	5170	0.001147584	-0.49413319
1303	SLC26A4	5172	7.12E-07	0.679360252
1304	PECAM1	5175	1.65E-07	0.704894336
1305	PEX7	5191	7.63E-07	0.67813092
1306	PF4	5196	0.0001307	0.562970781
1307	PFAS	5198	0.001007499	-0.498782077
1308	PFDN5	5204	0.00024451	-0.54509325
1309	ATP8B1	5205	0.001055758	-0.497079816
1310	PFKFB1	5207	5.50E-09	0.756276344
1311	PFKM	5213	0.005418042	-0.432983165
1312	PFKP	5214	0.006278015	-0.42655638
1313	PFN1	5216	0.006875145	-0.422525067
1314	PGAM1	5223	0.004282714	-0.443043712
1315	PGC	5225	0.001355991	0.488187428
1316	PGM5	5239	7.28E-08	0.717998629
1317	ABCB1	5243	2.56E-07	0.697454741
1318	SLC25A3	5250	0.005377445	-0.433282686
1319	PHF1	5252	2.03E-08	0.737732904
1320	PHKB	5257	0.008168373	-0.414470729
1321	PHKG1	5260	1.09E-10	0.808559445
1322	PHKG2	5261	1.06E-09	0.778357911
1323	SERPINA4	5267	2.27E-08	0.73600323
1324	SERPINB8	5271	4.04E-11	0.823115606
1325	SERPINB10	5273	4.04E-07	0.689523616
1326	SERPINB13	5275	0.003716709	0.449010112
1327	PIGC	5279	0.008376565	-0.413285883
1328	PIGF	5281	0.000412252	-0.528978496
1329	PIK3C2A	5286	4.60E-05	-0.590611262
1330	PIK3C2G	5288	8.26E-11	0.812178476
1331	PIK3CB	5291	0.008915497	-0.410316308
1332	PIK3CG	5294	0.000756454	0.508922726
1333	PIN1	5300	1.74E-06	0.662667267
1334	PIN1L	5301	1.20E-09	0.77665765
1335	PIN4	5303	0.00027509	-0.541549682
1336	PITPNA	5306	0.000213064	-0.549162195
1337	PITX3	5309	2.91E-10	0.795302006
1338	PKLR	5313	1.33E-09	0.775240811
1339	PKHD1	5314	1.12E-10	0.808242153
1340	PKP1	5317	1.56E-10	0.803901389
1341	PLA2G1B	5319	0.00566321	0.431113305
1342	PLA2G5	5322	5.99E-11	0.817413284
1343	PLCB2	5330	1.84E-11	0.836616269
1344	PLCB3	5331	0.001039742	-0.497620008

1345	PLCD1	5333	1.52E-08	0.742032905
1346	PLCG2	5336	3.67E-07	0.691276063
1347	PLD1	5337	0.002724719	0.461587807
1348	PLG	5340	7.99E-05	0.576427997
1349	PLIN	5346	5.59E-09	0.756031849
1350	FXYD3	5349	0.000262843	0.542900245
1351	PLN	5350	2.77E-06	0.653663786
1352	PLRG1	5356	0.000725146	-0.510306712
1353	PLXNA1	5361	0.00291109	-0.458989164
1354	PLXNA2	5362	7.65E-10	0.782813974
1355	PMCH	5367	0.003822502	0.447841382
1356	Unknown	5370	1.21E-05	0.622526221
1357	PMM2	5373	0.006551107	-0.424708711
1358	PMP2	5375	2.34E-06	0.656956152
1359	PMS2L3	5387	0.00725627	-0.420054521
1360	EXOSC10	5394	0.001047258	-0.497360386
1361	PNLIP	5406	8.20E-07	0.676838547
1362	PNLIPRP1	5407	4.00E-10	0.791164267
1363	PNN	5411	5.82E-05	-0.584600689
1364	4-Sep	5414	8.99E-10	0.780720614
1365	PODXL	5420	0.002517216	-0.46478332
1366	POLB	5423	2.18E-07	0.700163433
1367	POLD1	5424	6.03E-05	0.583665935
1368	POLD2	5425	0.0022455	-0.469170557
1369	POLE2	5427	0.001409934	0.486648736
1370	POLR2A	5430	0.000536123	-0.520502876
1371	POLR2B	5431	3.71E-08	-0.72845218
1372	POLR2E	5434	3.43E-05	-0.597950414
1373	POLR2G	5436	0.008479476	-0.412677132
1374	POLR2H	5437	0.000528432	-0.520963471
1375	POLR2I	5438	0.006234247	-0.426851843
1376	POLR2J	5439	8.65E-05	-0.574340047
1377	POLR2L	5441	0.001388903	-0.487259851
1378	PON3	5446	9.04E-07	0.675003873
1379	POR	5447	0.001542069	-0.483355686
1380	POU2F2	5452	0.000860967	0.504387711
1381	POU3F1	5453	9.07E-11	0.811071764
1382	POU3F2	5454	1.49E-08	0.742334438
1383	POU3F3	5455	0.000160069	0.557346259
1384	POU4F2	5458	0.000101406	0.570064767
1385	POU6F1	5463	8.98E-06	0.629037616
1386	PPARA	5465	0.00186353	-0.476175834
1387	PPARG	5468	0.000636258	0.51476712
1388	MED1	5469	0.000505558	0.522431428
1389	PPAT	5471	0.003075233	0.45685537

1390	PPBP	5473	2.44E-08	0.734921351
1391	PPEF1	5475	4.46E-07	0.687789761
1392	CTSA	5476	0.001114876	-0.495152038
1393	PPIA	5478	0.000178323	-0.55428696
1394	PPIB	5479	0.00477502	-0.438446286
1395	PPID	5481	0.005098671	-0.435624445
1396	PPL	5493	0.00249499	-0.465136207
1397	PPM1G	5496	0.00237527	0.467134387
1398	PPP1CA	5499	0.001591642	-0.482164677
1399	PPP1R1A	5502	9.34E-08	0.714049539
1400	PPP1R2	5504	0.009275447	-0.408389435
1401	PPP1R3A	5506	4.10E-05	0.593451523
1402	PPP1R3C	5507	3.10E-09	0.764105749
1403	PPP1R7	5510	0.005474187	-0.432536834
1404	PPP1R8	5511	0.000534405	-0.520605803
1405	PPP2CB	5516	0.009404943	-0.407708323
1406	PPP2R2C	5522	4.07E-05	0.593678932
1407	PPP2R4	5524	0.007911408	-0.416027024
1408	PPP2R5A	5525	0.00928476	-0.408339197
1409	PPP2R5C	5527	0.003809865	-0.447996018
1410	PPP2R5E	5529	0.008392045	-0.41319154
1411	PPP3CA	5530	0.003483548	-0.45171079
1412	PPP4C	5531	0.005838565	-0.429744306
1413	PPP3CB	5532	0.000868853	-0.504073803
1414	PPP3R2	5535	7.31E-08	0.717940214
1415	PRB1	5542	5.35E-10	0.787377487
1416	PRB4	5545	5.01E-11	0.82048759
1417	PRCC	5546	2.91E-08	0.732157473
1418	PRCP	5547	0.002536278	-0.464502027
1419	PREP	5550	1.91E-08	0.738630904
1420	PRF1	5551	2.69E-10	0.796315134
1421	Unknown	5554	0.002151804	0.470779868
1422	PRKACA	5566	0.000468033	-0.524974871
1423	PRKAG1	5571	0.002092318	-0.471869388
1424	PRKAR1A	5573	0.00231427	-0.468051979
1425	PRKAR1B	5575	1.78E-10	0.801972572
1426	PRKAR2A	5576	7.61E-06	-0.632742813
1427	PRKCB1	5579	4.95E-09	0.757689312
1428	PRKCD	5580	0.002914455	-0.458944187
1429	PRKCI	5584	0.005023381	-0.436287741
1430	PKN2	5586	0.002679139	-0.462296171
1431	PRKCQ	5588	3.99E-06	0.646270174
1432	PRKCZ	5590	0.002731664	-0.4614796
1433	PRKG2	5593	1.05E-08	0.747230457
1434	MAPK3	5595	0.001409661	-0.486660436

1435	MAPK4	5596	4.97E-06	0.641705402
1436	MAPK7	5598	3.73E-09	0.761703679
1437	MAPK8	5599	8.79E-06	0.629561288
1438	MAPK9	5601	0.008685209	-0.411536653
1439	MAP2K2	5605	0.000128508	-0.563440272
1440	MAP2K3	5606	0.005290391	-0.434045403
1441	MAP2K5	5607	1.03E-08	0.747498668
1442	EIF2AK2	5610	0.003948608	-0.446449354
1443	DNAJC3	5611	0.004472884	-0.441177905
1444	PRL	5617	2.39E-06	0.656563295
1445	PRLR	5618	2.24E-05	0.608265876
1446	PRM1	5619	1.36E-09	0.774996033
1447	PROP1	5626	1.57E-09	0.773180728
1448	PRPH	5630	3.96E-11	0.823584659
1449	LGMN	5641	6.33E-06	0.636665432
1450	PRSS1	5644	0.000752557	0.509108843
1451	MASP1	5648	5.03E-09	0.757438065
1452	PRSS7	5651	3.49E-06	0.648961805
1453	PSAP	5660	0.008452682	-0.412822266
1454	PSG1	5669	1.61E-11	0.838159137
1455	Unknown	5670	2.07E-10	0.799883248
1456	PSG3	5671	2.15E-09	0.76886931
1457	Unknown	5672	1.16E-08	0.745790163
1458	Unknown	5676	2.66E-10	0.796437344
1459	PSG9	5678	8.36E-07	0.67645345
1460	PSG11	5680	5.20E-08	0.723294186
1461	PSKH1	5681	0.00028626	0.540343206
1462	PSMA1	5682	0.000386167	-0.531020216
1463	PSMA2	5683	0.005652671	-0.431187948
1464	PSMA3	5684	0.003525827	-0.451229074
1465	PSMA4	5685	0.001016508	-0.498449142
1466	PSMA5	5686	0.004941995	-0.436988775
1467	PSMA6	5687	0.001014007	-0.498558133
1468	PSMA7	5688	0.003809767	-0.448002262
1469	PSMB1	5689	0.000907952	-0.502521915
1470	PSMB3	5691	0.005039784	-0.436156364
1471	PSMB4	5692	3.60E-05	-0.596724095
1472	PSMB6	5694	0.008639992	-0.411783272
1473	PSMB7	5695	0.002826626	-0.460131297
1474	PSMC1	5700	0.002565219	-0.464043241
1475	PSMC5	5705	7.55E-07	-0.678299658
1476	PSMC6	5706	0.000315467	-0.537405288
1477	PSMD1	5707	6.14E-05	-0.583184279
1478	PSMD2	5708	0.005357332	-0.433458075
1479	PSMD4	5710	0.006594072	-0.424418581

1480	PSMD12	5718	0.003134312	-0.456058122
1481	PSMD13	5719	0.001595106	-0.482077651
1482	PSME1	5720	0.003541725	-0.451028388
1483	PSME2	5721	0.001373827	-0.487660517
1484	PTBP1	5725	5.36E-05	-0.58675034
1485	TAS2R38	5726	1.56E-09	0.773257502
1486	PTGDR	5729	3.09E-05	0.600439022
1487	PTGIR	5739	9.53E-07	0.67404846
1488	PTHLH	5744	2.25E-06	0.657744546
1489	PTK2	5747	0.000425452	-0.527973754
1490	PTK7	5754	2.48E-06	0.655762808
1491	TWF1	5756	0.004133398	-0.444572985
1492	PTMA	5757	0.00299105	-0.457908083
1493	PTMS	5763	0.003114091	-0.456319499
1494	QSOX1	5768	7.94E-10	0.782296822
1495	PTPN4	5775	0.005328196	-0.433706983
1496	PTPN6	5777	0.002645085	-0.462800295
1497	PTPN7	5778	0.004524694	0.440729605
1498	PTPN9	5780	0.004880771	0.437514954
1499	PTPN11	5781	0.003280353	0.454161928
1500	PTPN12	5782	0.000621292	-0.515606779
1501	PTPRA	5786	0.003432343	-0.452293235
1502	PTPRD	5789	0.000702303	0.51143074
1503	PTPRF	5792	0.000173928	-0.554984985
1504	PTPRN	5798	4.56E-09	0.758868237
1505	PTPRS	5802	0.008070581	-0.415084394
1506	PTS	5805	0.005295904	-0.433998732
1507	PURA	5813	0.008070299	-0.415099111
1508	PVR	5817	3.56E-11	0.825410133
1509	PVRL1	5818	6.28E-09	0.75443486
1510	PVRL2	5819	0.004877875	-0.437547193
1511	PWP2	5822	7.00E-05	-0.579809689
1512	ABCD3	5825	0.000321765	-0.536804294
1513	PXMP2	5827	0.009684165	0.406373367
1514	PXMP3	5828	0.000453191	0.525971143
1515	PEX5	5830	0.007378692	-0.419253815
1516	ALDH18A1	5832	0.00016293	-0.556829713
1517	PCYT2	5833	1.90E-05	-0.612152872
1518	PYGM	5837	2.01E-06	0.660003064
1519	QARS	5859	2.49E-08	-0.734521717
1520	RAB1A	5861	0.001723238	-0.479189018
1521	RAB2A	5862	0.002066628	-0.47233646
1522	RAB3B	5865	2.64E-11	0.830163617
1523	RAB3IL1	5866	0.004528116	0.440691661
1524	RAB4A	5867	0.0041349	-0.44455467

1525	RAB5A	5868	0.000714755	0.510806398
1526	RAB5B	5869	0.000179351	-0.554102837
1527	RAB6A	5870	0.000258327	-0.543405294
1528	RABGGTA	5875	0.007425447	-0.418963214
1529	RABGGTB	5876	0.001409314	-0.486673772
1530	RAB5C	5878	1.63E-07	0.705178946
1531	RAC1	5879	0.001612787	-0.481680957
1532	RAD21	5885	0.002143924	-0.470928496
1533	RAD23B	5887	0.008245248	-0.414045077
1534	RAD51	5888	1.26E-09	0.776004987
1535	RAD51L1	5890	3.14E-09	0.763922128
1536	RAD51L3	5892	1.83E-05	0.612999872
1537	RAF1	5894	4.07E-05	-0.593690423
1538	RAG1	5896	2.68E-08	0.733472588
1539	RALA	5898	0.001474834	-0.484987678
1540	RANBP2	5903	0.005475175	-0.432520828
1541	RAP1A	5906	0.000619643	-0.515689228
1542	RAP1B	5908	0.000314738	-0.537484578
1543	RAP1GAP	5909	0.002904388	-0.459084344
1544	RAP2B	5912	0.006102597	-0.427760626
1545	RARG	5916	7.52E-10	0.783030367
1546	RARS	5917	0.003262165	-0.454414276
1547	RASA1	5921	0.000990375	-0.499398747
1548	RASA2	5922	0.001875971	-0.475924026
1549	JARID1A	5927	0.006320249	-0.426261259
1550	RBBP4	5928	0.004045897	-0.445449138
1551	RBBP7	5931	2.91E-05	-0.601899732
1552	RBL1	5933	0.002446414	0.465932944
1553	RBL2	5934	6.51E-05	-0.581697676
1554	RBMS1	5937	0.002928586	-0.458740576
1555	RBMS2	5939	2.45E-08	0.734824017
1556	RBP2	5948	6.39E-05	0.582184558
1557	RCN1	5954	0.004340549	-0.442467717
1558	RCVRN	5957	3.02E-08	0.731584393
1559	PRPH2	5961	0.000216819	0.548628314
1560	RDX	5962	0.005740443	-0.430498093
1561	RECQL	5965	0.004291941	-0.44295767
1562	REL	5966	2.46E-08	0.734770749
1563	REGL	5969	1.30E-11	0.843810142
1564	RELA	5970	0.001392287	-0.487157051
1565	RENBP	5973	0.000765184	0.508537729
1566	UPF1	5976	0.004364949	-0.442212864
1567	DPF2	5977	0.002372247	-0.467182428
1568	REST	5978	7.42E-06	0.633268652
1569	RET	5979	2.48E-10	0.797403933

1570	REV3L	5980	0.007135591	-0.420876547
1571	RFC1	5981	0.000298158	-0.539108182
1572	RFNG	5986	9.89E-06	-0.626918224
1573	RFX1	5989	0.007411542	-0.419045998
1574	RFXAP	5994	0.002919984	-0.458863021
1575	RGR	5995	1.62E-08	0.741111847
1576	RGS13	6003	0.001135714	0.494498329
1577	RIT2	6014	0.000910628	0.502416385
1578	RLBP1	6017	5.79E-08	0.721646034
1579	RNASE2	6036	2.94E-05	0.601617517
1580	RNASE3	6037	3.01E-07	0.694646668
1581	BRD2	6046	0.007690252	-0.417305808
1582	RNF4	6047	0.001740197	-0.478795911
1583	RNF5	6048	1.54E-05	0.616956247
1584	RNF6	6049	0.000969744	-0.500115601
1585	RNH1	6050	4.02E-05	-0.593992423
1586	RNPEP	6051	0.003122869	-0.456206087
1587	ABCE1	6059	0.004007396	-0.445869895
1588	ROBO2	6092	0.000264808	0.54267646
1589	ROM1	6094	0.001357963	-0.488114738
1590	RORB	6096	0.001760271	0.478358716
1591	RPE	6120	0.004237526	-0.443538011
1592	RPL3	6122	0.000395435	-0.530301898
1593	RPL3L	6123	5.02E-12	0.863420446
1594	RPL4	6124	0.000427423	-0.527815574
1595	RPL5	6125	0.000915985	-0.502190452
1596	RPL6	6128	0.000285631	-0.540407918
1597	RPL7	6129	0.00110897	-0.495354641
1598	RPL7A	6130	0.002487794	-0.465271483
1599	RPL8	6132	0.000905822	-0.502615362
1600	RPL9	6133	0.000926622	-0.501767125
1601	RPL10	6134	1.72E-05	-0.614377305
1602	RPL11	6135	6.70E-06	-0.6354481
1603	RPL12	6136	0.000143754	-0.56038006
1604	RPL13	6137	0.000454457	-0.525875314
1605	RPL15	6138	0.000185781	-0.553090373
1606	RPL17	6139	0.000779683	-0.50784486
1607	RPL18	6141	6.53E-05	-0.581610915
1608	RPL19	6143	8.71E-06	-0.629778682
1609	RPL21	6144	2.44E-05	-0.606184423
1610	RPL22	6146	0.000124081	-0.564420173
1611	RPL23A	6147	2.45E-06	-0.656034811
1612	RPL24	6152	3.96E-05	-0.594327276
1613	RPL26	6154	0.000163436	-0.556732465
1614	RPL27	6155	0.001605749	-0.481837425

1615	RPL30	6156	0.000479862	-0.524125779
1616	RPL27A	6157	5.63E-05	-0.585521218
1617	RPL28	6158	0.000166835	-0.556180012
1618	RPL29	6159	8.54E-06	-0.630214322
1619	RPL31	6160	0.000160338	-0.557291404
1620	RPL32	6161	8.16E-07	-0.676948412
1621	RPL34	6164	3.80E-05	-0.595385546
1622	RPL35A	6165	0.000407214	-0.529348238
1623	RPL36AL	6166	0.001748061	-0.478628318
1624	RPL37	6167	0.000269385	-0.542161121
1625	RPL37A	6168	0.000116652	-0.566192105
1626	RPL38	6169	2.20E-05	-0.608726088
1627	RPL39	6170	0.000126478	-0.563890853
1628	RPL41	6171	0.000254336	-0.543871504
1629	RPL36A	6173	0.000605725	-0.51649617
1630	RPLP0	6175	0.001842829	-0.476615862
1631	RPLP1	6176	9.14E-05	-0.572832119
1632	RPLP2	6181	0.000101313	-0.570096167
1633	RPN2	6185	0.00143689	-0.485984579
1634	RPS2	6187	0.000222652	-0.547857455
1635	RPS3	6188	4.31E-05	-0.592222744
1636	RPS3A	6189	0.000711138	-0.510983881
1637	RPS4X	6191	8.02E-05	-0.576296571
1638	RPS4Y1	6192	0.000255713	0.543704433
1639	RPS5	6193	0.000496898	-0.522989949
1640	RPS6	6194	5.86E-05	-0.584369425
1641	RPS6KB1	6198	6.76E-05	-0.580716632
1642	RPS6KB2	6199	0.001241043	-0.491357084
1643	RPS7	6201	0.000105198	-0.569065459
1644	RPS8	6202	2.72E-05	-0.603521413
1645	RPS9	6203	0.000689178	-0.512080204
1646	RPS10	6204	4.33E-06	-0.644503698
1647	RPS11	6205	0.000317821	-0.537173281
1648	RPS12	6206	8.99E-06	-0.6290223
1649	RPS13	6207	1.98E-05	-0.611101505
1650	RPS14	6208	0.000254165	-0.543901506
1651	RPS15	6209	3.52E-05	-0.59728554
1652	RPS15A	6210	0.000187657	-0.552801804
1653	RPS16	6217	2.74E-06	-0.653878005
1654	RPS17	6218	0.001292534	-0.489900642
1655	RPS18	6222	6.30E-06	-0.636779741
1656	RPS19	6223	6.08E-05	-0.583481686
1657	RPS20	6224	0.000197378	-0.551336641
1658	RPS21	6227	0.000193349	-0.551941829
1659	RPS23	6228	0.000313489	-0.537603782

1660	RPS24	6229	1.33E-05	-0.620319833
1661	RPS25	6230	2.64E-05	-0.604294307
1662	RPS26	6231	0.006098651	-0.427801658
1663	RPS27	6232	0.002871319	-0.459541154
1664	RPS27A	6233	0.000174093	-0.554954664
1665	RPS28	6234	0.000270115	-0.542083132
1666	RPS29	6235	0.000163116	-0.556788292
1667	RRAS	6237	0.002871382	-0.459534997
1668	RRBP1	6238	0.004712648	-0.439004259
1669	RRM1	6240	0.005726679	-0.430598168
1670	RTKN	6242	3.23E-06	-0.650522856
1671	RS1	6247	0.001524035	0.483779788
1672	RYK	6259	0.002044484	-0.472729075
1673	S100A3	6274	7.18E-05	0.579175026
1674	S100A5	6276	0.000215244	0.548829966
1675	S100A7	6278	0.001301306	0.489639
1676	S100A13	6284	0.000593238	-0.517194517
1677	S100B	6285	0.007683339	0.41735622
1678	VPS52	6293	0.00666036	-0.423954657
1679	SAG	6295	2.01E-05	0.610762797
1680	MAPK12	6300	2.82E-08	0.732638706
1681	SAT1	6303	0.003278492	-0.454197776
1682	SBF1	6305	0.003380175	-0.45293002
1683	SC4MOL	6307	8.62E-05	-0.57441695
1684	ATXN8OS	6315	5.33E-06	0.64026575
1685	SERPINB3	6317	9.81E-07	0.673487639
1686	SERPINB4	6318	1.60E-10	0.803630052
1687	SCN1B	6324	5.48E-10	0.78700245
1688	SCN2A	6326	1.45E-08	0.742773863
1689	SCN3A	6328	9.80E-10	0.77939112
1690	SCN4A	6329	0.000774697	0.508063177
1691	SCN5A	6331	0.005952465	0.428858443
1692	SCN7A	6332	0.000908774	0.50248329
1693	SCN10A	6336	3.18E-08	0.730840831
1694	SCNN1A	6337	0.000926434	-0.501778952
1695	CCL5	6352	3.17E-06	0.650871613
1696	CCL13	6357	3.77E-11	0.824253924
1697	CCL16	6360	3.90E-06	0.646724151
1698	CCL19	6363	6.01E-06	0.637729852
1699	CCL22	6367	1.20E-05	0.622710308
1700	CCL23	6368	2.74E-09	0.765683006
1701	CCL25	6370	2.11E-07	0.700762658
1702	XCL1	6375	2.36E-05	0.606931734
1703	SDF2	6388	0.005198458	-0.434811195
1704	SDHA	6389	0.000178944	-0.55417944

1705	SDHC	6391	0.003923444	-0.446698008
1706	SEC13	6396	0.002472217	-0.465526179
1707	SEC14L1	6397	0.000100877	0.570210502
1708	TRAPPC2	6399	0.000348295	-0.534296155
1709	SELP	6403	1.15E-10	0.8079243
1710	SELPLG	6404	2.94E-06	0.652442568
1711	SEMG1	6406	4.43E-05	0.591583366
1712	SEMG2	6407	0.005840543	0.429715941
1713	SEPW1	6415	0.001135714	-0.494498682
1714	MAP2K4	6416	0.001639095	-0.481079833
1715	SET	6418	0.002108035	-0.47158325
1716	SFRS1	6426	0.000425027	-0.528007492
1717	SFRS2	6427	0.000250018	-0.544390314
1718	SFRS3	6428	6.38E-05	-0.582250213
1719	SFRS5	6430	0.004982839	-0.436615238
1720	SFRS6	6431	0.001059629	-0.496956792
1721	SFRS7	6432	4.52E-05	-0.591107316
1722	SFRS10	6434	0.001701353	-0.479689061
1723	SFTPC	6440	1.25E-07	0.709405623
1724	SGCA	6442	6.72E-06	0.635367854
1725	SGCD	6444	6.85E-06	0.634965921
1726	SGSH	6448	0.000112297	0.567219188
1727	SGTA	6449	0.004249756	-0.443413322
1728	SH3BGR	6450	3.73E-11	0.824767318
1729	ITSN1	6453	3.14E-08	0.731018198
1730	SH3GL1	6455	5.86E-09	0.755327981
1731	SH3GL3	6457	1.70E-10	0.802562408
1732	SHB	6461	2.12E-05	0.609581211
1733	SHBG	6462	1.31E-08	0.744177921
1734	SHC1	6464	0.000875944	-0.503774842
1735	FBXW4	6468	0.00356587	-0.450752388
1736	SI	6476	0.00016242	0.556921497
1737	SIAH1	6477	0.006927804	-0.422191561
1738	ST6GAL1	6480	0.008382311	-0.413248655
1739	ST3GAL3	6487	4.85E-08	0.724395148
1740	SIM1	6492	0.000327547	0.536287036
1741	SIM2	6493	2.06E-11	0.834879385
1742	SIPA1	6494	3.16E-10	0.794327005
1743	SKP1	6500	0.000235213	-0.54623309
1744	SKP2	6502	0.00315518	-0.455780153
1745	SLA	6503	0.007678358	0.417386568
1746	SLAMF1	6504	5.26E-11	0.819620155
1747	SLC1A2	6506	0.000111245	0.567513032
1748	SLC1A4	6509	3.32E-06	0.649957546
1749	SLC1A7	6512	1.23E-06	0.669348281

1750	SLC2A2	6514	3.77E-06	0.647445272
1751	SLC3A2	6520	0.008003905	-0.41549093
1752	SLC4A1	6521	1.86E-06	0.661492372
1753	SLC4A2	6522	8.70E-05	-0.574149538
1754	SMTN	6525	0.006210296	0.427012739
1755	Unknown	6526	0.000748581	-0.509282013
1756	SLC6A1	6529	1.56E-08	0.741639437
1757	SLC6A4	6532	0.005060949	0.435965665
1758	SLC6A7	6534	3.41E-10	0.793268948
1759	SLC6A12	6539	9.64E-06	0.627475547
1760	SLC7A1	6541	0.002198617	-0.469986938
1761	SLC8A2	6543	0.001324516	0.489030492
1762	SLC7A4	6545	1.18E-10	0.807542761
1763	SLC8A3	6547	0.000422195	0.528222839
1764	SLC9A1	6548	1.59E-09	0.772938869
1765	SLC10A1	6554	1.46E-05	0.618129878
1766	SLC10A2	6555	1.09E-06	0.671599697
1767	SLC12A3	6559	1.63E-09	0.772614118
1768	SLC15A2	6565	1.01E-06	0.672958622
1769	SLC16A2	6567	0.002265273	0.468835453
1770	SLC17A1	6568	3.60E-06	0.648334164
1771	SLC34A1	6569	0.000617315	0.51585659
1772	SLC18A1	6570	0.000185481	0.55314424
1773	SLC19A1	6573	0.000350256	0.534102866
1774	SLC20A2	6575	0.004710348	0.439043135
1775	SLCO2A1	6578	4.14E-09	0.760207068
1776	SLCO1A2	6579	0.000328779	0.536167286
1777	SLC22A2	6582	0.00017219	0.555259271
1778	SLIT1	6585	2.08E-08	0.737398062
1779	SLPI	6590	0.005097061	-0.435641307
1780	SMARCA4	6597	0.001941846	-0.474636227
1781	SMARCC2	6601	1.88E-10	0.80125734
1782	SMARCD1	6602	8.16E-11	0.812387653
1783	SMARCD2	6603	0.001925983	-0.47493897
1784	SMARCD3	6604	0.002578854	-0.463803267
1785	SMPD1	6609	0.000444737	0.526555922
1786	SUMO2	6613	2.27E-05	-0.607897267
1787	SNAI1	6615	1.33E-09	0.775315241
1788	SNCG	6623	9.77E-10	0.779464794
1789	SNRP70	6625	8.65E-05	-0.574311439
1790	SNRPA	6626	0.002422839	-0.466334064
1791	SNRPA1	6627	0.000141949	-0.560720999
1792	SNRPD2	6633	0.000271919	-0.541888219
1793	SNRPD3	6634	0.000439477	-0.52695306
1794	SNRPE	6635	0.003128111	-0.456142749

1795	SNRPG	6637	0.000770368	-0.508272289
1796	SOD1	6647	3.52E-05	-0.597264345
1797	SOD3	6649	1.53E-06	0.665224526
1798	SON	6651	0.000268102	-0.54229986
1799	SORL1	6653	0.006504632	-0.425020746
1800	SOS2	6655	4.92E-08	0.724152804
1801	SOX4	6659	0.000654559	-0.513841048
1802	SOX5	6660	3.75E-11	0.824513484
1803	SOX9	6662	0.004033096	-0.445578144
1804	SOX10	6663	1.01E-10	0.80969197
1805	SP4	6671	6.74E-07	0.68042129
1806	SPAM1	6677	2.29E-05	0.607649408
1807	SPAST	6683	0.003332926	-0.453501254
1808	SPG7	6687	0.004206914	-0.443861829
1809	SPIB	6689	3.97E-11	0.823472225
1810	SPINT1	6692	0.006445275	-0.425440878
1811	SPN	6693	6.75E-07	0.680390358
1812	SPRR1A	6698	9.80E-10	0.779392749
1813	SPRR2B	6701	8.69E-10	0.781212844
1814	SPRR2G	6706	0.000502239	0.522658904
1815	SPTB	6710	8.03E-11	0.812795802
1816	SRC	6714	6.55E-11	0.815510461
1817	AKR1D1	6718	0.002506022	0.464949656
1818	SRP9	6726	0.007862702	-0.416324455
1819	SRP14	6727	0.001723637	-0.479170501
1820	SRP19	6728	0.002926965	-0.458764957
1821	SRP54	6729	0.000801414	-0.50691639
1822	SRP68	6730	1.22E-06	-0.669417495
1823	SRP72	6731	0.000447142	-0.526377234
1824	SRPK2	6733	0.001733863	-0.478946666
1825	SRY	6736	3.29E-11	0.826416429
1826	TROVE2	6738	0.006724696	-0.423531139
1827	SSB	6741	0.003980496	-0.446131132
1828	SSR2	6746	7.89E-06	-0.631931915
1829	SSR3	6747	0.001073408	-0.496471468
1830	SSR4	6748	0.000932347	-0.501530274
1831	SSRP1	6749	0.002882395	-0.459382398
1832	SSX1	6756	1.08E-09	0.778152623
1833	SSX5	6758	1.30E-11	0.843899945
1834	ST14	6768	0.001053941	-0.497149399
1835	STAC	6769	1.61E-11	0.83795843
1836	STAT2	6773	0.000645838	-0.514305704
1837	STAT3	6774	0.000123112	0.56463243
1838	STAT4	6775	2.61E-08	0.733850632
1839	STAT6	6778	0.000210867	-0.54944959

1840	STAU1	6780	2.98E-05	-0.601303834
1841	SULT1E1	6783	0.006972112	0.421890243
1842	ELOVL4	6785	0.004583956	0.44016835
1843	NEK4	6787	0.009047722	-0.409575627
1844	CDKL5	6792	0.000250299	0.544348507
1845	AURKC	6795	8.59E-10	0.781346955
1846	STX1A	6804	2.20E-08	0.736491412
1847	STX3	6809	1.90E-09	0.770500047
1848	STXBP3	6814	0.001657088	-0.480674404
1849	SULT2A1	6822	3.20E-05	0.599653321
1850	SUPT5H	6829	0.008382635	0.413242003
1851	SUPT6H	6830	1.10E-06	0.671432029
1852	ABCC8	6833	7.87E-10	0.782420564
1853	SURF1	6834	0.00122358	-0.491876476
1854	SURF6	6838	4.57E-05	-0.59075191
1855	SYCP1	6847	1.18E-08	0.745616368
1856	T	6862	5.85E-05	0.584441477
1857	TACR2	6865	0.005944332	0.428917358
1858	TAC3	6866	3.23E-08	0.730553749
1859	ADAM17	6868	0.004358043	-0.442285786
1860	TACR3	6870	2.06E-05	0.610237834
1861	TAF2	6873	0.003113391	0.45633265
1862	TAF4	6874	0.000418883	-0.528452929
1863	TAF7	6879	0.000290931	-0.539843857
1864	TAF11	6882	0.000878704	-0.503669297
1865	MAP3K7	6885	0.00528529	-0.434099293
1866	TAL2	6887	1.49E-11	0.84030786
1867	TALDO1	6888	0.000969425	-0.50014738
1868	TAPBP	6892	0.000911255	-0.502389983
1869	TARBP1	6894	2.38E-06	0.656643758
1870	TARBP2	6895	0.008854876	0.41063796
1871	TAT	6898	1.89E-07	0.70259381
1872	TAZ	6901	2.03E-08	0.73779581
1873	TBCA	6902	0.004948917	-0.436907603
1874	TBCE	6905	0.009386992	-0.407819376
1875	SERPINA7	6906	2.25E-05	0.608092164
1876	TBL1X	6907	0.002689724	-0.462102716
1877	TBP	6908	1.06E-07	0.712077044
1878	TBX5	6910	0.008100221	0.414910012
1879	TCEA2	6919	1.09E-05	-0.624799311
1880	TCEA3	6920	0.004086835	-0.445025875
1881	TCEB1	6921	0.007817057	-0.416582145
1882	TCEB2	6923	0.004430185	-0.441577288
1883	TCEB3	6924	7.42E-07	0.678632176
1884	TBX3	6926	9.16E-09	0.749130913

1885	HNF1B	6928	9.47E-10	0.77996084
1886	TCF3	6929	0.001371867	-0.487722179
1887	TCF7	6932	0.002077367	-0.472132985
1888	TCF7L2	6934	0.000776717	-0.507968946
1889	C2orf3	6936	0.000438373	0.527029026
1890	TCF12	6938	0.000478491	-0.524229964
1891	TCF19	6941	1.12E-05	0.624191131
1892	VPS72	6944	0.008901712	-0.410398141
1893	MLX	6945	3.81E-09	0.761380755
1894	TCN1	6947	0.002064725	0.472372256
1895	TCN2	6948	0.00531861	0.433799794
1896	TCOF1	6949	7.41E-09	0.752142405
1897	TCP1	6950	0.005023381	-0.436286515
1898	TECTB	6975	0.009467115	0.407414235
1899	PPP1R11	6992	0.003434473	-0.452266187
1900	DYNLT1	6993	0.002773361	-0.460855784
1901	TDO2	6999	0.000629431	0.515178533
1902	PRDX2	7001	0.001231002	-0.491648648
1903	TEAD1	7003	0.002431204	-0.466191232
1904	TEAD3	7005	0.004030277	-0.445613723
1905	TEGT	7009	0.003195208	-0.455246746
1906	TEK	7010	2.71E-08	0.733256424
1907	TERF1	7013	0.001889992	-0.475629024
1908	TERT	7015	5.84E-07	0.683046214
1909	TFAP4	7023	2.14E-10	0.799492368
1910	TFCP2	7024	1.18E-07	0.71035711
1911	TFDP1	7027	0.00444744	-0.44142633
1912	TFDP2	7029	0.000781159	-0.507760055
1913	TFE3	7030	3.21E-05	-0.599528719
1914	TGFB3	7043	1.24E-06	0.669193007
1915	TGM4	7047	1.37E-08	0.74348685
1916	TGIF1	7050	0.006909641	-0.422307627
1917	TGM3	7053	3.93E-05	0.594545615
1918	TH	7054	0.005624668	0.431409585
1919	THBS3	7059	6.43E-07	0.681307041
1920	THPO	7066	4.76E-07	0.686615074
1921	THRSP	7069	5.89E-10	0.786076987
1922	TIMP1	7076	0.002529453	-0.464609134
1923	TIMP4	7079	0.000349751	0.534162439
1924	TKT	7086	0.003565527	-0.450761121
1925	TLE1	7088	0.001008891	-0.498723089
1926	TLE3	7090	0.00489164	-0.437417829
1927	TLL1	7092	2.26E-05	0.608026086
1928	TLL2	7093	5.21E-10	0.787670352
1929	TLOC1	7095	0.002916344	-0.458911174

1930	TLR1	7096	2.58E-06	0.654994892
1931	TLR2	7097	1.22E-09	0.776377136
1932	TLR4	7099	4.11E-06	0.645616083
1933	NR2E1	7101	2.21E-07	0.699956195
1934	TM4SF4	7104	0.007256985	0.420045543
1935	TMEM1	7109	0.007634485	-0.417655467
1936	TMF1	7110	0.004297702	-0.442898845
1937	TMPRSS2	7113	6.94E-07	0.679847043
1938	CLEC3B	7123	0.001152118	0.493983512
1939	TNF	7124	2.03E-10	0.80022734
1940	TNNC2	7125	0.000158088	0.557698203
1941	TNP2	7142	0.000368384	0.532474831
1942	TOP1	7150	0.000617904	-0.515799621
1943	TOP2B	7155	4.26E-06	-0.644865414
1944	TP53BP1	7158	4.04E-05	-0.59383399
1945	TP53BP2	7159	0.007337434	-0.419506497
1946	TP73	7161	2.78E-05	0.602952733
1947	TPD52	7163	0.009634006	-0.406622475
1948	TPD52L2	7165	0.000701853	-0.511464664
1949	TPM3	7170	0.000311118	-0.537820118
1950	TPO	7173	0.002606464	0.46339576
1951	TPP2	7174	0.003373178	-0.45302377
1952	TPT1	7178	5.61E-05	-0.585604306
1953	NR2C2	7182	1.39E-06	-0.666952833
1954	HSP90B1	7184	0.006221373	-0.426940288
1955	TRAF1	7185	5.60E-07	0.683802182
1956	TRAF3	7187	0.005791333	0.430116073
1957	TRAF6	7189	9.94E-06	0.62679706
1958	TRH	7200	0.001220635	0.491961624
1959	TRHR	7201	7.45E-05	0.578204251
1960	CCT3	7203	6.12E-06	-0.63733575
1961	TRIO	7204	7.66E-06	0.632581956
1962	TRIP6	7205	0.000865078	-0.504228285
1963	TRPC3	7222	0.000155794	0.558133792
1964	TRPC6	7225	2.26E-11	0.832978793
1965	TRPM2	7226	1.33E-06	0.667773885
1966	TSN	7247	0.000384573	-0.531145829
1967	TSG101	7251	0.000236794	-0.546037144
1968	TSPY1	7258	3.69E-10	0.792234285
1969	TSPYL1	7259	0.00029409	-0.539530791
1970	TST	7263	0.001472948	-0.485042674
1971	TTC3	7267	0.000392012	-0.530571258
1972	TTC4	7268	0.000520898	-0.521461934
1973	TTN	7273	0.006025419	0.428296672
1974	TTR	7276	2.38E-10	0.79800044

1975	TUBA4A	7277	0.009164687	-0.408965209
1976	TUFM	7284	0.000143561	-0.560419935
1977	TUFT1	7286	0.007129224	-0.420926158
1978	TULP2	7288	0.000199611	0.551017845
1979	HIRA	7290	0.004633622	-0.439745653
1980	TYR	7299	1.34E-10	0.805862085
1981	TYRO3	7301	1.14E-06	0.670727604
1982	TYRO3P	7302	1.25E-05	0.621667787
1983	U2AF1	7307	1.77E-06	-0.66239263
1984	UBA52	7311	5.28E-06	-0.640477996
1985	UBC	7316	0.000486682	-0.523693698
1986	UBA1	7317	2.68E-05	-0.60385622
1987	UBE2D2	7322	0.001830073	-0.476879869
1988	UBE2D3	7323	0.002695648	-0.46200094
1989	UBE2E1	7324	9.44E-06	-0.627954017
1990	UBE2E2	7325	0.002181504	-0.470288078
1991	UBE2G1	7326	0.008485897	-0.41264219
1992	UBE2G2	7327	0.004851576	-0.437797427
1993	UBE2H	7328	0.001870059	-0.476034588
1994	UBE2I	7329	0.006846967	-0.422718472
1995	UBE3A	7337	0.001355991	-0.488193782
1996	SUMO1	7341	0.006914967	-0.422267741
1997	UBP1	7342	9.42E-08	-0.713927502
1998	UBTF	7343	0.000121372	-0.565046394
1999	UCHL3	7347	0.004371863	-0.442148367
2000	UCP2	7351	0.005584892	-0.431710371
2001	UCP3	7352	0.000179034	0.554160516
2002	SLC35A2	7355	0.004029988	-0.445621592
2003	SCGB1A1	7356	0.000280209	0.540998155
2004	UGCG	7357	0.006705236	-0.423657214
2005	UMOD	7369	1.68E-06	0.663335769
2006	UCK2	7371	7.00E-05	0.579813612
2007	UMPS	7372	1.47E-08	0.742523347
2008	UQCRB	7381	0.002685526	-0.462186616
2009	UQCRC1	7384	0.000464435	-0.525220706
2010	UQCRC2	7385	2.51E-05	-0.605427382
2011	UROD	7389	0.000301263	-0.538787882
2012	UROS	7390	0.001719381	-0.479272264
2013	USF2	7392	1.95E-06	0.660538162
2014	USH2A	7399	5.81E-05	0.58466071
2015	CLRN1	7401	1.59E-11	0.838624587
2016	UTRN	7402	0.003694075	-0.449263673
2017	UTX	7403	0.000905822	-0.502618241
2018	VAV2	7410	1.85E-05	0.612688864
2019	VCL	7414	0.007400429	-0.419119499

2020	VDAC2	7417	0.002470923	-0.465560142
2021	VDAC3	7419	0.001740503	-0.478784707
2022	VEGFB	7423	0.001945488	-0.47456403
2023	VHL	7428	9.08E-08	0.71447902
2024	VIL1	7429	1.04E-07	0.712287908
2025	VIP	7432	2.48E-08	0.734610807
2026	VIPR2	7434	3.37E-06	0.649655303
2027	VRK2	7444	0.007595786	-0.417899067
2028	VSNL1	7447	0.000513386	0.521938119
2029	VWF	7450	2.64E-10	0.796637345
2030	WAS	7454	0.000107477	0.568486748
2031	EIF4H	7458	0.000183147	-0.553499281
2032	WHSC2	7469	0.003660364	-0.449634386
2033	WNT1	7471	0.001092387	0.495861867
2034	WNT6	7475	9.58E-10	0.779789861
2035	WNT10B	7480	5.59E-06	0.639242266
2036	WNT11	7481	0.00042435	0.52806685
2037	WNT2B	7482	0.000152682	0.558703464
2038	WT1	7490	3.68E-05	-0.596203781
2039	XBP1	7494	0.000773939	-0.508097853
2040	XDH	7498	6.11E-10	0.785570024
2041	XIST	7503	0.003295903	-0.453975996
2042	XPC	7508	0.000396618	-0.530207582
2043	XPNPEP1	7511	0.000233544	-0.546450303
2044	XPNPEP2	7512	6.08E-06	0.637489206
2045	XPO1	7514	1.73E-06	-0.662823292
2046	XRCC5	7520	0.001078767	-0.496290894
2047	YY1	7528	0.001328866	-0.488911067
2048	YWHAB	7529	0.000630393	-0.515121184
2049	YWHAE	7531	0.000331151	-0.535942446
2050	YWHAG	7532	0.001184916	-0.493010362
2051	ZAP70	7535	0.002898846	0.459155147
2052	SF1	7536	0.003060809	-0.457028233
2053	ZFP37	7539	2.38E-10	0.797907064
2054	ZFY	7544	1.67E-09	0.772350323
2055	ZNF8	7554	3.05E-05	0.600738098
2056	ZNF12	7559	5.75E-05	-0.584920929
2057	Unknown	7567	6.35E-11	0.815889584
2058	ZNF22	7570	0.003145924	-0.455908711
2059	ZNF24	7572	0.004684029	-0.439294451
2060	Unknown	7577	0.00048293	0.523928972
2061	ZSCAN20	7579	3.75E-10	0.792027415
2062	ZNF32	7580	0.00133335	-0.488794447
2063	ZNF33A	7581	0.000600248	-0.516792462
2064	ZNF35	7584	3.88E-10	0.791536903

2065	ZKSCAN1	7586	0.003976815	-0.446169401
2066	ZNF37A	7587	0.005995584	-0.42851469
2067	ZNF76	7629	0.000136193	-0.561872664
2068	ZNF80	7634	0.005115725	-0.435496414
2069	ZNF85	7639	0.008134473	-0.414677965
2070	ZNF90	7643	3.18E-07	0.693695941
2071	ZNF124	7678	0.002080213	0.472082423
2072	MKRN3	7681	0.000337691	0.535306299
2073	ZNF132	7691	7.11E-06	0.634147769
2074	ZNF143	7702	0.001775611	-0.478029324
2075	PCGF2	7703	0.005418041	-0.432988416
2076	ZNF146	7705	0.001001241	-0.499007841
2077	TRIM25	7706	1.81E-08	0.739473303
2078	ZBTB17	7709	0.003894053	-0.447029039
2079	ZNF154	7710	8.48E-09	0.750202504
2080	VEZF1	7716	0.001161471	-0.493694836
2081	ZNF174	7727	0.008539672	-0.412337998
2082	ZNF179	7732	9.66E-05	0.57134472
2083	ZNF187	7741	5.86E-09	0.755377111
2084	ZNF189	7743	1.32E-09	0.775392402
2085	ZNF193	7746	0.003739635	0.448760892
2086	ZMYM2	7750	0.000158881	-0.557559296
2087	ZNF202	7753	9.77E-10	0.779481927
2088	ZNF204	7754	0.002881758	0.459395371
2089	ZNF207	7756	0.000525614	-0.521147258
2090	ZFAND5	7763	9.72E-05	-0.571190156
2091	ZNF217	7764	0.000169735	-0.555674006
2092	ZNF230	7773	0.005869184	0.429475332
2093	SLC30A1	7779	0.001639738	-0.481056177
2094	ZP2	7783	5.90E-07	0.682868509
2095	LUZP1	7798	0.000896357	-0.503016518
2096	BSND	7809	3.37E-11	0.826052049
2097	CSDE1	7812	0.001190828	-0.492811422
2098	DAP3	7818	2.15E-05	-0.609208562
2099	NPHS2	7827	6.25E-08	0.720383652
2100	GCS1	7841	0.000445048	-0.526523037
2101	RNF103	7844	6.62E-06	-0.635708436
2102	PAX8	7849	0.005447524	-0.432734831
2103	BRPF1	7862	0.000750347	0.50920109
2104	IFRD2	7866	0.001368382	0.487818849
2105	MAPKAPK3	7867	9.10E-09	0.74923751
2106	SLMAP	7871	0.003129811	-0.456113183
2107	USP7	7874	0.001562755	-0.48283163
2108	ALDH5A1	7915	0.003038719	-0.457304737
2109	BAT2	7916	0.002549921	-0.464272198

2110	BAT3	7917	0.001355991	-0.48818276
2111	BAT5	7920	0.000295913	-0.539353301
2112	LST1	7940	8.17E-06	0.631170511
2113	TFEB	7942	3.03E-09	0.764448608
2114	EPM2A	7957	2.02E-07	0.701471201
2115	JTV1	7965	0.00243657	-0.466095473
2116	MAFK	7975	0.003387566	-0.452821611
2117	SHFM1	7979	0.002286533	-0.468470013
2118	ARHGEF5	7984	0.002385699	-0.466927715
2119	ZNF212	7988	0.001216889	-0.492062519
2120	MYST3	7994	0.002275929	-0.468652028
2121	GLRA3	8001	0.000995566	0.499206135
2122	BRD3	8019	0.001032795	-0.497886177
2123	LHX3	8022	2.78E-10	0.795899464
2124	MLLT10	8028	1.93E-07	0.702240931
2125	CUBN	8029	1.30E-11	0.84433756
2126	NCOA4	8031	0.002130094	-0.471191536
2127	SHOC2	8036	0.006739242	-0.423433136
2128	RASSF7	8045	0.000330565	-0.535996469
2129	PTP4A2	8073	7.50E-05	-0.578041968
2130	FGF23	8074	0.001375336	0.487609231
2131	MLF2	8079	0.002425403	-0.466293157
2132	AAAS	8086	1.25E-06	0.669074164
2133	FXR1	8087	0.002692635	-0.462049744
2134	CDK2AP1	8099	0.002594068	-0.46357862
2135	IFT88	8100	0.007660913	0.41750586
2136	PABPN1	8106	0.000268215	-0.5422826
2137	DPF3	8110	1.08E-10	0.808698434
2138	GPR68	8111	0.000284922	0.5404803
2139	TCL1A	8115	6.35E-11	0.815955158
2140	ANP32A	8125	0.002537609	-0.464457111
2141	C16orf35	8131	2.40E-05	0.606573767
2142	AKAP1	8165	0.000492534	-0.523303913
2143	SLC14A2	8170	7.12E-07	0.679372595
2144	MADCAM1	8174	0.000169007	0.555808075
2145	SF3A2	8175	1.90E-09	0.770522617
2146	ELL	8178	1.39E-07	0.707738078
2147	MKKS	8195	0.001244343	-0.491252084
2148	C21orf33	8209	4.51E-07	-0.687566686
2149	LZTR1	8216	4.28E-07	0.688528183
2150	CLTCL1	8218	2.82E-10	0.795659837
2151	DGCR14	8220	0.006277376	-0.426565442
2152	HDHD1A	8226	0.008862868	-0.410597095
2153	USP11	8237	0.008867658	-0.410570561
2154	RBM10	8241	9.60E-07	-0.673897113

2155	JARID1C	8242	2.49E-06	0.655723701
2156	SMC1A	8243	0.002233184	-0.469391483
2157	ARD1A	8260	0.004761291	-0.438582869
2158	JARID1D	8284	1.71E-05	0.614551017
2159	USP9Y	8287	0.000122654	0.564749561
2160	EPX	8288	3.44E-05	0.597889641
2161	ARID1A	8289	0.00186567	-0.47612201
2162	HIST3H3	8290	0.001127412	-0.494769076
2163	TRRAP	8295	0.000859537	-0.504443051
2164	PICALM	8301	0.000224893	-0.547559412
2165	AXIN1	8312	0.00261697	0.463238005
2166	BAP1	8314	0.001322844	-0.489081231
2167	FZD8	8325	2.55E-06	0.655223003
2168	FZD9	8326	0.003909273	0.446858531
2169	HIST1H2AK	8330	8.97E-05	0.573359506
2170	HIST1H2BL	8340	0.004186839	0.444049229
2171	HIST1H2BF	8343	6.87E-07	0.680079989
2172	HIST1H2BH	8345	3.15E-09	0.763864866
2173	HIST1H3J	8356	5.77E-07	0.683273855
2174	HIST1H4F	8361	0.000995056	0.499238756
2175	HIST1H4C	8364	0.002315096	-0.468030657
2176	HYAL3	8372	0.009093075	-0.409333602
2177	OR1A1	8383	5.61E-09	0.755977744
2178	OR1E1	8387	3.54E-10	0.792829501
2179	OR1G1	8390	0.002139593	0.47103364
2180	PIP5K1A	8394	0.000317821	0.537174381
2181	PIP4K2B	8396	0.000951114	-0.500792633
2182	PLA2G6	8398	0.000107137	0.568570128
2183	SPOP	8405	0.000690215	-0.512024716
2184	TAGLN2	8407	0.008317635	-0.413666038
2185	ULK1	8408	0.00898591	-0.409922288
2186	UXT	8409	0.000488587	-0.523577918
2187	EEA1	8411	0.008070299	-0.415097319
2188	STX7	8417	0.008498291	-0.412555919
2189	BBOX1	8424	5.27E-08	0.723074498
2190	SOAT2	8435	6.12E-11	0.816903703
2191	NCK2	8440	0.00018054	-0.553915454
2192	GNPAT	8443	8.31E-05	-0.575384832
2193	DYRK3	8444	4.46E-06	0.643937529
2194	DUSP11	8446	0.001442744	-0.485833874
2195	CUL4A	8451	0.000228757	-0.547059394
2196	CUL3	8452	4.57E-05	-0.590753193
2197	FOXN1	8456	0.003200444	0.455184631
2198	IRS4	8471	2.93E-08	0.732073601
2199	OGT	8473	0.00645716	-0.425355964

2200	RAE1	8480	0.002117424	-0.471410354
2201	SEMA7A	8482	3.64E-08	0.728720744
2202	PRSS12	8492	2.11E-07	0.700714725
2203	PPFIBP2	8495	0.000383505	-0.531240252
2204	CNTNAP1	8506	0.001727142	0.479088442
2205	NIPSNAP1	8508	0.000213339	0.549122569
2206	NDST2	8509	0.000775546	-0.508019236
2207	KCNAB2	8514	3.45E-07	0.692342346
2208	IKBKG	8517	0.00985974	0.405483253
2209	IKBKAP	8518	2.15E-05	-0.609216696
2210	Unknown	8519	0.000454171	-0.525898881
2211	GAS7	8522	1.07E-06	0.671906208
2212	CYP4F2	8529	1.62E-06	0.664070598
2213	CSDA	8531	0.007244173	-0.420165481
2214	BCAS1	8537	0.000720718	0.510514183
2215	API5	8539	1.93E-05	-0.611694721
2216	CGGBP1	8545	2.63E-05	-0.604305458
2217	AP3B1	8546	4.43E-11	0.821815067
2218	CDK10	8558	0.009887456	-0.405348231
2219	DENR	8562	0.001412434	-0.486581757
2220	THOC5	8563	0.000527151	-0.521047121
2221	Unknown	8564	1.78E-11	0.837093204
2222	YARS	8565	1.85E-08	0.739165949
2223	PDXK	8566	0.001151778	-0.49399825
2224	RRP1	8568	0.001639855	-0.481048354
2225	AKR7A2	8574	0.004307191	-0.44280892
2226	PRKRA	8575	0.000850259	-0.504835745
2227	STK16	8576	0.000404083	0.52959735
2228	OR6A2	8590	6.04E-07	0.682438174
2229	NOL14	8602	0.001190084	-0.492836382
2230	C4orf8	8603	0.00315537	-0.45577267
2231	PLA2G4C	8605	2.23E-06	0.657935603
2232	RDH16	8608	0.000222185	0.547930663
2233	USO1	8615	6.33E-08	0.720139583
2234	NPFF	8620	6.01E-11	0.817250226
2235	CDC2L5	8621	0.007602637	-0.417853778
2236	ASMTL	8623	0.009724438	-0.406166
2237	RFXANK	8625	0.000239721	-0.545698851
2238	TP63	8626	8.75E-07	0.675603557
2239	JRK	8629	0.00060957	0.516290698
2240	DNAH17	8632	0.000762477	0.508665382
2241	UNC5C	8633	2.21E-07	0.699917944
2242	RTCD1	8634	0.001334838	-0.488742685
2243	RNASET2	8635	0.005588918	-0.431678324
2244	DCHS1	8642	8.39E-06	0.63058321

2245	PTCH2	8643	0.000222584	0.547871634
2246	CHRD	8646	3.06E-09	0.764305507
2247	MAP2K1IP1	8649	0.003177163	-0.455464441
2248	PDE5A	8654	8.96E-10	0.780799374
2249	DYNLL1	8655	0.000871542	-0.503954176
2250	EIF3A	8661	0.001268904	-0.490520493
2251	EIF3B	8662	2.61E-05	-0.604528276
2252	EIF3D	8664	6.47E-06	-0.636175028
2253	EIF3F	8665	0.000249491	-0.544476667
2254	EIF3G	8666	0.007723724	-0.41711642
2255	EIF3H	8667	0.000667939	-0.513107597
2256	EIF3I	8668	0.001875971	-0.475920892
2257	SLC4A4	8671	0.006770391	0.423226004
2258	EIF4G3	8672	0.008122569	-0.414771314
2259	VAMP8	8673	0.003095618	-0.456589664
2260	VAMP4	8674	5.43E-10	0.787165123
2261	STX11	8676	0.002027912	0.473029308
2262	STX10	8677	0.000140461	0.561021862
2263	BECN1	8678	0.001878883	-0.475853937
2264	KRT38	8687	1.19E-09	0.776829241
2265	HYAL2	8692	0.000554004	-0.51943377
2266	DGAT1	8694	0.005840543	-0.429718558
2267	B4GALT3	8703	0.000328919	0.536137476
2268	B3GALT1	8708	6.36E-07	0.681505468
2269	SERPINB7	8710	7.65E-10	0.782821454
2270	MBTPS1	8720	0.001095298	-0.495763171
2271	EDF1	8721	0.000109993	-0.567858878
2272	EED	8726	0.000272946	-0.541782027
2273	RNMT	8731	0.0010635	-0.496817129
2274	GPAA1	8733	0.000247199	-0.544744155
2275	RIPK1	8737	5.88E-07	0.682911903
2276	CRADD	8738	3.26E-10	0.793856649
2277	ADAM21	8747	7.95E-06	0.631774932
2278	ADAM20	8748	1.25E-05	0.621742909
2279	ADAM7	8756	0.000811706	0.506445768
2280	PABPC4	8761	4.72E-06	-0.642778273
2281	CD164	8763	0.002367096	-0.46726053
2282	RAB11A	8766	0.004714806	-0.438977042
2283	SNAP23	8773	0.00064133	-0.514520026
2284	MTMR1	8776	0.003312068	-0.45378428
2285	SIGLEC5	8778	0.007138324	0.420845961
2286	RIOK3	8780	0.00426758	-0.44320996
2287	RGS11	8786	3.60E-11	0.825231881
2288	FBP2	8789	0.000227947	0.547170537
2289	TNFRSF11A	8792	1.09E-08	0.746640737

2290	SUCLG1	8802	1.21E-07	-0.709958826
2291	CREG1	8804	0.003087151	-0.45670036
2292	TRIM24	8805	0.000806275	-0.506719957
2293	IL18RAP	8807	0.001114815	0.495160778
2294	IL1RL2	8808	0.00064823	0.514181106
2295	IL18R1	8809	1.79E-10	0.801856178
2296	CDKL1	8814	0.000921655	0.501951628
2297	BANF1	8815	0.000192134	-0.552134746
2298	FGF18	8817	0.006013834	-0.428374196
2299	SAP30	8819	0.000251298	0.544225108
2300	HESX1	8820	6.98E-07	0.679731702
2301	FGF16	8823	4.25E-10	0.790321938
2302	LIN7A	8825	2.54E-10	0.797108698
2303	CD84	8832	9.93E-06	0.62681834
2304	SOCS2	8835	0.005410273	0.433046811
2305	WISP1	8840	2.73E-07	0.696299923
2306	ALKBH1	8846	2.82E-08	0.732675327
2307	TSC22D1	8848	0.002623167	-0.463130688
2308	CDK5R1	8851	3.28E-10	0.793784833
2309	DDEF2	8853	0.003171149	-0.45555157
2310	STK19	8859	0.001562755	-0.482831304
2311	PER3	8863	4.97E-09	0.757600181
2312	VNN2	8875	1.56E-10	0.803899932
2313	SQSTM1	8878	0.000555646	-0.519337642
2314	SGPL1	8879	0.004048158	-0.445407947
2315	APPBP1	8883	0.001738399	-0.478851356
2316	SLC5A6	8884	2.77E-08	0.732937834
2317	DDX18	8886	2.88E-06	0.652829487
2318	TAX1BP1	8887	0.000348295	-0.534288025
2319	MCM3AP	8888	0.007254494	-0.420082667
2320	BUD31	8896	0.005646809	-0.431242323
2321	MTMR3	8897	6.44E-09	0.754074743
2322	PRPF4B	8899	0.001408519	-0.486702726
2323	CPNE1	8904	0.001541546	-0.483372027
2324	HERC3	8916	0.000916944	0.502151458
2325	HERC2	8924	0.001089936	-0.495941757
2326	BSN	8927	5.95E-05	0.584008898
2327	FOXH1	8928	9.96E-11	0.809858553
2328	MBD4	8930	0.001544137	-0.483296343
2329	RAB7L1	8934	7.07E-07	0.679492303
2330	AP3D1	8943	8.88E-06	-0.629304185
2331	BTRC	8945	0.001719036	0.479284249
2332	HIST1H2AG	8969	0.003329054	0.45356322
2333	H1FX	8971	0.000345795	-0.534555917
2334	CHRNA6	8973	0.005362628	0.433405777

2335	P4HA2	8974	0.002686475	-0.462165419
2336	USP13	8975	0.001783072	0.477860603
2337	WASL	8976	0.003272556	-0.45427152
2338	STBD1	8987	0.007747676	0.416985742
2339	TRPA1	8989	1.58E-08	0.74141152
2340	SELENBP1	8991	0.001240768	-0.491369596
2341	ATP6V0E1	8992	0.001665792	-0.48048164
2342	LIMD1	8994	3.02E-08	0.731574442
2343	TNFSF18	8995	4.39E-05	0.591778827
2344	F2RL3	9002	2.27E-06	0.657573734
2345	TAF1C	9013	2.86E-06	0.652985673
2346	CH25H	9023	0.001540455	0.483405697
2347	BRSK2	9024	1.49E-06	0.665692735
2348	RNF8	9025	0.003387316	-0.452829552
2349	BAZ1B	9031	6.50E-07	0.681093686
2350	UBA3	9039	8.32E-08	-0.715926506
2351	UBE2M	9040	6.35E-11	0.815917084
2352	RPL14	9045	2.05E-06	-0.659633508
2353	DOK2	9046	8.35E-08	0.715852519
2354	ARTN	9048	9.96E-11	0.809841906
2355	MAP7	9053	0.002629153	-0.463031975
2356	NFS1	9054	0.002685526	-0.462182838
2357	SLC7A7	9056	1.35E-05	0.619854191
2358	PAPSS1	9061	5.47E-05	-0.586273993
2359	SYT7	9066	7.14E-08	0.718282095
2360	Unknown	9069	0.000602688	-0.516664177
2361	PRY	9081	0.000933247	0.50149018
2362	Unknown	9083	3.84E-06	0.647062442
2363	EIF1AY	9086	0.000402845	0.529693978
2364	DNAJA3	9093	0.001391498	-0.487191674
2365	UNC119	9094	0.000354826	-0.533700324
2366	TBX18	9096	6.30E-07	0.681659252
2367	USP8	9101	0.008373555	-0.413305149
2368	FCGR2C	9103	0.003944513	0.446491592
2369	MTMR7	9108	9.29E-09	0.748929391
2370	MTMR4	9110	0.000240185	-0.545617224
2371	MTA1	9112	0.001075848	-0.496385085
2372	LATS1	9113	2.68E-07	0.69659767
2373	INA	9118	0.000542807	0.520115434
2374	KRT75	9119	3.95E-10	0.791335129
2375	FAM50A	9130	0.001109313	-0.495328681
2376	AIFM1	9131	0.000631685	0.51502831
2377	RRP9	9136	0.003023156	-0.457493959
2378	CXorf1	9142	0.007611163	0.417799978
2379	SYNGR2	9144	0.008329695	-0.413603998

2380	HGS	9146	0.004415444	-0.441745181
2381	SDCCAG1	9147	0.003992807	-0.4460105
2382	CTDP1	9150	0.008552846	-0.412260721
2383	SLC28A1	9154	9.47E-06	0.627885658
2384	FIBP	9158	0.001375243	-0.487616959
2385	PCSK7	9159	0.002433727	0.466151002
2386	DGKI	9162	2.05E-05	0.610296371
2387	EBAG9	9166	0.002413022	-0.466491925
2388	TMSB10	9168	0.007880249	-0.416216961
2389	SFRS2IP	9169	4.55E-05	-0.590880233
2390	EDG4	9170	8.00E-05	0.576369385
2391	IL1RL1	9173	6.82E-09	0.7533161
2392	HTR3B	9177	0.002773019	0.460865392
2393	ARHGEF2	9181	1.44E-06	0.666372225
2394	BUB3	9184	0.001143909	-0.494243223
2395	REPS2	9185	9.46E-09	0.748690006
2396	SLC24A1	9187	0.000395887	-0.53026444
2397	DDX21	9188	0.000506319	-0.522379728
2398	DEDD	9191	0.0004857	-0.523762398
2399	SLC33A1	9197	0.005862766	-0.429537203
2400	ZMYM4	9202	0.000992572	-0.499325925
2401	LRRFIP1	9208	0.005322777	-0.433765594
2402	BMP15	9210	5.32E-05	0.586913332
2403	LGI1	9211	2.14E-06	0.658728691
2404	XPR1	9213	0.002988709	-0.457945794
2405	Unknown	9214	9.74E-10	0.779547544
2406	LARGE	9215	0.00024704	0.544779542
2407	VAPB	9217	8.55E-07	0.676043872
2408	VAPA	9218	0.003771974	-0.448408464
2409	DLGAP2	9228	2.04E-07	0.70135086
2410	DLGAP1	9229	0.001203099	0.492449235
2411	RAB11B	9230	0.005227184	0.434563001
2412	PTTG1	9232	4.42E-05	0.591597312
2413	PNMA1	9240	0.001860505	-0.476249087
2414	MSC	9242	1.19E-10	0.8074452
2415	GPR50	9248	2.10E-07	0.700834803
2416	RPS6KA5	9252	0.006978147	0.421846703
2417	NUMBL	9253	0.001909294	-0.47526491
2418	SCYE1	9255	0.001711688	-0.47946093
2419	PDLIM7	9260	0.007595786	0.417902715
2420	STK17B	9262	0.001236984	0.491476379
2421	PSCD1	9267	0.008213725	-0.414220988
2422	ITGB1BP1	9270	0.001827178	-0.476947768
2423	WDR46	9277	0.000186691	-0.552946485
2424	TAAR2	9287	8.66E-06	0.629897454

2425	TAAR3	9288	2.98E-07	0.694828441
2426	GPR56	9289	0.000155794	0.558129299
2427	GPR52	9293	2.43E-05	0.606252455
2428	SFRS11	9295	0.000333042	-0.535725292
2429	SOCS6	9306	0.003762093	0.448527908
2430	CD83	9308	1.93E-08	0.738478066
2431	ZNF235	9310	0.003939433	0.446542744
2432	KCNB2	9312	4.61E-08	0.725212938
2433	MMP20	9313	1.56E-05	0.616712738
2434	TRIP12	9320	0.001165318	-0.493575304
2435	HMGN3	9324	0.000810855	-0.506488435
2436	TRIP4	9325	0.001114815	-0.495159397
2437	GTF3C3	9330	4.91E-05	-0.588937833
2438	TGM5	9333	7.52E-06	0.632980326
2439	B4GALT5	9334	0.003954647	-0.446389656
2440	GLP2R	9340	1.93E-05	0.611708603
2441	SNAP29	9342	6.91E-06	0.634783626
2442	EFTUD2	9343	0.000781405	-0.50774495
2443	TAOK2	9344	5.38E-10	0.787284875
2444	RPL23	9349	0.000346137	-0.534511559
2445	SLC9A3R2	9351	0.009307209	-0.408217329
2446	TXNL1	9352	0.00047253	-0.524641223
2447	UBE4A	9354	9.00E-05	-0.573268561
2448	PPIG	9360	0.000827827	0.505735006
2449	LONP1	9361	0.00677331	-0.423204348
2450	RAB28	9364	0.00183834	-0.476711563
2451	COX5A	9377	0.009045521	-0.409600857
2452	NRXN1	9378	1.71E-09	0.771969783
2453	GRHPR	9380	3.23E-05	-0.599365319
2454	COG1	9382	1.18E-07	0.710354268
2455	SLC22A14	9389	1.18E-10	0.807586636
2456	CIAO1	9391	0.000393019	-0.53047655
2457	TGFBRAP1	9392	0.000492025	-0.523356898
2458	HS6ST1	9394	0.000742714	-0.509536387
2459	GRAP2	9402	1.25E-07	0.70949255
2460	LPXN	9404	4.71E-08	0.724862353
2461	ZRANB2	9406	0.001497504	-0.48442076
2462	ARHGAP29	9411	0.005418998	-0.432971382
2463	TJP2	9414	0.000914347	-0.502259587
2464	DDX23	9416	0.00068385	-0.512335415
2465	CRIPT	9419	0.000144149	0.560305179
2466	HAND1	9421	0.000152891	0.558652893
2467	ECEL1	9427	4.61E-05	0.590523148
2468	NCR2	9436	3.59E-08	0.728963593
2469	NCR1	9437	2.48E-06	0.655787867

2470	MED23	9439	0.001199904	-0.492548432
2471	MED17	9440	0.00909301	-0.409338991
2472	MED26	9441	0.001185759	-0.492982746
2473	ITM2B	9445	0.002471144	-0.465551674
2474	GSTO1	9446	0.006777034	-0.423178167
2475	AIM2	9447	0.009521349	0.407165201
2476	LY86	9450	4.71E-08	0.724886307
2477	EIF2AK3	9451	0.001897863	-0.475482636
2478	ITM2A	9452	3.01E-06	0.651924382
2479	FHL5	9457	7.08E-08	0.718409885
2480	ARHGEF6	9459	0.000705562	0.511283907
2481	HAND2	9464	1.04E-09	0.778656956
2482	CHST3	9469	0.000939743	0.501218004
2483	EIF4E2	9470	0.003705308	-0.449134578
2484	AKAP6	9472	1.18E-10	0.807586854
2485	MED20	9477	6.75E-08	0.719137992
2486	ONECUT2	9480	0.000180052	0.553990808
2487	PIGL	9487	2.10E-09	0.769208403
2488	PGS1	9489	0.00015719	-0.557860536
2489	KIF23	9493	1.24E-06	0.669243428
2490	MAGED1	9500	0.000157157	-0.557879848
2491	PAGE4	9506	7.13E-10	0.783688207
2492	PMPCB	9512	0.000359859	-0.533235047
2493	FXR2	9513	1.23E-07	0.70968605
2494	STXBP5L	9515	4.57E-09	0.758848925
2495	TBPL1	9519	0.005250483	-0.43437568
2496	GPSN2	9524	0.004916513	-0.437196878
2497	VPS4B	9525	0.001541353	-0.483381589
2498	MPDU1	9526	1.31E-08	0.744172798
2499	TMEM59	9528	0.004626823	-0.439804242
2500	BAG5	9529	0.001189804	-0.492849306
2501	POLR1C	9533	0.002574036	-0.463899012
2502	TP53I11	9537	2.22E-08	0.736326879
2503	EI24	9538	1.85E-05	-0.612752863
2504	NRG2	9542	1.84E-11	0.836541998
2505	APBA3	9546	0.002245619	-0.46916334
2506	ATP5J2	9551	0.000239935	-0.545656862
2507	SPAG7	9552	0.002758256	-0.461068809
2508	H2AFY	9555	0.000162676	-0.556875468
2509	C14orf2	9556	0.000140387	-0.561041328
2510	GTF2IRD1	9569	0.000110599	-0.567699402
2511	GOSR2	9570	3.80E-10	0.791815354
2512	GDF3	9573	2.18E-08	0.736629729
2513	CLOCK	9575	3.39E-08	0.729759351
2514	PREPL	9581	0.000564908	-0.518778881

2515	ENTPD4	9583	0.004030305	-0.44560816
2516	RBM39	9584	1.04E-05	-0.62586497
2517	PRDX6	9588	0.000812066	-0.506426816
2518	WTAP	9589	0.00071062	-0.511025358
2519	IER2	9592	0.001007182	-0.498797319
2520	SMAD5OS	9597	4.09E-10	0.790921323
2521	PDIA4	9601	0.005995584	-0.428514447
2522	NFE2L3	9603	0.006098651	0.427800622
2523	RNF14	9604	0.001901159	-0.475415092
2524	RAB36	9609	0.008432118	0.412943781
2525	NCOR2	9612	0.000502485	-0.522621263
2526	TRAF4	9618	0.009056391	-0.409525003
2527	KLK4	9622	5.42E-11	0.818905558
2528	AATK	9625	1.72E-07	0.704206311
2529	GUCA1C	9626	5.99E-11	0.817332038
2530	RGS6	9628	4.99E-06	0.641625507
2531	CLCA3	9629	4.87E-09	0.757931341
2532	GNA14	9630	0.000896167	0.503028617
2533	CLCA2	9635	0.00067143	0.512938644
2534	IKBKE	9641	1.13E-09	0.777441907
2535	MORF4L2	9643	2.83E-05	-0.602525258
2536	CTR9	9646	0.00777943	-0.416800867
2537	PPM1F	9647	1.40E-08	0.743262319
2538	GCC2	9648	0.00029386	-0.539557805
2539	RALGPS1	9649	5.13E-10	0.787859517
2540	MTFR1	9650	0.002605635	0.463411962
2541	TTLL4	9654	2.29E-09	0.768089016
2542	KIAA0430	9665	0.008501748	-0.412532793
2543	IPO13	9670	1.36E-09	0.775051968
2544	SLC25A44	9673	0.008948751	-0.410127689
2545	KIAA0406	9675	0.000858586	-0.504493363
2546	PHF14	9678	5.76E-05	-0.584887005
2547	JMJD2A	9682	0.006155476	0.427388752
2548	N4BP1	9683	1.02E-07	0.712626308
2549	Unknown	9684	3.51E-05	-0.597350747
2550	CLINT1	9685	0.003315611	0.453736319
2551	VGLL4	9686	4.03E-07	-0.689604036
2552	NUP93	9688	0.008400125	-0.413134378
2553	UBE3C	9690	0.004916513	-0.437195447
2554	RAPGEF2	9693	0.000469681	-0.524864485
2555	TTC35	9694	0.00174845	-0.478615622
2556	CROCC	9696	1.40E-10	0.805421553
2557	ESPL1	9700	0.008346152	0.413480828
2558	CEP57	9702	0.001588491	-0.482249219
2559	KIAA0100	9703	0.000214433	-0.54896511

2560	DHX34	9704	6.12E-11	0.816801652
2561	ST18	9705	3.89E-05	0.594823275
2562	ULK2	9706	0.003213917	-0.455006708
2563	HERPUD1	9709	0.00171773	-0.479319804
2564	FAM131B	9715	0.004165841	0.44426123
2565	AQR	9716	0.002110681	0.471526531
2566	ECE2	9718	0.00057833	0.518035705
2567	NOS1AP	9722	3.24E-06	0.650452718
2568	SART3	9733	0.001476484	-0.484941135
2569	USP34	9736	0.007804199	-0.416666861
2570	LAPTM4A	9741	0.003800574	-0.448098477
2571	IFT140	9742	5.69E-10	0.786543245
2572	FAM115A	9747	0.001461005	-0.485363836
2573	HDAC4	9759	0.002536488	0.464493784
2574	KIAA0152	9761	0.000300763	0.538838475
2575	KIAA0513	9764	2.99E-06	0.652068266
2576	KIAA0247	9766	0.00634394	-0.426093681
2577	PHF16	9767	0.004029772	-0.445628826
2578	RASSF2	9770	1.75E-08	0.740002073
2579	KIAA0195	9772	5.82E-05	0.584590633
2580	BCLAF1	9774	9.89E-05	-0.570743314
2581	EIF4A3	9775	0.002820489	-0.460216305
2582	KIAA0652	9776	0.002223031	0.469586493
2583	TM9SF4	9777	0.00097346	0.499977149
2584	TBC1D5	9779	0.003722139	-0.448947892
2585	FAM38A	9780	0.004469325	0.441217247
2586	MATR3	9782	0.000119643	-0.565433885
2587	RIMS3	9783	0.000296421	0.539295174
2588	SNX17	9784	1.39E-05	0.619329537
2589	KIAA0586	9786	1.75E-06	0.662559912
2590	MTSS1	9788	1.49E-06	0.665697808
2591	SPCS2	9789	7.54E-09	0.751922911
2592	BMS1	9790	0.003410249	-0.45257787
2593	MAML1	9794	0.00115829	-0.493796616
2594	KIAA0174	9798	0.000263888	-0.54278696
2595	DAZAP2	9802	0.000508323	-0.522253696
2596	TOMM20	9804	0.002385699	-0.466930027
2597	SCRN1	9805	0.00030058	-0.538860938
2598	IHPK1	9807	0.001887534	0.475682387
2599	RNF40	9810	1.17E-09	0.776953818
2600	SFI1	9814	0.009707453	-0.406246389
2601	KEAP1	9817	0.000274756	-0.541588129
2602	NUPL1	9818	0.000630509	-0.515105604
2603	SPATA2	9825	9.13E-06	0.62864396
2604	ARHGEF11	9826	1.33E-09	0.775260574

2605	RGP1	9827	0.007825316	-0.416535876
2606	ARHGEF17	9828	3.28E-08	0.730313888
2607	KIAA0125	9834	3.00E-08	0.731693706
2608	LCMT2	9836	0.002391579	-0.466828971
2609	ZEB2	9839	0.003869308	0.447303273
2610	KIAA0748	9840	0.002162371	0.470606939
2611	ELMO1	9844	9.07E-10	0.780611564
2612	GAB2	9846	0.004604767	-0.439993495
2613	KIAA0528	9847	0.003891932	-0.447053709
2614	MFAP3L	9848	0.003961046	0.446326799
2615	ZNF518	9849	0.00954876	-0.407024589
2616	KIAA0753	9851	0.001633219	0.481239246
2617	EPM2AIP1	9852	0.000127284	-0.563712376
2618	RUSC2	9853	0.000458992	0.525562129
2619	CEP350	9857	0.005862766	-0.429538424
2620	CEP170	9859	9.14E-09	0.749161193
2621	PSMD6	9861	5.72E-07	-0.683436865
2622	TRIM66	9866	0.000713987	0.510855338
2623	PJA2	9867	1.02E-08	0.747625719
2624	TOMM70A	9868	0.000112955	-0.567057369
2625	SETDB1	9869	0.007539911	-0.418226912
2626	KIAA0317	9870	0.004950862	-0.436877415
2627	TLK1	9874	0.000176782	-0.554526866
2628	ZC3H11A	9877	0.001263402	-0.490700739
2629	DDX46	9879	0.000989556	-0.499429519
2630	ZBTB39	9880	3.76E-07	0.690880838
2631	OSBPL2	9885	0.000592989	-0.517212464
2632	KIAA0329	9895	0.00026528	-0.54261091
2633	UBAP2L	9898	0.009255068	-0.408517974
2634	SV2B	9899	4.29E-07	0.688483368
2635	SV2A	9900	0.000165953	0.556326794
2636	MRC2	9902	0.000519931	-0.521532322
2637	DENND4B	9909	0.006320814	-0.426252659
2638	ARNT2	9915	0.004821294	-0.438047096
2639	SEC16A	9919	0.004111927	-0.44477521
2640	RNF10	9921	0.006386702	-0.425823806
2641	ZBTB40	9923	9.51E-06	0.627783572
2642	USP52	9924	0.008832052	-0.410786273
2643	ZBTB5	9925	0.001671368	-0.480332557
2644	HELZ	9931	0.000110789	-0.567638508
2645	KIAA0020	9933	0.002143908	-0.470934214
2646	XYLB	9942	0.000119643	0.565424873
2647	GFPT2	9945	0.005309207	0.43388619
2648	HS3ST3A1	9955	0.003817401	0.447904689
2649	HS3ST2	9956	2.58E-10	0.796872804

2650	USP3	9960	0.001148464	-0.494103937
2651	MVP	9961	0.005679178	-0.430966448
2652	THRAP3	9967	0.003963766	-0.44629705
2653	MED12	9968	0.000931646	-0.501558646
2654	MED13	9969	0.000199409	-0.551054415
2655	NR1I3	9970	2.54E-10	0.797087315
2656	NR1H4	9971	0.000589537	0.517415315
2657	CCS	9973	0.0080724	-0.415070332
2658	CLEC2B	9976	7.90E-07	0.677529816
2659	RBX1	9978	0.003631176	-0.449991198
2660	REC8	9985	0.009902979	-0.405257465
2661	RCE1	9986	0.000382943	-0.531292593
2662	HNRPDL	9987	3.93E-07	-0.690059778
2663	PPP4R1	9989	0.000593256	-0.517187755
2664	NAALAD2	10003	7.53E-06	0.632955143
2665	NAALADL1	10004	7.63E-05	0.577605586
2666	ACOT8	10005	0.000588499	0.517477653
2667	ABI1	10006	0.000795946	0.507136343
2668	HDAC6	10013	1.56E-09	0.773275559
2669	HDAC5	10014	2.60E-08	0.733921594
2670	PDCD6IP	10015	1.26E-05	-0.621558984
2671	PDCD6	10016	0.001521746	-0.483844245
2672	BCL2L11	10018	8.94E-08	0.714753661
2673	SH2B3	10019	8.51E-08	0.715537809
2674	HCN4	10021	4.58E-11	0.821461781
2675	FRAT1	10023	0.001877474	-0.475884584
2676	TROAP	10024	0.000401002	0.529844478
2677	MED16	10025	0.007120251	-0.42097974
2678	PARP2	10038	0.000359208	0.533299814
2679	TOM1L1	10040	0.006307323	-0.426349085
2680	HMG2L1	10042	0.001601847	-0.481926288
2681	CST8	10047	1.67E-09	0.772297058
2682	RANBP9	10048	0.000359949	-0.533222199
2683	DNAJB6	10049	0.006504632	-0.425025191
2684	SLC17A4	10050	4.68E-10	0.789036096
2685	GJC1	10052	4.03E-06	0.646029794
2686	FARSB	10056	0.003897896	0.446988686
2687	DNM1L	10059	0.000794404	-0.507205291
2688	SCAMP2	10066	0.000332667	0.535763992
2689	SCAMP3	10067	0.000930118	-0.501613748
2690	MUC12	10071	1.57E-11	0.839113011
2691	DPP3	10072	0.001981429	-0.473899362
2692	HUWE1	10075	0.000190625	-0.552374571
2693	TSSC4	10078	1.40E-08	0.743273519
2694	USH1C	10083	5.94E-08	0.721193437

2695	COL4A3BP	10087	0.007156028	-0.420729973
2696	KCNK7	10089	5.51E-05	0.586076807
2697	ACTR3	10096	0.005315026	-0.433829962
2698	TSPAN3	10099	0.000127663	-0.563622505
2699	TSFM	10102	0.001408473	-0.486709351
2700	CTDSP2	10106	2.81E-05	-0.602689632
2701	TRIM10	10107	0.002027265	0.473045272
2702	ARPC2	10109	1.46E-05	-0.618160031
2703	SGK2	10110	0.003505492	0.451469369
2704	PREB	10113	4.19E-05	-0.592931198
2705	HIPK3	10114	1.87E-05	-0.612444152
2706	ENAM	10117	1.45E-09	0.774216352
2707	ACTR1B	10120	3.09E-05	-0.600476237
2708	ARL4C	10123	0.009404943	-0.407708711
2709	ZNF263	10127	0.00064862	-0.514151938
2710	LRPPRC	10128	0.0013062	-0.489516185
2711	TRAP1	10131	1.20E-05	-0.622651191
2712	BCAP31	10134	0.001298015	-0.489734876
2713	Unknown	10137	0.000471327	-0.524730596
2714	YAF2	10138	3.56E-06	0.648525205
2715	ARFRP1	10139	0.000167523	-0.556044389
2716	C4orf6	10141	0.000146151	0.559941131
2717	AKAP9	10142	0.001606744	-0.481812068
2718	CLEC3A	10143	1.12E-10	0.808261406
2719	FAM13A1	10144	0.002676524	-0.462334718
2720	G3BP1	10146	3.56E-05	-0.59700187
2721	SFRS14	10147	0.002226066	-0.469535753
2722	CEBPZ	10153	0.000302517	-0.538645924
2723	TRIM28	10155	0.001765198	-0.478241516
2724	FARP1	10160	0.001891848	-0.475591549
2725	SERF2	10169	1.62E-05	-0.615841265
2726	DHRS9	10170	7.44E-07	0.678585858
2727	RCL1	10171	0.001510116	0.484104556
2728	Unknown	10181	0.000470461	-0.524812062
2729	TSHZ1	10194	0.005648571	-0.431225454
2730	PRMT3	10196	0.000254353	-0.543863581
2731	MPHOSPH10	10199	0.003364807	-0.453121163
2732	CALCRL	10203	5.42E-08	0.722642382
2733	NUTF2	10204	1.15E-09	0.777207493
2734	TRIM13	10206	0.003596073	-0.450375734
2735	EIF1	10209	0.000175009	-0.554808541
2736	SSX3	10214	0.000117771	0.565903862
2737	OLIG2	10215	1.61E-05	0.615983595
2738	CTDSPL	10217	0.000775524	-0.508025913
2739	ANGPTL7	10218	4.57E-05	0.590753509

2740	KLRG1	10219	0.001035286	0.497795806
2741	GDF11	10220	4.73E-09	0.758306167
2742	GPA33	10223	1.30E-11	0.844676936
2743	CD96	10225	0.009692888	0.4063211
2744	M6PRBP1	10226	0.000588742	-0.517459879
2745	TETRAN	10227	0.00721056	-0.420391238
2746	STX6	10228	0.002007218	-0.473387814
2747	RCAN2	10231	9.82E-08	0.713315984
2748	LRRC23	10233	0.000725146	0.510305511
2749	LRRC17	10234	0.005353879	0.433492416
2750	HNRNPR	10236	2.77E-05	-0.603128577
2751	GLYAT	10249	7.48E-11	0.813736265
2752	SRRM1	10250	0.00424918	-0.443423501
2753	SPRY1	10252	0.007333428	-0.419553467
2754	STAM2	10254	0.007102062	-0.421088377
2755	CNKSR1	10256	0.003140502	-0.455979141
2756	SF3B4	10262	0.003440129	-0.452192575
2757	CDK2AP2	10263	0.003600802	-0.45032417
2758	RAMP2	10266	2.91E-08	0.732159351
2759	FSTL3	10272	0.000624821	0.515414093
2760	OPRS1	10280	0.000227278	0.54725343
2761	DSCR4	10281	0.000969617	0.500128428
2762	SAP18	10284	0.000283442	-0.540631335
2763	SMNDC1	10285	0.005596154	-0.431624946
2764	LILRB2	10288	3.29E-08	0.73027724
2765	EIF1B	10289	1.67E-05	-0.615142378
2766	SF3A1	10291	0.000904184	-0.502711812
2767	MAEA	10296	0.000123136	-0.564621418
2768	PAK4	10298	7.41E-05	0.578366655
2769	6-Mar	10299	0.000578793	-0.518006923
2770	DSCR3	10311	0.000589851	0.517394149
2771	RTN3	10313	0.00080937	-0.506560117
2772	LANCL1	10314	0.00442797	-0.441605915
2773	NMUR1	10316	0.009270608	-0.408425551
2774	TNIP1	10318	0.008431046	-0.412953912
2775	SMYD5	10322	0.000140002	-0.561116401
2776	KBTBD10	10324	2.18E-08	0.736606568
2777	SIRPB1	10326	9.21E-11	0.810876103
2778	AKR1A1	10327	2.29E-05	-0.60766529
2779	COX4NB	10328	0.000613686	-0.516061713
2780	CNPY2	10330	0.00016934	-0.555751914
2781	B3GNT3	10331	3.11E-09	0.764061034
2782	TLR6	10333	1.43E-09	0.774364161
2783	MRVI1	10335	1.47E-05	0.618038086
2784	PCGF3	10336	0.001060275	-0.49692686

2785	CCL26	10344	0.000849035	0.504884425
2786	WARS2	10352	0.001403117	-0.486873444
2787	HMG20B	10362	0.009733256	-0.406124505
2788	HMG20A	10363	0.003330956	-0.453527214
2789	CBARA1	10367	0.001861272	-0.47622626
2790	CACNG2	10369	1.32E-05	0.620496651
2791	IRF9	10379	0.008639992	0.411775481
2792	TUBB3	10381	0.007111766	-0.421030739
2793	TUBB4	10382	2.95E-11	0.827892067
2794	TUBB2C	10383	0.008633609	-0.411828379
2795	CEPT1	10390	1.80E-07	0.703510349
2796	NOD1	10392	1.37E-06	0.667225307
2797	GNB2L1	10399	4.76E-05	-0.589703084
2798	PEMT	10400	0.008211553	-0.414236497
2799	IFITM3	10410	0.00015122	-0.558985557
2800	YAP1	10413	0.000509711	-0.522164831
2801	TESK2	10420	1.33E-06	0.667793451
2802	CD2BP2	10421	0.001952478	0.474431948
2803	CDIPT	10423	0.00012184	-0.564938893
2804	PGRMC2	10424	0.004712648	-0.43900679
2805	ARIH2	10425	2.65E-05	-0.604135192
2806	TUBGCP3	10426	0.008967197	-0.410015282
2807	SEC24B	10427	2.88E-06	-0.652865269
2808	CFDP1	10428	0.004379424	-0.442077019
2809	TMEM147	10430	0.002634484	-0.462952555
2810	RBM14	10432	0.001560551	0.482898514
2811	TIMM17A	10440	0.000837562	-0.505329751
2812	RP11-298P3.3	10443	0.004685389	-0.43927333
2813	ZER1	10444	0.000301891	0.538719857
2814	MCRS1	10445	1.22E-09	0.776382143
2815	MAP3K7IP1	10454	0.000753158	-0.509073735
2816	HAX1	10456	0.004851709	-0.437791148
2817	MAD2L2	10459	5.82E-05	0.584568547
2818	MERTK	10461	3.62E-10	0.792528592
2819	SLC30A9	10463	0.002199555	-0.469965862
2820	PIBF1	10464	0.000523211	-0.52129487
2821	PPIH	10465	0.000608904	0.516339819
2822	ZNHIT1	10467	0.005052174	-0.436050443
2823	TIMM44	10469	0.006401217	-0.425727413
2824	TADA3L	10474	0.000276039	-0.541452075
2825	UBE2E3	10477	0.001968442	-0.474142049
2826	SLC9A6	10479	0.002039077	-0.472821575
2827	EIF3M	10480	4.20E-05	-0.592913041
2828	HOXB13	10481	2.26E-11	0.832940436
2829	NXF1	10482	8.80E-06	-0.629524482

2830	SEC23B	10483	0.008070299	-0.415095964
2831	SEC23A	10484	0.002845179	-0.459891641
2832	C1orf61	10485	8.80E-07	0.675486746
2833	CAP1	10487	0.003318945	-0.453695965
2834	CREB3	10488	0.002923268	0.458819094
2835	VTI1B	10490	0.001094777	-0.495787761
2836	VAT1	10493	1.79E-07	0.703556271
2837	STK25	10494	4.75E-05	-0.589764465
2838	CARM1	10498	0.007565661	-0.418084109
2839	NCOA2	10499	0.003783874	-0.448291696
2840	SEMA6C	10500	1.55E-11	0.839901115
2841	SEMA6B	10501	6.46E-06	0.636218843
2842	SEMA4B	10509	1.96E-09	0.770144279
2843	APPBP2	10513	0.000196068	-0.551532903
2844	MYBBP1A	10514	0.008096045	-0.414936831
2845	FBXW10	10517	1.45E-10	0.804964964
2846	DDX17	10521	2.78E-05	-0.603007604
2847	DEAF1	10522	0.00218294	0.470255342
2848	HTATIP	10524	3.23E-05	-0.599423777
2849	HYOU1	10525	0.002907773	-0.459037208
2850	IPO8	10526	0.000599008	-0.516857725
2851	NOL5A	10528	0.002439404	-0.466040115
2852	PITRM1	10531	0.000296676	-0.539266012
2853	ANP32B	10541	0.000609155	-0.516316162
2854	HBXIP	10542	0.001092387	-0.495861651
2855	TM9SF1	10548	3.34E-05	-0.5985778
2856	PRDX4	10549	0.002140525	-0.470997336
2857	RPP38	10557	2.00E-05	0.610918792
2858	SPTLC1	10558	0.000408111	-0.529281418
2859	SLC35A1	10559	0.003339555	-0.45342667
2860	ARFGEF1	10565	3.60E-05	-0.596664322
2861	SLC34A2	10568	0.009742754	-0.406069206
2862	SLU7	10569	0.004217852	-0.44373153
2863	MRPL28	10573	5.78E-06	0.638599962
2864	CCT7	10574	0.003588211	-0.450474069
2865	CCT4	10575	0.006731735	-0.423480843
2866	CCT2	10576	0.002961127	-0.458329076
2867	SORBS1	10580	7.92E-05	0.576660776
2868	IFITM2	10581	0.001846158	-0.476540734
2869	COLEC10	10584	0.00018427	0.553336653
2870	POMT1	10585	0.001693309	-0.479863628
2871	MAB21L2	10586	2.59E-05	0.604710773
2872	PRPF8	10594	0.000164373	-0.556575373
2873	AHSA1	10598	0.004525366	-0.440718941
2874	USP16	10600	0.000627515	-0.515272367

2875	PAIP1	10605	0.004929218	-0.437095749
2876	PAICS	10606	0.008606936	-0.41197614
2877	MXD4	10608	0.000601698	-0.51671732
2878	ERLIN1	10613	0.001564121	-0.482792358
2879	RBCK1	10616	0.000243855	-0.545182283
2880	STAMBP	10617	0.002046834	-0.472680356
2881	TGOLN2	10618	8.52E-05	-0.57472917
2882	ARID3B	10620	1.66E-10	0.803105993
2883	POLR3F	10621	5.73E-05	0.585007849
2884	POLR3C	10623	0.002568375	-0.463985432
2885	IVNS1ABP	10625	0.001817721	-0.477140941
2886	TAF6L	10629	0.000656733	0.513709731
2887	ATP5L	10632	0.000173432	-0.555070243
2888	LEFTY1	10637	1.96E-09	0.770150736
2889	TUSC4	10641	0.001067631	-0.496676059
2890	CAMKK2	10645	0.002084795	-0.471999005
2891	MTX2	10651	0.00014214	-0.560681165
2892	PMVK	10654	0.008964716	-0.410041599
2893	CUGBP1	10658	5.84E-08	-0.721498383
2894	LBX1	10660	5.38E-05	0.586632923
2895	KLF1	10661	4.54E-05	0.590987013
2896	CTCF	10664	0.001510116	-0.484107072
2897	C6orf10	10665	6.08E-07	0.682308761
2898	FARS2	10667	1.90E-09	0.770503778
2899	CGRRF1	10668	0.004355821	-0.44230945
2900	GNA13	10672	0.00436261	-0.442237472
2901	CSPG5	10675	0.000166699	0.556206821
2902	Unknown	10682	0.000147165	-0.559742758
2903	RRH	10692	3.54E-06	0.648664735
2904	CCT8	10694	2.24E-06	-0.65780152
2905	CORIN	10699	0.004352703	0.442351125
2906	USP39	10713	0.002623167	-0.463135627
2907	POLD3	10714	1.38E-05	0.619368177
2908	TBR1	10716	0.000506319	0.522377095
2909	NRG3	10718	5.12E-05	0.587885879
2910	POLQ	10721	1.89E-10	0.801167678
2911	MGEA5	10724	0.001321206	-0.489125577
2912	NUDC	10726	0.000556554	-0.519279289
2913	PTGES3	10728	0.004328789	-0.442591861
2914	YME1L1	10730	0.000663972	-0.513303909
2915	TCFL5	10732	0.005860591	-0.429561547
2916	PLK4	10733	1.87E-05	0.612443676
2917	STAG2	10735	0.001360861	-0.488031051
2918	RBBP9	10741	2.91E-10	0.795308037
2919	RAI2	10742	0.008358249	-0.413392464

2920	RAI1	10743	0.000361623	-0.533071428
2921	PHTF1	10745	0.00171326	-0.479414771
2922	MAP3K2	10746	0.000719417	-0.510598733
2923	MASP2	10747	0.001887287	0.475692147
2924	JARID1B	10765	0.000953524	-0.500704867
2925	HBS1L	10767	4.32E-05	0.592192473
2926	AHCYL1	10768	3.72E-05	-0.595920975
2927	ZMYND11	10771	2.01E-05	-0.610806137
2928	FUSIP1	10772	0.000564908	-0.518777498
2929	ARPP-19	10776	0.001292936	-0.489880884
2930	ARPP-21	10777	2.54E-10	0.797050798
2931	ZNF271	10778	0.007706511	-0.417213439
2932	ZNF234	10780	0.000914347	0.502261403
2933	NEK6	10783	0.00207697	0.472150247
2934	WDR4	10785	0.000176563	-0.554570923
2935	SLC17A3	10786	0.000726064	0.510259535
2936	IQGAP2	10788	9.80E-05	0.570990087
2937	OR5I1	10798	1.69E-10	0.802749715
2938	SEC24A	10802	0.001036725	-0.497736472
2939	CCR9	10803	0.009224783	0.408658864
2940	STARD10	10809	0.003280181	-0.454169127
2941	NOXA1	10811	0.006631863	-0.424157671
2942	Unknown	10816	5.01E-08	0.723858892
2943	FRS2	10818	5.26E-08	0.723121149
2944	EPAG	10824	3.90E-10	0.791475201
2945	NEU3	10825	4.76E-10	0.788828807
2946	C5orf4	10826	1.64E-06	0.663912407
2947	C7orf16	10842	4.55E-10	0.789387866
2948	TUBGCP2	10844	0.006673873	-0.423867065
2949	PDE10A	10846	0.000121351	0.565063201
2950	PPP1R13L	10848	0.004038841	0.445516739
2951	CCL27	10850	2.03E-06	0.659750045
2952	SLC26A1	10861	0.007897257	-0.416112297
2953	TSPAN9	10867	0.007537134	-0.418261476
2954	USP19	10869	4.37E-08	-0.726002531
2955	FAM12A	10876	4.06E-07	0.689448622
2956	Unknown	10877	1.03E-05	0.625994486
2957	SMR3B	10879	6.99E-11	0.814634423
2958	NPFFR2	10886	6.67E-09	0.753607113
2959	RAB10	10890	0.001534772	-0.483539931
2960	MMP24	10893	0.004513574	0.440823949
2961	PPBPL2	10895	0.008849085	0.410683964
2962	OCLM	10896	1.16E-08	0.745881317
2963	YIF1A	10897	0.002828346	-0.460105127
2964	JTB	10899	2.98E-06	-0.652161259

2965	RUNDC3A	10900	4.11E-09	0.760301901
2966	BRD8	10902	7.38E-05	-0.578449525
2967	BLCAP	10904	4.54E-05	-0.590940164
2968	MAN1A2	10905	1.65E-05	0.615321404
2969	TRAFD1	10906	5.27E-08	0.723054669
2970	TXNL4A	10907	0.000806319	-0.506712559
2971	SUGT1	10910	0.000543893	-0.520040821
2972	UTS2	10911	2.33E-05	0.607281853
2973	PAPOLA	10914	0.003046787	-0.457191821
2974	MAGED2	10916	0.000631594	-0.515038329
2975	RNPS1	10921	0.000296358	-0.539307004
2976	FASTK	10922	1.87E-07	-0.702820362
2977	SPIN1	10927	4.64E-06	-0.643116017
2978	RALBP1	10928	0.000919252	-0.502053015
2979	SFRS2B	10929	0.000667613	-0.513127611
2980	APOBEC2	10930	2.10E-10	0.799723972
2981	PRDX3	10935	0.002431204	-0.466192353
2982	GPR75	10936	0.003586931	0.450502663
2983	EHD1	10938	0.003530972	-0.451167209
2984	AFG3L2	10939	0.000400732	-0.52986906
2985	POP1	10940	2.25E-05	0.60809977
2986	KDELR1	10945	0.000202895	-0.550563931
2987	SF3A3	10946	0.008918172	-0.410294183
2988	STARD3	10948	2.42E-07	0.698371034
2989	BTG3	10950	0.000648075	-0.514199789
2990	SEC61B	10952	7.46E-05	-0.578170597
2991	TMED2	10959	0.005614214	-0.431489324
2992	ERP29	10961	0.004580936	-0.440197525
2993	RAB40B	10966	0.002348104	-0.467540509
2994	EBNA1BP2	10969	0.003870747	-0.447284752
2995	TMED10	10972	0.000152964	-0.558634542
2996	UQCR	10975	0.000112474	-0.567174512
2997	RAB32	10981	1.27E-10	0.806513636
2998	MAPRE2	10982	1.07E-07	0.711938322
2999	CCNI	10983	0.000582009	-0.517833335
3000	COPS5	10987	2.48E-05	-0.605750925
3001	IMMT	10989	1.07E-05	-0.625101228
3002	LILRB5	10990	0.003212024	-0.455032517
3003	SF3B2	10992	6.48E-07	-0.681138937
3004	SDS	10993	3.96E-10	0.791304119
3005	ILVBL	10994	0.008168373	-0.414468813
3006	SPINK5	11005	2.25E-05	0.608085873
3007	IL24	11009	1.16E-05	0.623345373
3008	TMED1	11018	1.01E-05	0.626359347
3009	Unknown	11024	3.28E-06	0.650210365

3010	DSTN	11034	0.002715239	-0.461722502
3011	GTF2A1L	11036	9.89E-06	0.62691578
3012	SMA4	11039	0.008958102	0.41007601
3013	MID2	11043	0.000543103	0.520093963
3014	POLS	11044	9.21E-05	-0.572633351
3015	SLC35D2	11046	1.83E-05	-0.612958682
3016	CPSF6	11052	0.000658988	-0.513581066
3017	ZPBP	11055	0.007044629	0.421420815
3018	WWP2	11060	0.001862181	-0.47620488
3019	CEP110	11064	5.21E-08	0.723253937
3020	U1SNRNPBP	11066	0.000499161	-0.522856012
3021	CYB561D2	11068	0.003251997	-0.454538827
3022	TMEM115	11070	0.007176016	0.420607146
3023	TRIM31	11074	8.95E-09	0.749451481
3024	STMN2	11075	1.46E-09	0.774036512
3025	HSF2BP	11077	8.07E-06	0.631430247
3026	RER1	11079	0.001034115	-0.497835949
3027	DIDO1	11083	0.002503977	-0.464982579
3028	ADAM30	11085	1.22E-09	0.776444858
3029	WDR5	11091	1.40E-05	0.619165473
3030	ADAMTS13	11093	0.000281786	0.540824163
3031	HNRPUL1	11100	0.003040996	-0.457268146
3032	KRR1	11103	0.005143233	-0.435257061
3033	KATNA1	11104	0.004938017	-0.4370252
3034	FGFR1OP	11116	0.001899348	-0.475451657
3035	EMILIN1	11117	2.98E-07	0.694774397
3036	PTPRT	11122	3.27E-09	0.76340887
3037	CD160	11126	0.003518262	0.451320462
3038	POLR3A	11128	1.26E-05	0.621450749
3039	SFRS16	11129	4.15E-11	0.822780618
3040	CAPN10	11132	0.000844786	0.505039604
3041	SLC7A9	11136	1.08E-09	0.778171392
3042	PWP1	11137	0.003918035	-0.446752365
3043	TBC1D8	11138	0.001654308	-0.48073936
3044	CDC37	11140	0.007459231	-0.418761844
3045	PKIG	11142	0.005095479	-0.435657982
3046	MYST2	11143	2.31E-07	0.699181283
3047	DMC1	11144	1.35E-07	0.708208551
3048	HRASLS3	11145	0.007336777	-0.419525128
3049	GLMN	11146	0.00014172	0.560774047
3050	HHLA3	11147	8.56E-06	0.630154324
3051	WDR45	11152	5.00E-06	-0.641551769
3052	HYPE	11153	0.000276395	0.541411626
3053	AP4S1	11154	4.99E-05	0.588529622
3054	LDB3	11155	2.58E-07	0.697307928

3055	LSM6	11157	0.004001486	-0.445927814
3056	NUDT6	11162	2.73E-08	0.733173297
3057	NUDT5	11164	0.007045612	-0.421410267
3058	NUDT3	11165	0.00963653	0.406606872
3059	SOX21	11166	0.008911061	0.410341164
3060	ADAMTS6	11174	0.002849445	0.459835042
3061	BAZ2A	11176	0.00296182	-0.458306668
3062	LZTS1	11178	2.88E-07	0.69544508
3063	MAP4K5	11183	0.005132155	-0.435352173
3064	RASSF1	11186	1.03E-07	0.712553487
3065	PKP3	11187	0.00103846	-0.497679894
3066	NISCH	11188	0.002647533	-0.462758098
3067	PTENP1	11191	0.000158274	0.557662174
3068	KLK8	11202	0.002945793	-0.458518048
3069	AKAP11	11215	0.005313894	-0.433843079
3070	DDX20	11218	1.41E-08	0.743083343
3071	MRPL3	11222	0.002385194	-0.466959242
3072	RPL35	11224	4.48E-05	-0.591294821
3073	GALNT5	11227	0.000111958	0.567299594
3074	RNF24	11237	0.006792688	-0.423074497
3075	CA5B	11238	1.73E-10	0.802249142
3076	PADI2	11240	3.30E-07	0.69305497
3077	PMF1	11243	0.002306224	-0.468175092
3078	NXPH4	11247	1.03E-09	0.778759443
3079	NXPH3	11248	1.57E-10	0.803814548
3080	Unknown	11252	0.00140489	-0.486817532
3081	MAN1B1	11253	3.33E-05	-0.598662242
3082	HRH3	11255	0.000191859	0.552194597
3083	XPOT	11260	0.001816179	-0.47717663
3084	CHP	11261	3.35E-05	-0.598478053
3085	DUSP12	11266	0.007909628	-0.416040953
3086	SNF8	11267	0.001844063	-0.476583115
3087	NRM	11270	1.45E-07	0.70701427
3088	ATXN2L	11273	9.21E-11	0.810868348
3089	KLHL2	11275	0.000421545	-0.528271482
3090	AP1GBP1	11276	0.006803385	-0.422998809
3091	TREX1	11277	0.007701934	-0.41724168
3092	SCN11A	11280	5.20E-11	0.819825331
3093	POU6F2	11281	6.23E-07	0.681872831
3094	MGAT4B	11282	0.000933247	-0.501489791
3095	PNKP	11284	0.007939948	-0.41585564
3096	B4GALT7	11285	8.17E-06	-0.631153112
3097	SLCO2B1	11309	0.000215172	0.548851074
3098	VPS45	11311	0.009685316	-0.406363501
3099	CD300A	11314	1.13E-09	0.777489347

3100	PARK7	11315	0.001414411	-0.486532101
3101	RBPJL	11317	8.00E-10	0.782200883
3102	GPR182	11318	7.87E-11	0.81307076
3103	DDX42	11325	0.002399669	-0.466688637
3104	FKBP9	11328	7.64E-05	-0.577573573
3105	PHB2	11331	0.000499202	-0.522847706
3106	PDAP1	11333	0.000151873	0.558871005
3107	TUSC2	11334	2.70E-05	-0.603667976
3108	EXOC3	11336	0.000451488	-0.52608216
3109	GABARAP	11337	9.11E-05	-0.572955346
3110	U2AF2	11338	0.00431849	-0.442688177
3111	EXOSC8	11340	0.005429265	-0.432885508
3112	RNF13	11342	0.000886472	-0.503362911
3113	TWF2	11344	0.002015651	-0.473239317
3114	GABARAPL2	11345	0.004262637	-0.443262202
3115	CASC3	22794	0.001802762	-0.477455491
3116	LAMB4	22798	2.14E-09	0.768936676
3117	CLCA4	22802	3.56E-10	0.792747384
3118	COPZ1	22818	0.000570734	-0.518443175
3119	COPG	22820	0.004906827	-0.437288659
3120	MTF2	22823	0.0025563	-0.464177053
3121	DNAJC8	22826	0.004869969	-0.437617009
3122	PUF60	22827	0.001564235	-0.482784475
3123	RBM16	22828	0.005788699	-0.430143545
3124	NLGN4Y	22829	1.83E-07	0.703175366
3125	RNF44	22838	2.79E-05	-0.60289163
3126	ZNF507	22847	8.96E-06	0.629127219
3127	ADNP2	22850	0.000812496	-0.506405344
3128	LMTK2	22853	0.001357654	-0.488127488
3129	NLRP1	22861	0.002060416	0.472446454
3130	R3HDM2	22864	8.08E-06	-0.631396711
3131	SLITRK3	22865	2.38E-10	0.797916876
3132	FASTKD2	22868	0.000137381	-0.561631294
3133	SAPS1	22870	0.001440078	0.485897863
3134	SEC31A	22872	3.45E-05	-0.597786379
3135	INPP5F	22876	0.001200396	-0.492530087
3136	KIAA1012	22878	0.007190571	-0.42051882
3137	CLSTN1	22883	0.000126907	-0.563800782
3138	ABLIM3	22885	2.40E-05	0.606540433
3139	KIAA0907	22889	0.003324833	-0.453618142
3140	ZBTB1	22890	0.000139197	-0.561274032
3141	DIS3	22894	0.008923413	0.410260652
3142	CEP164	22897	0.009071109	-0.40944573
3143	ARHGEF15	22899	7.70E-10	0.782717301
3144	RUFY3	22902	0.007929349	-0.415923218

3145	BTBD3	22903	0.006430271	-0.425545434
3146	DHX30	22907	0.000316955	-0.537265052
3147	SACM1L	22908	8.68E-06	-0.629833324
3148	MTMR15	22909	0.006325403	-0.426220005
3149	RALY	22913	0.001786758	-0.477777252
3150	KLRK1	22914	4.60E-07	0.687217585
3151	ZP1	22917	0.000813493	0.506351639
3152	MSRB2	22921	0.006433826	-0.425519271
3153	ATF6	22926	5.89E-10	0.786038803
3154	HABP4	22927	0.002696412	0.461985905
3155	SEPHS1	22929	6.36E-05	0.582322765
3156	RAB3GAP1	22930	0.000140002	-0.561116121
3157	RAB18	22931	0.000765929	-0.508491614
3158	SIRT2	22933	0.00079384	0.507232111
3159	RPIA	22934	6.33E-05	-0.582428689
3160	ELL2	22936	2.50E-05	0.605573865
3161	SCAP	22937	0.000658277	-0.513622568
3162	SNW1	22938	0.000569761	-0.518503721
3163	DKK1	22943	0.002638132	0.462894852
3164	KIN	22944	0.005751105	0.430423583
3165	SLC4A1AP	22950	0.000720718	-0.510509674
3166	P2RX2	22953	4.12E-08	0.726897931
3167	SCMH1	22955	0.00150112	-0.484333131
3168	PAXIP1	22976	0.001463727	-0.485282664
3169	EFR3B	22979	5.26E-10	0.787568822
3170	MAST1	22983	2.43E-07	0.69830158
3171	PDCD11	22984	0.005043861	-0.436116161
3172	ACIN1	22985	0.005021979	-0.436307286
3173	AZI1	22994	0.001610237	-0.481737109
3174	RIMS1	22999	3.56E-06	0.648567881
3175	KIAA0265	23008	0.003624718	-0.450064545
3176	Unknown	23016	0.000584768	-0.517683434
3177	CNOT1	23019	0.000223223	-0.547778398
3178	ASCC3L1	23020	1.18E-06	-0.670030684
3179	TMCC1	23023	4.62E-05	-0.590442806
3180	JMJD2B	23030	1.65E-05	0.615414936
3181	MAST3	23031	0.000659152	-0.513564344
3182	USP33	23032	0.000617085	-0.515873198
3183	PHLPPL	23035	0.000282123	0.540767796
3184	ZNF292	23036	0.009270608	-0.408427036
3185	MYT1L	23040	6.00E-08	0.721016584
3186	TNIK	23043	3.49E-09	0.762561745
3187	FNBP1	23048	0.003794969	-0.448158497
3188	KIAA0913	23053	0.002219098	-0.469650709
3189	NCOA6	23054	0.00025846	-0.543385516

3190	ZNF609	23060	0.001015685	0.498490814
3191	TBC1D9B	23061	0.000169574	-0.555704342
3192	WAPAL	23063	0.001229468	-0.491704257
3193	KIAA0090	23065	0.006170827	-0.427284451
3194	SETD1B	23067	0.002576083	-0.463862523
3195	KIAA0082	23070	0.008849085	-0.410679234
3196	TXNDC4	23071	0.000291185	-0.539808238
3197	RRP1B	23076	7.84E-05	-0.57693097
3198	MYCBP2	23077	0.007495638	-0.418506236
3199	KIAA0241	23080	0.001965618	-0.474194509
3200	PPRC1	23082	0.003380175	-0.452935261
3201	ERC1	23085	2.15E-07	0.700427888
3202	ZC3H13	23091	0.008834594	-0.410759517
3203	CDC2L6	23097	0.005307841	-0.433901222
3204	DDN	23109	6.46E-10	0.784957029
3205	ATG2A	23130	3.50E-05	-0.597438305
3206	GPATCH8	23131	1.90E-05	0.612085145
3207	ZC3H3	23144	1.05E-09	0.778472076
3208	FCHO1	23149	0.000317805	-0.537186869
3209	FRMD4B	23150	0.003109391	-0.456387844
3210	DIP	23151	8.81E-05	0.573829057
3211	NCDN	23154	0.000401924	-0.529769171
3212	SNX13	23161	0.003212024	-0.455034333
3213	RTF1	23168	0.000627083	-0.51529811
3214	GPD1L	23171	0.000122935	-0.564679799
3215	METAP1	23173	0.000175008	-0.554815062
3216	ZCCHC14	23174	0.002696578	-0.461972975
3217	8-Sep	23176	0.005083919	-0.435767749
3218	CEP68	23177	0.006519085	-0.424931288
3219	DIP2A	23181	0.006256526	-0.426706744
3220	ANKRD15	23189	0.000598164	-0.516904116
3221	UBXD2	23190	3.50E-05	-0.597420915
3222	CYFIP1	23191	0.001997249	-0.473572991
3223	ATG4B	23192	0.000327547	-0.536287465
3224	MDN1	23195	0.005286988	-0.434077884
3225	FAM120A	23196	0.000795001	-0.507177266
3226	PSME4	23198	0.006759878	-0.423295702
3227	ATP11B	23200	6.40E-06	0.636399797
3228	PLEKHM2	23207	0.00018197	-0.553682719
3229	SYT11	23208	1.42E-09	0.774507341
3230	RRS1	23212	2.21E-06	0.658141784
3231	XPO6	23214	4.54E-05	-0.590995479
3232	BAT2D1	23215	0.002198617	-0.469985477
3233	DTX4	23220	0.000605703	0.516503123
3234	ARHGEF9	23229	0.002257504	0.468959568

3235	SNF1LK2	23235	0.000152682	-0.558703548
3236	KIAA0922	23240	4.81E-10	0.788650359
3237	ANKRD28	23243	0.000883377	-0.503487658
3238	PDS5A	23244	0.000169772	-0.555662192
3239	BOP1	23246	0.002495217	-0.465122128
3240	KIAA0460	23248	0.000456653	0.525722811
3241	OTUD3	23252	5.29E-08	0.723002357
3242	SCFD1	23256	0.000566233	-0.518692887
3243	CAMTA1	23261	0.003324399	-0.453628061
3244	MCF2L	23263	4.42E-08	0.725820944
3245	MGA	23269	7.25E-07	0.679055107
3246	TSPYL4	23270	1.67E-05	-0.615150594
3247	CAMSAP1L1	23271	0.006147406	-0.427464275
3248	LPHN3	23284	1.84E-07	0.703059256
3249	KIAA1107	23285	0.000265817	0.542545039
3250	AGTPBP1	23287	7.15E-06	0.634040987
3251	FBXW11	23291	0.00124912	-0.491111159
3252	SMG6	23293	3.93E-06	0.646547681
3253	ANKS1A	23294	0.005666511	-0.431078633
3254	MGRN1	23295	0.0059645	-0.428756313
3255	WSCD1	23302	0.000357594	0.533467812
3256	ACSL6	23305	2.27E-10	0.798671094
3257	ICOSLG	23308	1.30E-11	0.846045608
3258	SIN3B	23309	1.05E-05	0.625491234
3259	USP22	23326	7.54E-05	-0.577899092
3260	KIAA0984	23329	8.39E-09	0.750356247
3261	TTC28	23331	0.001617331	-0.48158542
3262	CLASP1	23332	0.003909972	0.446846861
3263	DPY19L1	23333	1.81E-09	0.771203104
3264	PHF15	23338	0.009648173	-0.406543157
3265	VPS39	23339	0.00140431	-0.48683613
3266	FAM62A	23344	0.00092714	-0.501744576
3267	SMCHD1	23347	0.008077391	-0.415035861
3268	DOCK9	23348	0.002273764	-0.468694616
3269	SR140	23350	0.004805681	-0.438202365
3270	UBR4	23352	0.00033502	-0.535536689
3271	UNC84A	23353	1.34E-07	-0.708363355
3272	KIAA0841	23354	6.96E-06	0.634603816
3273	ANGEL1	23357	0.004569283	-0.440320646
3274	USP24	23358	0.001031668	-0.497930778
3275	LARP1	23367	1.34E-05	-0.620066254
3276	PUM2	23369	0.009897829	-0.405287361
3277	CRTC1	23373	3.84E-08	0.727924062
3278	KIAA0776	23376	0.000560099	-0.519068692
3279	KIAA0947	23379	0.000527151	-0.521046756

3280	SMG5	23381	7.41E-08	0.717707495
3281	KIAA0892	23383	0.002148031	-0.470848824
3282	NUDCD3	23386	0.00453698	-0.440610283
3283	KIAA0999	23387	0.009413622	-0.407661259
3284	KIAA0368	23392	0.000379194	-0.531592611
3285	ADNP	23394	8.00E-05	-0.576355806
3286	LARS2	23395	1.35E-07	0.708248209
3287	ATP13A2	23400	0.000276551	-0.541378214
3288	FRAT2	23401	0.009976242	-0.40492666
3289	SIRT3	23410	7.13E-06	0.634100604
3290	SIRT1	23411	0.001391513	-0.487180132
3291	COMMD3	23412	5.90E-06	-0.638118086
3292	FREQ	23413	1.61E-09	0.77280365
3293	KCNH4	23415	0.003413834	0.452533216
3294	MLYCD	23417	1.57E-08	0.741554764
3295	CRB1	23418	1.87E-05	0.612493085
3296	TMED3	23423	0.001075582	-0.496398299
3297	TDRD7	23424	0.001145776	-0.49418737
3298	GRIP1	23426	0.005490924	-0.432398456
3299	SLC7A8	23428	0.000964344	0.500324276
3300	RYBP	23429	3.71E-07	-0.691092305
3301	AP4E1	23431	5.53E-09	0.756194196
3302	RHOQ	23433	0.000346143	0.534505028
3303	C3orf27	23434	2.46E-05	0.605989445
3304	TARDBP	23435	0.000156214	-0.558055179
3305	HARS2	23438	0.001486086	-0.484702384
3306	OTP	23440	0.001140471	0.494352798
3307	SF3B1	23451	0.000127166	-0.563744161
3308	ABCB9	23457	1.48E-07	0.706727992
3309	ABCA6	23460	6.75E-07	0.680409596
3310	GCAT	23464	0.000249559	-0.544463378
3311	PHF3	23469	0.000297284	-0.539198992
3312	ETHE1	23474	3.63E-05	0.596503151
3313	BRD4	23476	0.001039532	-0.497641869
3314	ISCU	23479	0.000743308	-0.509501874
3315	SEC61G	23480	0.001651046	-0.480808191
3316	TGDS	23483	2.00E-08	0.738025581
3317	LEPROTL1	23484	0.001561066	0.482882438
3318	CBX7	23492	1.79E-10	0.801879769
3319	MACF1	23499	3.66E-06	-0.647992817
3320	ZFYVE26	23503	1.95E-10	0.800756654
3321	RIMBP2	23504	5.59E-05	0.585715042
3322	TMEM131	23505	9.37E-05	-0.572179477
3323	KIAA0240	23506	0.003224875	-0.454861765
3324	KCTD2	23510	0.001368382	-0.487823871

3325	Unknown	23511	0.002148173	-0.470841161
3326	SUZ12	23512	0.001236171	-0.491507365
3327	SCRIB	23513	0.005866575	-0.429507655
3328	ANP32C	23520	1.26E-08	0.744700541
3329	RPL13A	23521	2.38E-05	-0.606787568
3330	PIK3R5	23533	9.23E-05	0.572582123
3331	OR52A1	23538	1.39E-07	0.707760043
3332	SEC14L2	23541	0.000531495	0.52077256
3333	SEZ6L	23544	2.29E-07	0.699297016
3334	SYNGR4	23546	4.41E-11	0.821918119
3335	LILRA4	23547	0.001177698	0.493210358
3336	DNPEP	23549	0.000256208	0.543647284
3337	PSD4	23550	4.36E-09	0.759565064
3338	RASD2	23551	0.007628182	0.417703008
3339	CCRK	23552	2.00E-05	0.610878668
3340	SNAPIN	23557	0.000886463	-0.503371841
3341	WBP2	23558	0.001335532	-0.488720782
3342	GTPBP4	23560	0.001524148	-0.483771873
3343	CLDN14	23562	1.32E-05	0.62038249
3344	ARL2BP	23568	3.07E-09	0.764247844
3345	PADI4	23569	7.75E-07	0.677863924
3346	CDC42EP4	23580	0.000255579	-0.543724507
3347	CASP14	23581	4.08E-07	0.689370233
3348	KLHDC2	23588	1.05E-05	-0.625514414
3349	LEMD3	23592	0.002991657	-0.457895699
3350	ORC3L	23595	0.001970877	-0.474090628
3351	PATZ1	23598	5.85E-05	-0.584447835
3352	CD2AP	23607	0.0076713	-0.417440391
3353	MKRN1	23608	8.00E-05	-0.576361184
3354	PYY2	23615	0.000590858	0.517333429
3355	TSSK2	23617	0.000111852	0.567335269
3356	Unknown	23619	1.76E-09	0.77157996
3357	NTSR2	23620	0.00591211	0.42917621
3358	RUSC1	23623	1.27E-05	0.621274302
3359	CBLC	23624	0.007963534	-0.415719281
3360	PRND	23627	0.000436579	0.527167053
3361	CA14	23632	5.39E-08	0.722722902
3362	SSBP2	23635	2.01E-08	0.737953915
3363	RABGAP1	23637	0.000194368	-0.551788039
3364	SNHG1	23642	0.000315287	-0.537431596
3365	ARFIP2	23647	8.28E-10	0.78177701
3366	ZKSCAN5	23660	0.001119545	-0.495012463
3367	TMEM2	23670	0.00040068	0.529878675
3368	SH3BP4	23677	0.002667302	-0.462468022
3369	C9orf4	23732	1.72E-07	0.704204313

3370	EID1	23741	0.00154571	-0.483252814
3371	SDF2L1	23753	0.000199757	-0.550990844
3372	PITPNB	23760	8.81E-06	0.629502745
3373	PISD	23761	4.28E-05	-0.592400229
3374	FLRT1	23769	0.002241331	0.469248697
3375	FKBP8	23770	0.000104788	0.56919845
3376	BRD1	23774	8.11E-05	-0.575984491
3377	MTCH1	23787	4.39E-05	-0.591766614
3378	Unknown	24142	2.04E-09	0.769589795
3379	PRPF6	24148	1.85E-05	-0.612677418
3380	C11orf41	25758	0.00311229	0.456350266
3381	CXorf27	25763	7.37E-06	0.633392699
3382	HYPK	25764	2.35E-07	0.698911713
3383	SLC24A2	25769	1.14E-08	0.746062814
3384	C22orf31	25770	2.55E-09	0.766596773
3385	C22orf24	25775	0.00025662	0.543598779
3386	RIPK5	25778	0.002692224	-0.462061742
3387	RASGRP3	25780	1.20E-05	0.622684407
3388	Unknown	25784	4.97E-08	0.724006818
3389	DGCR11	25786	1.58E-05	0.616413374
3390	CIZ1	25792	0.003382503	-0.452900632
3391	FBXO7	25793	0.000376703	-0.531795308
3392	RHBDD3	25807	0.000969425	-0.500142669
3393	ATXN10	25814	0.001666307	-0.480452053
3394	CCRN4L	25819	1.55E-08	0.741775047
3395	ARIH1	25820	0.001961255	-0.474278187
3396	MTO1	25821	2.74E-06	0.65386827
3397	DNAJB5	25822	4.19E-10	0.790592638
3398	TPSG1	25823	8.61E-07	0.675913423
3399	PRDX5	25824	0.001293066	-0.489870931
3400	BACE2	25825	0.00296182	-0.458300891
3401	TMEM184B	25829	0.000582656	-0.517792932
3402	SULT4A1	25830	1.32E-07	0.708523814
3403	HECTD1	25831	0.000205028	-0.550250398
3404	COG4	25839	0.001047475	-0.497348385
3405	ABTB2	25841	0.008996825	-0.409863854
3406	LOC25845	25845	0.004253144	-0.443374423
3407	ANAPC13	25847	0.000905133	-0.502655429
3408	WDR40A	25853	0.00603894	-0.428207214
3409	BRMS1	25855	5.84E-06	0.638363893
3410	DKFZP566E164	25858	2.13E-10	0.799544964
3411	DFNB31	25861	0.0008426	0.505128251
3412	USP49	25862	2.10E-06	0.659085612
3413	ABHD14A	25864	0.008814092	-0.41088737
3414	PRKD2	25865	0.001016508	-0.498451324

3415	C3orf17	25871	0.000836108	0.505392991
3416	RPL36	25873	0.004339219	-0.442484045
3417	BRP44	25874	0.006193174	-0.427127299
3418	WDSOF1	25879	0.008342862	-0.413521388
3419	WDR51A	25886	1.81E-08	0.739515666
3420	ZNF473	25888	4.73E-06	0.64270725
3421	FAM119B	25895	0.003926902	0.446661363
3422	HOM-TES-103	25900	6.66E-06	0.635555263
3423	CNOT10	25904	0.009754855	-0.40599982
3424	C1orf43	25912	3.80E-05	-0.595366751
3425	C3orf60	25915	0.006643451	-0.424067049
3426	THUMPD3	25917	0.002434451	-0.46613557
3427	RP13-122B23.3	25920	0.000698732	-0.511614547
3428	DKFZP564J086[illegible]	25923	0.000858586	-0.50449839
3429	NOL11	25926	1.12E-05	-0.624223234
3430	PTPN23	25930	0.000825725	0.505816303
3431	WWTR1	25937	0.001480356	-0.48484705
3432	SAMHD1	25939	0.00253694	-0.464480959
3433	FAM98A	25940	0.000331322	-0.535922384
3434	RWDD3	25950	0.00326731	-0.454348776
3435	SFRS18	25957	0.009358337	-0.407967985
3436	GPR124	25960	0.00024681	0.544816448
3437	KIAA1429	25962	0.000357718	-0.533452272
3438	TMEM87A	25963	0.000456733	-0.525711926
3439	C2CD2	25966	0.002845179	-0.459894604
3440	UNC50	25972	1.16E-07	-0.710627815
3441	MMACHC	25974	2.15E-10	0.799423803
3442	NECAP1	25977	2.77E-10	0.795935434
3443	C20orf4	25980	7.04E-05	0.579680424
3444	DNAH1	25981	0.001528643	0.483672708
3445	MIZF	25988	1.77E-05	-0.613795919
3446	SNED1	25992	2.54E-07	0.697572025
3447	HIGD1A	25994	0.000662437	-0.513382805
3448	REXO2	25996	0.002464887	-0.465653187
3449	IBTK	25998	0.001295519	-0.489800544
3450	TBC1D10B	26000	3.37E-09	0.763011318
3451	GORASP2	26003	0.002395803	-0.466758997
3452	C2CD3	26005	0.00443965	-0.441494935
3453	NELF	26012	0.003319065	0.453689334
3454	RPAP1	26015	1.48E-11	0.840812174
3455	UPF2	26019	0.000983794	-0.499629147
3456	LRP10	26020	0.0009278	-0.501713787
3457	PTCD1	26024	0.003660896	-0.449623923
3458	ACOT11	26027	2.81E-08	0.732708679
3459	ATRNL1	26033	0.00015971	0.557409277

3460	PIP3-E	26034	0.000228749	0.54706651
3461	CHD5	26038	8.05E-10	0.78211614
3462	SS18L1	26039	0.001381713	-0.487441494
3463	ZNF294	26046	0.000118149	-0.565815809
3464	CNTNAP2	26047	7.33E-09	0.75232837
3465	ZNF500	26048	0.005714541	-0.430687811
3466	SENP6	26054	0.001702078	-0.47966982
3467	ANKRD17	26057	0.001140199	-0.494365776
3468	APPL1	26060	0.000467327	-0.525023071
3469	LSM14A	26065	0.000347647	-0.534356809
3470	POLDIP2	26073	0.000880826	0.503579925
3471	KLK13	26085	0.00062689	0.515312898
3472	GPSM1	26086	4.34E-08	0.726114743
3473	HERC4	26091	0.001243801	-0.491276881
3474	CCDC9	26093	1.59E-09	0.772951907
3475	WDR21A	26094	0.008358249	-0.413398986
3476	C1orf77	26097	0.003752991	-0.448624225
3477	C1orf144	26099	7.20E-08	0.718169348
3478	WIPI2	26100	1.21E-05	-0.62244969
3479	PYGO1	26108	7.08E-05	0.579553832
3480	TANC2	26115	0.000161217	0.557121886
3481	WSB1	26118	0.002473112	-0.465508562
3482	LDLRAP1	26119	0.000742591	0.509550151
3483	PRPF31	26121	3.80E-05	-0.595364293
3484	EPC2	26122	0.001822859	-0.477035548
3485	TRPC4AP	26133	0.003374309	-0.453006061
3486	ZBTB20	26137	0.000346137	0.534512441
3487	IRF2BP1	26145	4.42E-07	0.687934533
3488	PHF19	26147	2.33E-10	0.798255032
3489	RIBC2	26150	0.000205172	0.550219678
3490	NAT9	26151	0.00820869	-0.41425531
3491	GTPBP5	26164	5.00E-06	0.641546204
3492	SENP3	26168	0.001774062	-0.478063057
3493	INTS1	26173	0.00023976	-0.545688322
3494	OR1F2P	26184	2.79E-06	0.653486713
3495	OR1C1	26188	0.002480979	0.465374588
3496	OR1A2	26189	2.20E-05	0.608654995
3497	FBXW2	26190	0.002377597	-0.467085234
3498	GMEB2	26205	0.001455934	-0.485518453
3499	OR1J4	26219	1.24E-06	0.669097466
3500	ARL5A	26225	0.002725694	-0.461570229
3501	Unknown	26226	7.64E-06	0.632650827
3502	B3GAT3	26229	4.24E-05	-0.592646396
3503	TIAM2	26230	6.35E-11	0.815923533
3504	FBXL4	26235	0.004010891	-0.445828191

3505	C6orf123	26238	2.50E-09	0.766823375
3506	LCE2B	26239	0.003820153	0.447871956
3507	OR4C1P	26242	5.54E-10	0.786873452
3508	OR2M4	26245	8.68E-05	0.574228021
3509	OR2L2	26246	2.72E-05	0.603525454
3510	OR2K2	26248	2.01E-11	0.835515253
3511	KCNG2	26251	2.87E-06	0.65295513
3512	OPTC	26254	3.09E-06	0.651396673
3513	CABYR	26256	6.58E-11	0.815320394
3514	NKX2-8	26257	0.006189532	-0.427159916
3515	PLDN	26258	0.000192192	-0.552102215
3516	TSPAN17	26262	0.000810845	-0.506494593
3517	FBXO10	26267	5.80E-09	0.755546473
3518	FBXO9	26268	0.001670131	-0.480367399
3519	PLA2G2D	26279	1.97E-07	0.701877418
3520	IL1RAPL2	26280	1.00E-07	0.712976435
3521	ERAL1	26284	0.000800025	0.506973533
3522	CLDN17	26285	0.000229937	0.546900333
3523	ANKRD2	26287	1.16E-08	0.745839345
3524	AK5	26289	2.95E-11	0.827733859
3525	GALNT8	26290	4.98E-06	0.641639156
3526	FGF21	26291	7.88E-06	0.631975253
3527	EHF	26298	0.004121787	-0.444678932
3528	GAPDHS	26330	8.87E-09	0.749576305
3529	OR7A17	26333	0.00588508	0.429358357
3530	OR5K1	26339	4.77E-09	0.758208093
3531	GNL3	26354	2.64E-06	-0.654546136
3532	C3orf28	26355	0.001930745	-0.474845446
3533	PPP1R14B	26472	0.001785711	-0.477801731
3534	OR8G2	26492	8.00E-10	0.782190193
3535	OR8B8	26493	1.30E-05	0.620785599
3536	OR8G1	26494	2.21E-06	0.658066675
3537	OR10A3	26496	5.16E-08	0.72341769
3538	PLEK2	26499	9.54E-11	0.81040364
3539	NARF	26502	0.005497003	-0.432338823
3540	SLC17A5	26503	2.12E-07	0.700604917
3541	CNNM4	26504	0.001083311	-0.496139666
3542	CNNM3	26505	6.11E-06	-0.637380415
3543	HEYL	26508	1.22E-10	0.807024085
3544	INTS6	26512	0.003330843	-0.453533762
3545	FXC1	26515	0.001405002	-0.486809418
3546	TIMM8B	26521	0.000674267	-0.512810265
3547	DAZAP1	26528	2.54E-05	-0.605197731
3548	OR11A1	26531	0.000288595	0.540095797
3549	OR10H3	26532	3.59E-06	0.648363925

3550	OR10H1	26539	0.003887211	0.447112779
3551	OR10D1P	26541	2.82E-11	0.828791999
3552	ITGB1BP2	26548	6.37E-09	0.754228243
3553	AATF	26574	0.003827729	-0.447782303
3554	RGS17	26575	2.29E-07	0.699308749
3555	SRPK3	26576	0.007293908	0.41981368
3556	BSCL2	26580	0.000111245	-0.56752532
3557	TBL2	26608	0.000416979	-0.528597036
3558	ELP4	26610	0.002388855	0.466872566
3559	OR7E19P	26651	2.04E-07	0.701278723
3560	OR7A5	26659	3.71E-11	0.824933407
3561	OR7C1	26664	0.002012656	0.473293594
3562	OR4D1	26689	0.004379424	0.442071596
3563	OR2H1	26716	1.56E-06	0.664884888
3564	OR1J2	26740	2.55E-05	0.605049973
3565	NUFIP1	26747	0.00103066	-0.497966203
3566	SH3YL1	26751	0.000147281	-0.55971673
3567	HAVCR1	26762	1.66E-06	0.663568936
3568	SNORA72	26775	0.000378165	0.53167309
3569	SNORA71A	26777	2.27E-08	0.736073634
3570	SNORA65	26783	6.58E-10	0.784727688
3571	SMR3A	26952	9.61E-07	0.673877047
3572	HBP1	26959	0.000422265	-0.528212231
3573	PABPC1	26986	9.05E-06	-0.628858621
3574	TRUB2	26995	0.004168718	-0.444231194
3575	FETUB	26998	1.57E-06	0.664728558
3576	TCL6	27004	0.001568059	0.482689984
3577	USP21	27005	0.003264084	-0.454388465
3578	FGF22	27006	1.30E-11	0.843500682
3579	KCNV1	27012	2.65E-05	0.604161211
3580	C2orf24	27013	0.000858586	-0.504496817
3581	NGFRAP1	27018	0.005819636	-0.429886707
3582	NPTN	27020	0.001104316	-0.495494914
3583	FOXD3	27022	1.28E-06	0.668539713
3584	ACAD8	27034	0.005920336	-0.429103189
3585	PKD2L2	27039	5.56E-08	0.722267098
3586	SND1	27044	4.64E-06	-0.643129538
3587	STAU2	27067	0.000875346	0.503806776
3588	PPA2	27068	0.000522378	-0.521369114
3589	LAMP3	27074	1.30E-11	0.844987471
3590	LYPD3	27076	1.57E-10	0.803809376
3591	RPUSD2	27079	0.009212434	0.408722465
3592	MTBP	27085	0.000618182	0.515780804
3593	UQCRQ	27089	8.02E-05	-0.576291657
3594	CACNG5	27091	0.000131841	0.56274264

3595	CACNG4	27092	0.000386094	0.531031474
3596	TAF5L	27097	0.008826264	-0.410822478
3597	EIF2AK1	27102	5.56E-06	-0.639354809
3598	SDCBP2	27111	5.81E-05	0.584612218
3599	DKKL1	27120	0.003356914	0.453219151
3600	AFF4	27125	0.000978293	-0.499815947
3601	HSPB7	27129	5.98E-08	0.721097471
3602	KCNH5	27133	0.000780583	0.507787597
3603	FILIP1	27145	0.001376506	0.487576932
3604	Unknown	27146	6.12E-11	0.816684049
3605	DENND2A	27147	8.84E-09	0.749628124
3606	STK36	27148	6.44E-05	0.581997673
3607	ZNF777	27153	0.000714458	-0.510824358
3608	BRPF3	27154	4.76E-10	0.788843102
3609	ASAHL	27163	0.00124912	0.49111052
3610	SALL3	27164	1.96E-08	0.738284406
3611	GLS2	27165	6.58E-07	0.680867087
3612	PRELID1	27166	0.007327298	-0.41961164
3613	TUBG2	27175	0.001760271	0.478353892
3614	IL1F8	27177	0.000551585	0.519593925
3615	SIGLEC9	27180	0.001036401	0.497751689
3616	DISC1	27185	8.41E-08	0.715731747
3617	IL17B	27190	3.25E-05	0.599213246
3618	OXGR1	27199	0.004710959	0.439027857
3619	GPR78	27201	0.000152682	0.558699749
3620	SERP1	27230	0.000137052	-0.561703602
3621	SULT1C4	27233	0.000359096	0.533317189
3622	ARFIP1	27236	0.001279369	-0.49024559
3623	ARHGEF16	27237	7.75E-09	0.751492293
3624	GPKOW	27238	0.000658277	-0.513618242
3625	GPR162	27239	0.000279896	0.541040141
3626	BBS9	27241	0.008701962	-0.411445127
3627	AHDC1	27245	0.001684745	0.480043236
3628	ZNF364	27246	0.002852372	-0.45978914
3629	NFU1	27247	0.000727181	-0.510208648
3630	C2orf30	27248	4.82E-08	0.724495042
3631	C2orf25	27249	0.001043486	-0.497487377
3632	Unknown	27251	1.98E-05	0.611192504
3633	SULT1B1	27284	1.94E-06	0.660656712
3634	RBMXL2	27288	1.13E-11	0.852113186
3635	RND1	27289	7.05E-07	0.679551196
3636	SMPDL3B	27293	2.91E-07	0.69523482
3637	C20orf10	27296	2.45E-08	0.734861646
3638	ADAMDEC1	27299	2.13E-09	0.768976576
3639	BMP10	27302	2.16E-05	0.609104517

3640	MOCS3	27304	1.53E-10	0.804261418
3641	PGDS	27306	1.07E-06	0.671926194
3642	RAB30	27314	1.04E-06	0.672440789
3643	PCDH11X	27328	0.000471398	0.524720355
3644	P2RY10	27334	0.006846769	0.422724779
3645	EIF3K	27335	0.000153707	-0.558493917
3646	UTP20	27340	6.29E-11	0.816240399
3647	KCNMB4	27345	0.000415394	0.52872647
3648	TMEM97	27346	0.008636948	-0.411807985
3649	D15Wsu75e	27351	0.001591642	0.482161574
3650	HTRA2	27429	0.00826711	0.413930913
3651	TOR2A	27433	0.000731903	-0.510007339
3652	POLM	27434	0.004866884	-0.43766126
3653	EML4	27436	0.001756258	-0.478438637
3654	CECR5	27440	0.000125689	-0.564064378
3655	CECR2	27443	7.86E-09	0.751280926
3656	IGHV7-81	28378	3.50E-05	0.597433597
3657	IGHV1-69	28461	0.001571543	0.482613677
3658	NKIRAS2	28511	1.22E-06	0.6694864
3659	CDH19	28513	6.04E-09	0.754943933
3660	Unknown	28562	0.000232011	0.546634895
3661	Unknown	28595	0.001094777	0.495783392
3662	Unknown	28683	0.005356296	0.433470471
3663	Unknown	28797	0.001860163	0.476262125
3664	Unknown	28826	4.81E-10	0.788678939
3665	CCDC22	28952	6.23E-05	-0.582811723
3666	MAPBPIP	28956	0.006182167	-0.427210025
3667	MRPS28	28957	0.006856946	-0.422652404
3668	CCDC56	28958	0.00495392	-0.436849542
3669	BZW2	28969	0.006477966	-0.425213107
3670	C11orf54	28970	0.001249463	-0.491090709
3671	C11orf67	28971	0.007204611	-0.420428194
3672	SPCS1	28972	0.003150704	-0.45583464
3673	MRPS18B	28973	0.005025329	-0.436267027
3674	C19orf53	28974	0.004188587	-0.44402896
3675	ACAD9	28976	0.00449947	-0.440953037
3676	FLVCR1	28982	0.003531793	-0.451153694
3677	MCTS1	28985	0.008421989	-0.413006469
3678	DBNL	28988	0.001763033	-0.478294464
3679	COMMD5	28991	0.005006459	-0.436426078
3680	MACROD1	28992	0.000208321	-0.549794944
3681	KLF15	28999	1.30E-11	0.845482002
3682	PRO1768	29018	5.46E-05	0.586289452
3683	C20orf30	29058	0.000708672	-0.51113792
3684	ZC3H7A	29066	0.000948439	-0.500900396

3685	SETD2	29072	3.76E-06	-0.647471383
3686	MED4	29079	0.004429567	-0.44158761
3687	PHPT1	29085	0.001750895	-0.47855947
3688	THYN1	29087	0.005817298	-0.429912196
3689	HSPC157	29092	0.002776957	0.460795408
3690	MRPL22	29093	0.002603325	-0.463447378
3691	CNIH4	29097	0.001381103	-0.487460981
3692	COMMD9	29099	0.000638537	0.514652068
3693	SSU72	29101	0.001292079	-0.489917254
3694	RNASEN	29102	5.26E-05	-0.587213781
3695	N6AMT1	29104	2.78E-10	0.795862294
3696	C16orf80	29105	0.003625401	-0.450052569
3697	NXT1	29107	0.005669042	-0.43104351
3698	TAGLN3	29114	3.15E-07	0.693852927
3699	SAP30BP	29115	0.004245592	-0.443459135
3700	DDX25	29118	1.13E-09	0.777489665
3701	CTNNA3	29119	0.000125073	0.564210903
3702	ANKRD11	29123	0.00165015	-0.480831139
3703	LGALS13	29124	3.80E-06	0.647258653
3704	C11orf21	29125	2.05E-09	0.769522589
3705	CD274	29126	2.33E-05	0.607291971
3706	USP25	29761	9.53E-05	0.571732566
3707	DKFZP434P211	29774	5.08E-10	0.788000361
3708	PARVB	29780	1.49E-05	0.61764201
3709	OLA1	29789	0.002375859	-0.467120622
3710	UCRC	29796	4.60E-05	-0.590555696
3711	DKFZp434K191	29797	3.06E-06	0.651587944
3712	YPEL1	29799	2.91E-06	0.652629897
3713	ZDHHC8	29801	0.003109391	-0.456392003
3714	REPIN1	29803	8.73E-05	-0.574067733
3715	ICOS	29851	8.18E-06	0.631108959
3716	ALG5	29880	0.000650724	-0.514046473
3717	ANAPC2	29882	0.004674282	-0.439386018
3718	STRN4	29888	0.004100676	-0.444883789
3719	GNL2	29889	0.001970737	-0.47409844
3720	SAC3D1	29901	1.05E-07	0.712123949
3721	EEF2K	29904	0.005772776	-0.430263609
3722	ST8SIA5	29906	4.56E-10	0.789340753
3723	UBIAD1	29914	0.001787733	-0.477748574
3724	SNX11	29916	1.28E-06	0.668548976
3725	GMPPA	29926	0.001568059	-0.48269195
3726	SEC61A1	29927	7.76E-05	-0.577193109
3727	TIMM22	29928	7.04E-09	0.752867354
3728	PCDHB1	29930	5.01E-11	0.820519955
3729	LOH3CR2A	29931	4.89E-09	0.75786719

3730	NENF	29937	4.85E-06	0.642195827
3731	SERTAD3	29946	7.14E-07	0.679310525
3732	DNMT3L	29947	0.004941995	0.436983371
3733	FTSJ2	29960	0.003262165	-0.454415151
3734	A1CF	29974	1.53E-06	0.665256347
3735	UBQLN2	29978	8.68E-05	-0.57423186
3736	UBQLN1	29979	0.00064172	-0.514496575
3737	SLC39A2	29986	1.79E-08	0.739619492
3738	SLC2A8	29988	0.007843156	-0.416431725
3739	OBP2A	29991	3.52E-05	0.597271154
3740	BAZ2B	29994	0.00273399	-0.461434414
3741	GLTSCR2	29997	0.000398404	-0.530059753
3742	FSCN3	29999	4.67E-06	0.642996653
3743	TBX21	30009	1.04E-09	0.778664043
3744	TLX3	30012	6.35E-11	0.816097179
3745	RAX	30062	2.64E-10	0.796619161
3746	SOX8	30812	6.65E-11	0.815150205
3747	KCNIP2	30819	2.09E-09	0.769235387
3748	KCNIP1	30820	7.81E-08	0.716895315
3749	CXXC1	30827	3.98E-09	0.760750318
3750	ZNF354C	30832	3.10E-06	0.651335369
3751	NT5C	30833	0.005792806	-0.430090863
3752	ZNRD1	30834	0.002565595	-0.464029754
3753	CD209	30835	4.17E-11	0.822570624
3754	DNTTIP2	30836	2.04E-09	0.769605605
3755	EHD2	30846	3.78E-08	0.728136446
3756	TAX1BP3	30851	5.09E-05	-0.588025755
3757	KLK14	43847	4.29E-10	0.790166213
3758	KLK12	43849	4.57E-05	0.590758925
3759	SCAPER	49855	0.000199611	0.551015852
3760	WDR8	49856	0.008392045	-0.413187623
3761	CLDN20	49861	1.06E-08	0.747077513
3762	MINK1	50488	0.006756881	0.423317822
3763	DUOX2	50506	1.24E-07	0.709609212
3764	NOX3	50508	0.004807943	0.438174121
3765	PODXL2	50512	0.005130813	-0.435367057
3766	1-Dec	50514	0.006283344	0.42651911
3767	CHST11	50515	3.66E-10	0.79238262
3768	IL20	50604	2.35E-08	0.735491517
3769	IL22	50616	7.69E-05	0.57742363
3770	ITSN2	50618	0.000347107	-0.534411195
3771	CUZD1	50624	6.81E-06	0.63510341
3772	CYHR1	50626	0.001028993	-0.498026849
3773	TMEM16G	50636	0.00019475	0.551731357
3774	ARHGEF3	50650	0.004420408	-0.441688764

3775	RDH8	50700	0.001165213	0.493584363
3776	IRX4	50805	3.39E-13	0.884829446
3777	AK3	50808	9.00E-05	-0.573295834
3778	COPS7A	50813	0.000369118	-0.532414375
3779	TAS2R4	50832	8.35E-08	0.715869284
3780	TAS2R7	50837	0.000716316	0.510730916
3781	TAS2R13	50838	7.74E-06	0.632351322
3782	DHH	50846	3.14E-11	0.826884877
3783	F11R	50848	5.77E-05	-0.584820073
3784	TRAT1	50852	4.23E-05	0.592702825
3785	VILL	50853	0.001260308	-0.490799543
3786	HNT	50863	0.000264963	0.542648644
3787	HEBP1	50865	0.000518216	-0.521639516
3788	SLC35B3	51000	0.001169136	-0.493463766
3789	TPRKB	51002	0.000100487	-0.57032344
3790	MED31	51003	0.009042995	-0.409629811
3791	COQ6	51004	0.001468721	-0.485163742
3792	AMDHD2	51005	0.000121352	-0.565056788
3793	SLC35C2	51006	0.000502255	-0.522652057
3794	ASCC1	51008	0.001041277	0.497568964
3795	FAHD2A	51011	4.50E-06	-0.643762739
3796	EXOSC1	51013	0.000104831	-0.569175785
3797	TMED7	51014	0.007453309	-0.418802906
3798	ISOC1	51015	0.001004462	0.498890776
3799	MRPS16	51021	0.001251359	-0.49103235
3800	FIS1	51024	6.08E-05	-0.583448669
3801	Magmas	51025	0.008850185	-0.410664075
3802	FAM18B	51030	0.000159628	-0.557428432
3803	LOC51035	51035	0.000619357	-0.51570844
3804	ZNF593	51042	0.000246166	-0.544903593
3805	ST8SIA3	51046	8.69E-05	0.574200579
3806	PI15	51050	4.55E-05	0.590865494
3807	PRLH	51052	0.009093825	0.409322811
3808	FAM135B	51059	1.90E-10	0.801100153
3809	TXNDC11	51061	0.001025339	0.498146413
3810	FAM26B	51063	2.29E-10	0.798523927
3811	C3orf32	51066	1.46E-09	0.774024602
3812	YARS2	51067	0.008378473	-0.413271797
3813	NOSIP	51070	0.008028238	-0.415351738
3814	TXNDC14	51075	0.000359208	-0.533296586
3815	CUTC	51076	0.0001305	-0.563021286
3816	NDUFA13	51079	1.03E-05	-0.626051714
3817	POLR1D	51082	0.002093429	-0.471840925
3818	MLXIPL	51085	1.66E-10	0.802958923
3819	SIDT2	51092	0.003877611	-0.447211217

3820	ADIPOR1	51094	0.003560108	0.450824067
3821	IFT52	51098	2.71E-06	0.654110985
3822	ABHD5	51099	0.000659574	0.513531822
3823	NDUFAF1	51103	0.004869087	-0.4376289
3824	PHF20L1	51105	0.001120558	0.494979017
3825	TFB1M	51106	6.66E-11	0.815106892
3826	METTL9	51108	0.001481368	-0.484814259
3827	RDH11	51109	0.009692888	-0.406318832
3828	TTC15	51112	0.001847741	-0.476507383
3829	FAM82B	51115	0.008127391	-0.414732966
3830	COQ4	51117	0.006339976	-0.426127756
3831	RPL26L1	51121	0.000550874	-0.51963658
3832	ZNF706	51123	0.000229882	-0.546918598
3833	SAR1B	51128	0.006728238	-0.423505856
3834	ASB3	51130	0.000197131	0.551375125
3835	PHF11	51131	0.000348295	0.534291181
3836	LOC51136	51136	0.006616432	0.424269001
3837	LOC51145	51145	0.000877323	0.503722094
3838	A4GNT	51146	0.00253694	0.464476882
3839	ING4	51147	8.80E-06	0.629530793
3840	CERCAM	51148	0.000383479	0.531248195
3841	LOC51149	51149	0.008443816	-0.41287383
3842	LOC51152	51152	3.07E-07	0.694295224
3843	MRTO4	51154	0.005989168	-0.428580742
3844	SERPINA10	51156	4.37E-07	0.688169851
3845	ZNF580	51157	1.88E-09	0.770722706
3846	VPS28	51160	0.000656122	-0.513742589
3847	DBR1	51163	0.000930039	0.501622034
3848	DCTN4	51164	0.000571202	-0.518413813
3849	CYB5R4	51167	5.28E-09	0.756806195
3850	MYO15A	51168	0.001383163	0.487402934
3851	TUBD1	51174	0.004217852	-0.443733156
3852	LEF1	51176	0.006789757	-0.423096175
3853	HAO2	51179	1.46E-05	0.618225282
3854	CRBN	51185	0.000711138	-0.510992363
3855	SS18L2	51188	0.002685526	-0.462194434
3856	C9orf53	51198	8.77E-10	0.781106072
3857	CLDN18	51208	3.91E-07	0.690146558
3858	LUZP4	51213	9.17E-10	0.780443537
3859	C14orf91	51217	5.88E-05	0.584284728
3860	TCEB3B	51224	0.000755715	0.508962395
3861	COPZ2	51226	3.95E-08	0.727527122
3862	TMEM85	51234	0.00214038	-0.471007253
3863	C8orf30A	51236	2.11E-07	0.700733767
3864	C3orf19	51244	8.33E-05	-0.575293737

3865	PAIP2	51247	0.000634985	-0.514836688
3866	PDZD11	51248	0.007488109	-0.418573585
3867	C6orf203	51250	0.003217301	-0.45495946
3868	NT5C3	51251	0.00694703	-0.42205427
3869	MRPL51	51258	0.008673264	-0.411601278
3870	CXorf26	51260	6.65E-05	-0.581117515
3871	MRPL27	51264	0.000557804	-0.519209413
3872	CDKL3	51265	5.26E-07	0.684925412
3873	CLEC1B	51266	3.25E-05	0.599269445
3874	CLEC1A	51267	1.09E-08	0.746678766
3875	TFDP3	51270	1.60E-11	0.83840234
3876	BET1L	51272	0.002257504	-0.468955126
3877	KLF3	51274	0.001108111	-0.495384065
3878	SCAND1	51282	0.003428155	-0.452351993
3879	RASL12	51285	4.57E-10	0.789315998
3880	CHCHD8	51287	0.000767714	-0.508399783
3881	RXFP3	51289	1.20E-09	0.776709489
3882	CD320	51293	0.0066939	-0.423726894
3883	PCDH12	51294	6.59E-07	0.680846179
3884	ECSIT	51295	0.008416196	-0.413054061
3885	PLUNC	51297	5.05E-10	0.788083654
3886	THEG	51298	0.000475095	0.524471569
3887	NRN1	51299	0.007268796	0.419967333
3888	C3orf1	51300	0.000390625	-0.530680764
3889	GCNT4	51301	1.74E-08	0.740095831
3890	KCNK9	51305	4.95E-09	0.75768061
3891	C5orf5	51306	0.002615013	-0.463274184
3892	FAM53C	51307	6.55E-05	-0.581549632
3893	TLR8	51311	0.000107078	0.568596216
3894	Unknown	51314	0.000160644	0.557230604
3895	PHF21A	51317	0.000290104	-0.539937996
3896	MRPL35	51318	1.81E-05	0.613238202
3897	AMZ2	51321	0.000308934	-0.538027738
3898	WAC	51322	4.23E-07	-0.688715551
3899	SPG21	51324	0.00095689	-0.500575691
3900	ARL17P1	51326	0.008889992	-0.4104555
3901	ERAF	51327	2.43E-08	0.734964609
3902	ARL6IP4	51329	0.002479434	-0.46540097
3903	TNFRSF12A	51330	8.98E-08	0.714667147
3904	SPTBN5	51332	3.28E-05	0.598975306
3905	MS4A4A	51338	0.00049655	0.523015608
3906	FZR1	51343	2.07E-07	0.701092651
3907	TAOK3	51347	1.72E-08	0.740266602
3908	MBTPS2	51360	0.00010801	-0.568359845
3909	HOOK1	51361	0.008343445	-0.4135135

3910	CDC40	51362	0.003832889	-0.447707913
3911	UBR5	51366	0.00566756	-0.431058491
3912	POP5	51367	0.000638052	-0.514679868
3913	TEX264	51368	0.003647423	-0.449791227
3914	POMP	51371	0.000103987	-0.569410485
3915	CCDC72	51372	4.38E-06	-0.644296532
3916	C2orf28	51374	0.000866943	-0.504152487
3917	CRLF3	51379	0.003560108	-0.450819884
3918	WNT16	51384	0.000936858	0.501336265
3919	EIF3EIP	51386	7.93E-05	-0.576627804
3920	RWDD1	51389	0.009742754	-0.406064962
3921	AIG1	51390	0.004282714	-0.443048399
3922	C19orf56	51398	0.000232027	-0.546626862
3923	TRAPPC4	51399	0.005868932	-0.429484866
3924	BIN2	51411	0.000670176	0.51299878
3925	AMOTL2	51421	0.006674161	-0.423859731
3926	DDX41	51428	0.000719812	-0.510576811
3927	C1orf9	51430	0.007250727	-0.420120274
3928	ANAPC5	51433	0.000117649	-0.565938951
3929	MAGEC2	51438	1.06E-08	0.747074935
3930	FAM8A1	51439	0.009215708	-0.4087032
3931	YTHDF2	51441	6.06E-06	-0.637568699
3932	IHPK2	51447	9.68E-06	-0.627375511
3933	LCMT1	51451	0.000400102	-0.529924495
3934	REV1	51455	0.005061888	-0.435953591
3935	ISYNA1	51477	0.000297697	-0.539155704
3936	HSD17B7	51478	0.008477357	-0.412692102
3937	ANKFY1	51479	0.004022289	-0.445705741
3938	C9orf114	51490	0.009206028	-0.408760571
3939	HSPC111	51491	1.92E-05	0.611841794
3940	CWC15	51503	1.09E-10	0.808517901
3941	HSPC152	51504	0.001251073	-0.491045065
3942	UFC1	51506	0.00011179	-0.567355193
3943	C20orf43	51507	0.002907773	-0.459033624
3944	NCKIPSD	51517	3.23E-07	0.693400084
3945	LARS	51520	0.000597098	-0.516978925
3946	TMEM14C	51522	0.001705914	-0.479580773
3947	CXXC5	51523	0.001829004	-0.476904343
3948	TMEM138	51524	0.001082882	-0.496157442
3949	C14orf100	51528	0.007381065	-0.419236221
3950	ANAPC11	51529	7.23E-05	-0.579011137
3951	PHF7	51533	9.57E-11	0.81035211
3952	ZCCHC17	51538	0.000170345	-0.555570434
3953	VPS54	51542	0.002385699	-0.466938132
3954	SIRT6	51548	7.40E-08	0.717749123

3955	LARP7	51574	0.000324482	-0.536559145
3956	AZIN1	51582	0.006562285	-0.424628304
3957	TRIM33	51592	0.00037513	-0.531930083
3958	ARS2	51593	0.000136239	-0.561857949
3959	NAG	51594	0.001014302	-0.498543584
3960	CUTA	51596	1.87E-05	-0.612479945
3961	LSR	51599	0.00861617	-0.411923569
3962	LIPT1	51601	0.003793032	-0.44819167
3963	NOP5/NOP58	51602	0.004677154	-0.439358407
3964	KIAA0859	51603	0.002744604	-0.461291798
3965	PIGT	51604	0.000390437	0.530700055
3966	ATP6V1H	51606	0.008626198	0.411872526
3967	TAF9B	51616	0.001244343	-0.491256044
3968	HMP19	51617	5.80E-09	0.755524592
3969	UBE2D4	51619	3.58E-10	0.792673112
3970	KLF13	51621	0.003703182	-0.449160174
3971	C7orf28A	51622	1.04E-05	0.625820682
3972	LUC7L2	51631	9.63E-05	-0.571444558
3973	RBMX2	51634	0.009271793	-0.408415353
3974	DHRS7	51635	0.001397034	0.487021989
3975	C14orf166	51637	0.000108368	-0.56825383
3976	SF3B14	51639	0.008346152	-0.413480198
3977	TMBIM4	51643	0.000406073	-0.52944976
3978	FAM96B	51647	0.004355821	-0.442310503
3979	MRPS33	51650	0.000106112	-0.568835946
3980	PTRH2	51651	0.000943251	-0.50107145
3981	CDK5RAP1	51654	0.002696578	-0.461977671
3982	BRP44L	51660	0.000149108	-0.559357415
3983	ZFR	51663	0.000254336	-0.543872982
3984	ASB4	51666	5.88E-11	0.817672792
3985	NUB1	51667	0.001725589	-0.479128484
3986	C1orf41	51668	0.002252398	-0.46904132
3987	TMEM66	51669	0.007772967	-0.416838362
3988	OAZ3	51686	1.91E-10	0.80101557
3989	LSM7	51690	0.005089832	-0.435719776
3990	CPSF3	51692	0.004217852	-0.443747898
3991	HECA	51696	0.00047739	-0.52432453
3992	NLK	51701	0.002269357	-0.468764615
3993	PADI3	51702	4.05E-05	0.593807677
3994	SELT	51714	0.001005564	-0.498851827
3995	UIMC1	51720	0.006593101	-0.424429394
3996	FBXO40	51725	0.000823632	0.50590322
3997	SEPX1	51734	0.00013895	0.561329361
3998	GHRL	51738	1.06E-05	0.625349951
3999	WWOX	51741	2.68E-07	0.696601846

4000	CD244	51744	3.24E-09	0.763496726
4001	Unknown	51750	2.72E-08	0.733215126
4002	SKIP	51763	0.000808775	0.506593483
4003	GNG13	51764	0.000105209	0.569056597
4004	TM7SF3	51768	0.004768385	-0.438507514
4005	RSF1	51773	0.001267881	-0.490558675
4006	MYOZ2	51778	8.97E-10	0.780772363
4007	COQ3	51805	0.00336205	0.453155117
4008	TUBA8	51807	2.28E-09	0.768121234
4009	GALNT7	51809	0.00385744	-0.447428242
4010	BTBD1	53339	0.002315096	-0.468029159
4011	NUDT9	53343	0.002082024	-0.472048291
4012	TM6SF2	53345	0.002533448	0.464550526
4013	TM6SF1	53346	0.000153433	0.558551194
4014	UBASH3A	53347	2.96E-11	0.827615109
4015	ZFYVE1	53349	0.003854951	-0.447468048
4016	NUP54	53371	0.001669858	-0.480378187
4017	TPCN1	53373	0.006894973	-0.422399325
4018	BCMO1	53630	3.01E-10	0.794899438
4019	PRKAG3	53632	6.16E-08	0.720590818
4020	PTOV1	53635	0.000700932	-0.511511166
4021	FXYD7	53822	5.48E-10	0.787014835
4022	FXYD4	53828	0.000104831	0.56918099
4023	GPR84	53831	2.03E-11	0.83534175
4024	C11orf24	53838	0.006445275	-0.425436235
4025	MUPCDH	53841	6.36E-08	0.720074784
4026	MYO3A	53904	0.002578854	0.463799029
4027	DUOX1	53905	0.003413834	0.45253044
4028	RAB24	53917	0.000438373	-0.527034341
4029	SLCO1C1	53919	2.72E-07	0.696353187
4030	PPIL3	53938	0.003150183	-0.455847998
4031	A4GALT	53947	0.000632477	0.514975309
4032	CPSF2	53981	0.000920825	0.501990165
4033	PCBP3	54039	2.26E-11	0.833503655
4034	C21orf58	54058	2.61E-10	0.796766345
4035	C21orf49	54067	1.66E-10	0.803066365
4036	C21orf45	54069	0.009296448	-0.408273699
4037	C21orf42	54072	0.001988474	0.473766648
4038	C21orf37	54076	7.01E-09	0.752920446
4039	C21orf23	54088	0.000456285	0.525751315
4040	C21orf22	54089	0.000868853	0.504069481
4041	SETD4	54093	0.002465115	-0.465644621
4042	RIPK4	54101	0.000691772	-0.511947329
4043	CHRAC1	54108	0.007687291	-0.417331089
4044	C21orf74	54143	0.004766497	0.438532499

4045	MRPL39	54148	0.007196196	-0.420483546
4046	C21orf91	54149	0.002186316	-0.47019869
4047	NANS	54187	0.004348296	-0.442392911
4048	CYCS	54205	0.008653046	-0.411703719
4049	KCNK10	54207	3.21E-07	0.693502787
4050	TREM1	54210	2.05E-06	0.659615859
4051	GPR85	54329	2.26E-10	0.798725853
4052	DPM3	54344	0.000114408	-0.566719018
4053	SLC38A2	54407	0.000230533	-0.546820398
4054	DNAJC10	54431	0.001495681	-0.484470526
4055	YIPF1	54432	0.001723536	0.479177914
4056	NOLA1	54433	0.002381013	-0.467026296
4057	SEMA5B	54437	5.63E-05	0.585477305
4058	CXorf9	54440	7.52E-05	0.577963911
4059	RIN2	54453	0.000742591	-0.509547062
4060	ATAD2B	54454	0.008789655	-0.411007532
4061	FBXO42	54455	6.21E-07	0.681941065
4062	MOV10L1	54456	2.55E-09	0.76659221
4063	PRR13	54458	5.43E-06	-0.639858755
4064	MRPS21	54460	0.000651488	-0.514000741
4065	FBXW5	54461	0.000815965	-0.506238529
4066	KIAA1128	54462	5.89E-10	0.786026193
4067	XRN1	54464	0.004226156	-0.443651558
4068	FLJ20323	54468	0.000128011	-0.563548824
4069	ZFAND6	54469	0.000102853	-0.569714773
4070	ARMCX6	54470	0.000131305	-0.56285006
4071	TOLLIP	54472	0.005820193	-0.429877896
4072	DGCR8	54487	0.00533582	-0.433643623
4073	C11orf71	54494	0.000407189	-0.529359947
4074	TXNDC10	54495	0.002766018	-0.460965189
4075	Unknown	54496	0.007254575	-0.420073895
4076	HEATR5B	54497	2.39E-05	-0.606636209
4077	SMOX	54498	1.81E-07	0.703403381
4078	ZDHHC13	54503	3.96E-08	0.727486874
4079	EXOSC4	54512	0.001857344	-0.476322442
4080	DDX4	54514	0.000127284	0.563708654
4081	APBB1IP	54518	0.000117148	0.566062788
4082	ASNSD1	54529	1.58E-05	-0.616428943
4083	MIER2	54531	3.73E-11	0.824719765
4084	CCHCR1	54535	3.13E-07	0.693934221
4085	FAM35A	54537	3.08E-05	-0.600561274
4086	ROBO4	54538	2.12E-09	0.769060163
4087	NDUFB11	54539	0.0001565	-0.55800274
4088	CRCT1	54544	4.59E-08	0.725281905
4089	MTMR12	54545	0.004501556	-0.440930987

4090	SDK2	54549	1.52E-05	0.617270639
4091	EFCBP2	54550	3.14E-05	0.600083393
4092	WDR5B	54554	0.007884411	-0.416191097
4093	DDX49	54555	0.000214836	-0.548903971
4094	SCAND2	54581	2.23E-11	0.8338319
4095	EGLN1	54583	0.00722909	-0.420266491
4096	C9orf11	54586	0.000300149	0.538905444
4097	DDX56	54606	2.28E-08	0.735986628
4098	PARP14	54625	0.006148718	-0.427445794
4099	HES2	54626	6.21E-07	0.681944249
4100	KIAA1383	54627	8.16E-05	0.57583932
4101	TBC1D13	54662	1.91E-10	0.801005903
4102	LRRN3	54674	0.00436261	0.442239138
4103	CRLS1	54675	0.000655847	-0.513772352
4104	PH-4	54681	0.000478345	-0.524244554
4105	CNGB3	54714	1.67E-05	0.615077165
4106	A2BP1	54715	0.000813251	0.506372016
4107	BTN2A3	54718	0.008652977	0.411709155
4108	OTUD4	54726	1.93E-05	-0.611707965
4109	NKX1-1	54729	4.98E-07	0.685837991
4110	TMED9	54732	0.00015415	-0.558415202
4111	RAB39	54734	6.96E-05	0.579980582
4112	MPHOSPH8	54737	0.004180519	-0.44410868
4113	FEV	54738	0.001550248	0.483131702
4114	Unknown	54741	0.003346659	-0.453341894
4115	FBLIM1	54751	0.003856108	0.447445817
4116	FNDC8	54752	4.28E-09	0.759795561
4117	FAM22F	54754	2.32E-05	0.607415034
4118	PCSK4	54760	2.66E-11	0.82991495
4119	ROPN1	54763	3.28E-05	0.598974108
4120	ZRANB1	54764	0.002693327	-0.462035594
4121	TRIM44	54765	5.78E-06	-0.638588927
4122	BTG4	54766	4.67E-06	0.643003982
4123	RNF111	54778	0.009608067	-0.40674078
4124	ALKBH4	54784	1.19E-09	0.77682064
4125	C17orf59	54785	9.28E-09	0.74896243
4126	MED18	54797	0.004253144	-0.443371014
4127	DCHS2	54798	7.14E-11	0.814307173
4128	KLHL24	54800	0.002821951	-0.46019321
4129	TRIT1	54802	0.000363365	-0.532891882
4130	DYM	54808	0.001290635	-0.489957735
4131	ZNF562	54811	0.008051899	-0.415216114
4132	AFTPH	54812	0.000471106	0.524755713
4133	KLHL28	54813	0.000529229	-0.520905525
4134	ERCC6L	54821	1.84E-09	0.77098751

4135	TRPM7	54822	9.98E-05	0.570514071
4136	FAM55D	54827	4.57E-08	0.725338808
4137	BEST2	54831	5.03E-11	0.820339684
4138	VPS13C	54832	0.004722267	-0.438914222
4139	C10orf26	54838	5.55E-05	-0.585902764
4140	APTX	54840	0.001445977	0.485752016
4141	BIVM	54841	0.000447142	-0.526375723
4142	RBM35A	54845	0.000305818	-0.538318199
4143	SIDT1	54847	7.72E-09	0.751562539
4144	FLJ20184	54848	1.32E-05	0.620506788
4145	DEF8	54849	0.004313009	-0.442744473
4146	ANKRD49	54851	2.03E-08	0.737756251
4147	PAQR5	54852	5.99E-08	0.721044248
4148	WDR55	54853	1.70E-10	0.802596496
4149	GDPD2	54857	4.86E-05	0.589186706
4150	TMEM103	54859	0.001559717	0.482921102
4151	MS4A12	54860	3.91E-05	0.594640606
4152	SNRK	54861	0.001193764	-0.492729648
4153	FNBP1L	54874	0.000685223	-0.512271178
4154	DPP8	54878	0.006303943	-0.426374561
4155	BCOR	54880	0.001279369	-0.490242244
4156	CCDC49	54883	0.009275447	0.408394535
4157	RP11-35N6.1	54886	3.39E-10	0.793389876
4158	ALKBH5	54890	1.36E-06	-0.667369252
4159	RNF43	54894	0.005127686	-0.435405264
4160	PQLC2	54896	2.60E-11	0.830658183
4161	CASZ1	54897	0.001328391	-0.488927886
4162	CDKAL1	54901	3.35E-05	0.59850618
4163	TTC19	54902	0.001753882	-0.478493391
4164	C10orf18	54906	0.003304592	-0.453873663
4165	KIAA1797	54914	0.007927287	-0.415943677
4166	YTHDF1	54915	8.11E-05	-0.576001935
4167	C14orf101	54916	0.001777468	-0.477978968
4168	CMTM6	54918	0.00391474	-0.446787592
4169	HEATR2	54919	2.43E-05	-0.606255296
4170	RASIP1	54922	1.99E-07	0.701739059
4171	ZNF434	54925	2.90E-08	0.732236013
4172	TMEM161A	54929	0.001718305	-0.479303482
4173	C12orf41	54934	9.61E-06	-0.627545191
4174	ADPRHL2	54936	6.70E-05	-0.580966556
4175	SARS2	54938	2.07E-09	0.769387697
4176	OCIAD1	54940	1.00E-05	-0.626643613
4177	MRPL16	54948	0.000168791	-0.555846743
4178	C1orf27	54953	0.004450646	-0.441389727
4179	FAM120C	54954	0.00829655	-0.41378112

4180	C1orf109	54955	0.003615833	-0.450172299
4181	TMEM160	54958	2.04E-07	0.701357381
4182	GEMIN8	54960	0.008751038	-0.41119713
4183	SSH3	54961	0.007536625	-0.418269262
4184	UCKL1	54963	0.007511447	-0.418409175
4185	CXorf48	54967	0.000562107	0.518954811
4186	BANP	54971	5.03E-05	-0.588354628
4187	CPSF3L	54973	0.000111887	-0.567321357
4188	SLC25A38	54977	0.003176633	-0.455475603
4189	C2orf18	54978	4.69E-07	0.686884323
4190	C2orf42	54980	0.000254719	-0.543819468
4191	Unknown	54982	7.16E-09	0.752639179
4192	C1orf123	54987	0.000515946	0.521785614
4193	ACSM5	54988	6.14E-07	0.682152542
4194	ZNF770	54989	0.00033142	-0.535894735
4195	C1orf159	54991	2.31E-10	0.798424202
4196	C20orf11	54994	0.000180775	-0.553869801
4197	OXSM	54995	0.006602392	0.424360211
4198	AURKAIP1	54998	0.000233606	-0.546431019
4199	C11orf59	55004	0.000790603	-0.507358698
4200	FLJ20628	55006	0.002745951	-0.461269691
4201	PIH1D1	55011	0.000111782	-0.567387261
4202	CCDC109B	55013	0.000358796	-0.533358123
4203	1-Mar	55016	8.61E-07	0.675907205
4204	C17orf73	55018	0.000332638	0.535778602
4205	PHIP	55023	0.007572151	-0.418040547
4206	FBXO34	55030	0.003109311	-0.456404918
4207	NOL8	55035	0.003820153	-0.447868492
4208	PTCD3	55037	0.002168435	-0.470503358
4209	PLEKHB2	55041	3.89E-05	-0.594771959
4210	MRPL20	55052	0.000544986	-0.519977788
4211	FLJ10038	55056	0.000656119	-0.513754478
4212	AIM1L	55057	0.006520958	-0.424909952
4213	SUSD4	55061	1.25E-09	0.776141438
4214	ZCWPW1	55063	0.004150125	-0.444407197
4215	GPR172B	55065	4.29E-05	0.592348719
4216	PDPR	55066	0.007472969	0.418669978
4217	C7orf42	55069	0.000105028	-0.569117485
4218	DET1	55070	0.004748095	-0.438705083
4219	C9orf40	55071	9.56E-07	0.673980563
4220	RNF31	55072	2.72E-10	0.796155774
4221	LRRC37A4	55073	1.99E-06	0.660124202
4222	OXR1	55074	0.004883045	-0.437492657
4223	FEZF2	55079	0.000276539	0.54139169
4224	TAPBPL	55080	0.002500364	-0.465042044

4225	ARGLU1	55082	0.003201915	-0.455163323
4226	SLC38A4	55089	0.001550268	0.483125729
4227	SAMD4B	55095	0.000242959	0.54528512
4228	WDR70	55100	3.29E-11	0.826396107
4229	FLJ10241	55101	0.001963998	-0.474226986
4230	ATG2B	55102	0.002926407	-0.458776905
4231	BSDC1	55108	9.12E-06	-0.628685031
4232	XKR8	55113	0.001639179	-0.481072658
4233	TMEM39B	55116	0.008282184	-0.413854282
4234	SLC6A15	55117	0.00011694	0.566119032
4235	FANCL	55120	0.000818748	-0.506108621
4236	C6orf166	55122	0.000344884	-0.534634386
4237	PIWIL2	55124	1.53E-05	0.617123751
4238	ELP3	55140	4.61E-05	0.590522224
4239	LRRC8D	55144	0.005990501	-0.42856717
4240	PAPD1	55149	0.00140816	-0.486721941
4241	FLJ10490	55150	3.82E-10	0.791727329
4242	DALRD3	55152	0.003767834	-0.448457977
4243	DARS2	55157	1.82E-07	0.703305512
4244	MSL2L1	55167	1.16E-09	0.777150379
4245	KLHL11	55175	6.22E-09	0.75459655
4246	FAM82C	55177	1.87E-10	0.801316234
4247	RIF1	55183	0.002286533	-0.468472413
4248	SLC25A36	55186	0.001536456	-0.483493934
4249	NUDT11	55190	0.005052235	0.436044732
4250	DNAJC17	55192	0.005772776	-0.430262171
4251	P15RS	55197	0.003903608	-0.446920703
4252	Unknown	55199	0.009642722	-0.406570919
4253	MAP1S	55201	0.000816337	-0.506219217
4254	GOLPH3L	55204	0.00346136	-0.451966633
4255	ZNF532	55205	0.008309008	-0.413711926
4256	SBNO1	55206	0.00113097	0.494666611
4257	ARL8B	55207	0.004280451	-0.443075235
4258	DPPA4	55211	0.003172435	0.455526774
4259	TMLHE	55217	0.009614102	0.40671051
4260	EXDL2	55218	4.98E-09	0.757564781
4261	KLHDC8A	55220	0.001860505	0.476245158
4262	LRRC20	55222	1.69E-09	0.772139739
4263	TRIM62	55223	2.94E-07	0.695084688
4264	ETNK2	55224	1.68E-07	0.704595641
4265	NAT10	55226	0.006476066	-0.42523467
4266	FLJ10781	55228	0.004282714	-0.443040344
4267	CCDC87	55231	1.97E-06	0.660370337
4268	FLJ10815	55238	0.008929003	-0.410230692
4269	C1orf75	55248	5.88E-05	0.584289373

4270	ELP2	55250	0.00842626	-0.412981387
4271	PCMTD2	55251	0.000117286	-0.566027421
4272	TMEM39A	55254	0.001911319	-0.475224846
4273	THNSL2	55258	0.009856772	-0.40550554
4274	PSPC1	55269	0.000240488	-0.54557796
4275	IMP3	55272	0.004529735	-0.440673435
4276	PHF10	55274	0.006944882	-0.422076508
4277	VPS53	55275	1.71E-09	0.771927952
4278	QRSL1	55278	6.33E-06	0.636654475
4279	CWF19L1	55280	0.000681543	0.512451298
4280	ACOXL	55289	5.20E-11	0.81994853
4281	UEVLD	55293	0.009773614	0.405902259
4282	RNF121	55298	0.001354545	-0.48823869
4283	GIMAP4	55303	0.00122958	0.491690131
4284	SLC29A3	55315	6.05E-07	0.682417206
4285	RSAD1	55316	1.14E-06	0.670779376
4286	C20orf46	55321	1.16E-05	0.623398536
4287	ABCF3	55324	0.00848814	-0.412621968
4288	LIN7C	55327	0.005999881	-0.42848232
4289	DRAM	55332	4.21E-06	0.645132073
4290	SYNJ2BP	55333	0.003330843	-0.453536926
4291	GIMAP5	55340	2.36E-08	0.735433167
4292	STRBP	55342	0.002229484	-0.469468416
4293	TCP11L1	55346	0.001035935	0.497771065
4294	ABHD10	55347	8.85E-10	0.780983891
4295	VNN3	55350	1.14E-10	0.808001704
4296	TMEM63B	55362	1.42E-09	0.774504497
4297	IMPACT	55364	0.007358482	-0.419370287
4298	LGR4	55366	0.001923539	-0.474994958
4299	PPP4R1L	55370	1.43E-09	0.77436218
4300	TMCO6	55374	0.00169625	-0.4798023
4301	MEG3	55384	4.25E-06	0.644903959
4302	PRO2214	55387	1.33E-07	0.708473302
4303	PRO2266	55389	4.41E-11	0.821950849
4304	CYorf14	55410	6.57E-11	0.815428141
4305	PRO2964	55415	5.07E-10	0.788031283
4306	C17orf85	55421	0.007137927	-0.420853295
4307	SIRPG	55423	2.50E-08	0.734501175
4308	ALS2CR2	55437	0.007900701	-0.416090141
4309	C14orf167	55449	0.003292861	-0.454013483
4310	PARL	55486	0.004168824	-0.444224968
4311	H2AFY2	55506	2.99E-06	0.652071089
4312	GPRC5D	55507	1.32E-05	0.62037925
4313	SAGE1	55511	5.12E-06	0.641079077
4314	SVOP	55530	0.000180593	0.553901759

4315	SLC30A10	55532	1.44E-06	0.666297161
4316	CDCA7L	55536	0.003343486	-0.453380308
4317	KCNQ1DN	55539	2.27E-06	0.657539028
4318	IL17RB	55540	2.20E-07	0.700042983
4319	RBM38	55544	0.001415685	-0.486492555
4320	ENOSF1	55556	0.007039372	-0.421459844
4321	PLXNA3	55558	0.001834109	0.476802882
4322	CDC42BPG	55561	2.59E-11	0.830804988
4323	DNAH3	55567	5.60E-10	0.786734725
4324	FOXRED1	55572	0.000720718	-0.510501964
4325	CDV3	55573	0.003374309	-0.453001343
4326	STAB2	55576	3.23E-10	0.794012047
4327	NAGK	55577	7.53E-08	0.717451171
4328	UBE2Q1	55585	0.001914058	-0.475168731
4329	VEZT	55591	0.005071864	-0.435870755
4330	ZCCHC8	55596	0.000742486	-0.509562892
4331	CDKN2AIP	55602	0.002942791	-0.458563832
4332	FAM46A	55603	0.000126455	0.563901617
4333	ANKRD10	55608	0.006492996	-0.425109546
4334	ZNF280C	55609	0.008112442	-0.414825282
4335	C20orf42	55612	9.69E-09	0.748368464
4336	TASP1	55617	0.001393354	-0.487121755
4337	TTC27	55622	0.006935655	0.422137806
4338	THUMPD1	55623	0.000743308	-0.509501249
4339	POMGNT1	55624	0.000204582	-0.550319122
4340	AMBRA1	55626	1.14E-05	-0.623775958
4341	ZNF407	55628	0.001458387	-0.485431712
4342	SLC39A4	55630	0.008373334	-0.413311321
4343	TBC1D22B	55633	1.44E-05	0.618443229
4344	DEPDC1	55635	2.99E-08	0.731745667
4345	BTBD2	55643	0.001309687	-0.489410437
4346	OSGEP	55644	0.008998692	-0.409850505
4347	PIGV	55650	0.001022665	-0.498242854
4348	NOLA2	55651	0.000543272	-0.520079207
4349	FLJ20489	55652	0.000404758	-0.529545253
4350	BCAS4	55653	6.46E-06	0.636192651
4351	INTS8	55656	0.000878816	-0.503659745
4352	PRPF40A	55660	0.002230963	-0.469433965
4353	DDX27	55661	0.000109567	-0.567960046
4354	HIF1AN	55662	0.000590244	-0.517369152
4355	NPLOC4	55666	0.000571486	-0.518393679
4356	SMEK1	55671	3.81E-05	0.595306306
4357	NBPF1	55672	9.00E-06	0.629000499
4358	IWS1	55677	9.75E-06	-0.627201912
4359	KIAA1310	55683	0.000874119	-0.503854462

4360	PACS1	55690	1.37E-05	0.619555843
4361	RBM22	55696	0.001150203	-0.494051699
4362	VAC14	55697	1.55E-06	0.664976922
4363	IARS2	55699	0.003349537	-0.453301193
4364	MAP7D1	55700	0.003329054	-0.453567694
4365	CCDC88A	55704	3.21E-07	0.693514346
4366	NECAP2	55707	5.25E-05	-0.587300428
4367	MLSTD1	55711	4.45E-09	0.759272719
4368	POLR3E	55718	0.000732413	-0.509981068
4369	TSR1	55720	0.006883252	-0.4224696
4370	C12orf11	55726	0.002794923	-0.460553985
4371	ATF7IP	55729	0.004258462	-0.443308
4372	C17orf63	55731	3.25E-05	0.599215548
4373	ZFP64	55734	0.002966597	0.458233296
4374	VPS35	55737	0.0057013	0.430779583
4375	ARFGAP1	55738	0.005632281	-0.431348741
4376	FLJ10769	55739	0.000347406	-0.53438175
4377	EDEM2	55741	0.000526816	-0.521076655
4378	NUP133	55746	0.003582357	-0.450561623
4379	CCAR1	55749	1.18E-05	-0.622924687
4380	CDK5RAP2	55755	8.36E-07	0.676473501
4381	RCOR3	55758	0.002392043	-0.466817015
4382	WDR12	55759	0.000103309	-0.569588391
4383	EXOC1	55763	0.000313559	-0.537591636
4384	NGLY1	55768	6.46E-05	-0.58190076
4385	PRR11	55771	0.008742655	-0.411245282
4386	TDP1	55775	2.73E-06	0.65394529
4387	C6orf70	55780	0.003421825	-0.452421938
4388	FGD6	55785	2.44E-06	0.656141258
4389	CXorf15	55787	0.008096045	-0.414935336
4390	ChGn	55790	0.000689906	0.512043594
4391	PCID2	55795	0.000112551	-0.567151427
4392	HR	55806	0.001691709	0.479899563
4393	SAC	55811	4.27E-10	0.790255215
4394	JMJD1A	55818	4.60E-05	-0.590586276
4395	VPS11	55823	0.000477967	-0.524284628
4396	IQWD1	55827	0.003871288	-0.447274467
4397	GLT8D1	55830	1.41E-05	-0.618885183
4398	CAND1	55832	0.000346813	-0.53444031
4399	UBAP2	55833	0.006590844	-0.424447624
4400	CENPN	55839	1.09E-09	0.777970494
4401	ARHGAP15	55843	3.07E-08	0.731317248
4402	C3orf10	55845	0.000905822	-0.502620287
4403	ITFG2	55846	0.00811112	-0.41483681
4404	CISD1	55847	0.001421378	-0.486354899

4405	USE1	55850	0.000983579	-0.49964148
4406	TEX2	55852	0.000334261	-0.535604237
4407	ZC3H15	55854	9.26E-05	-0.572483677
4408	C20orf19	55857	0.005439508	-0.432805153
4409	ACTR10	55860	0.001810808	-0.477287085
4410	ECHDC1	55862	0.003635309	-0.449945798
4411	SLC22A11	55867	1.66E-07	0.704765125
4412	ASH1L	55870	0.000907468	-0.50254943
4413	CBWD1	55871	0.00767149	-0.417431639
4414	GABRQ	55879	0.000185407	0.553166792
4415	ZNF167	55888	0.006678581	-0.423819527
4416	LENEP	55891	7.85E-08	0.716788517
4417	ZNF395	55893	0.006991046	-0.421760342
4418	MESP1	55897	0.000331336	0.535915132
4419	UNC45A	55898	0.000338201	-0.535244255
4420	ZNF302	55900	2.42E-07	0.69835029
4421	ACSS2	55902	0.006822475	-0.422881394
4422	MLL5	55904	0.00243561	-0.466114148
4423	ZNF313	55905	0.000352251	-0.533926274
4424	KIAA1166	55906	8.87E-11	0.811451616
4425	LOC55908	55908	5.21E-07	0.68507071
4426	ERBB2IP	55914	0.008160937	-0.414525057
4427	LANCL2	55915	0.004742705	0.438751721
4428	RCC2	55920	0.006678581	-0.423819376
4429	C1orf183	55924	1.30E-11	0.847380766
4430	DMAP1	55929	0.000503936	-0.522528022
4431	MYO5C	55930	0.007398041	-0.419137142
4432	APOM	55937	0.004069239	0.445195951
4433	LIN37	55957	2.97E-06	0.652223389
4434	3-Sep	55964	1.98E-05	0.611188331
4435	NSFL1C	55968	0.001337375	-0.488671991
4436	RAG1AP1	55974	0.005091721	-0.435695615
4437	NXF5	55998	1.82E-06	0.661869271
4438	NXF3	56000	7.46E-08	0.717588631
4439	NXF2	56001	3.67E-07	0.691275964
4440	PDGFC	56034	0.000950499	-0.500823557
4441	UBFD1	56061	3.85E-05	-0.595091156
4442	KLHL4	56062	0.000135616	0.56199089
4443	C1orf91	56063	7.35E-08	0.717837329
4444	Unknown	56097	3.17E-07	0.693722277
4445	Unknown	56098	6.25E-11	0.816322986
4446	Unknown	56099	1.12E-05	0.624041201
4447	Unknown	56105	0.001123385	-0.494884674
4448	Unknown	56107	5.78E-07	0.683219386
4449	Unknown	56114	0.000119039	0.565593465

4450	PCDHB15	56121	2.44E-06	0.656081121
4451	PCDHB11	56125	2.21E-06	0.658076124
4452	PCDHB9	56127	2.05E-07	0.701202424
4453	PCDHB6	56130	9.96E-11	0.809829995
4454	PCDHB4	56131	2.41E-10	0.797724844
4455	PCDHB3	56132	0.004941995	0.436979919
4456	Unknown	56134	4.54E-09	0.758982344
4457	Unknown	56135	1.65E-06	0.663786459
4458	PCDHA6	56142	0.000248202	0.544631526
4459	Unknown	56143	0.007556499	-0.41813623
4460	Unknown	56145	6.59E-11	0.815247336
4461	Unknown	56146	2.06E-05	0.61013201
4462	TEX15	56154	0.001133176	0.494592057
4463	TEX13B	56156	7.93E-07	0.677443765
4464	TEX13A	56157	5.99E-08	0.721039957
4465	RNF17	56163	4.24E-06	0.644945686
4466	STK31	56164	3.02E-07	0.694555615
4467	FAM54B	56181	0.002075693	-0.472181465
4468	ZNF253	56242	1.55E-08	0.741687865
4469	BTNL2	56244	0.003514454	0.451363788
4470	MRAP	56246	5.51E-09	0.756239539
4471	RNF20	56254	0.000594394	-0.517126867
4472	GKN1	56287	0.009818197	-0.405688736
4473	IL1F9	56300	2.79E-10	0.795785465
4474	TRPV5	56302	1.86E-10	0.801452514
4475	Unknown	56311	0.001603931	0.481879179
4476	LTB4R2	56413	2.35E-09	0.767742947
4477	CTPS2	56474	0.000709144	-0.511106771
4478	EIF4ENIF1	56478	2.72E-05	-0.603506986
4479	TUBB4Q	56604	3.81E-05	0.595354451
4480	ERO1LB	56605	0.001457299	0.485472057
4481	DIABLO	56616	0.000495121	-0.523102699
4482	INPP5E	56623	7.43E-07	0.678603781
4483	BCCIP	56647	0.002987595	-0.457964009
4484	C18orf2	56651	1.85E-08	0.739163691
4485	OR2S2	56656	2.17E-08	0.736713131
4486	TRIM39	56658	1.06E-07	0.712054685
4487	KCNK13	56659	0.000651511	0.513993581
4488	PANX2	56666	0.007755547	0.416941098
4489	SUCNR1	56670	6.33E-06	0.636657373
4490	C11orf17	56672	7.36E-06	0.633426664
4491	ASCL3	56676	2.41E-08	0.735145396
4492	SAR1A	56681	0.000223941	-0.547682304
4493	C21orf59	56683	0.004539873	-0.440581919
4494	JPH1	56704	2.79E-11	0.828951004

4495	SLC2A4RG	56731	0.005373936	0.433317335
4496	BARHL1	56751	0.000427447	0.527807968
4497	FMN2	56776	2.12E-06	0.658908937
4498	ZC3HAV1	56829	0.002332886	-0.467762961
4499	SLAMF8	56833	1.86E-07	0.702848964
4500	Unknown	56848	0.003189841	-0.455310366
4501	GRIPAP1	56850	0.000576863	-0.518114276
4502	C15orf24	56851	0.001356364	-0.488168452
4503	RAD18	56852	2.48E-11	0.831519455
4504	UGCGL1	56886	0.000537905	-0.520396809
4505	KCMF1	56888	0.003731278	-0.448855938
4506	TM9SF3	56889	0.000807961	-0.506636097
4507	MDM1	56890	0.006265596	-0.42664682
4508	LGALS14	56891	3.42E-05	0.597986044
4509	UBQLN4	56893	0.009380799	-0.407862012
4510	AGPAT3	56894	0.00250261	-0.465006388
4511	ANKS1B	56899	1.52E-09	0.773569265
4512	C1orf119	56900	0.002476825	-0.465452716
4513	PNO1	56902	3.13E-07	0.693980913
4514	STARD7	56910	0.001021893	-0.4982715
4515	C11orf60	56912	0.004192395	-0.443991084
4516	DHX33	56919	0.001657415	-0.480662543
4517	SEMA3G	56920	2.14E-09	0.768942404
4518	MCCC1	56922	0.006492117	-0.42512097
4519	NMUR2	56923	7.42E-10	0.78322053
4520	PAK6	56924	1.45E-07	0.707094212
4521	NCLN	56926	0.002006048	0.473412424
4522	SPPL2B	56928	2.39E-07	0.698601791
4523	DUS3L	56931	6.25E-09	0.754502186
4524	CA10	56934	1.04E-07	0.712337892
4525	C14orf162	56936	4.58E-09	0.758793326
4526	MRPS22	56945	0.000167441	-0.556064826
4527	C2orf33	56947	0.000298808	-0.539037603
4528	C14orf124	56948	0.0004402	0.5268773
4529	C5orf15	56951	0.003076735	-0.456833127
4530	NIT2	56954	0.000765717	-0.508505577
4531	MEPE	56955	2.24E-10	0.798954589
4532	SHD	56961	4.10E-08	0.726982917
4533	WDR93	56964	3.26E-08	0.730435589
4534	PARP6	56965	0.009739325	-0.406094372
4535	CEACAM19	56971	3.79E-11	0.824158965
4536	PRDM11	56981	0.000361914	0.533024077
4537	KTELC1	56983	0.000522378	-0.521357798
4538	PSMG2	56984	0.000708472	-0.511152021
4539	BBX	56987	0.00363614	-0.449924171

4540	CDC42SE2	56990	0.000424414	-0.528053534
4541	TOMM22	56993	2.10E-07	0.700829618
4542	SLC12A9	56996	0.000202792	-0.550583264
4543	CABC1	56997	0.002477976	-0.465431643
4544	CTNNBIP1	56998	0.000344426	-0.534676532
4545	Unknown	57000	0.008932035	0.410212109
4546	C7orf36	57002	0.006062552	-0.428050061
4547	CCDC47	57003	0.001475692	-0.484963694
4548	CABP4	57010	1.14E-05	0.62382415
4549	AKR1B10	57016	3.50E-05	0.597444617
4550	SLC17A7	57030	0.000176776	0.554533929
4551	ANKMY2	57037	0.00062224	-0.51555694
4552	RARS2	57038	0.001179236	-0.493164413
4553	PLSCR3	57048	0.007257618	0.420036998
4554	UTP3	57050	0.001507649	-0.484178719
4555	CHRNA10	57053	5.56E-09	0.756119799
4556	DDX24	57062	0.00534583	-0.433558564
4557	C20orf32	57091	0.007889007	0.416158193
4558	C1orf128	57095	0.002124635	-0.471287206
4559	RPGRIP1	57096	1.50E-07	0.706413511
4560	PARP11	57097	3.90E-09	0.761075454
4561	AVEN	57099	0.007539911	-0.418231623
4562	CYSLTR2	57105	0.000332646	0.535771801
4563	PDSS2	57107	6.57E-08	0.719586425
4564	TRPC7	57113	0.000725146	0.510302419
4565	PGLYRP4	57115	2.95E-11	0.827779432
4566	Unknown	57116	4.56E-05	0.59083852
4567	INTS12	57117	0.003137581	-0.456016703
4568	GOPC	57120	0.002756974	-0.461117114
4569	NUP107	57122	0.000439499	-0.526932843
4570	PLXDC1	57125	1.49E-07	0.706609626
4571	CD177	57126	1.78E-10	0.801986413
4572	RHBG	57127	2.36E-06	0.65675854
4573	CHMP1B	57132	1.94E-10	0.800805085
4574	MAN1C1	57134	2.14E-05	0.609361946
4575	C20orf3	57136	0.00899292	-0.409886208
4576	ADCK1	57143	3.97E-05	0.594264914
4577	PAK7	57144	0.008639992	0.411774031
4578	SCYL3	57147	0.002069721	-0.472281772
4579	KIAA1219	57148	6.37E-09	0.754251033
4580	LYRM1	57149	0.003594914	-0.450392857
4581	TMEM63C	57156	0.009206028	-0.408755286
4582	PHTF2	57157	0.008420254	-0.413024832
4583	JPH2	57158	1.15E-09	0.777218109
4584	SALL4	57167	1.56E-10	0.803909513

4585	CAMK1G	57172	1.59E-09	0.77301542
4586	CORO1B	57175	0.001429194	-0.486165207
4587	VARS2	57176	7.22E-09	0.75253831
4588	ZMIZ1	57178	0.004693138	-0.439197396
4589	KIAA1191	57179	0.004465322	-0.441254921
4590	ACTR3B	57180	5.55E-05	-0.585900849
4591	SLC39A10	57181	0.005383799	-0.433233721
4592	Unknown	57190	0.000493108	-0.523253215
4593	VN1R1	57191	9.00E-05	0.573275759
4594	MCOLN1	57192	0.000914347	0.502261515
4595	ATP10A	57194	1.99E-07	0.701712403
4596	KIAA0495	57212	0.000918994	-0.502067168
4597	THAP11	57215	0.003372591	-0.453035205
4598	TTC7A	57217	2.33E-05	0.607231438
4599	KIAA1244	57221	3.70E-09	0.761797358
4600	ERGIC1	57222	0.005067031	-0.435911034
4601	SMEK2	57223	0.000921655	-0.50195288
4602	LOC57228	57228	2.04E-09	0.769582748
4603	SNX14	57231	3.49E-09	0.762569625
4604	ZNF630	57232	1.68E-09	0.772202443
4605	KIAA0509	57242	0.00063202	0.515007126
4606	KIAA0114	57291	0.000328919	-0.53613968
4607	KIR2DL5A	57292	0.001685219	0.480028661
4608	CSRP2BP	57325	1.84E-07	0.703076893
4609	RCN3	57333	2.98E-09	0.764669552
4610	ZNF287	57336	9.63E-08	0.713592475
4611	SENP7	57337	0.002652193	-0.462676814
4612	JPH3	57338	2.04E-10	0.800160092
4613	GJD2	57369	0.001785236	0.477815858
4614	AICDA	57379	0.005880495	0.429398005
4615	RHOJ	57381	6.90E-10	0.784105259
4616	NMRAL1	57407	0.0049599	-0.43680009
4617	AS3MT	57412	1.97E-09	0.770058798
4618	BIRC6	57448	6.69E-07	0.680581833
4619	KIAA1143	57456	0.000957606	-0.500547383
4620	GATAD2B	57459	9.99E-07	0.673151783
4621	KIAA1161	57462	0.000425875	0.527928196
4622	AMIGO1	57463	3.46E-05	0.597740863
4623	FAM40B	57464	1.35E-11	0.842276875
4624	SLC12A5	57468	9.70E-09	0.748340348
4625	LRRC47	57470	5.36E-05	-0.58675816
4626	CNOT6	57472	0.002181967	-0.470275574
4627	ZNF512B	57473	9.93E-05	-0.570629638
4628	SHROOM4	57477	0.000762528	0.508657567
4629	NLN	57486	0.009751025	-0.40602069

4630	FAM62B	57488	0.004236163	-0.443560175
4631	KIAA1239	57495	2.55E-07	0.697510753
4632	KIDINS220	57498	0.002269009	-0.468775192
4633	MTA3	57504	0.001639095	0.481083105
4634	INTS2	57508	0.004379424	-0.442067083
4635	GPR158	57512	7.58E-09	0.751837598
4636	CDGAP	57514	0.00694848	0.422035878
4637	STARD9	57519	5.97E-05	0.583924127
4638	KIAA1303	57521	1.08E-08	0.74683045
4639	KIAA1305	57523	0.009062645	-0.409492451
4640	Unknown	57524	1.07E-05	0.62509233
4641	KCTD16	57528	1.48E-11	0.841277494
4642	RGAG1	57529	6.36E-09	0.754270143
4643	NUFIP2	57532	0.002320211	-0.467950123
4644	TBC1D14	57533	0.001705877	-0.479586999
4645	MICAL3	57553	5.96E-08	0.721144347
4646	NLGN2	57555	1.68E-06	0.663388891
4647	SEMA6A	57556	6.75E-07	0.680388653
4648	USP35	57558	0.004267688	-0.443203753
4649	ARRDC3	57561	0.00094145	-0.501146188
4650	ARHGAP20	57569	0.004451202	0.441379933
4651	TRMT5	57570	0.004164268	-0.444280068
4652	DOCK6	57572	0.000852094	0.504759986
4653	KCNT1	57582	0.003483548	0.451711843
4654	TMEM181	57583	0.000260862	0.543125374
4655	ARHGAP21	57584	9.22E-05	-0.57260162
4656	CRAMP1L	57585	1.87E-08	0.738967166
4657	KIAA1432	57589	3.99E-09	0.760704142
4658	MKL1	57591	7.75E-09	0.751477915
4659	ZNF687	57592	0.000418261	0.52850611
4660	BEGAIN	57596	0.000157157	0.557878352
4661	BAHCC1	57597	3.16E-10	0.794313876
4662	PITPNM2	57605	2.60E-07	0.697150908
4663	SLAIN2	57606	9.21E-06	0.628465073
4664	KIAA1462	57608	0.001109313	0.49532817
4665	DIP2B	57609	0.007647134	-0.417580898
4666	RANBP10	57610	3.28E-06	0.650148655
4667	ISLR2	57611	0.00146307	0.485308022
4668	KIAA1468	57614	0.001039532	-0.497637091
4669	SHROOM3	57619	0.009606137	-0.40675393
4670	ZBTB2	57621	0.008299302	-0.413762959
4671	ZFAT1	57623	1.52E-05	0.617223263
4672	KIAA1486	57624	4.78E-08	0.724603224
4673	HCN3	57657	1.30E-11	0.843855675
4674	ZBTB4	57659	4.89E-05	-0.58904433

4675	KIAA1542	57661	0.001478939	-0.484882944
4676	KIAA1543	57662	0.00746133	-0.418745786
4677	PLEKHA4	57664	0.000192181	0.552120091
4678	RDH14	57665	0.002193958	-0.470077649
4679	KIAA1553	57673	0.006622274	-0.424225007
4680	CHD8	57680	0.005687194	-0.430892909
4681	CACHD1	57685	0.001460138	-0.485388208
4682	KIAA1576	57687	5.68E-05	0.585266987
4683	LRRC4C	57689	5.65E-09	0.75587032
4684	ZNF317	57693	0.000552136	0.519553637
4685	FANCM	57697	1.46E-08	0.742611561
4686	KIAA1598	57698	0.008832376	-0.410779703
4687	KIAA1600	57700	0.003358835	-0.453193831
4688	WDFY4	57705	1.59E-09	0.773008275
4689	KIAA1609	57707	5.34E-06	0.640216122
4690	SLC7A14	57709	5.57E-10	0.786805317
4691	ZNF529	57711	0.008918172	-0.410298505
4692	SEMA4G	57715	2.60E-09	0.766292423
4693	TMEM16H	57719	1.89E-10	0.80118539
4694	GPR107	57720	0.004099012	-0.444903612
4695	KIAA1627	57721	0.0001273	-0.563699229
4696	KIAA1632	57724	0.003621567	0.450105852
4697	NCOA5	57727	0.001275296	-0.490358448
4698	SPTBN4	57731	5.42E-11	0.818936215
4699	RBAK	57786	0.000691528	-0.511963483
4700	SF4	57794	0.001481268	-0.484821975
4701	FAM5B	57795	1.44E-06	0.666394906
4702	)KFZP761C171	57796	7.69E-06	0.632494629
4703	HAMP	57817	0.002663429	0.462522703
4704	G6PC2	57818	3.74E-09	0.761661267
4705	SLAMF7	57823	2.66E-05	0.604018855
4706	HMHB1	57824	5.28E-09	0.756807354
4707	C6orf47	57827	0.002132098	-0.471155016
4708	C19orf15	57828	5.94E-06	0.63796184
4709	ZP4	57829	1.65E-05	0.615403386
4710	CYP4F11	57834	6.11E-10	0.785591812
4711	CADM3	57863	3.64E-10	0.792446827
4712	Unknown	57876	1.54E-06	0.665076666
4713	PTBP2	58155	8.78E-07	0.675527763
4714	NEUROD4	58158	0.003421657	0.452429021
4715	CTDSP1	58190	0.00224139	-0.469242343
4716	SQRDL	58472	0.007639431	0.417625173
4717	MS4A7	58475	5.22E-05	0.587435325
4718	ENOPH1	58478	0.000318174	0.537131181
4719	NLRC4	58484	5.29E-06	0.640410861

4720	TRAPPC1	58485	0.009789114	-0.405833468
4721	ZBED5	58486	0.000126403	-0.563918016
4722	CREBZF	58487	0.000539393	-0.520306492
4723	FAM108C1	58489	1.39E-06	0.666994328
4724	JAM2	58494	2.18E-09	0.768650523
4725	OVOL2	58495	0.009976242	-0.40492803
4726	PRUNE	58497	0.009941556	-0.405084371
4727	PROL1	58503	4.42E-07	0.687935345
4728	ARHGAP22	58504	0.00455955	0.440414683
4729	MLL3	58508	0.000816572	-0.506204914
4730	PRODH2	58510	2.83E-08	0.732567188
4731	DLGAP3	58512	4.30E-06	0.644684699
4732	EPS15L1	58513	1.94E-07	0.702180613
4733	SELK	58515	0.000172058	-0.55528502
4734	DMRT3	58524	5.16E-07	0.685267442
4735	PRM3	58531	6.54E-10	0.784810323
4736	TMEM8	58986	1.46E-06	0.666127056
4737	IL21	59067	2.73E-06	0.653928254
4738	ICEBERG	59082	3.36E-08	0.729905759
4739	HIVEP3	59269	0.005817298	-0.429913299
4740	C21orf63	59271	6.90E-09	0.75314179
4741	ACE2	59272	0.007488109	0.418570983
4742	CACNG8	59283	1.57E-07	0.705706935
4743	CACNG6	59285	2.64E-10	0.796559072
4744	UBL5	59286	0.000748737	-0.509270042
4745	PRDM13	59336	4.09E-06	0.645705061
4746	HRH4	59340	6.45E-07	0.681232082
4747	SENP2	59343	8.36E-05	0.575210889
4748	ALOXE3	59344	6.63E-06	0.635658108
4749	KLHL12	59349	2.35E-05	-0.60700687
4750	FAM3A	60343	0.001375015	-0.487627769
4751	AVPI1	60370	8.96E-06	0.6291156
4752	TSKS	60385	1.69E-05	0.614821867
4753	SLC25A19	60386	0.000359476	-0.533270177
4754	EDA2R	60401	1.75E-09	0.771651385
4755	TGIF2	60436	0.003737814	-0.448783436
4756	BACH2	60468	0.008168373	0.414474654
4757	SLC5A7	60482	1.76E-08	0.739905928
4758	TRMT11	60487	0.001573058	-0.482577401
4759	MRPS35	60488	0.00376933	-0.4484386
4760	PPCDC	60490	0.002251749	-0.469061831
4761	NIF3L1	60491	4.76E-07	-0.686620556
4762	CCDC90B	60492	0.004685389	-0.439277544
4763	HPSE2	60495	2.74E-06	0.653873199
4764	AASDHPPT	60496	0.00049206	-0.52334893

4765	AGBL5	60509	0.000546017	-0.519918141
4766	C2orf43	60526	3.96E-08	0.727450575
4767	SPCS3	60559	0.000479736	-0.524139147
4768	MAK10	60560	0.003431395	-0.452308246
4769	RINT1	60561	0.00573911	-0.430512
4770	SCOC	60592	0.004415278	-0.441751832
4771	DHX35	60625	4.15E-06	0.645412378
4772	RIC8A	60626	0.00017849	-0.554250593
4773	GAS5	60674	0.003023156	-0.457498571
4774	PAPPA2	60676	3.17E-10	0.794277608
4775	EEFSEC	60678	0.006895771	-0.422389696
4776	BRUNOL5	60680	4.21E-10	0.790480406
4777	SMAP1	60682	2.96E-07	0.694931908
4778	FLJ12716	60684	0.000130261	-0.563073021
4779	C14orf93	60686	0.006882184	-0.422480732
4780	C6orf85	63027	6.44E-07	0.681257236
4781	ELA2A	63036	0.002533933	-0.464538633
4782	SRR	63826	0.000936438	0.501369856
4783	ABHD4	63874	0.003649703	0.44976387
4784	PKNOX2	63876	5.34E-11	0.819282218
4785	C10orf84	63877	1.77E-05	0.613772137
4786	RNF123	63891	3.92E-07	0.69011539
4787	C14orf133	63894	9.91E-08	0.713147279
4788	FAM38B	63895	0.008124781	0.414755507
4789	HEATR6	63897	0.003156282	-0.455753576
4790	NSUN3	63899	8.93E-10	0.780860613
4791	DUSP21	63904	2.67E-10	0.796396822
4792	MANBAL	63905	0.001364246	-0.487941885
4793	GPATCH3	63906	8.23E-08	0.716099993
4794	NAPB	63908	0.009047722	-0.409573289
4795	C20orf59	63910	0.001201483	0.492496462
4796	ELMO2	63916	5.87E-07	0.682962626
4797	GALNT11	63917	2.26E-05	-0.607977819
4798	LOC63920	63920	0.000653153	-0.513909192
4799	CIDEC	63924	1.59E-11	0.838582416
4800	ANKRD5	63926	0.001634119	0.48120496
4801	MRPS14	63931	0.009707453	-0.40624962
4802	C20orf67	63935	4.62E-06	0.643221264
4803	GPSM3	63940	0.002657107	0.462608675
4804	NEUROD6	63974	3.69E-10	0.792242718
4805	PRDM14	63978	3.68E-10	0.792276614
4806	PCGEM1	64002	0.000624605	0.515441257
4807	TSPYL2	64061	2.48E-10	0.797425158
4808	RBM26	64062	0.001739849	-0.478813395
4809	LHPP	64077	3.96E-11	0.823579762

4810	GOLPH3	64083	0.00222921	-0.469477958
4811	CLSTN2	64084	0.003156282	0.455751905
4812	GFRA4	64096	9.26E-05	0.572483917
4813	CRLF2	64109	8.84E-09	0.749633875
4814	NPVF	64111	5.02E-06	0.641488197
4815	MOAP1	64112	0.001889851	0.475636944
4816	DUS1L	64118	0.001552714	-0.483069989
4817	XYLT1	64131	8.65E-07	0.675812036
4818	ZFYVE20	64145	4.20E-05	-0.592878523
4819	C17orf75	64149	0.000970115	-0.500086903
4820	CARD9	64170	0.002597781	-0.463524851
4821	SPATA1	64173	1.68E-05	0.614949005
4822	DPEP2	64174	0.002757868	0.461079014
4823	LEPRE1	64175	1.36E-06	0.667411896
4824	FAM12B	64184	4.08E-07	0.689369062
4825	C14orf4	64207	0.005255175	-0.434326956
4826	MMS19	64210	0.000651488	-0.514003232
4827	DNAJC1	64215	0.008340434	-0.413537964
4828	SEMA4A	64218	9.12E-06	0.628688621
4829	PJA1	64219	0.000779777	-0.507829691
4830	TOR3A	64222	0.0006925	-0.511910864
4831	GBL	64223	0.009087006	-0.409370372
4832	HERPUD2	64224	1.24E-05	-0.6218613
4833	ARL6IP2	64225	4.74E-05	-0.589812124
4834	MS4A5	64232	0.000148343	0.559499028
4835	PAPD5	64282	0.006935655	-0.422141974
4836	NOC3L	64318	0.006154552	-0.427399632
4837	RNF25	64320	2.25E-10	0.798893292
4838	SOX17	64321	0.008889992	-0.410456106
4839	XPO4	64328	0.001508365	0.48415809
4840	ARHGAP9	64333	0.004819654	0.438070059
4841	HS1BP3	64342	0.000528432	0.520954201
4842	HIF3A	64344	2.95E-08	0.731948319
4843	NXN	64359	0.000686006	-0.512226246
4844	SIL1	64374	0.005541189	-0.432018387
4845	CDH24	64403	1.47E-07	0.706790965
4846	CDH22	64405	2.16E-08	0.736823229
4847	C5orf28	64417	0.001494606	-0.48450448
4848	TMEM168	64418	0.006674161	0.423855005
4849	SUSD1	64420	4.75E-06	0.642637422
4850	ATG3	64422	0.00107818	-0.496313212
4851	MRPS25	64432	0.000154656	-0.558326428
4852	C7orf4	64433	1.02E-10	0.809561768
4853	NOM1	64434	0.002489494	-0.465243065
4854	CPEB1	64506	2.48E-05	0.605748577

4855	NDST4	64579	0.001016508	0.498459335
4856	CLEC7A	64581	1.10E-05	0.624480275
4857	RBMY3AP	64593	4.03E-08	0.727197686
4858	PERQ1	64599	1.51E-10	0.804402854
4859	PLA2G2F	64600	2.23E-05	0.608360798
4860	VPS16	64601	0.001749379	-0.478592959
4861	HIAT1	64645	0.006141764	-0.427504078
4862	SPANXC	64663	0.000366059	0.53266598
4863	ANAPC1	64682	0.002448386	0.465900453
4864	GORASP1	64689	0.000713987	-0.510855398
4865	CTAGE1	64693	0.002167431	0.470524152
4866	COPS7B	64708	0.000440843	0.526821668
4867	NUCKS1	64710	0.003310534	-0.453805779
4868	HS3ST6	64711	0.009045521	-0.409594479
4869	UNKL	64718	0.000933373	-0.501479987
4870	WDR13	64743	0.000936683	-0.501356253
4871	METT11D1	64745	0.001391513	-0.487182646
4872	ACBD3	64746	0.00152402	-0.483785668
4873	MFSD1	64747	1.37E-05	0.61964805
4874	SMURF2	64750	7.85E-05	-0.576898297
4875	CCDC136	64753	1.30E-10	0.806231966
4876	C16orf58	64755	0.00025311	-0.544029635
4877	MOSC1	64757	0.007758978	-0.416918742
4878	TNS3	64759	0.009109144	-0.409242938
4879	PARP12	64761	0.003806315	-0.448039696
4880	CREB3L2	64764	0.00370084	-0.449187834
4881	S100PBP	64766	0.005307841	-0.433900953
4882	C6orf106	64771	2.32E-09	0.767876458
4883	RMND5B	64777	1.18E-06	0.670027994
4884	RBM15	64783	0.001637922	-0.481113871
4885	RABL5	64792	0.009654801	-0.406505419
4886	CCDC21	64793	0.007875313	0.416246535
4887	DDX31	64794	2.94E-07	0.695034182
4888	ELOVL1	64834	0.002458026	-0.465757673
4889	KLC2	64837	7.26E-09	0.752455134
4890	PORCN	64840	0.000111245	0.567512738
4891	ISL2	64843	3.10E-07	0.694117791
4892	7-Mar	64844	0.001249329	-0.491099678
4893	SPATA20	64847	0.002194031	-0.47007108
4894	C1orf80	64853	0.000549083	-0.519752994
4895	RFXDC2	64864	0.000950915	-0.500804629
4896	AGXT2	64902	3.76E-08	0.728230182
4897	BCL11B	64919	1.19E-07	0.710247133
4898	TTC23	64927	3.89E-07	0.690268815
4899	CENPH	64946	2.19E-07	0.700102234

4900	MRPS26	64949	0.000512042	-0.522024079
4901	Unknown	64951	3.02E-05	-0.601022492
4902	MRPS9	64965	0.004071617	-0.445169769
4903	MRPS6	64968	2.83E-05	-0.602584834
4904	MRPL38	64978	8.78E-08	0.715035007
4905	MRPL36	64979	0.004135084	-0.444547769
4906	MRPL34	64981	0.008948965	-0.410121627
4907	MRPL32	64983	1.96E-05	-0.611352084
4908	MRPL9	65005	0.001049717	-0.497277543
4909	MRPL1	65008	0.00528529	-0.434095003
4910	NDRG4	65009	0.000361914	0.533017919
4911	GPBP1	65056	0.000522378	-0.52135872
4912	PFTK2	65061	1.35E-11	0.842289499
4913	NBEAL1	65065	0.005245225	-0.434423154
4914	ALS2CR10	65072	0.00761608	0.417769765
4915	RTN4R	65078	0.001370414	0.487761051
4916	NOL6	65083	8.08E-05	0.576091888
4917	TMEM135	65084	0.003905164	-0.446901223
4918	UPF3A	65110	0.00028041	-0.540972897
4919	RSRC2	65117	6.13E-05	-0.583224346
4920	WNK1	65125	4.54E-08	0.725460508
4921	NADK	65220	0.007156028	-0.420735545
4922	SPATS2	65244	0.003066001	-0.456964416
4923	MPPE1	65258	0.000494146	0.523170574
4924	WNK3	65267	4.15E-11	0.822641082
4925	BRD9	65980	0.001672708	-0.480295739
4926	ZSCAN18	65982	0.00076144	-0.50871709
4927	AACS	65985	0.000364756	-0.532775485
4928	ZNF747	65988	3.77E-11	0.824257916
4929	C20orf116	65992	0.000160649	-0.557223686
4930	MGC3032	65998	0.000502422	-0.522630768
4931	LRRC61	65999	0.001133176	0.494589742
4932	CYP4F12	66002	1.23E-06	0.66938029
4933	SLC2A11	66035	8.00E-05	0.576376234
4934	BOLL	66037	0.002076773	0.472158852
4935	CRELD1	78987	0.00833845	-0.41355245
4936	MRP63	78988	1.48E-05	-0.617877866
4937	OTUB2	78990	3.56E-09	0.762297913
4938	PRR14	78994	2.78E-05	0.603037205
4939	C1orf135	79000	3.41E-10	0.793277917
4940	MIS12	79003	0.002051874	-0.472594029
4941	CUEDC2	79004	0.000337914	0.535273314
4942	DDX50	79009	0.001665792	-0.480478599
4943	CAMKV	79012	0.00645137	0.42539519
4944	DDA1	79016	3.95E-05	0.594433372

4945	C17orf39	79018	0.000123946	-0.564453248
4946	MGC5590	79024	0.002181476	0.470293972
4947	PRNPIP	79033	0.002650175	-0.462718886
4948	C7orf26	79034	0.000333042	-0.535721331
4949	C19orf50	79036	0.006975804	-0.421864769
4950	TSEN34	79042	0.00246426	-0.465667189
4951	SECISBP2	79048	0.005788873	-0.430137183
4952	NOC4L	79050	0.006786704	-0.423118545
4953	ALG8	79053	0.000199121	-0.551097863
4954	PRRG3	79057	9.11E-06	0.628716245
4955	ASPSCR1	79058	2.20E-05	-0.608652789
4956	MGC3196	79064	0.003847921	-0.447547826
4957	ATG9A	79065	0.00014773	-0.559628006
4958	Unknown	79066	3.81E-09	0.761374558
4959	FASTKD3	79072	0.002239775	-0.469282703
4960	C11orf48	79081	0.000798649	-0.507030249
4961	WDR77	79084	1.12E-07	0.711144842
4962	ZNF426	79088	0.005855806	0.429602579
4963	CHAC1	79094	1.97E-08	0.738240084
4964	TRIM48	79097	0.000748573	0.509288149
4965	MGC4473	79100	0.000382884	0.531302842
4966	DHX58	79132	0.001308557	-0.489443026
4967	C20orf7	79133	0.009188819	-0.408845149
4968	FAM134A	79137	1.91E-05	-0.612025743
4969	CHCHD7	79145	0.000499917	-0.522801421
4970	FKRP	79147	3.00E-08	0.731691194
4971	MMP28	79148	1.28E-06	0.668632015
4972	MGC4859	79150	4.37E-05	0.59192522
4973	C7orf23	79161	0.004712975	-0.438996454
4974	LILRP2	79166	5.86E-06	0.638261771
4975	Unknown	79168	1.30E-05	0.620727996
4976	RBM42	79171	0.007135591	-0.420871068
4977	CENPO	79172	2.93E-09	0.764876964
4978	CRELD2	79174	0.000869694	-0.504034837
4979	ZNF343	79175	5.46E-08	0.722538547
4980	FBXL15	79176	0.00025666	-0.543588461
4981	ZNF576	79177	0.001794872	-0.47761407
4982	TMEM43	79188	0.008197438	-0.414314212
4983	ZNF557	79230	5.29E-09	0.756775869
4984	MMEL1	79258	0.000995206	0.499224156
4985	WDR32	79269	7.09E-05	0.579512903
4986	OR51B4	79339	1.65E-08	0.74081304
4987	OR51B2	79345	6.43E-06	0.636311087
4988	ZXDC	79364	0.000228784	-0.54705022
4989	BHLHB3	79365	0.001714066	-0.479394094

4990	FCRL2	79368	0.005853615	0.429621818
4991	BCL2L14	79370	1.87E-10	0.801340334
4992	NOX5	79400	2.04E-08	0.737668494
4993	GLB1L	79411	5.28E-09	0.756837746
4994	WDR25	79446	0.004424596	-0.441645918
4995	ULBP3	79465	3.40E-06	0.649492586
4996	FAM65A	79567	0.000304467	0.538443087
4997	C2orf47	79568	9.40E-05	-0.57209279
4998	EPS8L3	79574	0.000318693	0.537081176
4999	ABHD8	79575	0.007135591	-0.420874948
5000	GPR172A	79581	0.007812909	-0.416607964
5001	CORO7	79585	4.62E-06	0.643246161
5002	Unknown	79587	0.003383442	-0.45288021
5003	MRPL24	79590	0.000555073	-0.519372969
5004	C1orf166	79594	0.000135429	0.562031446
5005	LRRIQ2	79598	2.41E-10	0.797709404
5006	ADIPOR2	79602	0.007580154	-0.417989265
5007	C14orf138	79609	0.004555962	-0.440448496
5008	CCNJL	79616	9.59E-10	0.779755306
5009	HMBOX1	79618	0.000231959	0.546653053
5010	C16orf33	79622	0.007996353	-0.415538105
5011	GALNT14	79623	4.68E-10	0.789024629
5012	TNFAIP8L2	79626	1.57E-09	0.77317894
5013	SH3TC2	79628	8.60E-06	0.630051195
5014	EFTUD1	79631	0.001990745	-0.473696338
5015	FAT4	79633	0.000398017	0.530092559
5016	TMEM53	79639	0.001469048	-0.485151125
5017	CHMP6	79643	5.71E-10	0.786481373
5018	PANK3	79646	0.000709213	-0.511093571
5019	MCPH1	79648	2.18E-06	0.658436284
5020	NARG2	79664	0.00106671	-0.496719422
5021	DHX40	79665	3.43E-05	-0.597916099
5022	C3orf52	79669	0.000288044	0.540159055
5023	FN3KRP	79672	0.00757297	-0.418031187
5024	FASTKD1	79675	0.001751588	-0.478541172
5025	ZDHHC14	79683	3.06E-10	0.794690775
5026	ZNF322A	79692	0.001763033	-0.478292194
5027	ZMAT4	79698	2.81E-08	0.732755358
5028	PRKRIP1	79706	0.008076232	-0.415046622
5029	GLT25D1	79709	0.000360713	0.533152278
5030	MORC4	79710	1.16E-06	0.670427105
5031	IPO4	79711	0.003166533	0.455618155
5032	GTDC1	79712	0.000205172	0.550222157
5033	CCDC51	79714	0.0059645	-0.428752198
5034	NPEPL1	79716	5.17E-05	-0.587633314

5035	TBL1XR1	79718	0.004754449	-0.438645811
5036	ANKRD55	79722	1.60E-10	0.803573259
5037	ZNF768	79724	0.004399172	-0.441888968
5038	LIN28	79727	5.18E-07	0.685190578
5039	KCTD17	79734	0.000308891	0.538037556
5040	BBS10	79738	0.00705693	-0.421343488
5041	Unknown	79741	5.38E-11	0.819138659
5042	CXorf36	79742	1.05E-10	0.809166836
5043	ZNF419	79744	3.07E-08	0.731352652
5044	LMAN1L	79748	0.002417658	0.466416716
5045	ZNF659	79750	0.000327568	0.53627918
5046	SLC25A22	79751	0.009024064	-0.409728428
5047	DHRS12	79758	8.43E-07	0.67631156
5048	ELMO3	79767	0.005497447	-0.432328243
5049	TXNDC15	79770	0.00704417	-0.421428511
5050	MCTP1	79772	0.000133214	0.562472736
5051	ZFHX4	79776	0.00939265	0.407786076
5052	LRRC31	79782	5.96E-07	0.682667086
5053	MYH14	79784	8.43E-07	0.676308903
5054	ZNF665	79788	0.001988601	-0.473759067
5055	FBXO31	79791	0.006387572	-0.425813431
5056	GSDMDC1	79792	0.007276307	-0.41992235
5057	C12orf49	79794	1.05E-07	0.712176484
5058	ZNF408	79797	0.000177507	0.554410148
5059	KIAA1822L	79802	1.01E-07	0.712785047
5060	HPS6	79803	0.007600706	-0.417868795
5061	GSTCD	79807	9.75E-05	0.57110518
5062	SLTM	79811	4.32E-07	-0.688378337
5063	AGMAT	79814	1.01E-07	0.712880462
5064	ARHGAP28	79822	0.009887456	0.405343521
5065	C2orf34	79823	6.11E-05	0.583340639
5066	CCDC48	79825	4.39E-09	0.759463424
5067	ASAM	79827	1.35E-11	0.842494813
5068	NAT11	79829	0.006870837	-0.42256464
5069	QSER1	79832	2.87E-05	-0.602213296
5070	SGK269	79834	0.001627043	-0.481366091
5071	ZBTB3	79842	9.74E-07	0.67362712
5072	FAM124B	79843	4.43E-08	0.725809676
5073	SNX22	79856	0.006639524	0.424103079
5074	FLJ13224	79857	1.45E-08	0.742726471
5075	NEK11	79858	7.73E-09	0.751537586
5076	TUBAL3	79861	0.000203196	0.55051948
5077	ZNF669	79862	0.003546	0.450980697
5078	C18orf22	79863	0.007330864	-0.419572141
5079	C11orf63	79864	0.003657963	0.449668194

5080	TREML2	79865	1.98E-05	-0.611142468
5081	TCTN2	79867	0.008055489	-0.41519098
5082	FLJ12529	79869	4.80E-05	-0.589471251
5083	RABEP2	79874	4.20E-06	0.645155742
5084	UBA5	79876	1.09E-07	0.711584471
5085	ZC3H14	79882	0.004646654	-0.439622535
5086	HDAC11	79885	0.009108408	-0.409251258
5087	AYTL2	79888	0.005666511	-0.43107177
5088	RIN3	79890	3.01E-06	0.651902778
5089	ZNF672	79894	1.36E-06	0.667443561
5090	ATP8B4	79895	0.000693518	0.511856348
5091	FLJ14213	79899	8.99E-08	0.714625969
5092	NUP85	79902	0.000777206	-0.507944578
5093	FLJ14154	79903	0.002438934	-0.466052031
5094	TMC7	79905	0.001067914	0.496650947
5095	MORN1	79906	2.92E-06	0.65256711
5096	BTNL8	79908	1.45E-10	0.805011102
5097	MAGIX	79917	4.88E-11	0.820787174
5098	SETD6	79918	0.000587849	-0.517515196
5099	C2orf54	79919	0.000214117	0.549009593
5100	TCEAL4	79921	0.001858432	-0.476297904
5101	NANOG	79923	1.02E-09	0.778924975
5102	ADM2	79924	0.002410992	-0.466525578
5103	GRRP1	79927	2.28E-06	0.65742297
5104	MAP6D1	79929	3.08E-10	0.794578786
5105	KIAA0319L	79932	0.00391213	-0.446821932
5106	SYNPO2L	79933	0.000550874	0.519638221
5107	CNTD2	79935	1.48E-11	0.840876041
5108	CNTNAP3	79937	4.17E-10	0.790662226
5109	SLC35E1	79939	0.003276948	-0.454219532
5110	ZNF696	79943	0.001987793	-0.473783364
5111	PDZD7	79955	9.99E-06	0.626686694
5112	DENND1C	79958	4.30E-09	0.759729319
5113	DENND2D	79961	0.002986382	-0.457983362
5114	Unknown	79963	1.22E-10	0.807098191
5115	C6orf134	79969	3.78E-08	0.728155894
5116	ZNF442	79973	6.19E-11	0.816531977
5117	CXorf34	79979	8.04E-06	0.631518194
5118	DNAJB14	79982	0.001345656	-0.488467467
5119	SVEP1	79987	0.000597673	0.516939504
5120	ANKRD53	79998	4.45E-07	0.687834705
5121	KIAA1772	80000	1.60E-10	0.80357228
5122	RBM35B	80004	0.008743394	0.411236821
5123	TMEM156	80008	4.70E-07	0.686835385
5124	PHC3	80012	0.000113187	-0.567000866

5125	C10orf97	80013	0.000659152	-0.513562125
5126	C12orf30	80018	2.71E-06	0.65405884
5127	UBTD1	80019	0.001504023	-0.484266284
5128	FOXRED2	80020	0.000333135	0.535707667
5129	SLC24A6	80024	2.20E-07	0.699993328
5130	PANK2	80025	1.39E-06	-0.666959993
5131	FBXL18	80028	6.11E-10	0.785594896
5132	FAM130A2	80034	8.17E-07	0.676911782
5133	TRPM3	80036	1.03E-06	0.672539338
5134	Unknown	80060	0.00537726	0.433289197
5135	C6orf208	80069	8.59E-08	0.715385904
5136	CCDC15	80071	2.82E-08	0.732654587
5137	Unknown	80078	8.45E-08	0.715647761
5138	Unknown	80093	0.00591211	0.429180547
5139	FAM128B	80097	0.000133977	-0.562312123
5140	Unknown	80108	1.92E-09	0.770387005
5141	ZNF614	80110	6.61E-10	0.784663646
5142	C3orf36	80111	0.00131181	0.489354244
5143	YSK4	80122	8.45E-10	0.781525698
5144	ZNF703	80139	2.10E-05	0.6097904
5145	PTGES2	80142	0.001428315	-0.486190208
5146	THOC7	80145	0.006972112	-0.421891021
5147	UXS1	80146	0.006617065	-0.424260149
5148	PQLC1	80148	0.002616336	-0.463251642
5149	CENPT	80152	0.00410981	-0.444798931
5150	EDC3	80153	5.04E-11	0.82028409
5151	CXYorf2	80161	3.05E-05	0.60074472
5152	FLJ22184	80164	9.40E-06	0.628048228
5153	MOGAT2	80168	1.20E-10	0.807329569
5154	C17orf68	80169	2.95E-11	0.827805579
5155	TMEM134	80194	1.83E-06	-0.661706687
5156	OPA3	80207	9.71E-06	0.62730827
5157	SPG11	80208	0.003260961	-0.454437856
5158	C13orf23	80209	0.00063282	-0.51495295
5159	CCDC92	80212	0.001164794	-0.493601116
5160	TM2D3	80213	0.001060719	-0.496908412
5161	PAAF1	80227	1.09E-09	0.77792262
5162	RUFY1	80230	0.000502321	-0.522642369
5163	WDR26	80232	0.005821253	0.429865854
5164	PIGZ	80235	2.38E-07	0.698664564
5165	KIAA1539	80256	6.81E-08	0.719002491
5166	C16orf70	80262	7.55E-11	0.813570903
5167	TRIM45	80263	2.46E-08	0.734749774
5168	ITPKC	80271	1.21E-06	0.669569743
5169	CDK5RAP3	80279	0.00806224	-0.415150482

5170	MTERFD3	80298	2.26E-07	0.699522423
5171	C2orf44	80304	8.78E-08	0.715033672
5172	FER1L4	80307	5.03E-06	0.64143193
5173	SKIP	80309	3.65E-08	0.728673164
5174	LRRC27	80313	0.000553308	0.519481443
5175	EPC1	80314	0.00013064	-0.56298844
5176	PPP1R2P9	80316	6.93E-05	0.580097333
5177	ZKSCAN3	80317	3.33E-10	0.793621753
5178	GKAP1	80318	0.002542747	-0.464378057
5179	CXXC4	80319	7.45E-07	0.678560966
5180	ULBP1	80329	1.42E-06	0.666618134
5181	DNAJC5	80331	1.92E-05	-0.611827752
5182	WDR82	80335	1.48E-06	-0.665849022
5183	BPIL1	80341	1.73E-05	0.614282889
5184	TRAF3IP3	80342	1.67E-08	0.740636769
5185	REEP4	80346	0.000632477	-0.514974483
5186	COASY	80347	0.007491313	-0.418544085
5187	TNKS2	80351	0.008157246	-0.414553063
5188	PDCD1LG2	80380	2.20E-05	0.608719026
5189	CD276	80381	0.000160649	0.5572174
5190	UBXD1	80700	0.006087998	-0.427876848
5191	ESX1	80712	0.000264432	0.542726373
5192	TTYH3	80727	0.000119643	0.565429911
5193	LY6G5C	80741	0.000173436	0.555063398
5194	PRR3	80742	6.92E-06	0.634740984
5195	TSEN2	80746	0.000429119	-0.527675633
5196	STARD5	80765	4.61E-07	0.687173966
5197	CYB5B	80777	0.000234164	0.546356244
5198	COL18A1	80781	0.009071109	-0.409445119
5199	ASXL3	80816	9.69E-09	0.748363795
5200	KIAA1712	80817	7.31E-05	-0.578720127
5201	ZNF436	80818	1.47E-05	0.618014477
5202	KIAA1706	80820	5.49E-08	0.722441624
5203	DDHD1	80821	0.002420338	0.466374137
5204	TAS1R1	80835	6.91E-07	0.679948807
5205	SH3BP5L	80851	8.13E-11	0.812545837
5206	HCG4P6	80868	1.44E-05	0.618472092
5207	ILKAP	80895	0.000651511	-0.513987971
5208	TMPRSS5	80975	0.000224011	0.547668054
5209	GJA9	81025	0.009714481	0.406212236
5210	TUBB1	81027	3.40E-09	0.762892117
5211	WNT5B	81029	0.001070869	0.496549525
5212	COLEC12	81035	5.80E-05	0.584707391
5213	CLPTM1L	81037	0.000522586	-0.521334616
5214	OR7E104P	81137	0.000262455	0.542949518

5215	OR51E2	81285	6.35E-05	0.58234262
5216	Unknown	81488	0.001067914	0.496656227
5217	PTDSS2	81490	0.001417951	-0.486436567
5218	CFHR5	81494	0.000163116	0.556789123
5219	ITFG1	81533	0.00163323	-0.481233494
5220	SLC38A1	81539	0.003280181	-0.454171947
5221	LRRC3	81543	0.007966593	0.415699134
5222	TDRD3	81550	0.000773939	0.508102508
5223	STMN4	81551	5.39E-07	0.684470433
5224	ECOP	81552	0.004328789	-0.442593219
5225	FAM49A	81553	0.00067947	0.512563335
5226	WBSCR16	81554	0.000111145	-0.56755373
5227	YIPF5	81555	0.000906432	-0.502589415
5228	LMAN2L	81562	0.001387524	-0.487298402
5229	NDEL1	81565	0.000218074	-0.548465994
5230	FAM130A1	81566	5.84E-06	0.63833503
5231	CLPB	81570	4.77E-10	0.788761734
5232	C9orf45	81571	1.20E-09	0.776668844
5233	GFOD2	81577	9.34E-10	0.780159695
5234	CDADC1	81602	0.002997626	-0.457816941
5235	TRIM8	81603	0.000103429	-0.569553839
5236	SNX27	81609	0.003641287	0.449861216
5237	TMEM163	81615	1.17E-06	0.670263504
5238	ACSBG2	81616	6.05E-11	0.817041008
5239	Unknown	81619	0.001157112	0.493844607
5240	CDT1	81620	4.26E-08	0.72638956
5241	C1orf25	81627	0.001940476	-0.474664777
5242	TSC22D4	81628	0.007937302	-0.415873797
5243	TSSK3	81629	1.58E-08	0.741455356
5244	MAP1LC3B	81631	3.61E-08	0.728875553
5245	C6orf62	81688	6.40E-05	-0.582153238
5246	ISCA1	81689	0.002554415	-0.464207374
5247	OR5V1	81696	0.000657969	0.513643536
5248	OR2B2	81697	3.27E-05	0.59907159
5249	DOCK8	81704	5.25E-05	0.587305332
5250	TLR10	81793	3.98E-05	0.594237361
5251	OR12D3	81797	4.36E-10	0.789962381
5252	NETO1	81832	3.85E-05	0.595037978
5253	RNF146	81847	0.00599227	-0.428550806
5254	ZNF611	81856	0.0031781	-0.455448851
5255	KRTAP2-1	81872	9.01E-05	0.573229346
5256	RAB1B	81876	7.89E-06	0.631953552
5257	LAS1L	81887	0.003248175	-0.454590425
5258	HYI	81888	0.000808625	-0.506604791
5259	C14orf156	81892	0.000350256	-0.534103939

5260	FAM108A1	81926	0.006244107	-0.426786976
5261	HDHD3	81932	0.000115687	-0.566402335
5262	FCRL5	83416	1.76E-08	0.739884033
5263	FCRL4	83417	3.30E-10	0.793728265
5264	ADPGK	83440	0.008443816	-0.41288124
5265	ZNHIT4	83444	1.05E-10	0.808983417
5266	CCDC70	83446	0.004093139	0.444965483
5267	SLC25A31	83447	2.56E-07	0.69744497
5268	PUS7L	83448	4.19E-10	0.790572971
5269	PMFBP1	83449	2.98E-05	0.601332112
5270	MXD3	83463	3.37E-07	0.692699037
5271	ARHGAP24	83478	4.25E-10	0.790351411
5272	SCRT1	83482	3.85E-07	0.690460524
5273	DNAL1	83544	1.30E-11	0.843908019
5274	RTBDN	83546	1.13E-11	0.852850934
5275	RILP	83547	3.40E-06	0.649448526
5276	COG3	83548	0.001172092	-0.493363256
5277	UCK1	83549	0.007958858	-0.415752514
5278	GPR101	83550	8.79E-05	0.573880286
5279	TAAR8	83551	1.45E-08	0.742662286
5280	AKR1CL2	83592	1.30E-11	0.846387187
5281	Unknown	83595	0	1
5282	Unknown	83596	1.37E-05	0.619619114
5283	RTP3	83597	0.001495258	0.484485079
5284	Unknown	83598	0.000509193	0.522199499
5285	CCM2	83605	0.000559527	-0.519109695
5286	AMMECR1L	83607	0.000474406	-0.524518232
5287	TEX101	83639	8.92E-08	0.71478542
5288	FAM103A1	83640	1.87E-08	0.739030367
5289	RP3-402G11.5	83642	5.40E-07	0.684446979
5290	Unknown	83656	0.000118437	0.565749607
5291	SESN2	83667	2.35E-05	0.60710091
5292	CD99L2	83692	8.84E-10	0.781009965
5293	CALN1	83698	5.54E-11	0.818616385
5294	SH3BGRL2	83699	3.62E-07	0.691552798
5295	TRPT1	83707	0.002565595	-0.464027408
5296	YPEL3	83719	0.00071062	-0.51102269
5297	RIOK1	83732	0.007956973	-0.415766914
5298	ITCH	83737	0.000808775	0.506587713
5299	TFAP2D	83741	0.000320952	0.536878612
5300	L3MBTL2	83746	8.93E-10	0.780862564
5301	C20orf57	83747	5.43E-06	0.639856357
5302	Unknown	83752	0.001241208	-0.491347311
5303	KRTAP4-12	83755	4.53E-10	0.789436125
5304	FRMD8	83786	0.000207759	-0.549877299

5305	ARMC10	83787	0.003119344	-0.456250498
5306	KCNK16	83795	0.000218823	0.548364435
5307	USP26	83844	7.11E-06	0.634162247
5308	SYT15	83849	0.00371721	0.449000014
5309	FAM62C	83850	0.000402519	0.529722306
5310	SYT16	83851	0.002243879	0.46920002
5311	SETDB2	83852	0.009820222	-0.405671433
5312	ANGPTL6	83854	2.94E-05	0.6016203
5313	TTTY5	83863	1.78E-11	0.837013566
5314	TTTY11	83866	1.04E-09	0.778564272
5315	TTTY13	83868	4.00E-05	0.594089478
5316	RAB34	83871	0.000407189	-0.529355954
5317	MRO	83876	0.000192134	0.552135515
5318	SLC25A2	83884	3.59E-05	0.596737829
5319	PRSS27	83886	8.39E-08	0.715774275
5320	WDR87	83889	1.51E-07	0.706386817
5321	SPATA9	83890	5.04E-08	0.723764521
5322	SPATA16	83893	0.000866817	0.504162572
5323	KRTAP1-5	83895	4.46E-09	0.759216091
5324	KRTAP3-1	83896	0.000784074	0.507638449
5325	KRTAP3-2	83897	1.92E-09	0.770378021
5326	KRTAP9-3	83900	4.45E-07	0.687820367
5327	KRTAP17-1	83902	1.23E-07	0.709669291
5328	GSG2	83903	1.08E-07	0.711762099
5329	STK40	83931	2.75E-05	0.603220116
5330	EIF2A	83939	0.009400202	-0.40774376
5331	FCAMR	83953	1.05E-06	0.672327124
5332	FKSG83	83954	1.49E-10	0.8046122
5333	NACAP1	83955	4.68E-05	-0.590111527
5334	FKSG43	83957	1.24E-10	0.806858776
5335	SPNS1	83985	0.003888949	-0.447086262
5336	CCDC8	83987	0.006206795	-0.427039222
5337	CTTNBP2	83992	0.004048158	-0.445415381
5338	REG4	83998	0.009796599	0.405797649
5339	TMPRSS13	84000	5.54E-10	0.786878037
5340	GPR98	84059	7.49E-11	0.813690568
5341	KIRREL2	84063	9.84E-10	0.779338296
5342	HDHD2	84064	0.001573931	-0.482554137
5343	C1orf49	84066	2.35E-07	0.698922957
5344	C12orf25	84070	2.61E-11	0.830389913
5345	MYCBPAP	84073	2.59E-11	0.830773784
5346	QRICH2	84074	4.41E-11	0.821901473
5347	CCDC55	84081	0.003793032	-0.448190137
5348	USP44	84101	3.47E-05	0.597614184
5349	C4orf17	84103	4.87E-10	0.788523028

5350	Unknown	84105	0.005168137	-0.435063476
5351	PRAM1	84106	0.009877676	-0.405399927
5352	Unknown	84126	0.000553399	-0.519470805
5353	RUNDC2A	84127	0.002541916	0.464394522
5354	WDR75	84128	0.002153243	-0.470752297
5355	TMEM166	84141	0.002757868	0.461080453
5356	RNASEH2C	84153	0.005838565	-0.429749441
5357	KIAA1109	84162	5.64E-05	-0.585426958
5358	GTF2IRD2	84163	3.89E-06	0.646792801
5359	NLRC5	84166	6.31E-08	0.72021822
5360	RBED1	84173	1.48E-10	0.804739267
5361	SLA2	84174	3.42E-11	0.825890262
5362	TMEM164	84187	1.78E-09	0.771416579
5363	C12orf26	84190	8.19E-09	0.750682525
5364	FAM96A	84191	0.001198763	-0.492589137
5365	SETD3	84193	0.000340664	-0.535023224
5366	USP48	84196	0.004215279	-0.443783046
5367	FLJ23356	84197	1.57E-11	0.839348523
5368	TXNDC2	84203	1.16E-09	0.777064191
5369	MEX3B	84206	3.48E-05	0.597542672
5370	DKFZp434F142	84214	2.41E-06	0.656387356
5371	ZNF541	84215	2.62E-05	0.604426939
5372	TMEM117	84216	1.82E-07	0.703293125
5373	TMEM191A	84222	0.00815267	-0.414580182
5374	C2orf16	84226	3.55E-05	0.597047121
5375	LRRC8C	84230	1.15E-11	0.849687289
5376	TRAF7	84231	0.002117424	-0.471415638
5377	MAF1	84232	0.000413984	-0.528838552
5378	TMEM126A	84233	0.007928965	-0.415930265
5379	DKFZp547J222	84237	0.000118947	0.5656187
5380	ATP13A4	84239	7.84E-08	0.71683089
5381	LDOC1L	84247	0.00033142	-0.535902011
5382	SGIP1	84251	1.15E-08	0.74590662
5383	GARNL3	84253	0.002377117	0.467097455
5384	CAMKK1	84254	0.000299705	0.538951217
5385	FLYWCH1	84256	1.48E-11	0.841353654
5386	DCUN1D5	84259	0.00309394	-0.456613773
5387	PSMG3	84262	0.000810845	-0.506497163
5388	ALKBH7	84266	3.98E-06	0.646304649
5389	POLDIP3	84271	4.31E-07	0.688391711
5390	C4orf14	84273	0.00042435	-0.528063734
5391	NICN1	84276	0.000151914	0.558857886
5392	BTBD10	84280	0.003854951	-0.447467109
5393	RNF135	84282	0.00010467	-0.569238677
5394	TMEM79	84283	0.001045713	0.497411047

5395	C1orf57	84284	0.008906827	-0.410370242
5396	EIF1AD	84285	9.90E-05	-0.570722997
5397	ZDHHC16	84287	0.000403934	-0.529613481
5398	CAPNS2	84290	6.12E-11	0.81672554
5399	GINS4	84296	1.31E-07	0.708686579
5400	C6orf125	84300	0.009287133	-0.408323899
5401	NUDT22	84304	0.002560241	-0.464119659
5402	NUDT16L1	84309	0.000864137	-0.504271742
5403	C7orf50	84310	5.31E-05	-0.586962646
5404	MON1A	84315	0.005627109	0.431388187
5405	LSMD1	84316	0.000518216	-0.521638235
5406	ACBD6	84320	0.000399733	0.529955963
5407	C18orf12	84322	0.000183128	0.55350818
5408	Unknown	84324	0.001292936	-0.489879681
5409	HVCN1	84329	0.000536158	0.520495079
5410	ZNF414	84330	5.49E-05	0.58614681
5411	C16orf14	84331	0.003843997	-0.447594663
5412	AKT1S1	84335	5.56E-09	0.756104215
5413	ELOF1	84337	0.002618299	-0.463215395
5414	ZNF289	84364	0.005290391	-0.434043612
5415	GPR123	84435	2.54E-10	0.7971215
5416	ZNF528	84436	1.85E-05	0.612701529
5417	KIAA1822	84439	7.87E-10	0.782421565
5418	MAML2	84441	0.005326463	-0.433724256
5419	FRMPD3	84443	9.30E-07	0.674495878
5420	DOT1L	84444	3.96E-11	0.823676515
5421	SYVN1	84447	0.001088258	-0.495994568
5422	ABLIM2	84448	8.72E-09	0.749821463
5423	ZNF333	84449	8.75E-08	0.715110298
5424	PHYHIPL	84457	0.006742954	0.423406933
5425	LCOR	84458	0.001994833	-0.473618137
5426	KIAA1787	84461	8.32E-09	0.750485605
5427	BTBD12	84464	0.001456118	0.485508859
5428	MEGF11	84465	1.50E-05	0.617599265
5429	JPH4	84502	6.60E-09	0.753744314
5430	DCTN5	84516	2.78E-08	0.732906297
5431	ARPM1	84517	2.60E-05	0.604606648
5432	Unknown	84519	0.004268046	0.443191797
5433	JAGN1	84522	4.75E-05	-0.5897671
5434	C15orf41	84529	0.009386015	-0.407829605
5435	KIAA1853	84530	3.99E-10	0.79119803
5436	C21orf67	84536	0.003691019	0.449302954
5437	MRPL43	84545	0.001372157	-0.487704328
5438	COL25A1	84570	5.08E-05	0.588080363
5439	GNPTG	84572	0.000901784	-0.502806835

5440	PARD6B	84612	0.006165277	-0.427323586
5441	ZBTB37	84614	6.75E-08	0.719153118
5442	ZGPAT	84619	0.000507417	-0.522310024
5443	KRBA1	84626	8.61E-07	0.675908906
5444	KIAA1856	84629	0.00045143	0.526091726
5445	TTBK1	84630	2.56E-05	0.60499019
5446	LCE3D	84648	4.02E-05	0.59397213
5447	EBPL	84650	7.26E-05	-0.578894008
5448	SPZ1	84654	1.16E-07	0.710600685
5449	DPY30	84661	0.001509171	-0.484135564
5450	USP32	84669	0.004576881	-0.440240194
5451	TTTY6	84672	0.002089115	0.471925347
5452	SLC9A7	84679	1.94E-07	0.702202549
5453	HINT2	84681	8.70E-07	0.6757207
5454	INSM2	84684	2.24E-06	0.657822792
5455	MCEE	84693	0.004944042	-0.43695949
5456	GJA10	84694	5.56E-11	0.818475071
5457	Unknown	84695	4.26E-08	0.726368839
5458	ABHD1	84696	3.96E-11	0.823617058
5459	CREB3L3	84699	1.95E-09	0.770179698
5460	HDGF2	84717	0.004647598	-0.439609875
5461	PIGO	84720	5.99E-11	0.817385241
5462	KIAA0515	84726	0.002276472	-0.468638628
5463	CNDP1	84735	5.28E-05	0.587149137
5464	MGC10981	84740	2.06E-06	0.659479581
5465	FUT10	84750	1.73E-10	0.802251681
5466	MGC4655	84752	0.00214038	-0.471005058
5467	EFCAB4B	84766	2.40E-09	0.767460421
5468	ZNF607	84775	0.005685966	-0.430906201
5469	ARD1B	84779	6.35E-11	0.816119913
5470	MGC2889	84789	2.91E-05	0.601916817
5471	MGC12966	84792	0.008070581	-0.415084293
5472	MGC12982	84793	0.001081133	0.496212555
5473	C19orf48	84798	0.003667694	-0.449553199
5474	TA-NFKBH	84807	1.80E-07	0.703441897
5475	PLCD4	84812	4.13E-09	0.760239677
5476	PPAPDC3	84814	6.88E-07	0.680037615
5477	MGC12916	84815	0.000114978	0.566572006
5478	IL17RC	84818	6.84E-07	0.68013641
5479	FCRLA	84824	8.62E-11	0.811722511
5480	SFT2D3	84826	4.62E-05	0.590464459
5481	USMG5	84833	0.000361914	-0.533026343
5482	MGC15705	84843	0.000873429	0.503883806
5483	PHF5A	84844	0.009487732	-0.407319234
5484	MGC16075	84847	8.65E-06	0.629934489

5485	C9orf70	84850	5.63E-05	0.585514291
5486	MGC16291	84856	0.000961207	0.500428387
5487	MINA	84864	0.001935057	-0.474761621
5488	PTPN5	84867	3.55E-07	0.691856319
5489	CBR4	84869	0.000138708	-0.5613791
5490	AGBL4	84871	3.79E-09	0.76144274
5491	ZC3H10	84872	0.008497349	-0.412568561
5492	MFSD2	84879	0.000362528	0.532960825
5493	RPUSD4	84881	0.000715303	-0.510778146
5494	ZSCAN10	84891	3.24E-06	0.650446303
5495	C3orf39	84892	0.002973268	-0.458145351
5496	FBXO18	84893	1.86E-09	0.770848994
5497	TMTC4	84899	0.000109044	-0.568089316
5498	NFATC2IP	84901	0.00916935	-0.40893985
5499	CCDC123	84902	1.56E-08	0.741673695
5500	C9orf100	84904	3.75E-11	0.824552308
5501	ZNF341	84905	0.004268863	-0.443177959
5502	FAM136A	84908	2.86E-05	-0.602312039
5503	TMEM87B	84910	0.000569538	-0.51852085
5504	SLC35B4	84912	0.000316353	0.537322347
5505	FIZ1	84922	5.10E-08	0.723589492
5506	FAM104A	84923	0.004237526	-0.443542279
5507	C12orf52	84934	7.92E-08	0.716660554
5508	ZFYVE19	84936	0.00694848	-0.422040019
5509	CORO6	84940	3.49E-05	0.597521501
5510	HSH2D	84941	2.38E-10	0.797905906
5511	WDR73	84942	0.008130463	-0.414712702
5512	MAEL	84944	7.11E-06	0.634158791
5513	PRPF38A	84950	1.51E-07	0.706389122
5514	TNS4	84951	4.50E-10	0.78957904
5515	FBXL20	84961	0.000737644	-0.509766532
5516	IGSF21	84966	1.24E-08	0.744965346
5517	C1orf94	84970	3.71E-08	0.728454767
5518	SNHG7	84973	0.009531316	-0.407108019
5519	C12orf62	84987	0.00057972	-0.517955242
5520	RBM17	84991	5.15E-05	-0.587731079
5521	PIGY	84992	0.00019373	-0.551878743
5522	MGC16275	85001	0.004707585	0.439069764
5523	FAM86B1	85002	1.26E-09	0.775994946
5524	MGC2848	85003	0.00016705	0.556141085
5525	RERG	85004	0.008033733	0.415319847
5526	TMEM128	85013	0.000204344	-0.550355386
5527	REPS1	85021	0.001618544	-0.481555889
5528	TMEM60	85025	0.004660596	-0.439503135
5529	C1orf79	85028	0.00486863	-0.437637602

5530	KRTAP9-4	85280	5.57E-11	0.818411066
5531	KRTAP4-5	85289	1.06E-08	0.74707327
5532	KRTAP4-3	85290	5.94E-06	0.637952227
5533	KRTAP4-2	85291	1.46E-08	0.7425684
5534	ABCC11	85320	4.02E-06	0.6461085
5535	SYDE1	85360	9.07E-10	0.780614084
5536	TRIM5	85363	0.005613848	-0.431496991
5537	ALG2	85365	0.001188925	-0.492887469
5538	Unknown	85367	2.42E-05	0.606318822
5539	KIAA1654	85368	1.36E-05	0.619732579
5540	FAM40A	85369	5.36E-09	0.756606036
5541	KIAA1659	85373	1.51E-07	0.706331092
5542	CTA-221G9.4	85379	0.003171322	-0.455544298
5543	RGS8	85397	2.07E-10	0.799906746
5544	SLC22A16	85413	2.07E-10	0.799885588
5545	ZIC5	85416	0.004013436	0.445800252
5546	CCNB3	85417	0.008498291	0.412553944
5547	ZCRB1	85437	0.000986334	-0.499544228
5548	C4orf35	85438	0.003150183	0.455845756
5549	PRIC285	85441	0.003694075	-0.449263075
5550	CNTNAP4	85445	5.56E-08	0.722259964
5551	ZFHX2	85446	2.84E-05	0.602437998
5552	RP5-1054A22.3	85449	4.22E-08	0.726530372
5553	UNK	85451	0.000310306	0.537902574
5554	KIAA1751	85452	4.15E-06	0.645436506
5555	DISP2	85455	1.74E-09	0.77174712
5556	KIAA1737	85457	0.009519681	-0.407177309
5557	TANC1	85461	0.006378951	-0.42588021
5558	SSH2	85464	1.18E-07	0.710298511
5559	DNAJC5B	85479	2.16E-06	0.658558241
5560	GALP	85569	0.000223923	0.5476906
5561	ANKRD13A	88455	0.008568114	-0.412175009
5562	TRIM4	89122	2.88E-08	0.732309983
5563	SERPINB12	89777	0.001492806	0.484548242
5564	SERPINB11	89778	6.53E-06	0.635986267
5565	HPS4	89781	0.000399007	-0.530012095
5566	GAL3ST3	89792	2.95E-07	0.694989789
5567	PPP1R3F	89801	0.000119293	0.565534723
5568	ABCC10	89845	0.009769763	-0.405923184
5569	FAM125B	89853	2.93E-10	0.795195505
5570	SEC16B	89866	7.97E-05	0.576506451
5571	TRIM15	89870	6.83E-06	0.635027355
5572	OR6W1P	89883	0.002692224	0.462060339
5573	LHX4	89884	0.000242191	0.545383104
5574	FATE1	89885	8.45E-08	0.71564108

5575	SLAMF9	89886	1.40E-08	0.743282176
5576	WDR34	89891	0.003538973	-0.451066378
5577	TMEM116	89894	0.005772776	-0.430270394
5578	UBE3B	89910	0.00042745	-0.527801796
5579	RHOT2	89941	0.001133574	-0.494573281
5580	KIR3DX1	90011	2.41E-10	0.797723922
5581	LACRT	90070	1.86E-08	0.739112255
5582	LOC90113	90113	0.00018978	0.552494763
5583	TSR2	90121	0.004754449	-0.438649812
5584	BTBD6	90135	0.000471106	-0.524758306
5585	TSPAN18	90139	8.19E-11	0.812334447
5586	C14orf143	90141	3.31E-08	0.730176213
5587	FRMD7	90167	0.004418758	0.441707531
5588	EMILIN3	90187	0.001003425	0.4989278
5589	KIAA2013	90231	0.00297962	-0.458066644
5590	ZNF551	90233	2.46E-09	0.767133155
5591	C10orf75	90271	0.000409408	-0.52918175
5592	C3orf25	90288	0.001225341	0.491819778
5593	KLHL13	90293	9.00E-06	0.628979533
5594	CCDC97	90324	0.002388601	-0.46688159
5595	ZNF160	90338	0.00236498	-0.467295892
5596	LOC90379	90379	7.85E-09	0.751299893
5597	C15orf42	90381	6.59E-11	0.815278008
5598	IFT20	90410	0.003101252	-0.456505312
5599	MCFD2	90411	0.002125234	-0.471272423
5600	C15orf23	90417	1.19E-09	0.776813735
5601	BMF	90427	0.002635542	-0.462933533
5602	ZNF622	90441	0.001463188	-0.48529989
5603	GADD45GIP1	90480	0.003749228	-0.448665021
5604	LOC90499	90499	0.000228622	0.54708721
5605	C6orf142	90523	2.55E-11	0.831139639
5606	SHF	90525	4.90E-07	0.686116013
5607	DUOXA1	90527	3.74E-07	0.690982638
5608	C19orf52	90580	0.008549367	-0.412287012
5609	C21orf105	90625	2.14E-08	0.736967952
5610	C6orf176	90632	0.004026126	0.445666423
5611	COX19	90639	2.27E-06	0.657552666
5612	TGIF2LY	90655	9.00E-05	0.573248882
5613	C14orf121	90668	0.003518882	-0.451308924
5614	CCDC126	90693	0.000439499	0.526927621
5615	FAM104B	90736	0.000695659	-0.511760623
5616	MGC45800	90768	0.000188904	0.552625944
5617	ZNF479	90827	1.01E-07	0.712841988
5618	ZNF598	90850	2.71E-05	0.60363382
5619	HN1L	90861	0.00122358	-0.491875669

5620	C9orf123	90871	0.000215172	-0.548856815
5621	ESAM	90952	5.62E-11	0.818249048
5622	ADCK2	90956	6.05E-08	0.720886985
5623	ZNF251	90987	0.001239663	-0.491402905
5624	CREB3L1	90993	1.75E-07	0.703957503
5625	FMNL3	91010	0.000119552	0.565468793
5626	LASS5	91012	0.002684817	-0.462218923
5627	DPP9	91039	1.13E-09	0.77747537
5628	DKFZp761B107	91050	3.20E-05	0.599660807
5629	ANKRD30A	91074	0.001327432	0.4889561
5630	LOC91149	91149	2.52E-10	0.797203609
5631	NUP210L	91181	0.002684906	0.462212369
5632	SLC39A13	91252	0.005056065	-0.436011647
5633	FAM44B	91272	0.00028189	-0.540798131
5634	CDKN2AIPNL	91368	6.91E-06	0.634782371
5635	ANKRD40	91369	0.002284048	-0.468517138
5636	XRCC6BP1	91419	0.004989655	-0.436559868
5637	ACBD5	91452	0.003900924	-0.446955775
5638	ISX	91464	0.000688399	0.512119007
5639	UBXD5	91544	5.86E-08	0.721451378
5640	RPS19BP1	91582	0.00811112	-0.414840217
5641	CCDC16	91603	1.07E-08	0.746935144
5642	IDI2	91734	0.000898207	0.502948991
5643	YTHDC1	91746	2.13E-06	-0.658851445
5644	ZNF804A	91752	5.21E-10	0.787665238
5645	NEK9	91754	0.000864137	-0.504266536
5646	CABLES1	91768	2.61E-05	0.60457432
5647	FAM55C	91775	7.11E-11	0.814395867
5648	WDR20	91833	0.002855054	-0.459751601
5649	RFT1	91869	0.000295396	-0.539406183
5650	TIMD4	91937	1.29E-06	0.668447509
5651	LOC91948	91948	0.000109238	0.568039774
5652	COG7	91949	0.005254363	-0.434337914
5653	MYOZ3	91977	1.20E-09	0.776625513
5654	FAM58A	92002	0.002970833	-0.458178889
5655	MGC21675	92070	9.14E-11	0.810977445
5656	ZC3HAV1L	92092	0.000233702	-0.546414069
5657	OXNAD1	92106	5.42E-11	0.818964278
5658	MTDH	92140	0.000220102	-0.548188164
5659	ABBA-1	92154	2.50E-09	0.766826078
5660	MTG1	92170	1.83E-07	0.703193244
5661	UBTD2	92181	0.000266432	-0.542477743
5662	RCSD1	92241	7.85E-05	0.576902195
5663	ZNF461	92283	1.66E-06	0.663545831
5664	ZNF585B	92285	1.57E-10	0.803840932

5665	LYK5	92335	3.27E-08	0.730378168
5666	C1orf156	92342	0.009599156	-0.406788168
5667	LOC92345	92345	0.006841496	-0.422769831
5668	C1orf105	92346	2.60E-07	0.697175108
5669	LOC92482	92482	0.001267881	-0.490556853
5670	LDHAL6B	92483	3.27E-08	0.730350012
5671	ATXN3L	92552	6.37E-07	0.681477762
5672	G6PC3	92579	1.50E-07	0.706426237
5673	ASB16	92591	8.86E-06	0.629363784
5674	MOBKL1A	92597	0.002175522	-0.470389914
5675	TMEM169	92691	1.84E-10	0.801541924
5676	TMEM183A	92703	8.32E-11	0.812064708
5677	WDR85	92715	0.008701804	-0.411450964
5678	HELB	92797	0.002059661	0.472463998
5679	MGC16385	92806	7.66E-06	0.632587409
5680	HIST3H2A	92815	0.002005927	-0.473425369
5681	IMP4	92856	0.004876083	-0.437565892
5682	HNRPLL	92906	0.000470617	-0.524796751
5683	NT5C1B	93034	2.41E-11	0.832070631
5684	KCNG4	93107	4.80E-05	0.589503057
5685	TMEM44	93109	0.009174765	-0.40891123
5686	ORAI3	93129	0.006520208	-0.424919531
5687	OLFM2	93145	0.000471183	0.524745149
5688	HTR7P	93164	2.46E-08	0.734761987
5689	PRDM6	93166	5.47E-06	0.639720307
5690	PIGM	93183	2.91E-08	0.73217348
5691	IGSF8	93185	0.000986565	-0.49952689
5692	TMEM10	93377	2.68E-09	0.765943711
5693	MYLC2PL	93408	0.000521394	0.521429009
5694	ARMC6	93436	0.002235702	-0.469348596
5695	LOC93444	93444	2.18E-08	0.736646134
5696	C14orf32	93487	0.000757805	-0.508863691
5697	ANUBL1	93550	5.65E-06	0.639012118
5698	CACNA2D4	93589	0.002652169	0.462682557
5699	MRFAP1	93621	0.004820187	-0.438060752
5700	MGC16169	93627	1.65E-06	0.6637851
5701	MYOCD	93649	4.55E-07	0.687421739
5702	ACPT	93650	8.17E-06	0.631166267
5703	ST7OT1	93653	7.91E-09	0.751169086
5704	CAPZA3	93661	1.67E-05	-0.615141036
5705	HPYR1	93668	2.22E-07	0.699816726
5706	ACTR8	93973	0.000379962	-0.531525044
5707	ATPIF1	93974	0.001456744	-0.485489691
5708	CAMK2N2	94032	0.009911612	0.405216066
5709	ZNF101	94039	0.000302691	0.538618336

5710	SFXN1	94081	0.008999574	-0.4098415
5711	ORMDL3	94103	0.008346152	-0.413486101
5712	C21orf66	94104	0.005187165	-0.434921177
5713	MGC14327	94107	0.000327301	0.536319549
5714	ARHGAP12	94134	0.000437236	-0.527113184
5715	RP1L1	94137	6.68E-07	0.680592738
5716	ABCC12	94160	0.000207757	0.549883697
5717	FNIP1	96459	0.000811188	-0.506470469
5718	MRLC2	103910	0.000358455	-0.533390173
5719	EGLN3	112399	0.000392723	0.530509312
5720	GTF3C6	112495	0.00397377	-0.446201992
5721	CMTM7	112616	0.001377542	-0.487547691
5722	STX1B	112755	0.000375576	0.531885456
5723	TP53RK	112858	2.77E-10	0.795938263
5724	VPS26B	112936	0.001626025	-0.481391672
5725	Unknown	112937	2.26E-11	0.833213246
5726	PLCD3	113026	1.93E-07	0.702245463
5727	SAAL1	113174	2.38E-10	0.797953263
5728	CASC4	113201	0.00928476	-0.408341528
5729	LOC113230	113230	0.000152252	0.558795751
5730	SLC46A1	113235	0.004262849	0.443255107
5731	C12orf57	113246	0.000631228	-0.515060955
5732	LARP4	113251	0.008781479	-0.411049485
5733	TMEM106A	113277	0.001074758	0.496427396
5734	HEL308	113510	2.38E-07	0.698669332
5735	MGC16703	113691	5.83E-08	0.721532841
5736	ODF3	113746	1.12E-09	0.777670686
5737	PIK3IP1	113791	2.19E-06	0.65828354
5738	SLC35A4	113829	0.004950862	-0.436884274
5739	DTX2	113878	0.002515454	-0.46481234
5740	ZIM3	114026	0.001171326	0.493400389
5741	Unknown	114036	6.80E-05	0.580576798
5742	C21orf84	114038	2.79E-11	0.829020388
5743	C21orf88	114041	0.000433108	0.527412839
5744	C21orf89	114042	1.46E-06	0.666074102
5745	MCM3APAS	114044	0.000343759	0.534735199
5746	WBSCR22	114049	0.003582357	-0.450558213
5747	TXNRD3	114112	8.40E-09	0.750330775
5748	UCN3	114131	0.005200553	0.434791088
5749	SIGLEC11	114132	0.000908391	0.502501684
5750	SLC2A13	114134	1.48E-11	0.840732824
5751	Unknown	114224	5.40E-07	0.684412371
5752	NLRP3	114548	0.001563029	0.482820168
5753	SLC22A9	114571	7.61E-06	0.632755171
5754	TIRAP	114609	2.78E-11	0.829226257

5755	ERMAP	114625	0.001810808	-0.477283649
5756	CYGB	114757	1.21E-05	0.62246709
5757	COP1	114769	1.25E-10	0.806770264
5758	PGLYRP3	114771	4.71E-08	0.724852875
5759	BTBD9	114781	6.91E-08	0.718804517
5760	CSMD2	114784	0.000377988	0.531691986
5761	GPRIN1	114787	4.60E-09	0.758730369
5762	CSMD3	114788	0.008639992	0.411773587
5763	STK11IP	114790	0.00038423	-0.531182434
5764	FMNL2	114793	0.000969792	-0.500108393
5765	SLITRK1	114798	4.15E-11	0.822652396
5766	MYSM1	114803	0.004344269	-0.442431571
5767	RNF157	114804	1.64E-07	0.704981465
5768	GALNT13	114805	0.000427257	0.52783237
5769	KIAA1920	114817	7.00E-11	0.814592291
5770	SMYD4	114826	2.19E-09	0.768612109
5771	FHAD1	114827	5.89E-10	0.786023268
5772	SLAMF6	114836	2.33E-07	0.699049213
5773	OSBPL5	114879	5.28E-07	0.684860479
5774	OSBPL7	114881	1.21E-10	0.807200698
5775	OSBPL9	114883	2.18E-06	-0.658383747
5776	C1QTNF1	114897	0.000122935	0.564674318
5777	C1QTNF3	114899	0.002673686	0.462376113
5778	C1QTNF6	114904	1.86E-07	0.702920729
5779	C8orf40	114926	0.000619276	-0.515718067
5780	PTPMT1	114971	0.003594246	-0.450409643
5781	C6orf150	115004	0.008516381	-0.412459904
5782	CCDC124	115098	0.000915985	0.502188624
5783	LOC115110	115110	1.99E-07	0.701783527
5784	OMA1	115209	0.003683086	-0.44938418
5785	SLC25A26	115286	0.003117245	-0.456279141
5786	PCMTD1	115294	0.003447438	-0.452112991
5787	FCRL1	115350	1.74E-07	0.704093803
5788	FCRL3	115352	4.76E-08	0.724692567
5789	C7orf30	115416	3.75E-06	-0.647519864
5790	UHRF2	115426	0.005718206	-0.430658657
5791	GEFT	115557	6.96E-06	0.634600807
5792	TNFRSF13C	115650	2.30E-06	0.657297614
5793	KIR3DL3	115653	6.87E-10	0.784163715
5794	SNX26	115703	2.37E-05	0.606892698
5795	C14orf172	115708	1.37E-06	0.667298145
5796	RAB3C	115827	2.48E-09	0.766974572
5797	MGC20983	115948	0.000341294	0.534960909
5798	ZNF653	115950	0.009045521	-0.409596003
5799	C16orf75	116028	0.00058542	0.517645637

5800	LRRC58	116064	0.001066753	-0.496712507
5801	SLC22A12	116085	7.55E-11	0.813592802
5802	ZMYND19	116225	0.00469768	-0.439151659
5803	FAM36A	116228	0.001067801	-0.496665526
5804	MOGAT1	116255	0.005130426	0.435375088
5805	ACSM1	116285	3.76E-10	0.791956348
5806	PANX3	116337	2.20E-10	0.799176096
5807	SLC26A8	116369	7.24E-10	0.783512384
5808	LYPD1	116372	0.003581769	-0.450574689
5809	GRIN3A	116443	0.000489273	0.523526568
5810	TOP1MT	116447	0.005420044	-0.43295374
5811	MIST	116449	7.08E-08	0.718413015
5812	C1orf19	116461	5.86E-06	0.638281079
5813	MAS1L	116511	0.000114978	0.566573783
5814	MRPL54	116541	0.002385564	-0.466946028
5815	DYSFIP1	116729	2.13E-06	0.65886851
5816	CNTROB	116840	3.99E-07	0.689805922
5817	C1orf142	116841	0.003437968	-0.452219901
5818	MED12L	116931	7.71E-07	0.677956273
5819	CLRN1OS	116933	0.001059629	0.496960474
5820	ART5	116969	2.29E-10	0.798531217
5821	CENTG3	116988	0.000215178	-0.548844098
5822	CATSPER1	117144	0.000393019	0.530478163
5823	THEM4	117145	0.009030704	0.409694089
5824	SH2D1B	117157	0.007036604	0.421480129
5825	DCD	117159	2.36E-07	0.698814158
5826	WFIKKN1	117166	5.96E-08	0.721158743
5827	RAB3IP	117177	0.003251984	0.454544349
5828	SSX2IP	117178	0.001546962	0.483204815
5829	MRGPRX2	117194	2.55E-05	0.605059848
5830	HRASLS5	117245	0.004866932	0.437655626
5831	FTSJ3	117246	7.11E-06	0.634150057
5832	SLC16A10	117247	0.004775955	0.438433879
5833	DEFB118	117285	6.72E-08	0.719222477
5834	CIB3	117286	0.000115537	0.566440373
5835	TAGAP	117289	3.48E-10	0.793053846
5836	TMC1	117531	0.00183834	0.476713511
5837	PARD3B	117583	3.80E-10	0.791820822
5838	RFFL	117584	0.00684775	0.422708899
5839	ZNF354B	117608	1.04E-07	0.712431992
5840	Unknown	117852	3.82E-11	0.824052209
5841	C21orf100	118421	9.14E-09	0.749168507
5842	UBE2J2	118424	0.002430462	-0.466212397
5843	LOH12CR1	118426	2.75E-08	0.733035064
5844	GPR62	118442	1.14E-06	0.670764443

5845	C10orf90	118611	5.42E-11	0.818967381
5846	FAM24A	118670	0.001171998	0.493371394
5847	ZNF488	118738	3.06E-10	0.794705145
5848	MMP21	118856	0.000108032	0.568348395
5849	SFXN2	118980	0.001671209	-0.480341148
5850	C10orf104	119504	3.50E-05	-0.597364269
5851	OR5P2	120065	6.39E-09	0.754173889
5852	SLC36A4	120103	2.48E-09	0.766971985
5853	CASP12	120329	0.002479434	0.465401047
5854	Unknown	120400	0.003143507	0.455940678
5855	AMICA1	120425	4.76E-05	0.589680099
5856	C11orf46	120534	0.007539911	-0.418226824
5857	CCDC38	120935	4.62E-08	0.725177053
5858	C12orf59	120939	2.39E-05	0.606676114
5859	C12orf45	121053	1.30E-11	0.843450249
5860	TMEM132D	121256	0.009979638	0.404904861
5861	SLC15A4	121260	0.001474151	-0.485007924
5862	RHEBL1	121268	4.69E-07	0.686869909
5863	ZNF641	121274	0.002628414	0.46304691
5864	TPH2	121278	0.001443678	0.485807858
5865	AEBP2	121536	0.003588211	-0.450478392
5866	FOXN4	121643	3.39E-09	0.762930898
5867	C13orf16	121793	8.55E-07	0.676035721
5868	RXFP2	122042	0.000278302	0.541200897
5869	C13orf26	122046	7.01E-08	0.718576835
5870	C13orf28	122258	3.05E-05	0.600755682
5871	AK7	122481	8.16E-11	0.812451949
5872	C14orf79	122616	0.00214424	-0.470918148
5873	PLD4	122618	1.45E-08	0.742701535
5874	RNASE11	122651	0.000412723	0.528934896
5875	TPPP2	122664	0.000157169	0.557870254
5876	C14orf148	122945	0.000570044	0.518483615
5877	ACOT4	122970	2.98E-08	0.7318011
5878	SLC24A4	123041	0.00266784	0.462455761
5879	LRRC28	123355	1.21E-08	0.745223256
5880	AGBL1	123624	1.91E-06	0.660884998
5881	FSD2	123722	6.89E-06	0.634838625
5882	C16orf63	123811	2.31E-05	-0.607523301
5883	C16orf78	123970	6.96E-07	0.679788932
5884	CDYL2	124359	2.14E-07	0.700494465
5885	ANKS3	124401	0.009816363	-0.405701337
5886	FAM100A	124402	2.81E-07	0.695816365
5887	12-Sep	124404	8.83E-09	0.749655361
5888	ZNF720	124411	0.000290543	-0.539885591
5889	SLIC1	124460	4.02E-07	0.689652607

5890	LOC124512	124512	1.82E-05	-0.613124193
5891	HSF5	124535	3.90E-07	0.690209569
5892	OR4D2	124538	3.78E-06	0.647383674
5893	MSI2	124540	0.001561805	0.482861797
5894	MGC15523	124565	0.000104139	-0.569368618
5895	USH1G	124590	0.000598164	0.516907007
5896	ZPBP2	124626	0.00018503	0.553225242
5897	C17orf46	124783	1.13E-11	0.854725004
5898	CCDC43	124808	0.006297582	-0.426417934
5899	CNTD1	124817	1.43E-06	0.666510252
5900	WFIKKN2	124857	0.00067544	0.512748017
5901	B4GALNT2	124872	1.70E-06	0.663162515
5902	C17orf49	124944	0.005564099	-0.431859558
5903	C17orf57	124989	6.40E-07	0.681372293
5904	WDR81	124997	0.00469533	0.439175137
5905	TBC1D16	125058	0.00367154	-0.449510908
5906	GJD3	125111	1.30E-11	0.84527514
5907	KRT222P	125113	1.28E-09	0.77577684
5908	SMCR7	125170	0.005687377	-0.430886477
5909	C18orf51	125704	4.73E-09	0.758315975
5910	ZNF543	125919	8.71E-06	0.629763342
5911	RAVER1	125950	0.001770529	0.478133011
5912	COX6B2	125965	3.16E-06	0.650929411
5913	MBD3L2	125997	4.26E-08	0.72640379
5914	PCP2	126006	0.005362628	-0.433407064
5915	ZNF491	126069	0.00848814	0.412621529
5916	C19orf39	126074	0.000630393	-0.51511679
5917	C19orf41	126123	3.79E-09	0.761465536
5918	LOC126147	126147	9.58E-06	0.6276166
5919	NLRP8	126205	0.00181	0.477320154
5920	MOBKL2A	126308	1.49E-10	0.804568748
5921	C19orf28	126321	0.001334838	-0.488744918
5922	GIPC3	126326	1.35E-05	0.619889185
5923	NDUFA11	126328	0.00700223	-0.421688685
5924	LRRC25	126364	1.05E-10	0.809172838
5925	WTIP	126374	0.001776667	-0.478004574
5926	HSPB6	126393	7.41E-09	0.752146457
5927	CYP4F22	126410	0.005672202	0.431017501
5928	LOC126536	126536	0.002034269	0.47290459
5929	FAM148C	126567	3.23E-10	0.794012762
5930	RPTN	126638	2.37E-10	0.79806807
5931	SHE	126669	4.69E-09	0.758440327
5932	C1orf172	126695	0.009563222	-0.406948675
5933	C1orf96	126731	6.68E-09	0.753572776
5934	LRRC38	126755	0.001498294	0.484398469

5935	B3GALT6	126792	0.003476039	-0.451813161
5936	C1orf161	126868	0.007336777	-0.419522538
5937	LOC126917	126917	0.000169449	0.555729332
5938	ASB17	127247	2.21E-05	0.608510681
5939	DMBX1	127343	0.001014489	0.498532311
5940	TMCO2	127391	2.17E-08	0.736710374
5941	ZNF684	127396	1.42E-08	0.742986689
5942	C1orf83	127428	0.007323717	0.419635675
5943	GJB4	127534	8.86E-08	0.714902831
5944	DCST2	127579	8.27E-07	0.676673164
5945	TEDDM1	127670	1.31E-07	0.708698212
5946	C1orf122	127687	0.001393138	-0.487132226
5947	FLJ32784	127731	1.03E-09	0.778702492
5948	SYT2	127833	2.40E-08	0.735216397
5949	LOC127841	127841	2.00E-05	0.610927054
5950	UHMK1	127933	6.88E-05	-0.580291283
5951	WDR64	128025	0.000323	0.53668878
5952	C1orf131	128061	0.002537566	-0.464463123
5953	EDARADD	128178	2.05E-05	0.610361134
5954	KLF17	128209	1.61E-09	0.772768393
5955	TMEM125	128218	0.000271397	-0.541945279
5956	C1orf182	128229	2.30E-11	0.832679494
5957	IQGAP3	128239	1.30E-05	0.620756636
5958	APOA1BP	128240	0.000242191	-0.545374326
5959	MRPL55	128308	0.002741339	-0.461337701
5960	TMEM77	128338	3.97E-06	-0.646353244
5961	BHLHB4	128408	0.002774528	0.460830631
5962	WFDC12	128488	0.005922723	0.429082329
5963	C20orf174	128611	0.000361914	0.533020114
5964	Unknown	128646	6.76E-10	0.784356869
5965	PROKR2	128674	7.42E-08	0.717672629
5966	C20orf94	128710	0.002772233	-0.460880464
5967	CSTL1	128817	0.000137158	0.561678043
5968	C20orf166	128826	9.50E-06	0.62779779
5969	C20orf144	128864	3.49E-06	0.648936204
5970	GAB4	128954	3.08E-06	0.651469498
5971	LOC128977	128977	0.002490401	-0.46522229
5972	C22orf25	128989	0.000425514	-0.527963724
5973	ZNF280A	129025	0.000302674	0.538625942
5974	SGSM1	129049	0.000903605	0.502740625
5975	CCDC128	129285	0.006639524	-0.424099966
5976	TMEM150	129303	0.000479292	0.524171079
5977	NUP35	129401	0.002686607	-0.462158331
5978	MITD1	129531	0.001513695	-0.484023837
5979	MBOAT2	129642	2.04E-09	0.769601731

5980	CNTNAP5	129684	2.68E-08	0.733445591
5981	TAF8	129685	0.000247199	0.544751838
5982	ACMSD	130013	1.60E-05	0.616047863
5983	LOC130074	130074	4.09E-05	-0.593511981
5984	RFTN2	130132	2.40E-09	0.767454388
5985	LOC130355	130355	0.003732523	-0.448838818
5986	ACVR1C	130399	0.008398649	0.413151552
5987	TTC32	130502	0.002628414	-0.463050496
5988	UBR3	130507	0.003636433	-0.449913532
5989	KCTD18	130535	0.009257461	-0.408502559
5990	ALS2CR12	130540	5.86E-06	0.638248501
5991	ZNF513	130557	0.001882895	-0.475773509
5992	GALM	130589	0.003816448	-0.447924202
5993	CPO	130749	3.04E-09	0.76435902
5994	PQLC3	130814	0.003694138	-0.449257135
5995	LOC130951	130951	7.41E-08	0.717716752
5996	KCNH8	131096	6.57E-11	0.815372664
5997	FAM3D	131177	5.88E-10	0.78611512
5998	ZPLD1	131368	3.40E-06	0.649476461
5999	LYZL4	131375	0.000152576	0.558736011
6000	FAM131A	131408	0.004309288	-0.442786113
6001	CD200R1	131450	1.59E-06	0.664439119
6002	TMEM42	131616	1.86E-06	-0.661440727
6003	C3orf44	131831	1.81E-07	0.703352916
6004	METTL6	131965	0.004239132	0.443519103
6005	C3orf31	132001	1.35E-07	0.70814389
6006	C3orf49	132200	2.66E-06	0.654406715
6007	SYNPR	132204	1.52E-06	0.665350348
6008	SCLT1	132320	0.004017313	0.445755202
6009	TMEM155	132332	0.000686006	0.512228012
6010	LOC132430	132430	3.37E-11	0.826060807
6011	ADAD1	132612	2.28E-06	0.657489724
6012	NHEDC2	133308	0.00094145	0.501151812
6013	SLCO6A1	133482	0.004638593	0.439696158
6014	LOC133491	133491	1.44E-05	0.618458912
6015	FLJ40243	133558	3.96E-06	0.646391178
6016	EGFLAM	133584	1.82E-05	0.613183435
6017	UGT3A1	133688	4.64E-07	0.687087711
6018	LOC133874	133874	1.32E-05	0.62048714
6019	LOC134145	134145	0.005324417	-0.433743711
6020	CMBL	134147	0.0044628	-0.441280619
6021	POU5F2	134187	1.38E-09	0.77480315
6022	TMEM174	134288	2.98E-08	0.731812425
6023	LSM11	134353	0.000613269	0.516087177
6024	C5orf37	134359	4.72E-05	0.589919398

6025	ANKRD43	134548	2.01E-09	0.769807661
6026	SHROOM1	134549	8.99E-08	0.714639754
6027	C5orf24	134553	0.001055296	-0.49709886
6028	RLBP1L2	134829	1.23E-06	0.669309755
6029	TAAR9	134860	0.000534853	0.520576117
6030	TAAR1	134864	0.000204791	0.550286436
6031	C6orf141	135398	4.93E-08	0.724132865
6032	WBSCR28	135886	0.00249499	0.465139698
6033	TRIM50	135892	2.46E-09	0.767134148
6034	C7orf34	135927	8.53E-08	0.715484247
6035	NOBOX	135935	5.33E-06	0.64023165
6036	OR6B1	135946	1.02E-05	0.626255134
6037	EMID2	136227	1.22E-08	0.745129976
6038	LOC136242	136242	1.99E-06	0.660144862
6039	KLF14	136259	7.02E-10	0.783899048
6040	C7orf45	136263	0.007330394	0.419579792
6041	MTPN	136319	2.91E-05	0.601889739
6042	ASB10	136371	6.03E-08	0.720937541
6043	TRY1	136541	0.003532095	0.451145336
6044	C7orf11	136647	0.004880422	-0.437522842
6045	GOT1L1	137362	5.46E-09	0.756380611
6046	ABRA	137735	5.06E-05	0.588180223
6047	SGCZ	137868	0.000327978	0.536239284
6048	LOC137886	137886	0.006078891	-0.427938387
6049	WDR21C	138009	0.000176219	0.554626825
6050	RALYL	138046	2.23E-11	0.833681184
6051	C9orf41	138199	0.007217773	-0.420342373
6052	LCN8	138307	4.53E-06	0.643644317
6053	TAF1L	138474	2.80E-09	0.765409886
6054	ANKRD19	138649	3.18E-08	0.730828248
6055	C9orf23	138716	1.15E-06	0.670497604
6056	C9orf131	138724	2.97E-07	0.694887254
6057	LOC138948	138948	1.46E-09	0.774014981
6058	MAGEC3	139081	0.00929841	0.40826018
6059	CXorf20	139105	0.002647533	0.462759869
6060	FAM123B	139285	5.49E-05	0.586168048
6061	FUNDC1	139341	1.26E-05	-0.621565579
6062	VENTXP1	139538	7.86E-10	0.782467437
6063	FOXR2	139628	0.003222905	0.454893722
6064	GAB3	139716	6.71E-06	0.635416162
6065	PNCK	139728	2.02E-07	0.701471738
6066	SPIN4	139886	0.001063471	-0.496823628
6067	KRTAP13-1	140258	8.32E-08	0.715936537
6068	MUC17	140453	2.60E-09	0.766313314
6069	ASB6	140459	1.92E-06	0.660870401

6070	ASB8	140461	0.007875313	-0.416251383
6071	MYL6B	140465	0.000772296	-0.508181236
6072	RNF32	140545	4.69E-09	0.758466263
6073	APOBEC3D	140564	3.05E-07	0.69441595
6074	ZFP28	140612	1.51E-05	0.617414052
6075	C20orf96	140680	9.49E-06	0.627822738
6076	C20orf70	140683	6.53E-07	0.680999537
6077	ZBTB46	140685	0.000511259	-0.522073232
6078	CTCFL	140690	3.42E-06	0.649330868
6079	C20orf160	140706	7.92E-09	0.751154299
6080	BRI3BP	140707	1.41E-08	0.743102829
6081	C20orf118	140711	0.002812568	-0.460328516
6082	SPAG4L	140732	0.001591642	0.48215879
6083	NRSN1	140767	9.11E-05	0.572921955
6084	SMCR8	140775	0.000969617	-0.500125246
6085	TRPM6	140803	1.83E-05	0.612976496
6086	KRT72	140807	1.63E-05	0.615615362
6087	SRXN1	140809	0.00159282	-0.482129018
6088	Unknown	140828	0.005245981	0.434412581
6089	ZSWIM3	140831	4.08E-06	0.645759586
6090	C20orf62	140834	3.73E-11	0.824690695
6091	C20orf79	140856	0.000465291	0.525163218
6092	WFDC6	140870	2.87E-08	0.732392233
6093	C20orf173	140873	5.10E-05	0.58800089
6094	C20orf93	140875	3.75E-08	0.728271623
6095	DEFB129	140881	6.90E-07	0.679989633
6096	SFRS12	140890	0.001122863	0.494904614
6097	C20orf152	140894	7.93E-09	0.751123167
6098	STK35	140901	3.50E-05	-0.597429715
6099	C5orf20	140947	2.52E-05	0.605339471
6100	MIB2	142678	4.42E-05	-0.591594922
6101	SLC34A3	142680	3.33E-08	0.730090757
6102	RAB40A	142684	9.49E-10	0.779923753
6103	ASB15	142685	7.26E-09	0.752465352
6104	ASB14	142686	0.000133491	0.562414865
6105	ASB12	142689	2.18E-08	0.736595475
6106	LIPJ	142910	1.55E-08	0.741760311
6107	C10orf46	143384	0.001157462	-0.493829642
6108	PSMA8	143471	0.00093728	0.501314184
6109	C11orf40	143501	0.000311118	0.537824869
6110	OR51E1	143503	1.21E-10	0.807225455
6111	LOC143678	143678	1.27E-06	0.668714124
6112	FAM76B	143684	0.006914967	-0.422270397
6113	SESN3	143686	0.00128077	0.490202511
6114	TMEM86A	144110	2.66E-11	0.829943601

6115	OR10A5	144124	1.65E-06	0.663753616
6116	DNHD1	144132	0.000100607	0.570288242
6117	CPNE8	144402	3.62E-07	0.691530944
6118	GLT1D1	144423	0.003428813	0.452339919
6119	LOC144438	144438	0.000254029	-0.543921782
6120	A2ML1	144568	6.36E-08	0.720056302
6121	FBXL14	144699	0.000116937	0.566125892
6122	LOC144766	144766	0.00091958	0.502036557
6123	LOC144817	144817	3.00E-09	0.764541018
6124	LOC144920	144920	0.007254494	0.420083589
6125	RDH12	145226	2.31E-05	0.607516742
6126	GSC	145258	0.000738835	0.509713387
6127	SERPINA12	145264	1.17E-06	0.670306078
6128	ABHD12B	145447	1.25E-06	0.668982699
6129	C15orf43	145645	0.005917881	0.429128509
6130	LOC145678	145678	2.63E-07	0.696947927
6131	LOC145694	145694	1.89E-11	0.836056651
6132	LOC145757	145757	7.84E-08	0.716838756
6133	FAM81A	145773	1.93E-08	0.738532998
6134	LOC145783	145783	0.007254494	0.420094597
6135	LOC145820	145820	7.31E-11	0.814013095
6136	LOC145837	145837	4.73E-05	0.589866686
6137	TMCO5	145942	3.02E-10	0.794853418
6138	LOC145945	145945	0.001472948	0.48503934
6139	SPATA8	145946	8.43E-05	0.574995958
6140	NRG4	145957	0.000148081	0.559549619
6141	TMEM83	145978	0.000140539	0.561001715
6142	VWA3A	146177	0.001136836	0.494462289
6143	ZFP90	146198	0.008658779	-0.411672436
6144	BEAN	146227	5.11E-06	0.641118559
6145	LOC146325	146325	0.004318306	-0.442695009
6146	FLJ32252	146336	1.29E-09	0.775659103
6147	FLJ25404	146378	0.000290104	0.539932082
6148	LOC146429	146429	0.008598288	-0.41202094
6149	MGC45438	146556	8.02E-06	0.631590296
6150	C16orf71	146562	0.000949119	0.500873077
6151	HRNBP3	146713	5.88E-11	0.817689944
6152	CD300LF	146722	1.02E-09	0.778911955
6153	TCAM1	146771	2.55E-08	0.734207692
6154	LOC146795	146795	2.04E-05	0.610498479
6155	CCDC42	146849	1.95E-06	0.660580388
6156	C17orf38	146850	2.16E-08	0.736793786
6157	ODF4	146852	0.000617749	0.515826481
6158	AMAC1	146861	6.04E-05	0.583621082
6159	EME1	146956	4.22E-08	0.726546832

6160	TMC8	147138	0.003043136	0.457238524
6161	TRIM16L	147166	9.04E-05	0.573122967
6162	LRRC37B2	147172	6.35E-11	0.816040954
6163	TMEM99	147184	8.10E-08	0.716339659
6164	C18orf15	147276	0.000145322	0.560089885
6165	CBLN2	147381	1.78E-06	0.662276014
6166	DSG4	147409	1.15E-09	0.777276182
6167	ANKRD29	147463	0.008555853	0.412241736
6168	LOC147646	147646	8.82E-06	0.629445787
6169	Unknown	147694	3.74E-09	0.761633961
6170	KLC3	147700	5.44E-11	0.818830865
6171	LYPD4	147719	4.42E-07	0.687941112
6172	LOC147727	147727	0.009443226	-0.407525501
6173	HIPK4	147746	3.79E-09	0.761434007
6174	ZNF524	147807	2.28E-06	0.657400382
6175	ZNF563	147837	1.08E-09	0.778112827
6176	SPC24	147841	0.001728918	0.479049792
6177	FLJ32658	147872	1.08E-05	0.624862953
6178	SIX5	147912	0.003482559	-0.451742179
6179	IGFL2	147920	8.52E-09	0.750123434
6180	ZNF542	147947	9.92E-09	0.748033676
6181	ZNF582	147948	1.17E-06	0.670325565
6182	ZNF583	147949	4.97E-09	0.757623389
6183	CAPN12	147968	3.31E-08	0.730143295
6184	C19orf23	148046	0.003829154	0.447762951
6185	ZNRF4	148066	1.14E-06	0.670715956
6186	ZNF599	148103	0.000416979	0.528601438
6187	TMEM162	148109	1.64E-07	0.705076779
6188	C19orf55	148137	2.26E-11	0.832982582
6189	LOC148145	148145	0.001356364	0.488162907
6190	ZNF558	148156	0.001646804	-0.480901758
6191	FLJ25328	148231	1.76E-05	0.613895877
6192	C1orf58	148362	0.001002715	-0.498954992
6193	C1orf52	148423	0.000925988	-0.50179917
6194	PHF13	148479	1.26E-05	-0.621486047
6195	NBPF4	148545	0.000124423	0.564345883
6196	UBE2U	148581	0.000813738	0.50633692
6197	LOC148709	148709	2.54E-07	0.697542751
6198	C1orf76	148753	0.000413628	0.528868351
6199	MFSD4	148808	0.004574277	0.44026936
6200	PM20D1	148811	1.66E-09	0.772381286
6201	Unknown	148824	1.67E-06	0.663508523
6202	C1orf213	148898	8.04E-06	0.631531759
6203	KNCN	148930	5.53E-06	0.639463295
6204	GLIS1	148979	6.71E-10	0.784454886

6205	MGC27382	149047	7.11E-07	0.679387191
6206	ZNF362	149076	0.000929855	-0.501633719
6207	LOC149086	149086	3.27E-09	0.763399853
6208	DCST1	149095	3.28E-06	0.650155744
6209	IL23R	149233	1.66E-10	0.803007142
6210	FAM78B	149297	5.59E-05	0.585688743
6211	TMEM58	149345	0.001777468	-0.477980647
6212	FLJ30430	149373	8.19E-10	0.781902204
6213	WDR65	149465	2.72E-10	0.796192363
6214	C1orf210	149466	0.000936858	0.501333469
6215	CCDC24	149473	0.006944882	-0.422077128
6216	RNF187	149603	0.000301316	-0.538777012
6217	Unknown	149628	5.17E-09	0.757095363
6218	FAM71A	149647	2.61E-11	0.830486466
6219	LOC149684	149684	2.50E-10	0.797328098
6220	GTSF1L	149699	1.65E-07	0.704940068
6221	Unknown	149703	0.000225154	0.547516788
6222	LOC149773	149773	4.94E-08	0.72409831
6223	PRNT	149830	0.004171547	0.444196268
6224	LOC149837	149837	4.33E-05	0.592154047
6225	COMMD7	149951	0.000110688	-0.567673448
6226	C20orf186	149954	5.63E-08	0.722059234
6227	LIPI	149998	6.54E-11	0.815559831
6228	LOC150005	150005	2.16E-07	0.700336747
6229	LOC150051	150051	8.15E-11	0.81250751
6230	IGSF5	150084	3.55E-06	0.648595465
6231	C21orf129	150135	0.000146907	0.55979941
6232	C21orf121	150142	4.46E-07	0.687772198
6233	C21orf128	150147	2.01E-05	0.610776702
6234	NHEDC1	150159	0.000110105	0.567827624
6235	FLJ32575	150197	2.58E-08	0.734044232
6236	LOC150223	150223	0.000821216	-0.506001972
6237	C22orf15	150248	9.18E-05	0.572715729
6238	HORMAD2	150280	3.10E-09	0.764118832
6239	DUSP18	150290	1.61E-11	0.838217731
6240	C22orf27	150291	0.002589064	0.463654729
6241	RP1-127L4.6	150297	1.72E-07	0.704277815
6242	MEI1	150365	1.68E-10	0.80284509
6243	FAM109B	150368	1.27E-05	0.621316964
6244	NFAM1	150372	6.04E-09	0.75494328
6245	TTL	150465	0.000702303	-0.511435289
6246	CKAP2L	150468	1.03E-10	0.809462496
6247	FLJ32063	150538	4.23E-05	0.592744056
6248	LOC150568	150568	0.00041012	0.529127791
6249	SMYD1	150572	1.01E-08	0.747826148

6250	LOC150577	150577	0.008721184	0.41134765
6251	C2orf15	150590	0.000730994	-0.510049576
6252	MYEOV2	150678	8.18E-05	-0.575769662
6253	ALS2CR13	150864	0.00135515	-0.488219073
6254	FAM59B	150946	4.22E-07	0.688764479
6255	DKFZp434G179	150992	5.28E-07	0.684829764
6256	ZSWIM2	151112	3.69E-08	0.728534528
6257	LOC151146	151146	4.04E-11	0.823117616
6258	LOC151171	151171	1.33E-07	0.708498339
6259	FAM132B	151176	2.10E-11	0.834651013
6260	PPP1R1C	151242	1.69E-10	0.802758224
6261	ALS2CR11	151254	2.50E-09	0.766888145
6262	FLJ39822	151258	0.001543218	0.483320851
6263	CCDC140	151278	1.57E-11	0.839191246
6264	SLC23A3	151295	1.05E-10	0.808996021
6265	GPBAR1	151306	4.29E-08	0.726254884
6266	WDSUB1	151525	0.000200385	-0.550903705
6267	UPP2	151531	2.30E-09	0.768027036
6268	LOC151534	151534	6.35E-11	0.815885974
6269	DTX3L	151636	0.000434901	-0.527290611
6270	FAM19A4	151647	4.76E-05	0.589700771
6271	LOC151657	151657	1.01E-06	0.672962022
6272	LOC151760	151760	7.49E-07	0.678449472
6273	CPNE9	151835	0.006039118	0.428200887
6274	DPPA2	151871	0.000208734	0.549737483
6275	LOC151877	151877	1.63E-06	0.664001784
6276	BTLA	151888	0.000756454	0.508922013
6277	CCDC12	151903	0.000832436	-0.505546916
6278	PPP4R2	151987	3.08E-08	-0.731279312
6279	LOC152024	152024	1.07E-07	0.711844953
6280	FLJ31715	152048	1.25E-07	0.709492891
6281	C3orf22	152065	0.003528416	0.451197969
6282	LOC152118	152118	0.001236984	0.491475677
6283	CMTM8	152189	0.006537485	-0.424792982
6284	Unknown	152206	1.41E-06	0.666692013
6285	C3orf30	152405	0.005358365	0.433445693
6286	SH3D19	152503	0.000514746	0.521857394
6287	NPAL1	152519	2.07E-10	0.799943271
6288	LOC152578	152578	0.005446392	0.432747776
6289	SCFD2	152579	0.001801878	0.477476874
6290	Unknown	152586	6.13E-05	0.58325401
6291	C4orf38	152641	4.64E-08	0.725109481
6292	Unknown	152687	0.000111782	0.567377781
6293	C4orf39	152756	3.72E-10	0.792121587
6294	THAP6	152815	5.86E-09	0.755365711

6295	C4orf26	152816	6.02E-10	0.78579159
6296	FAM53A	152877	2.48E-08	0.734609224
6297	RASGEF1B	153020	3.39E-08	0.729784442
6298	DAB2IP	153090	0.005915112	-0.429151955
6299	SPINK5L3	153218	1.11E-05	0.624300638
6300	IRX2	153572	0.002491069	0.465205449
6301	ARSK	153642	0.000528432	0.520957724
6302	FAM71B	153745	0.000171101	0.555439248
6303	RNF145	153830	0.009921588	-0.405170461
6304	LOC153910	153910	0.000720469	0.510538459
6305	SAMD3	154075	9.11E-05	0.572920946
6306	RP4-662A9.2	154089	1.42E-10	0.805247673
6307	LOC154092	154092	0.004053032	0.445359682
6308	MBOAT1	154141	0.00686772	-0.422587189
6309	HDGFL1	154150	6.87E-10	0.784168499
6310	C6orf112	154442	0.000559587	0.519100698
6311	LOC154761	154761	8.30E-11	0.812108655
6312	Unknown	154791	3.08E-05	-0.600541795
6313	AMOT	154796	0.000813493	0.506357214
6314	VKORC1L1	154807	0.001007818	-0.498766788
6315	AMOTL1	154810	5.41E-05	0.586514795
6316	LOC154822	154822	2.48E-11	0.831540469
6317	LOC154872	154872	2.94E-05	0.601613483
6318	GIMAP8	155038	0.007847222	0.416406382
6319	ZNF746	155061	0.001743441	0.478724762
6320	ATP6V0E2	155066	0.008833647	-0.410768882
6321	AMZ1	155185	4.66E-05	0.590223036
6322	VPS37D	155382	2.09E-10	0.799779523
6323	RBM33	155435	0.006002764	-0.428459058
6324	MGC27345	157247	0.005373936	-0.433322318
6325	PEBP4	157310	2.91E-05	0.601857488
6326	CDCA2	157313	8.09E-09	0.750851438
6327	LOC157562	157562	0.002197314	0.470016059
6328	FAM87A	157693	2.88E-06	0.652878402
6329	Unknown	157740	0.00018132	0.553786654
6330	TMEM74	157753	2.77E-11	0.829348619
6331	C9orf62	157927	5.28E-11	0.819532634
6332	LOC157931	157931	6.02E-11	0.817148116
6333	C9orf66	157983	4.43E-10	0.789787759
6334	C9orf14	158035	1.11E-09	0.777796103
6335	C9orf163	158055	2.45E-08	0.73485445
6336	C9orf98	158067	2.62E-10	0.796696524
6337	OR1Q1	158131	0.000475307	0.52445311
6338	TTLL11	158135	1.88E-08	0.738896359
6339	HSD17B7P2	158160	1.37E-07	0.707900166

6340	LOC158257	158257	0.000196217	0.551500938
6341	C9orf138	158297	1.54E-09	0.77337214
6342	C9orf84	158401	0.000105028	0.569121114
6343	C9orf97	158427	2.57E-06	0.655111508
6344	ZNF645	158506	6.95E-07	0.679823041
6345	LOC158572	158572	8.31E-06	0.630795112
6346	ZXDB	158586	0.004048158	-0.445411903
6347	FLJ30672	158696	1.88E-07	0.702668987
6348	RP13-102H20.1	158763	0.001439271	0.485921137
6349	LOC158830	158830	1.95E-05	0.611549987
6350	DGAT2L3	158833	0.000315467	0.53740395
6351	FAM122C	159091	3.00E-09	0.764559279
6352	RBMY2FP	159162	5.56E-09	0.756096163
6353	TMEM20	159371	6.57E-10	0.784742299
6354	CCDC67	159989	0.000386879	0.530957573
6355	PATE	160065	0.000617749	0.515824547
6356	CLEC12A	160364	2.10E-07	0.700795722
6357	C12orf50	160419	8.74E-06	0.629684952
6358	IFLTD1	160492	1.71E-07	0.704375796
6359	CCDC60	160777	8.81E-06	0.629482298
6360	FAM71D	161142	3.55E-10	0.792771634
6361	C14orf49	161176	2.59E-09	0.766364887
6362	CLEC14A	161198	9.80E-10	0.779407401
6363	MDGA2	161357	1.92E-07	0.702384601
6364	C14orf174	161394	1.15E-09	0.777218584
6365	TBC1D21	161514	5.63E-10	0.786664697
6366	LOC161635	161635	0.003855851	0.447453504
6367	ODF3L1	161753	9.17E-10	0.780427126
6368	PGBD4	161779	0.001368579	0.48780872
6369	ADAL	161823	1.11E-07	0.711278325
6370	EXDL1	161829	0.000855905	0.504615457
6371	ZFPM1	161882	9.43E-10	0.780023512
6372	ADAD2	161931	1.18E-10	0.807633956
6373	LOC162073	162073	0.004112418	-0.444765629
6374	MGC34800	162137	7.07E-10	0.783809679
6375	ANKFN1	162282	3.62E-08	0.728819549
6376	SLFN5	162394	0.003854951	-0.447468487
6377	FAM134C	162427	0.006492117	-0.425119898
6378	TRPV3	162514	3.65E-09	0.762017396
6379	SLC16A11	162515	1.55E-10	0.804056221
6380	FBXO39	162517	0.000264958	0.542655326
6381	IMP5	162540	0.000591333	0.517304455
6382	FLJ16287	162962	3.75E-10	0.792014066
6383	ZNF610	162963	1.36E-05	0.619822737
6384	ZNF579	163033	0.003101252	-0.456509382

6385	ZNF383	163087	2.54E-06	0.655357837
6386	ZNF781	163115	5.56E-11	0.818489855
6387	C19orf46	163183	0.009649957	-0.406530648
6388	ZNF540	163255	1.70E-10	0.80251989
6389	GBP6	163351	5.80E-06	0.638493957
6390	PAP2D	163404	5.00E-05	0.588484858
6391	FNDC7	163479	1.51E-08	0.742129954
6392	TDRD5	163589	2.50E-10	0.797294562
6393	Unknown	163742	6.12E-11	0.81672991
6394	SPRR4	163778	1.25E-07	0.709403324
6395	C1orf55	163859	0.005699553	-0.43079625
6396	FAM43B	163933	2.00E-07	0.701681068
6397	C1orf65	164127	0.007804386	-0.41666077
6398	UBL4B	164153	0.00694511	0.422070053
6399	APCDD1L	164284	0.003839468	0.44764179
6400	WBP2NL	164684	3.43E-11	0.825810509
6401	GPR113	165082	2.11E-09	0.76913826
6402	OXER1	165140	1.70E-05	0.614690213
6403	CLEC4F	165530	0.000995206	0.499222783
6404	PARP15	165631	9.34E-10	0.780177784
6405	XIRP1	165904	1.08E-06	0.671764253
6406	DCLK2	166614	0.009561489	0.40696103
6407	GPR125	166647	0.000222834	-0.547829934
6408	ZNF509	166793	1.31E-10	0.806122272
6409	OLIG3	167826	1.35E-10	0.805820286
6410	RNF133	168433	3.75E-06	0.64755108
6411	THAP5	168451	0.007158153	-0.420713363
6412	GIMAP7	168537	0.000105066	0.569102441
6413	CNBD1	168975	2.26E-11	0.83317005
6414	SLC30A8	169026	5.56E-07	0.683931248
6415	C9orf96	169436	8.16E-11	0.812424368
6416	KCNV2	169522	1.49E-08	0.742295014
6417	C9orf71	169693	2.47E-05	0.605898974
6418	ZNF169	169841	9.50E-07	0.674107502
6419	SPIN3	169981	0.003421181	0.45244195
6420	MGC17403	170082	1.18E-10	0.80764684
6421	C10orf128	170371	4.88E-11	0.820822296
6422	FUT11	170384	1.02E-08	0.74767606
6423	OIT3	170392	0.000870209	0.504011415
6424	C10orf91	170393	0.000786078	0.50755146
6425	SSBP4	170463	0.001224683	-0.491842085
6426	CLEC4C	170482	6.40E-05	0.582164877
6427	HTR3C	170572	1.39E-05	0.619286641
6428	GIMAP1	170575	2.29E-08	0.735920663
6429	COMMD6	170622	0.000899769	-0.502885397

6430	PSORS1C2	170680	0.007254494	0.420079514
6431	NUDT10	170685	2.22E-06	0.658050276
6432	GSX2	170825	2.41E-06	0.656315354
6433	KCNG3	170850	3.14E-11	0.826871499
6434	ZNF721	170960	0.001461263	-0.485352682
6435	ANKRD24	170961	0.008355459	-0.413420996
6436	ZNF384	171017	8.96E-06	0.629097671
6437	ASXL1	171023	0.003588211	-0.450477987
6438	NS3BP	171391	4.70E-09	0.758417418
6439	CLYBL	171425	0.000161739	0.557034056
6440	PLAC4	191585	0.005198458	0.434818019
6441	PGAM5	192111	0.001484798	0.484735423
6442	HIGD2A	192286	0.00198926	-0.47373176
6443	EIF2C4	192670	0.004610277	-0.439944824
6444	RDHE2	195814	0.003521046	0.451282011
6445	IMMP1L	196294	0.003101004	-0.456518872
6446	KRT78	196374	7.73E-06	0.63238342
6447	DNAH10	196385	1.00E-05	0.626610522
6448	METTL7B	196410	6.58E-06	0.635817207
6449	CCDC131	196441	0.005800575	-0.430035532
6450	FAM71C	196472	5.78E-06	0.638557118
6451	C12orf12	196477	0.004559741	0.440407878
6452	C12orf53	196500	7.95E-05	0.576566525
6453	DCP1B	196513	0.00560324	-0.431572618
6454	ARID2	196528	0.005966705	-0.428727018
6455	LOC196541	196541	5.64E-08	0.722033856
6456	C10orf72	196740	0.000150344	0.559137673
6457	ADCY4	196883	1.32E-09	0.775455672
6458	LOC196913	196913	1.15E-11	0.849226233
6459	C15orf51	196968	0.002733455	0.461446481
6460	MGC15885	197003	4.84E-11	0.820936217
6461	UBR1	197131	0.006288174	-0.426484859
6462	Unknown	197187	0.002029749	0.472986379
6463	LDHD	197257	0.005295904	-0.433994636
6464	ZNF778	197320	1.30E-11	0.844483534
6465	ZNF553	197407	0.00607273	-0.427981751
6466	ZNF627	199692	0.009449501	-0.407493836
6467	GGN	199720	1.41E-06	0.666777308
6468	U2AF1L4	199746	2.05E-10	0.800123346
6469	ZNF626	199777	5.20E-11	0.819832874
6470	ALG14	199857	7.52E-10	0.783023739
6471	RP13-15M17.2	199953	0.000662311	0.513394155
6472	C1orf86	199990	0.006416265	-0.425633264
6473	CC2D1B	200014	8.36E-07	0.676475288
6474	C1orf100	200159	4.65E-09	0.75859295

6475	KRTCAP2	200185	0.000116947	-0.566111259
6476	CRTC2	200186	0.007235581	-0.420226746
6477	C1orf69	200205	0.008421081	-0.413015841
6478	LOC200261	200261	2.21E-05	0.608574369
6479	RNF215	200312	7.19E-05	0.579157283
6480	APOBEC3F	200316	6.56E-06	0.635897592
6481	CREG2	200407	0.00021282	0.549198213
6482	GKN2	200504	8.45E-10	0.781531107
6483	C2orf51	200523	3.32E-06	0.6499236
6484	PIP5K3	200576	0.00739786	-0.419143265
6485	LOC200609	200609	6.11E-10	0.785566584
6486	KRTCAP3	200634	0.002306224	-0.468173518
6487	LOC200830	200830	0.0082477	-0.414028274
6488	KCTD6	200845	0.000662474	-0.513375293
6489	DHFRL1	200895	0.008807475	-0.410922229
6490	RPL22L1	200916	0.005419738	-0.432961077
6491	OSTalpha	200931	1.56E-07	0.705868967
6492	GABRR3	200959	1.29E-05	0.620993404
6493	FAM18B2	201158	6.89E-06	0.634854886
6494	FLCN	201163	5.62E-05	0.585561763
6495	LOC201175	201175	0.009045521	-0.409593173
6496	LOC201181	201181	9.07E-10	0.780598945
6497	C17orf74	201243	0.004636568	0.43971729
6498	LRRC45	201255	0.000235996	0.546139956
6499	RDM1	201299	4.40E-05	0.591720114
6500	FBXO15	201456	1.10E-07	0.711439748
6501	LOC201477	201477	1.50E-07	0.706419809
6502	ZSCAN4	201516	7.48E-06	0.633100478
6503	STT3B	201595	5.00E-05	-0.588492918
6504	FAM116A	201627	1.02E-05	-0.626271385
6505	VSTM3	201633	0.001205963	0.492364369
6506	TMEM154	201799	0.000152862	0.558663814
6507	TMEM192	201931	0.002442544	-0.465991514
6508	RWDD4A	201965	0.00484864	-0.437824807
6509	Unknown	201973	0.00348266	-0.451735808
6510	LOC202051	202051	0.00021378	0.549056613
6511	RANBP3L	202151	2.02E-09	0.769741223
6512	LOC202181	202181	2.34E-08	0.73556122
6513	LOC202459	202459	0.000170689	0.555512999
6514	KHDRBS2	202559	1.61E-08	0.74119357
6515	LOC202781	202781	0.000925988	-0.501799671
6516	ADCK5	203054	0.003247151	-0.454606751
6517	LGI3	203190	1.03E-10	0.809395486
6518	C9orf91	203197	0.000429113	0.527682054
6519	C9orf93	203238	0.000359079	0.53332818

6520	C9orf90	203245	0.000520024	0.521521346
6521	CXorf24	203414	1.04E-07	0.712389197
6522	ZCCHC5	203430	0.003436007	0.452245215
6523	LOC203547	203547	0.001001241	-0.499006384
6524	TMEM31	203562	1.05E-05	0.625516612
6525	TMEM16E	203859	0.008564681	0.41219594
6526	LASS3	204219	1.82E-08	0.739369797
6527	NLRP11	204801	0.003385683	0.452851742
6528	PLAC9	219348	0.000236794	0.546033065
6529	MTIF3	219402	0.008575588	-0.412135488
6530	GSX1	219409	1.88E-11	0.836306934
6531	OR5AK4P	219525	0.000159628	0.557428932
6532	YPEL4	219539	5.86E-09	0.755329836
6533	MED19	219541	0.002631158	-0.463000741
6534	MGC26647	219557	0.000631193	0.515068436
6535	ZNF804B	219578	7.22E-06	0.633826561
6536	LOC219690	219690	4.99E-05	0.588542832
6537	LOC219731	219731	1.64E-07	0.70500675
6538	TYSND1	219743	0.004010261	-0.445839107
6539	CCNY	219771	0.003794969	-0.448156059
6540	C10orf27	219793	9.96E-11	0.809857747
6541	SLC37A2	219855	0.002941377	0.45858572
6542	TMEM136	219902	5.77E-07	0.683278208
6543	MRPL21	219927	0.008882952	-0.410497137
6544	SPATA19	219938	1.50E-08	0.742248346
6545	PLAC1L	219990	1.55E-08	0.741752095
6546	MGC35295	219995	1.00E-04	0.57045113
6547	CYBASC3	220002	1.09E-08	0.746651753
6548	LOC220077	220077	0.001739684	-0.478821985
6549	DLEU7	220107	0.009263577	0.408466016
6550	DOK6	220164	0.002495217	0.465116796
6551	HEPACAM	220296	3.20E-06	0.650691292
6552	TIGD3	220359	0.003572446	0.450682323
6553	LOC220906	220906	0.009380799	-0.407866796
6554	8-Mar	220972	0.00220179	-0.469926737
6555	LOC220980	220980	6.67E-07	0.680635367
6556	HNRPA3	220988	0.001189231	-0.492869825
6557	C10orf38	221061	0.00115829	-0.493796321
6558	ALKBH3	221120	6.48E-05	-0.581800247
6559	LOC221122	221122	6.35E-11	0.816068824
6560	EFHA1	221154	7.33E-05	-0.578632634
6561	GPR114	221188	3.91E-09	0.761029162
6562	CES7	221223	0.001700278	0.479714927
6563	C18orf20	221241	4.58E-05	0.590686533
6564	FAM26D	221301	1.31E-08	0.744189504

6565	C6orf189	221303	1.69E-10	0.802719295
6566	C6orf170	221322	0.001623296	-0.481450982
6567	OPN5	221391	0.007502227	0.418467058
6568	GPR115	221393	0.006871884	0.422553611
6569	MGC45491	221416	1.25E-09	0.776149327
6570	C6orf154	221424	4.27E-10	0.790231534
6571	TREML2P	221438	5.75E-05	0.584917655
6572	C6orf128	221468	1.54E-08	0.741859096
6573	PI16	221476	1.05E-08	0.747305413
6574	C6orf81	221481	0.000556113	0.519307766
6575	ZBTB12	221527	2.33E-08	0.735641451
6576	ZSCAN12L1	221584	1.88E-09	0.77070781
6577	PHACTR1	221692	9.90E-06	0.626877425
6578	C6orf218	221718	0.000788127	0.507468778
6579	ZNF498	221785	0.000210862	0.549462499
6580	LOC221814	221814	0.002708448	0.461816172
6581	PRPS1L1	221823	4.34E-09	0.759616336
6582	C7orf47	221908	0.0014462	-0.485741553
6583	C7orf27	221927	0.001068943	-0.496615859
6584	FOXK1	221937	0.000813905	-0.506325056
6585	LOC221946	221946	1.24E-06	0.669228695
6586	DAGLB	221955	0.001135377	-0.494518752
6587	C7orf28B	221960	4.10E-09	0.760368163
6588	VSTM2A	222008	7.70E-09	0.751593475
6589	TMED4	222068	0.003618611	-0.450139928
6590	RSBN1L	222194	0.003846004	-0.447570828
6591	FBXL13	222235	0.000456036	0.52577253
6592	LNX2	222484	0.003418819	-0.4524741
6593	GPRC6A	222545	0.00019518	0.55166211
6594	RFXDC1	222546	2.00E-07	0.701652136
6595	BZRPL1	222642	3.26E-10	0.793868296
6596	KCTD20	222658	0.00018681	-0.55292406
6597	LHFPL5	222662	0.009240705	0.408585007
6598	SCUBE3	222663	0.000538308	0.520369585
6599	ZSCAN23	222696	1.30E-11	0.844051727
6600	TMEM130	222865	1.43E-10	0.805140968
6601	FERD3L	222894	2.46E-09	0.76712122
6602	CCDC129	223075	7.87E-11	0.813094028
6603	SEMA3D	223117	0.00011411	0.566789183
6604	MAPK15	225689	7.37E-05	0.578505946
6605	SPDYA	245711	0.005666511	0.431083168
6606	MS4A6E	245802	8.86E-07	0.675362417
6607	VGLL2	245806	5.77E-09	0.755604763
6608	CNPY4	245812	4.54E-09	0.758965533
6609	DEFB119	245932	3.66E-07	0.691336643

6610	DEFB122	245935	0.001952478	0.474430338
6611	DEFB125	245938	6.07E-08	0.720834182
6612	ATP6V0D2	245972	1.65E-06	0.663754779
6613	TTTY7	246122	0.00171326	0.479418395
6614	CYorf15A	246126	4.94E-09	0.757707099
6615	GAS2L2	246176	7.18E-05	0.579200045
6616	CDC26	246184	0.000119643	-0.565426788
6617	LACE1	246269	1.02E-08	0.747656012
6618	D21S2089E	246312	0.001403117	0.486868477
6619	FLJ13773	246318	0.008534921	-0.412369064
6620	PELI3	246330	2.72E-08	0.733196008
6621	STH	246744	2.79E-07	0.695963552
6622	IL27	246778	9.28E-05	0.572431557
6623	TMEM9	252839	9.01E-05	-0.573218513
6624	ZNF396	252884	7.10E-10	0.783752332
6625	FNDC5	252995	1.33E-09	0.775253
6626	LOC253044	253044	2.36E-08	0.735462735
6627	C22orf30	253143	0.007539911	-0.418226532
6628	LOC253264	253264	6.39E-09	0.754187408
6629	LOC253724	253724	0.000205172	0.550213563
6630	MSRB3	253827	5.07E-07	0.685557745
6631	ZDHHC20	253832	1.33E-06	0.667849929
6632	YTHDF3	253943	0.000785682	-0.50757215
6633	GARNL1	253959	0.001656653	-0.480688373
6634	LOC253962	253962	6.27E-07	0.68175887
6635	KCTD13	253980	2.59E-11	0.830766675
6636	ASPHD1	253982	2.05E-05	0.610310897
6637	Unknown	254013	2.34E-08	0.7355966
6638	LOC254028	254028	0.000192134	0.55214711
6639	FLJ25778	254048	0.00182678	0.476960328
6640	LOC254100	254100	1.47E-07	0.706800081
6641	FLJ30934	254122	2.21E-06	0.658146817
6642	LOC254128	254128	0.002708448	-0.461812987
6643	CXorf58	254158	2.00E-10	0.800424969
6644	FBXO33	254170	0.000904184	-0.502709488
6645	TTLL10	254173	8.76E-09	0.749759218
6646	RNF169	254225	0.007527369	-0.418322023
6647	FAM26E	254228	0.00469007	0.439231743
6648	BPIL2	254240	0.003794017	0.44817561
6649	C1orf62	254268	9.42E-11	0.810571031
6650	FLJ40244	254272	0.009206028	-0.408759563
6651	LOC254312	254312	0.005128956	0.435390895
6652	LOC254559	254559	2.36E-10	0.798107497
6653	Unknown	254778	0.000436619	0.527158499
6654	FAM26C	255022	1.31E-08	0.74415561

6655	LOC255025	255025	1.71E-05	0.614498283
6656	MPV17L	255027	1.46E-10	0.804861886
6657	FLJ35390	255031	2.76E-08	0.732995882
6658	C19orf26	255057	6.02E-11	0.817104802
6659	CCDC108	255101	7.12E-11	0.814357611
6660	Unknown	255130	3.83E-10	0.791692133
6661	LOC255167	255167	0.003762093	0.448530553
6662	C19orf34	255193	1.61E-05	0.615938614
6663	C3orf46	255330	0.000132576	0.562598288
6664	LOC255374	255374	3.02E-06	0.651858947
6665	TCP11L2	255394	1.67E-09	0.772297272
6666	ZNF718	255403	1.13E-11	0.85111575
6667	LOC255411	255411	0.000219134	0.548315175
6668	LOC255480	255480	0.00934366	0.408040875
6669	RNF144B	255488	0.004817478	0.438091813
6670	HIST1H2BA	255626	0.00060379	0.516605835
6671	PCSK9	255738	6.36E-06	0.636529014
6672	C16orf65	255762	2.95E-09	0.764765878
6673	Unknown	255812	0.000217569	-0.548532421
6674	ADAM5P	255926	9.31E-10	0.780234702
6675	PAN3	255967	0.001323078	-0.489070066
6676	ANKRD31	256006	1.09E-10	0.808531823
6677	ZNF549	256051	3.16E-09	0.763803989
6678	COL29A1	256076	0.000226476	0.547358019
6679	SYCE2	256126	7.94E-10	0.782288096
6680	NUDT14	256281	0.008175855	-0.414427802
6681	CCDC110	256309	0.004046374	-0.445439614
6682	EML3	256364	0.006627437	-0.424190147
6683	SCML4	256380	9.87E-10	0.779290595
6684	ST6GALNAC3	256435	6.01E-06	0.637732214
6685	TCERG1L	256536	2.05E-09	0.769466837
6686	GLIPR1L1	256710	0.00078032	0.507803344
6687	NPB	256933	4.14E-10	0.790773149
6688	C17orf66	256957	0.00067467	0.512787052
6689	SERINC5	256987	0.007344635	-0.419459165
6690	PLAC2	257000	8.21E-05	0.575684233
6691	FRMD3	257019	2.38E-10	0.798016863
6692	PLCXD2	257068	0.000977699	0.499840045
6693	GCET2	257144	2.23E-06	0.657905273
6694	LOC257152	257152	0.000118917	0.565633685
6695	UTS2D	257313	7.59E-09	0.751812683
6696	C21orf87	257357	5.71E-10	0.786468645
6697	LOC257358	257358	1.05E-10	0.809218241
6698	MAP3K7IP3	257397	0.000164131	-0.556618832
6699	NALCN	259232	1.59E-06	0.664420064

6700	WFDC11	259239	4.42E-09	0.75938842
6701	WFDC9	259240	0.001851349	0.476433007
6702	MDS2	259283	0.009902979	0.405256244
6703	TAS2R39	259285	1.80E-08	0.739536363
6704	TAS2R41	259287	0.000648196	0.514188519
6705	TAS2R45	259291	7.61E-10	0.782891955
6706	TAS2R48	259294	0.000262843	0.54289807
6707	TAS2R50	259296	0.000203733	0.55044504
6708	MAGI3	260425	4.38E-10	0.789912974
6709	PRSS33	260429	4.91E-08	0.72420797
6710	C4orf7	260436	5.75E-10	0.786365887
6711	TIPRL	261726	0.000943251	-0.501076088
6712	NPHP4	261734	0.001188925	-0.492894071
6713	OFCC1	266553	1.87E-08	0.739025868
6714	DKFZp686A162	266695	0.001142944	0.494274834
6715	Rgr	266747	0.006555772	-0.424671247
6716	D21S2088E	266917	1.66E-08	0.740728794
6717	PGBD2	267002	0.007473815	-0.418660424
6718	C11orf31	280636	0.00085918	-0.504466973
6719	SSX7	280658	2.97E-06	0.652238774
6720	WFDC10B	280664	9.91E-09	0.748060435
6721	LOC280665	280665	1.21E-05	0.62250174
6722	IL28A	282616	2.77E-11	0.829376484
6723	IL29	282618	2.91E-08	0.732168272
6724	AQP11	282679	0.000533063	0.520683092
6725	OR51B5	282763	2.10E-08	0.737260795
6726	OR5J2	282775	8.31E-06	0.630791959
6727	C10orf53	282966	9.20E-10	0.7803852
6728	C10orf39	282973	3.02E-07	0.694567696
6729	LOC282980	282980	2.80E-05	0.602832886
6730	LOC282997	282997	1.50E-05	0.617530353
6731	LOC283033	283033	2.06E-11	0.834877146
6732	LOC283089	283089	2.96E-11	0.827608456
6733	TncRNA	283131	0.004944681	-0.43694422
6734	LOC283143	283143	2.82E-07	0.695766291
6735	FOXR1	283150	6.09E-08	0.720776267
6736	OR8D2	283160	0.00014713	0.559755086
6737	PRR10	283165	2.27E-08	0.736032702
6738	C11orf44	283171	1.00E-10	0.809733904
6739	Unknown	283194	1.09E-08	0.746649803
6740	P4HA3	283208	1.46E-09	0.77410547
6741	FLJ33790	283212	1.31E-07	0.708652895
6742	LOC283214	283214	1.67E-05	0.615086393
6743	Unknown	283232	0.004371863	-0.442145242
6744	LOC283267	283267	1.45E-09	0.774122504

6745	Unknown	283278	0.008461682	-0.41277011
6746	IGSF22	283284	0.000481258	-0.524038297
6747	C11orf36	283303	2.31E-06	0.657140346
6748	UNQ2963	283314	1.66E-10	0.803014746
6749	LOC283352	283352	2.42E-08	0.735064071
6750	ANKRD52	283373	0.00952987	-0.407122883
6751	SPRYD4	283377	9.90E-10	0.779228698
6752	LOC283387	283387	2.64E-08	0.733658325
6753	LOC283392	283392	1.70E-09	0.772011157
6754	FLJ33996	283401	1.63E-05	0.615659875
6755	LOC283403	283403	2.27E-09	0.768213273
6756	LOC283435	283435	1.91E-08	0.738687719
6757	MYO1H	283446	6.38E-06	0.636476874
6758	MUC19	283463	4.57E-08	0.725352461
6759	Unknown	283482	9.38E-05	0.572141882
6760	OR7E156P	283491	4.03E-05	0.593890324
6761	SUGT1L1	283507	1.63E-06	0.664011676
6762	LOC283516	283516	0.000767107	0.508428917
6763	LOC283547	283547	0.000982972	0.499665857
6764	GPR137C	283554	0.000369503	0.532379815
6765	ZDHHC22	283576	3.09E-05	0.600426879
6766	TMED8	283578	0.001817721	-0.477138099
6767	C14orf178	283579	0.009887456	0.405339748
6768	LOC283585	283585	1.28E-05	0.621146972
6769	LOC283587	283587	0.000335396	0.535500265
6770	LOC283588	283588	3.31E-05	0.598805431
6771	C14orf68	283600	4.69E-08	0.724946041
6772	C14orf70	283601	0.000125223	0.564162407
6773	TSSK4	283629	7.07E-06	0.634296733
6774	KIAA0284	283638	0.001141315	-0.494324378
6775	PRTG	283659	0.000841999	0.505155257
6776	LOC283663	283663	3.04E-07	0.694470099
6777	LOC283674	283674	0.004295672	0.442921131
6778	LOC283683	283683	2.87E-11	0.828387823
6779	LOC283728	283728	2.52E-07	0.697683726
6780	LOC283755	283755	3.94E-09	0.760915404
6781	FLJ39743	283777	0.000392177	0.530553622
6782	FLJ35785	283796	0.009976242	0.404923662
6783	CCDC79	283847	9.88E-08	0.71320468
6784	LOC283856	283856	5.42E-06	0.639921014
6785	C16orf81	283860	0.000360094	0.533205119
6786	LOC283868	283868	5.48E-10	0.787027661
6787	NPW	283869	0.007473997	-0.418654307
6788	LOC283887	283887	1.42E-06	0.666619879
6789	LOC283888	283888	1.81E-06	0.661914486

6790	LOC283902	283902	1.12E-09	0.777658941
6791	LOC283914	283914	5.71E-05	0.58510495
6792	NUDT7	283927	0.000213562	0.549089254
6793	LOC283932	283932	8.55E-06	0.630180186
6794	MGC46336	283933	1.29E-05	0.620922217
6795	C17orf28	283987	0.000208163	0.54982075
6796	TSEN54	283989	0.006578491	-0.424524148
6797	LOC283999	283999	2.04E-06	0.659718933
6798	CCDC57	284001	2.72E-05	0.60355023
6799	LOC284023	284023	8.23E-07	0.67675284
6800	KIAA1267	284058	6.82E-05	-0.580488539
6801	LOC284080	284080	9.53E-07	0.674039165
6802	C17orf47	284083	4.20E-06	0.645168553
6803	PIGW	284098	0.00952987	-0.40711919
6804	C17orf78	284099	0.00210357	0.471665211
6805	LOC284100	284100	2.63E-06	0.654665207
6806	SLC26A11	284129	0.007364417	-0.419333759
6807	FLJ35220	284131	4.95E-06	0.641790258
6808	GDPD1	284161	8.02E-05	0.576278889
6809	C17orf89	284184	0.005043373	-0.436124974
6810	METRNL	284207	0.006659837	-0.423962857
6811	LOC284219	284219	0.00017997	0.554008649
6812	LOC284240	284240	3.48E-07	0.692203624
6813	FAM44C	284257	0.003860863	0.447391444
6814	LOC284260	284260	0.002622404	0.463151544
6815	LOC284263	284263	0.001364151	0.487949678
6816	ZADH2	284273	0.009310476	-0.408198225
6817	ZIK1	284307	0.000559186	0.519133011
6818	ZNF776	284309	0.004994956	-0.43651568
6819	ZSCAN1	284312	1.78E-08	0.739754001
6820	ZNF780A	284323	7.91E-05	0.576707796
6821	MGC70924	284338	2.04E-11	0.835211292
6822	TMEM145	284339	2.63E-08	0.733707464
6823	ZNF575	284346	0.000354905	0.533679496
6824	FLJ36070	284358	1.64E-10	0.803274074
6825	SIGLECP3	284367	4.77E-09	0.758194279
6826	ZNF615	284370	0.000769953	0.508302349
6827	Unknown	284385	0.002778012	0.460775813
6828	FLJ14959	284391	1.73E-10	0.802240898
6829	LOC284402	284402	1.30E-11	0.845853029
6830	SLC25A42	284439	8.92E-11	0.811342416
6831	HKR1	284459	0.005094367	-0.43567126
6832	SLC9A11	284525	6.93E-08	0.718738673
6833	Unknown	284541	9.78E-07	0.673555238
6834	LOC284551	284551	0.007156028	0.420731711

6835	LOC284578	284578	4.46E-09	0.759221977
6836	Unknown	284610	5.65E-05	0.585370742
6837	SYPL2	284612	2.02E-08	0.737860277
6838	CYB561D1	284613	0.00055007	-0.519690015
6839	ANKRD34A	284615	5.91E-07	0.682829821
6840	LOC284648	284648	0.000585687	0.517626646
6841	DKFZP564C196	284649	4.78E-07	0.686542936
6842	EPHA10	284656	0.000110334	0.567764279
6843	LOC284669	284669	7.64E-09	0.751713131
6844	Unknown	284677	0.000841028	-0.505195378
6845	C1orf111	284680	7.06E-12	0.859244491
6846	ZNF326	284695	0.00289144	-0.459253541
6847	FAM80A	284716	0.001566516	0.482732476
6848	SLC25A34	284723	9.42E-11	0.810539446
6849	LOC284798	284798	1.32E-06	0.667946816
6850	Unknown	284804	2.25E-10	0.798858867
6851	FLJ33706	284805	3.75E-10	0.79200826
6852	C21orf130	284835	0.009874229	-0.405419065
6853	LOC284865	284865	1.76E-09	0.771522462
6854	LOC284898	284898	9.07E-11	0.811087286
6855	LOC284912	284912	1.33E-06	0.667794689
6856	LOC284939	284939	4.39E-11	0.822063029
6857	FLJ39660	284992	1.13E-05	0.624006422
6858	LOC285045	285045	0.00251753	0.464773676
6859	C2orf61	285051	0.009400735	0.407736401
6860	LOC285074	285074	0.000340664	-0.535017114
6861	Unknown	285150	0.005917881	0.429123963
6862	LOC285191	285191	1.01E-08	0.747789459
6863	DUSP28	285193	0.001672524	-0.480304801
6864	LOC285194	285194	5.81E-05	0.584647133
6865	FBXW12	285231	0.001918498	-0.475086087
6866	ZNF619	285267	1.08E-09	0.778188034
6867	LOC285286	285286	1.01E-07	0.71289366
6868	LOC285300	285300	1.33E-08	0.743914355
6869	IGSF10	285313	6.65E-05	0.581123428
6870	C3orf23	285343	0.00062998	0.515147514
6871	ZNF660	285349	2.26E-11	0.833194243
6872	SUMF1	285362	0.000828736	-0.505696638
6873	PRRT3	285368	0.00989686	-0.405296387
6874	LOC285382	285382	6.81E-06	0.635083945
6875	LOC285389	285389	4.07E-09	0.760461024
6876	LOC285422	285422	4.08E-05	0.593599932
6877	LOC285423	285423	0.007344635	0.419458288
6878	FLJ35424	285492	0.001973537	0.474035504
6879	RNF212	285498	7.74E-06	0.632334835

6880	LOC285500	285500	0.005089888	0.43571411
6881	FAM13A1OS	285512	5.63E-06	0.639096217
6882	GPRIN3	285513	5.69E-10	0.786528974
6883	COX18	285521	8.44E-05	0.574973168
6884	FRYL	285527	0.003100435	-0.456530601
6885	LOC285550	285550	0.000361914	-0.533013147
6886	C4orf37	285555	0.000562501	0.518922961
6887	LOC285577	285577	9.98E-05	0.570504702
6888	SH3PXD2B	285590	0.000338439	0.535219033
6889	LOC285593	285593	0.000680037	0.512533226
6890	RELL2	285613	6.70E-07	0.680545022
6891	LOC285627	285627	1.11E-05	0.624427986
6892	LOC285638	285638	6.90E-11	0.814772698
6893	LOC285708	285708	6.36E-09	0.754273959
6894	LOC285733	285733	8.98E-08	0.714665167
6895	LOC285740	285740	2.96E-07	0.694931909
6896	Unknown	285754	4.08E-07	0.689337479
6897	LOC285766	285766	0.003623182	0.450084809
6898	LOC285768	285768	1.14E-09	0.777389104
6899	RP3-398D13.1	285780	0.000250237	0.544361102
6900	Unknown	285847	2.81E-05	0.602723507
6901	PNPLA1	285848	1.24E-05	0.621902394
6902	TREML4	285852	2.23E-09	0.76840229
6903	RPL7L1	285855	0.007096721	-0.421122465
6904	DKFZp564N247	285877	1.21E-06	0.669709522
6905	LOC285889	285889	0.005666511	0.431070629
6906	LOC285908	285908	3.00E-07	0.694686641
6907	LOC285941	285941	0.006846769	0.422731232
6908	Unknown	285943	5.79E-06	0.638539734
6909	Unknown	285949	1.90E-09	0.77050027
6910	C7orf40	285958	0.001877474	-0.47588384
6911	FLJ40852	285962	1.09E-08	0.746623452
6912	LOC285965	285965	5.48E-06	0.63966499
6913	FAM139A	285966	0.001810453	0.477301012
6914	Unknown	285971	1.09E-05	0.624814182
6915	LOC285972	285972	2.34E-06	0.656921527
6916	FLJ34048	285987	0.007566617	0.418074048
6917	tcag7.929	286009	7.02E-07	0.679652137
6918	FLJ40288	286023	1.55E-11	0.839665761
6919	NSMCE2	286053	9.46E-07	0.674195624
6920	LOC286059	286059	1.78E-07	0.703672311
6921	LOC286068	286068	0.001719728	0.479260227
6922	FAM83H	286077	0.007327794	-0.419603843
6923	LOC286083	286083	2.06E-05	0.610184403
6924	LOC286114	286114	7.31E-07	0.678893073

6925	C8orf31	286122	9.14E-07	0.674813099
6926	SCARA5	286133	8.00E-07	0.677277178
6927	LOC286149	286149	1.22E-09	0.776456196
6928	FBXO43	286151	5.80E-07	0.683155419
6929	LOC286161	286161	5.39E-07	0.684473212
6930	LOC286177	286177	0.003483548	0.451711302
6931	LOC286178	286178	4.44E-10	0.789748234
6932	LOC286189	286189	2.30E-11	0.832677484
6933	LOC286190	286190	0.00013537	0.562048619
6934	LOC286238	286238	0.008612883	0.411943756
6935	FAM78A	286336	8.50E-06	0.630303768
6936	LOC286370	286370	6.34E-06	0.636606706
6937	LOC286411	286411	4.16E-05	0.593101196
6938	H2BFM	286436	4.75E-07	0.68666075
6939	LOC286442	286442	6.67E-10	0.784527532
6940	YIPF6	286451	0.009547008	0.407037034
6941	MAGEB18	286514	0.000656122	-0.513745421
6942	P2RY8	286530	0.008444415	0.412865913
6943	Unknown	286756	1.72E-08	0.740249775
6944	TRIM42	287015	0.007495457	0.418512807
6945	EIF4E3	317649	0.000194879	0.551708106
6946	RFESD	317671	6.00E-12	0.861133666
6947	VN1R2	317701	4.54E-05	0.590940828
6948	VN1R5	317705	8.50E-06	0.630319445
6949	RP11-49G10.8	317716	1.68E-09	0.772197877
6950	KLHL10	317719	0.000218099	0.548456838
6951	C14orf25	319089	6.34E-06	0.636623808
6952	KRT73	319101	1.49E-11	0.840594129
6953	MMAB	326625	0.007035297	-0.421492451
6954	SERPINA9	327657	0.001820325	0.477086171
6955	PPM1J	333926	0.005922723	-0.42907241
6956	KRTAP7-1	337878	0.000766795	0.508446718
6957	KRTAP8-1	337879	7.70E-09	0.751604093
6958	KRTAP11-1	337880	1.87E-08	0.738977961
6959	KRTAP19-1	337882	0.000224968	0.547544574
6960	NLRP10	338322	2.31E-10	0.798399154
6961	LOC338328	338328	2.33E-08	0.735638905
6962	CLEC4D	338339	4.21E-10	0.790496147
6963	RAB7B	338382	0.000859537	0.50444477
6964	LOC338588	338588	1.42E-06	0.666555513
6965	LUZP2	338645	1.90E-08	0.738772255
6966	PMP22CD	338661	1.63E-09	0.772600726
6967	LOC338809	338809	2.42E-08	0.735052512
6968	LOC338817	338817	2.78E-11	0.829202755
6969	Unknown	338862	4.80E-06	0.642426159

6970	C1QTNF9	338872	0.000220325	0.54815557
6971	LOC338963	338963	8.45E-07	0.676253378
6972	RAB43	339122	0.000129588	0.563207699
6973	Unknown	339166	8.41E-05	0.57505336
6974	TMEM95	339168	7.52E-07	0.678384073
6975	ENPP7	339221	4.69E-06	0.642919484
6976	C17orf90	339229	0.000249614	-0.544439342
6977	ARL16	339231	0.001025273	-0.498154056
6978	LOC339240	339240	0.008346133	0.413495705
6979	C17orf51	339263	7.37E-09	0.752243304
6980	MSL-1	339287	0.001032359	-0.497904711
6981	CLEC4G	339390	1.67E-06	0.663493113
6982	C1orf174	339448	0.005784185	-0.430177928
6983	LOC339468	339468	1.79E-10	0.801833887
6984	ZBTB8OS	339487	0.009638607	0.406593135
6985	LOC339539	339539	9.27E-07	0.674552629
6986	LOC339568	339568	0.009840697	0.405581395
6987	LOC339593	339593	4.49E-10	0.789614885
6988	SLC35E4	339665	0.009117898	0.409199609
6989	LOC339666	339666	4.22E-07	0.688793755
6990	LOC339751	339751	1.27E-07	0.70912857
6991	LOC339766	339766	2.37E-09	0.767617736
6992	ESPNL	339768	0.000286969	0.540271106
6993	C2orf53	339779	5.57E-05	0.585782846
6994	C2orf46	339789	2.41E-06	0.656358382
6995	CCDC36	339834	0.009765215	0.405946987
6996	LOC339874	339874	0.000665582	0.513221931
6997	C3orf35	339883	0.002688886	0.462120189
6998	FLJ25770	339965	2.82E-10	0.795673257
6999	Unknown	339978	9.07E-11	0.811078042
7000	LOC340037	340037	2.46E-08	0.734798938
7001	Unknown	340085	0.002139887	-0.47102363
7002	LOC340094	340094	2.45E-07	0.698155602
7003	RP11-145H9.1	340156	0.003313463	0.453764254
7004	LOC340178	340178	9.81E-07	0.673472195
7005	TREML1	340205	2.79E-07	0.695954825
7006	TREML3	340206	0.000680837	0.512493192
7007	LOC340239	340239	5.67E-05	0.585283918
7008	ABCB5	340273	0.005139021	0.435292467
7009	tcag7.1188	340340	2.87E-11	0.828377527
7010	LOC340357	340357	1.37E-12	0.875073119
7011	RSPO2	340419	0.004099012	0.444907707
7012	RP11-262D11.5	340527	3.01E-06	0.651946782
7013	VSIG1	340547	2.14E-08	0.736924461
7014	ZC3H12B	340554	0.005497003	0.43233648

7015	LHFPL1	340596	0.000351952	0.533961458
7016	C10orf112	340895	3.38E-06	0.649596575
7017	ANKRD33	341405	2.90E-11	0.828222299
7018	H1FNT	341567	0.000373631	0.532045718
7019	FREM2	341640	0.002588832	0.463663186
7020	GOLGA6	342096	0.001744826	0.47869363
7021	FMN1	342184	1.26E-06	0.668835718
7022	ZKSCAN2	342357	1.06E-10	0.808853838
7023	SMTNL2	342527	3.55E-07	0.691864549
7024	STAC2	342667	2.70E-05	0.603755527
7025	ZNF284	342909	0.000222233	0.547918913
7026	ZSCAN22	342945	5.38E-08	0.722769799
7027	NANOS3	342977	0.001407011	0.48675884
7028	PRAMEF10	343071	6.57E-11	0.81535916
7029	RSPO4	343637	8.02E-06	0.631570327
7030	FIGLA	344018	1.07E-06	0.67191871
7031	PAQR9	344838	4.27E-05	0.592461108
7032	ATP13A5	344905	1.26E-10	0.806584881
7033	PLCXD3	345557	1.62E-05	0.615823368
7034	ZNF391	346157	1.64E-10	0.803239157
7035	7A5	346389	3.68E-10	0.79231251
7036	GNAT3	346562	4.65E-05	0.590268469
7037	MOGAT3	346606	1.93E-08	0.738496418
7038	FAM71F2	346653	7.52E-10	0.783025714
7039	ZNF81	347344	1.69E-05	0.614820218
7040	LOC347475	347475	4.72E-08	0.724800537
7041	TXNDC6	347736	3.76E-11	0.824455692
7042	C6orf52	347744	1.48E-05	0.617836392
7043	TBX10	347853	0.00027991	0.541032716
7044	PDDC1	347862	0.009127527	-0.409152495
7045	EP400NL	347918	2.60E-08	0.7339351
7046	RBPMS2	348093	1.90E-07	0.702522334
7047	ANKDD1A	348094	0.003165656	0.45563313
7048	LOC348120	348120	9.25E-05	0.572521052
7049	LOC348262	348262	0.006271149	-0.426608149
7050	MGC52498	348378	2.05E-10	0.800059842
7051	FAM131C	348487	0.000900075	0.502869467
7052	LOC348801	348801	3.92E-09	0.7609879
7053	LOC348808	348808	0.000143121	0.560502979
7054	TPRXL	348825	1.69E-08	0.740512969
7055	ICHTHYIN	348938	3.38E-08	0.729838012
7056	NUP43	348995	0.004944276	-0.436952487
7057	LOC349114	349114	1.65E-06	0.663757065
7058	RTN4RL2	349667	4.32E-07	0.688335016
7059	C6orf58	352999	6.44E-05	0.582008395

7060	RILPL1	353116	2.46E-09	0.767125746
7061	LCE1E	353135	1.73E-09	0.771826761
7062	LGICZ1	353174	0.000428001	0.527760058
7063	SPATA12	353324	6.25E-08	0.720362439
7064	BMP8A	353500	0.003107246	0.456433258
7065	Unknown	353514	0.001165318	0.493570291
7066	IRF2BP2	359948	0.000879397	-0.503634207
7067	GSTK1	373156	0.008004399	-0.415483364
7068	CDRT1	374286	0.001990745	0.473697493
7069	C10orf96	374355	5.36E-09	0.75661944
7070	TBC1D10C	374403	1.42E-06	0.666573374
7071	LOC374443	374443	2.41E-06	0.656330534
7072	C12orf42	374470	0.009853003	0.405526019
7073	HDDC3	374659	0.001925983	-0.47493705
7074	FAM39DP	374666	0.007495457	-0.418512337
7075	TEPP	374739	1.79E-07	0.703583138
7076	Unknown	374768	0.001268785	0.490529037
7077	EFCAB5	374786	1.09E-08	0.74671329
7078	C18orf34	374864	2.94E-07	0.695058575
7079	ATP9B	374868	0.000128807	0.56337019
7080	C19orf45	374877	6.32E-07	0.681610621
7081	UNQ501	374882	0.001545137	-0.483270178
7082	LOC374890	374890	1.76E-07	0.703871768
7083	ZNF568	374900	0.000118917	0.56564214
7084	IGFL1	374918	0.002773797	0.460845017
7085	C1orf187	374946	6.88E-10	0.784140141
7086	LOC374973	374973	0.005203725	0.43476336
7087	Unknown	374977	0.005942647	-0.428936032
7088	Unknown	375387	7.87E-11	0.813115792
7089	C5orf25	375484	0.002378316	-0.467069654
7090	GUSBL2	375513	1.22E-10	0.807026735
7091	C9orf165	375704	9.88E-10	0.779263984
7092	PTAR1	375743	0.00061645	-0.515926452
7093	AGRN	375790	0.009570952	-0.406911204
7094	MGC59937	375791	0.000875944	0.503775071
7095	RNF126P1	376412	1.15E-11	0.850523286
7096	ZC3H6	376940	0.004253631	-0.443357924
7097	Unknown	378820	7.99E-07	0.677319266
7098	RNF148	378925	1.63E-06	0.663964827
7099	LOC386597	386597	3.57E-11	0.825331235
7100	KRTAP10-11	386678	1.03E-05	0.626040738
7101	BC036928	386758	1.61E-11	0.8379975
7102	Unknown	387036	0.000681735	0.512437253
7103	Unknown	387097	0.007833943	0.416482653
7104	C6orf174	387104	3.27E-06	0.650282149

7105	C21orf131	387486	2.07E-07	0.701095023
7106	RASL11A	387496	1.34E-10	0.805934975
7107	psiTPTE22	387590	1.29E-09	0.775664202
7108	UST6	387601	2.62E-07	0.697052724
7109	CC2D2B	387707	5.31E-11	0.819413234
7110	GVIN1	387751	0.000492935	0.523269028
7111	INSC	387755	3.78E-07	0.690748508
7112	LOC387790	387790	0.004017183	0.445761668
7113	CLEC12B	387837	1.35E-08	0.743741969
7114	C13orf21	387923	3.50E-11	0.825597567
7115	C14orf23	387978	2.88E-06	0.652837786
7116	FAM148B	388125	0.000120465	0.565250089
7117	LOC388135	388135	0.006812273	0.422943749
7118	FLJ42289	388182	0.003682341	0.449401996
7119	LOC388284	388284	2.79E-11	0.828954602
7120	LOC388381	388381	0.000249614	0.544439036
7121	FLJ31222	388387	0.000397041	0.53016617
7122	LOC388419	388419	7.70E-10	0.782712169
7123	FLJ12120	388439	0.000661132	0.513451688
7124	RGS9BP	388531	2.34E-05	0.60714985
7125	KRTDAP	388533	5.54E-11	0.81865188
7126	BLOC1S3	388552	6.25E-11	0.816321368
7127	ZNF470	388566	0.000701853	0.511461131
7128	SLC6A17	388662	5.92E-06	0.638033498
7129	FLJ36116	388666	0.001786921	0.477768797
7130	NOTCH2NL	388677	0.006553362	-0.424690426
7131	FLG2	388698	4.66E-09	0.758551204
7132	C1orf46	388699	2.93E-08	0.732060886
7133	FMO6P	388714	1.01E-07	0.712889597
7134	LOC388789	388789	0.006128475	-0.427590878
7135	LOC388796	388796	0.003184512	-0.455373671
7136	LOC388820	388820	3.19E-07	0.693647458
7137	LOC388882	388882	6.67E-07	0.680650424
7138	C22orf36	388886	1.74E-09	0.771677814
7139	LOC388889	388889	4.25E-10	0.790321259
7140	LOC388906	388906	1.70E-06	0.66309592
7141	LOC388948	388948	0.000563562	0.518863289
7142	BOLA3	388962	0.002875368	-0.45948214
7143	LOC388963	388963	5.46E-10	0.787094739
7144	LOC388969	388969	0.002564878	-0.464053176
7145	MUSTN1	389125	4.16E-08	0.726752076
7146	FLJ16641	389170	0.000752876	0.50909057
7147	LOC389199	389199	9.00E-06	0.628986106
7148	CCDC4	389206	2.05E-07	0.701229969
7149	LOC389247	389247	0.005444984	0.432762609

7150	C5orf39	389289	0.007337434	-0.419506697
7151	FLJ27505	389320	0.00047193	0.524682805
7152	MGC23985	389336	0.001599547	0.481983292
7153	LOC389541	389541	0.000503092	-0.522581228
7154	C9orf144	389715	0.000941093	0.501169053
7155	FLJ46321	389763	1.57E-11	0.838996805
7156	IER5L	389792	0.005490924	-0.432393274
7157	UNQ2541	389812	0.007411626	0.419040386
7158	MAP3K15	389840	0.000178482	0.554257991
7159	USP27X	389856	6.69E-08	0.719311037
7160	tAKR	389932	0.005474187	0.432532766
7161	OR51B6	390058	1.68E-09	0.772212241
7162	OR51M1	390059	0.000253998	0.5439312
7163	OR51I1	390063	6.19E-11	0.816548061
7164	OR52D1	390066	0.002006048	0.473413336
7165	LOC390595	390595	7.90E-06	0.631919444
7166	ANKRD34C	390616	5.17E-05	0.587662509
7167	ZNF793	390927	0.004425237	-0.441633495
7168	LOC390940	390940	0.005453685	-0.43268243
7169	ZNF805	390980	0.004854976	0.43775602
7170	PRAMEF12	390999	4.89E-06	0.642026679
7171	LOC391267	391267	9.68E-10	0.779636796
7172	Unknown	392459	3.40E-10	0.793349594
7173	Unknown	394261	0.006990899	0.421766347
7174	SLC25A35	399512	1.27E-10	0.806445291
7175	FAM102A	399665	3.52E-08	0.729251885
7176	ZNF321	399669	2.75E-05	0.603299058
7177	MGC34774	399670	2.22E-05	0.608410145
7178	HEATR4	399671	0.001055119	0.497109651
7179	MGC50722	399693	0.005624124	-0.431418429
7180	DKFZp667F071[illegible]	399716	0.000328895	0.536151671
7181	C10orf114	399726	0.000660374	0.513490829
7182	RP11-144G6.7	399753	1.09E-06	0.671548458
7183	BMS1P5	399761	3.82E-07	0.690565602
7184	FLJ41350	399806	8.35E-09	0.750428973
7185	FLJ44653	399833	0.00070874	0.511129263
7186	LOC399875	399875	3.22E-08	0.730597155
7187	LOC399884	399884	0.000517888	0.521673463
7188	LOC399900	399900	0.001635117	0.481179856
7189	SNX19	399979	0.004495027	0.440994061
7190	LOC400027	400027	0.001080682	-0.496230991
7191	FLJ40142	400073	1.02E-05	0.626305071
7192	FLJ39779	400223	0.000271302	0.541960739
7193	LOC400238	400238	3.66E-09	0.761970322
7194	C14orf180	400258	1.59E-06	0.664442666

7195	C15orf54	400360	0.000415284	0.528739768
7196	Unknown	400410	7.20E-10	0.78358282
7197	LOC400456	400456	5.14E-07	0.685341409
7198	LOC400506	400506	1.82E-09	0.771093404
7199	FLJ21408	400512	2.66E-10	0.796459933
7200	LOC400581	400581	0.005817866	0.429903316
7201	FLJ38028	400643	8.53E-05	0.574686444
7202	LOC400654	400654	0.001409314	0.486679316
7203	LOC400655	400655	3.69E-09	0.761837972
7204	SIGLECP16	400709	7.70E-05	0.577394469
7205	LOC400748	400748	1.73E-09	0.771819887
7206	LOC400752	400752	0.000807961	0.50663492
7207	LOC400756	400756	0.002413509	0.466479707
7208	LOC400794	400794	8.18E-10	0.781926617
7209	FLJ90680	400926	0.000634456	0.514866639
7210	FLJ27365	400931	2.88E-06	0.652817897
7211	FLJ12334	400946	0.002241331	0.469251295
7212	Unknown	400949	9.65E-06	0.627439624
7213	FLJ30838	400955	0.006382902	0.425851554
7214	RGPD1	400966	2.28E-06	0.657400995
7215	DKFZp686O132	401014	6.12E-11	0.816869011
7216	LOC401022	401022	2.35E-05	0.607069131
7217	IQCF3	401067	0.003579887	0.450598512
7218	MGC48628	401145	8.41E-10	0.781600481
7219	LOC401152	401152	0.008338221	-0.41355867
7220	FLJ44606	401207	0.007797555	0.41670517
7221	FLJ22536	401237	2.11E-08	0.737172553
7222	DKFZp451B082	401282	8.31E-07	0.67658403
7223	C6orf122	401288	1.25E-08	0.744816547
7224	LOC401312	401312	1.80E-08	0.739535944
7225	LOC401321	401321	9.50E-07	0.674104457
7226	LOC401324	401324	5.99E-08	0.721057277
7227	LOC401397	401397	7.93E-11	0.812982324
7228	LOC401431	401431	0.006717607	0.423581759
7229	LOC401463	401463	0.007633544	0.417665414
7230	C8orf59	401466	0.000616658	-0.515904933
7231	FLJ35024	401491	0.000765184	0.508538886
7232	LOC401504	401504	0.000249119	-0.544522151
7233	Unknown	401505	0.002606515	-0.463389671
7234	FLJ25917	401585	1.90E-06	0.661091449
7235	Unknown	401588	0.006148421	-0.427452835
7236	MCART6	401612	1.08E-05	0.625010158
7237	C10orf132	401647	2.76E-07	0.696096632
7238	MGC57346	401884	5.03E-10	0.788163484
7239	Unknown	402160	8.01E-06	0.63163115

7240	SOHLH1	402381	7.28E-06	0.633669923
7241	LOC402779	402779	5.88E-11	0.817631218
7242	PPAPDC2	403313	0.001841171	-0.476650595
7243	APOBEC4	403314	2.20E-08	0.736502336
7244	MGC70870	403340	0.002398905	-0.466704758
7245	HAPLN4	404037	3.59E-05	0.596781217
7246	CUEDC1	404093	0.005060949	-0.435969263
7247	C4orf12	404201	2.62E-08	0.733777056
7248	C10orf85	404216	0.000139197	0.561272537
7249	C6orf201	404220	0.007288292	0.419848551
7250	AAA1	404744	3.35E-08	0.729990882
7251	HERV-FRD	405754	1.07E-06	0.671890245
7252	Unknown	406996	0.000609155	0.516321961
7253	C10orf31	414196	2.32E-10	0.798309797
7254	C10orf103	414245	1.02E-05	0.626307835
7255	LOC414300	414300	4.24E-05	0.59265786
7256	DDI1	414301	0.000445048	0.526518483
7257	C9orf103	414328	1.62E-08	0.741067183
7258	Unknown	414777	4.38E-06	0.644288649
7259	BLID	414899	0.004569035	0.440328807
7260	MGC70857	414919	0.004261634	-0.443275952
7261	C15orf50	414926	2.05E-09	0.769539675
7262	BC038740	415056	3.26E-08	0.730396962
7263	C12orf37	439916	0.000425525	0.527956976
7264	MXRA7	439921	0.001470253	-0.485119594
7265	C1orf180	439927	1.07E-07	0.711870984
7266	Unknown	439931	4.15E-11	0.822727312
7267	Unknown	439943	0.005925992	0.429046499
7268	FLJ32742	439944	1.37E-06	0.667353901
7269	LOC439951	439951	2.32E-10	0.798316375
7270	LOC439990	439990	3.72E-10	0.792121803
7271	KRTAP5-2	440021	2.56E-08	0.7341302
7272	TMEM41B	440026	0.000434901	-0.527286013
7273	LOC440028	440028	0.001307184	0.48948706
7274	IQSEC3	440073	2.45E-06	0.655998624
7275	FLJ12825	440101	2.90E-05	0.602009618
7276	LOC440117	440117	2.70E-05	0.603689671
7277	FLJ31945	440137	0.001017878	0.498402515
7278	MGC88374	440184	0.003224496	-0.454871225
7279	FLJ41170	440200	2.23E-11	0.833776084
7280	Unknown	440253	6.95E-09	0.753035961
7281	LOC440268	440268	4.18E-05	0.593007641
7282	LOC440356	440356	0.000714189	0.510841215
7283	MGC71993	440400	0.000766795	-0.508450483
7284	CLEC4GP1	440508	3.96E-07	0.689918161

7285	FLJ42875	440556	1.09E-09	0.778018699
7286	Unknown	440574	0.001634119	-0.481206854
7287	LOC440602	440602	7.80E-05	0.577080095
7288	NUDT4P1	440672	4.68E-07	0.686929293
7289	LRRC52	440699	6.99E-05	0.579890429
7290	TRIM67	440730	6.63E-08	0.719458547
7291	MGC44328	440757	9.78E-05	0.57104553
7292	MIAT	440823	2.14E-11	0.834323381
7293	CAPN14	440854	0.004690117	-0.439226096
7294	LOC440864	440864	8.52E-09	0.75013368
7295	LOC440888	440888	0.003653117	0.449720246
7296	LOC440905	440905	1.59E-11	0.838563312
7297	FLJ33065	440952	2.15E-07	0.700431859
7298	LOC440957	440957	1.25E-10	0.806683699
7299	LOC440983	440983	6.17E-07	0.682060728
7300	Unknown	440993	5.54E-06	0.639422008
7301	LOC441046	441046	6.50E-05	0.58172313
7302	11-Mar	441061	0.000415832	0.528691283
7303	LOC441086	441086	2.12E-07	0.700638896
7304	FLJ42709	441094	4.08E-05	0.593565364
7305	LOC441124	441124	2.17E-10	0.799341535
7306	LOC441150	441150	0.008049875	-0.415231094
7307	TMEM151B	441151	2.63E-05	0.604388995
7308	hCG_1820801	441167	3.34E-09	0.763125933
7309	LOC441204	441204	0.008576336	0.412126926
7310	LOC441212	441212	1.29E-06	0.668465917
7311	FLJ39080	441355	1.24E-07	0.709561127
7312	Unknown	441394	0.00010692	0.568638652
7313	LOC441461	441461	1.73E-10	0.802270518
7314	LOC441476	441476	0.004066645	0.445224033
7315	LOC441528	441528	0.000210715	0.549486763
7316	LOC441601	441601	3.16E-05	0.599881563
7317	LOC441617	441617	4.26E-06	0.644835989
7318	LOC442028	442028	3.09E-05	0.600464155
7319	OR2B3P	442184	0.000904184	0.502704163
7320	OR5U1	442191	1.01E-06	0.67289624
7321	OR10C1	442194	1.38E-07	0.707873898
7322	LOC442240	442240	1.14E-06	0.670797729
7323	LMOD2	442721	6.23E-09	0.754563476
7324	LOC474358	474358	4.84E-07	0.686309895
7325	C8orf22	492307	9.64E-06	0.627486608
7326	MGC52110	493753	1.85E-05	-0.612723963
7327	Unknown	494115	3.08E-10	0.79461261
7328	RNF165	494470	0.007720795	0.417136325
7329	C18orf56	494514	6.70E-08	0.719272529

7330	LOC494558	494558	2.29E-08	0.735894597
7331	TIFAB	497189	0.000373372	0.53207076
7332	LOC497256	497256	1.49E-07	0.706607827
7333	BDNFOS	497258	1.95E-10	0.800703293
7334	BEYLA	497634	0.000108126	0.56831462
7335	LOC503519	503519	1.90E-06	0.660984776
7336	FLJ35946	503569	3.99E-09	0.760701488
7337	Unknown	541472	4.50E-10	0.789545838
7338	LOC541473	541473	3.73E-11	0.824786015
7339	Unknown	548332	1.62E-06	0.66409626
7340	Unknown	550113	2.45E-11	0.831881779
7341	LOC552889	552889	0.00172686	-0.479099239
7342	LOC553103	553103	1.34E-08	0.743854006
7343	LOC554174	554174	1.89E-09	0.770608755
7344	CLLU1	574028	8.33E-07	0.676531286
7345	Unknown	574037	0.002574036	-0.463894798
7346	Unknown	574407	4.27E-10	0.790228243
7347	LOC595101	595101	1.50E-10	0.804490873
7348	LOC606495	606495	0.001783052	0.47786648
7349	LOC613126	613126	0.002724295	0.461598515
7350	CTXN3	613212	1.57E-07	0.705720018
7351	FDPSL2A	619190	2.19E-05	0.608785272
7352	LOC619207	619207	3.66E-07	0.691329346
7353	C8orf68	619343	7.75E-11	0.813288085
7354	C8orf75	619351	9.88E-10	0.779277554
7355	Unknown	619426	6.13E-08	0.720672504
7356	C8orf66	619427	0.000856584	0.50458027
7357	LOC641298	641298	0.000313191	0.537635431
7358	LOC641365	641365	1.68E-05	0.614903926
7359	ACOT6	641372	0.00725627	0.420056791
7360	SNHG6	641638	0.000611383	-0.516187545
7361	ECSM2	641700	0.009347581	0.408019021
7362	LOC642313	642313	1.38E-09	0.774811323
7363	hCG_1795283	642345	1.25E-09	0.776158805
7364	LOC642361	642361	0.009133799	-0.409120049
7365	C10orf109	642394	4.10E-08	0.726979986
7366	Unknown	642533	2.95E-10	0.795138227
7367	LOC642597	642597	3.85E-05	0.59503715
7368	FLJ37786	642691	0.008979121	0.409957411
7369	FLJ32756	642757	0.000478547	0.52422069
7370	LOC642776	642776	0.00011733	0.566011903
7371	LOC642864	642864	0.009294216	0.408288382
7372	LQK1	642946	8.20E-12	0.85751233
7373	LOC642980	642980	8.09E-06	0.631372259
7374	LOC643072	643072	0.007607571	-0.417823454

7375	LOC643085	643085	2.85E-11	0.828652485
7376	FLJ40292	643210	6.52E-09	0.75390174
7377	MGC157906	643236	3.42E-08	0.729646183
7378	FLJ44054	643365	0.00028756	0.540210095
7379	C14orf176	643382	3.26E-10	0.793903148
7380	FLJ40606	643549	3.68E-07	0.691250179
7381	LOC643650	643650	4.84E-06	0.642248018
7382	hCG_2019139	643659	0.000436514	0.52717716
7383	LOC643677	643677	5.31E-07	0.684745682
7384	LOC643714	643714	2.26E-07	0.699550321
7385	LOC643836	643836	0.001989053	-0.473740649
7386	CBLN3	643866	1.02E-05	0.62621255
7387	LOC643923	643923	4.55E-09	0.758913938
7388	LOC643988	643988	0.007810679	0.416624238
7389	MGC87895	644068	0.000802282	-0.50687855
7390	LOC644090	644090	3.92E-06	0.646644262
7391	LOC644096	644096	0.007675404	-0.417406673
7392	LOC644135	644135	0.000191859	0.552190279
7393	hCG_1776018	644139	2.26E-10	0.798794771
7394	WIPF3	644150	5.88E-11	0.817689011
7395	LOC644620	644620	9.41E-07	0.674285439
7396	LOC644714	644714	1.55E-08	0.741699357
7397	FLJ38596	644809	1.14E-05	0.623802321
7398	LOC644852	644852	0.005198458	0.434814528
7399	FLJ30064	644975	2.79E-05	0.602882844
7400	C1orf200	644997	1.35E-08	0.743723272
7401	LOC645212	645212	0.003583285	-0.450543761
7402	LOC645355	645355	2.23E-06	0.657914841
7403	LOC645591	645591	3.95E-07	0.689952177
7404	LOC645676	645676	1.55E-05	0.616876253
7405	LOC645687	645687	9.65E-05	0.571386445
7406	PABPC1L2B	645974	0.000248898	0.54455167
7407	LOC645984	645984	5.71E-05	0.585129571
7408	CBY3	646019	2.51E-11	0.83133225
7409	Unknown	646023	8.68E-05	0.57422998
7410	LOC646168	646168	3.86E-06	0.646930011
7411	LOC646241	646241	2.09E-08	0.737309956
7412	Unknown	646329	4.49E-09	0.759133131
7413	Unknown	646383	0.001423616	0.486299701
7414	LOC646482	646482	5.19E-06	0.640809004
7415	LOC646484	646484	2.77E-10	0.795928919
7416	LOC646588	646588	6.23E-10	0.785361977
7417	C3orf65	646600	7.05E-05	0.579651988
7418	LOC646626	646626	6.57E-11	0.815431839
7419	LOC646701	646701	6.11E-10	0.785601751

7420	LOC646778	646778	1.38E-08	0.743449401
7421	Unknown	646888	9.52E-09	0.748600547
7422	LOC647107	647107	1.01E-05	0.62653441
7423	FLJ36848	647115	1.47E-09	0.773939584
7424	LOC647309	647309	1.11E-07	0.711359515
7425	Unknown	647310	3.09E-05	0.600452841
7426	LOC647323	647323	3.38E-10	0.793444408
7427	Unknown	647476	4.32E-07	0.688333939
7428	Unknown	647946	0.001205613	-0.492378976
7429	LOC647979	647979	0.000195899	-0.551561387
7430	FLJ35776	649446	0.002507987	0.464917841
7431	PP8961	650662	5.56E-07	0.683931994
7432	Unknown	653110	5.71E-05	0.585111561
7433	ZBTB8	653121	0.002578619	-0.463818429
7434	Unknown	653390	1.13E-06	0.670918098
7435	PGM5P1	653394	0.009887456	0.405347812
7436	GPR89A	653519	0.001388903	-0.487255974
7437	PHLDB3	653583	0.009417965	-0.407637722
7438	FAM128A	653784	0.000610034	-0.516262891
7439	LOC653808	653808	3.14E-09	0.763891813
7440	LRTM2	654429	0.002110342	0.471537303
7441	SCARNA2	677766	2.95E-09	0.764773182
7442	SCARNA15	677778	0.006846769	-0.422725203
7443	SNORA28	677811	3.85E-10	0.791639158
7444	Unknown	692205	2.66E-11	0.829947773
7445	CXorf31	724087	0.00036806	0.532504921
7446	RNF208	727800	0.000340664	0.5350186
7447	LOC727818	727818	4.16E-07	0.689016193
7448	LOC727930	727930	0.000624605	0.515435837
7449	LOC728073	728073	0.007628226	0.417697619
7450	LOC728095	728095	0.009815317	0.405711388
7451	Unknown	728175	1.13E-11	0.851198941
7452	LOC728190	728190	6.77E-07	0.680328377
7453	LOC728192	728192	2.91E-07	0.695255899
7454	Unknown	728224	7.36E-11	0.81392733
7455	FLJ33544	728283	9.91E-08	0.713160649
7456	LOC728353	728353	1.61E-05	0.615893944
7457	hCG_2045828	728434	3.96E-09	0.760838961
7458	hCG_2020170	728437	0.005076426	0.435827206
7459	Unknown	728475	7.29E-05	0.578781846
7460	LOC728485	728485	2.26E-11	0.833166776
7461	LOC728498	728498	0.001546962	-0.483211342
7462	LOC728543	728543	4.61E-05	0.590545803
7463	CDC2L2	728642	0.001674556	-0.48024367
7464	RP11-345P4.4	728661	1.12E-05	-0.624232039

7465	LOC728730	728730	0.004076333	-0.445123109
7466	Unknown	728789	4.54E-09	0.758962365
7467	LOC728805	728805	4.26E-06	0.644865288
7468	LOC728855	728855	0.000219864	-0.548222523
7469	LOC728868	728868	5.70E-07	0.683504308
7470	Unknown	728927	3.42E-08	0.729666126
7471	LOC729059	729059	6.98E-05	0.579918639
7472	LOC729082	729082	0.001069704	-0.496588446
7473	LOC729121	729121	8.57E-05	0.574572263
7474	LOC729173	729173	0.000110183	0.567804016
7475	LOC729178	729178	0.003083326	0.456748525
7476	hCG_2044152	729224	5.40E-07	0.684411262
7477	FLJ78302	729230	2.65E-05	0.60416246
7478	LOC729291	729291	3.66E-05	0.596291781
7479	Unknown	729296	4.21E-10	0.790503687
7480	C6orf35	729515	1.73E-08	0.740147222
7481	DIRC3	729582	0.000447348	0.526356875
7482	Unknown	729614	5.72E-05	-0.585049801
7483	LOC729680	729680	0.000113897	0.566841328
7484	LOC729830	729830	4.27E-05	0.592474467
7485	LOC729839	729839	5.34E-11	0.819241642
7486	hCG_1994895	729866	5.14E-06	0.641003318
7487	LOC729870	729870	0.006616082	-0.424276227
7488	FLJ30403	729975	2.45E-05	0.606063744
7489	LOC730091	730091	4.10E-09	0.760364485
7490	LOC730101	730101	0.000218869	0.548352767
7491	LOC730184	730184	1.35E-11	0.842342955
7492	LOC730441	730441	3.40E-10	0.793336383
7493	Unknown	730755	0.002028185	0.4730194
7494	LOC731157	731157	5.27E-05	0.58720018
7495	Unknown	731223	0.000354826	0.533697632
7496	Unknown	731424	9.42E-11	0.810555218
7497	LOC731656	731656	4.45E-09	0.759280035
7498	Unknown	731779	3.97E-08	0.727411037
7499	LOC732096	732096	6.00E-05	0.583789269
7500	Unknown	732253	6.37E-09	0.754232617
7501	LOC751071	751071	0.000523755	-0.521259483
7502	PRCD	768206	2.64E-10	0.796584796
7503	FAM18A	780776	3.75E-07	0.690917021
7504	LOC790955	790955	0.00024674	-0.544835117
7505	Unknown	791114	8.00E-07	0.677280929
7506	Unknown	791115	2.94E-11	0.827976299
7507	LOC100049716	100049716	0.008347797	-0.41346732
7508	Unknown	100093630	0.004330259	-0.442574341
7509	LOC100093698	100093698	9.30E-05	0.572358021

7510	Unknown	100101266	1.05E-09	0.778523347
7511	Unknown	100101467	9.28E-09	0.748952041
7512	Unknown	100101938	2.95E-06	0.652392382
7513	LOC100126784	100126784	2.66E-05	0.604078588
7514	Unknown	100127888	0.000868853	0.504076197
7515	Unknown	100127940	0.000103566	0.569515265
7516	Unknown	100127972	8.35E-08	0.715851688
7517	Unknown	100127980	1.76E-09	0.771527759
7518	Unknown	100128003	1.33E-07	0.708401166
7519	Unknown	100128025	9.33E-10	0.780195141
7520	Unknown	100128071	5.34E-07	0.684634237
7521	Unknown	100128081	4.81E-05	0.589437247
7522	Unknown	100128098	0.000633865	0.514899455
7523	Unknown	100128126	3.34E-11	0.826228499
7524	Unknown	100128175	5.94E-08	0.72120058
7525	Unknown	100128198	6.27E-07	0.681749172
7526	Unknown	100128239	9.66E-06	0.627414058
7527	Unknown	100128252	0.007000776	-0.421701841
7528	Unknown	100128262	1.80E-07	0.703419255
7529	Unknown	100128281	0.000349102	0.53421904
7530	Unknown	100128292	5.77E-10	0.786326503
7531	Unknown	100128298	1.48E-05	0.617890428
7532	Unknown	100128343	0.008055489	-0.415188385
7533	Unknown	100128496	6.91E-07	0.679950298
7534	Unknown	100128554	6.31E-08	0.720205717
7535	Unknown	100128594	0.006293736	0.426446236
7536	Unknown	100128640	6.19E-11	0.8164623
7537	Unknown	100128731	0.000939426	-0.501233882
7538	Unknown	100128737	2.63E-09	0.766169604
7539	Unknown	100128751	0.009659056	0.406482647
7540	Unknown	100128782	0.005792806	0.430093144
7541	Unknown	100128822	0.005198458	-0.434818047
7542	Unknown	100128840	4.05E-05	0.593805163
7543	Unknown	100128946	0.004253631	0.443354076
7544	Unknown	100128988	6.20E-08	0.720492932
7545	Unknown	100128993	2.85E-06	0.653106481
7546	Unknown	100129058	3.52E-10	0.792889244
7547	Unknown	100129098	0.00718422	0.420558005
7548	Unknown	100129103	2.15E-09	0.768833324
7549	Unknown	100129129	9.82E-09	0.748178307
7550	Unknown	100129175	9.69E-10	0.779613267
7551	Unknown	100129196	7.61E-08	0.717292299
7552	Unknown	100129198	1.67E-06	0.663506298
7553	Unknown	100129239	1.33E-07	0.708381718
7554	Unknown	100129282	0.008685603	0.411529743

7555	Unknown	100129380	0.007471106	0.418689972
7556	Unknown	100129427	0.000265816	0.542551238
7557	Unknown	100129449	1.56E-08	0.741594174
7558	Unknown	100129461	3.17E-08	0.730872025
7559	Unknown	100129503	0.002815352	0.460289339
7560	Unknown	100129597	5.12E-10	0.787896205
7561	Unknown	100129603	3.10E-06	0.651335478
7562	Unknown	100129617	0.002945829	0.458512229
7563	Unknown	100129662	6.02E-11	0.81715151
7564	Unknown	100129827	2.40E-09	0.767475797
7565	Unknown	100129845	2.31E-09	0.767941074
7566	Unknown	100129852	0.002247354	-0.46913221
7567	Unknown	100129858	3.23E-07	0.693375811
7568	Unknown	100129884	2.38E-10	0.797943484
7569	Unknown	100129890	1.11E-06	0.671200485
7570	Unknown	100130078	0.00095186	0.500763243
7571	Unknown	100130097	5.29E-06	0.640417684
7572	Unknown	100130155	1.54E-09	0.773433441
7573	Unknown	100130175	6.35E-08	0.72010165
7574	Unknown	100130219	1.42E-08	0.743000289
7575	Unknown	100130249	2.92E-05	0.601806884
7576	Unknown	100130264	9.53E-06	0.627725775
7577	Unknown	100130278	8.15E-05	0.575886167
7578	Unknown	100130288	0.004424596	-0.441644005
7579	Unknown	100130322	3.84E-05	0.595107955
7580	Unknown	100130331	2.13E-09	0.769003249
7581	Unknown	100130353	0.004253144	0.443368797
7582	Unknown	100130357	2.60E-08	0.733921109
7583	Unknown	100130360	6.15E-06	0.637237242
7584	Unknown	100130417	0.003766817	0.448472861
7585	Unknown	100130418	2.31E-11	0.83249375
7586	Unknown	100130428	4.98E-05	0.588593762
7587	Unknown	100130456	7.06E-11	0.814490035
7588	Unknown	100130458	3.14E-06	0.65109045
7589	Unknown	100130502	1.06E-08	0.74718061
7590	Unknown	100130522	1.29E-08	0.74438235
7591	Unknown	100130548	0.002230852	0.469442577
7592	Unknown	100130609	0.001045713	0.497414443
7593	Unknown	100130613	1.42E-07	0.70741038
7594	Unknown	100130700	2.62E-07	0.697019028
7595	Unknown	100130705	3.89E-07	0.690278736
7596	Unknown	100130815	7.39E-07	0.678707908
7597	Unknown	100130837	0.006471896	-0.425264314
7598	Unknown	100130856	7.97E-05	0.57651016
7599	Unknown	100130938	0.008443816	0.412874875

7600	Unknown	100130958	1.64E-09	0.772558505
7601	Unknown	100130967	2.95E-08	0.731964876
7602	Unknown	100130992	1.90E-06	0.660988153
7603	Unknown	100130998	3.44E-06	0.649244808
7604	Unknown	100131015	1.96E-07	0.701987888
7605	Unknown	100131053	0.00777169	0.416849912
7606	Unknown	100131077	7.69E-11	0.813387559
7607	Unknown	100131112	1.35E-11	0.842527724
7608	Unknown	100131117	2.13E-08	0.736994696
7609	Unknown	100131180	7.22E-05	0.579050284
7610	Unknown	100131187	8.38E-05	-0.575165255
7611	Unknown	100131213	6.65E-10	0.784573312
7612	Unknown	100131227	8.57E-05	0.574587261
7613	Unknown	100131275	2.23E-11	0.833756196
7614	Unknown	100131283	7.98E-05	-0.576465023
7615	Unknown	100131298	4.17E-11	0.822523574
7616	Unknown	100131303	0.000692835	0.511890921
7617	Unknown	100131316	0.003298943	0.453938548
7618	Unknown	100131354	4.02E-11	0.823260466
7619	Unknown	100131366	0.001253308	0.49097833
7620	Unknown	100131508	0.005227184	0.434560749
7621	Unknown	100131510	0.001116324	0.495106551
7622	Unknown	100131561	0.000855905	0.504618603
7623	Unknown	100131581	4.89E-09	0.757860278
7624	Unknown	100131642	1.12E-05	0.62421874
7625	Unknown	100131655	2.87E-11	0.828447183
7626	Unknown	100131733	3.83E-07	0.690520895
7627	Unknown	100131755	0.000448765	0.526262776
7628	Unknown	100131766	5.36E-10	0.787345964
7629	Unknown	100131781	3.80E-06	0.647284687
7630	Unknown	100131801	0.003492906	-0.45161147
7631	Unknown	100131816	5.71E-09	0.755745841
7632	Unknown	100131825	0.005323946	0.433752227
7633	Unknown	100131864	1.08E-05	0.62497667
7634	Unknown	100131998	3.21E-10	0.794084072
7635	Unknown	100132005	1.70E-10	0.802528341
7636	Unknown	100132025	0.001973297	0.474044977
7637	Unknown	100132051	0.00955307	0.407001418
7638	Unknown	100132080	0.005187165	0.434917614
7639	Unknown	100132116	5.91E-08	0.721324116
7640	Unknown	100132178	7.31E-05	0.578696494
7641	Unknown	100132234	0.000353625	0.533808183
7642	Unknown	100132285	3.40E-10	0.793354502
7643	Unknown	100132354	3.80E-05	0.595366313
7644	Unknown	100132406	0.000624697	-0.515425715

7645	Unknown	100132460	5.05E-10	0.788078318
7646	Unknown	100132476	0.001244846	0.491233882
7647	Unknown	100132661	5.78E-07	0.683219583
7648	Unknown	100132686	2.48E-11	0.831556737
7649	Unknown	100132735	0.003822988	0.44783155
7650	Unknown	100132911	1.32E-09	0.775451172
7651	Unknown	100133039	0.004226156	-0.443650353
7652	Unknown	100133089	6.79E-07	0.68027413
7653	Unknown	100133130	0.008239719	0.414086811
7654	Unknown	100133131	2.48E-05	0.605790251
7655	Unknown	100133142	4.89E-08	0.724282911
7656	Unknown	100133461	1.46E-05	0.618146931
7657	Unknown	100133660	4.55E-05	0.590884004
7658	Unknown	100133790	1.55E-10	0.804105351
7659	Unknown	100133985	4.02E-11	0.823233495
7660	Unknown	100134259	1.30E-11	0.846738494
7661	Unknown	100134368	1.51E-07	0.706282931
7662	Unknown	100134713	0.000549478	0.519726572
7663	Unknown	100144602	0.000418883	0.528453782
7664	Unknown	100144604	0.002117692	0.471400693
7665	Unknown	100144748	2.77E-05	0.60306332
7666	Unknown	100169989	6.34E-05	0.582390617
7667	Unknown	100170229	8.74E-07	0.675629658
7668	Unknown	100170841	2.53E-05	0.605290898
7669	Unknown	100188953	0.000103002	0.569673423
7670	Unknown	100192378	5.89E-10	0.786065337
7671	Unknown	100192379	0.000915985	0.502195268
7672	Unknown	100192386	0.000442766	0.526691611
7673	Unknown	100240726	1.15E-11	0.849720757
7674	Unknown	100240728	0.004710959	0.439029151
7675	Unknown	100240734	0.001453372	0.485579695
7676	Unknown	100268168	2.38E-05	0.606721593
7677	Unknown	100272147	0.002688886	-0.462118711
7678	Unknown	100272228	4.93E-09	0.757736757
7679	Unknown	100286895	1.12E-10	0.808202628
7680	Unknown	100286921	2.94E-11	0.827988873
7681	Unknown	100286987	1.47E-07	0.706761811
7682	Unknown	100287027	0.009148342	0.409046501
7683	Unknown	100287037	0.001334571	0.488760167
7684	Unknown	100287076	0.001042073	0.497540014
7685	Unknown	100287105	6.05E-07	0.682397473
7686	Unknown	100287227	5.43E-10	0.787180395
7687	Unknown	100287290	1.12E-08	0.746282443
7688	Unknown	100287331	0.000650064	0.514081699
7689	Unknown	100287372	1.49E-07	0.706607316

7690	Unknown	100287426	5.60E-08	0.722132974
7691	Unknown	100287432	0.000291038	0.539827854
7692	Unknown	100287525	5.57E-11	0.818394998
7693	Unknown	100287547	1.04E-06	0.672439942
7694	Unknown	100287556	7.39E-09	0.752196622
7695	Unknown	100287558	5.26E-11	0.819618455
7696	Unknown	100287572	0.004759631	-0.438600852
7697	Unknown	100287584	3.21E-08	0.730670422
7698	Unknown	100287598	9.42E-06	0.627995513
7699	Unknown	100287621	0.003161257	0.455686635
7700	Unknown	100287627	0.005783272	0.430189065
7701	Unknown	100287704	0.000616747	0.51589488
7702	Unknown	100287813	1.64E-06	0.663859063
7703	Unknown	100287877	0.000114698	0.566644354
7704	Unknown	100287896	0.005868932	-0.429482164
7705	Unknown	100287927	4.13E-07	0.68914409
7706	Unknown	100287932	0.000458697	-0.525586078
7707	Unknown	100287951	0.002425943	0.466280232
7708	Unknown	100288092	1.59E-09	0.772926407
7709	Unknown	100288099	2.98E-06	0.652119034
7710	Unknown	100288123	0.000359079	-0.533324555
7711	Unknown	100288181	0.008186987	0.414369353
7712	Unknown	100288194	3.06E-11	0.827228803
7713	Unknown	100288224	3.16E-05	0.59988169
7714	Unknown	100288282	6.12E-11	0.816783343
7715	Unknown	100288490	3.97E-11	0.82351132
7716	Unknown	100288525	0.000659574	0.513532051
7717	Unknown	100288594	3.47E-05	0.597627135
7718	Unknown	100288673	0.003383141	0.452888679
7719	Unknown	100288701	1.34E-09	0.775221675
7720	Unknown	100288721	1.42E-07	0.707378759
7721	Unknown	100288730	0.007454102	-0.418793618
7722	Unknown	100288745	0.000998565	0.499103545
7723	Unknown	100288797	0.000478222	-0.524257697
7724	Unknown	100288805	0.006340658	0.42611847
7725	Unknown	100288810	4.80E-06	0.642402575
7726	Unknown	100288893	0.008239719	0.414089698
7727	Unknown	100288911	1.80E-06	0.662010302
7728	Unknown	100288954	3.81E-06	0.647201561
7729	Unknown	100289071	0.000125177	0.564183128
7730	Unknown	100289084	0.004577973	-0.440226265
7731	Unknown	100289086	0.001504697	0.484246487
7732	Unknown	100289098	4.19E-10	0.790602541
7733	Unknown	100289210	2.05E-10	0.800058889
7734	Unknown	100289251	1.70E-10	0.802631862

7735	Unknown	100289336	1.16E-05	0.623407946
7736	Unknown	100289361	0.008007397	-0.415463586
7737	Unknown	100289366	9.36E-09	0.748830365
7738	Unknown	100289373	3.61E-08	0.728861216
7739	Unknown	100289436	5.82E-08	0.72156681
7740	Unknown	100289465	0.001263402	0.490706198
7741	Unknown	100289508	0.003217301	0.454964222
7742	Unknown	100289509	3.22E-05	0.599451159
7743	Unknown	100289632	0.000416501	0.528640827
7744	Unknown	100289635	0.006569806	-0.424579567
7745	Unknown	100290278	8.07E-11	0.812713683
7746	Unknown	100292863	8.22E-07	0.676789066
7747	Unknown	100292909	4.77E-11	0.821085246
7748	Unknown	100293553	2.00E-06	0.660093668
7749	Unknown	100293559	2.61E-11	0.830385777
7750	Unknown	100294720	0.000122077	0.564878731
7751	Unknown	100302522	0.001843503	0.47659848
7752	Unknown	100302691	0.003247151	0.454610907
7753	Unknown	100302737	2.28E-05	0.607802477
7754	Unknown	100309464	4.25E-10	0.79032045
7755	Unknown	100316868	7.80E-09	0.751391023
7756	Unknown	100379661	0.000542327	0.520146493
7757	Unknown	100422737	0.000118917	0.56563129
7758	Unknown	100422781	0.00016056	0.557250201
7759	Unknown	100498859	0.004298526	0.44288665
7760	Unknown	100499177	0.003510985	-0.451403766
7761	Unknown	100499193	4.62E-05	0.590413475
7762	Unknown	100499194	9.36E-11	0.810699508
7763	Unknown	100499441	0.000280719	0.540937435
7764	Unknown	100499489	1.62E-08	0.741118048
7765	Unknown	100505478	8.01E-11	0.812870643
7766	Unknown	100505481	0.001588592	0.482241645
7767	Unknown	100505490	1.20E-11	0.848626377
7768	Unknown	100505498	9.40E-10	0.780066214
7769	Unknown	100505515	8.38E-09	0.750374521
7770	Unknown	100505522	0.005633326	-0.431336594
7771	Unknown	100505593	0.003502483	-0.451504824
7772	Unknown	100505598	2.51E-07	0.697796551
7773	Unknown	100505600	3.23E-07	0.693393466
7774	Unknown	100505601	0.00743293	-0.418913933
7775	Unknown	100505609	0.00049996	0.522793033
7776	Unknown	100505649	0.00095385	0.500683125
7777	Unknown	100505697	0.000379783	0.531543975
7778	Unknown	100505711	1.35E-07	0.708127316
7779	Unknown	100505712	0.000992659	-0.499317553

7780	Unknown	100505774	0.004175978	0.444152927
7781	Unknown	100505786	4.24E-06	0.644972177
7782	Unknown	100505797	5.15E-09	0.757131845
7783	Unknown	100505805	0.005208815	0.434716814
7784	Unknown	100505808	1.27E-10	0.806549999
7785	Unknown	100505820	1.70E-10	0.802629319
7786	Unknown	100505833	0.000618268	0.515770869
7787	Unknown	100505842	2.12E-08	0.737124116
7788	Unknown	100505852	0.000669466	0.513035422
7789	Unknown	100505862	9.65E-11	0.810226386
7790	Unknown	100505875	6.57E-05	0.581471872
7791	Unknown	100505881	9.56E-05	0.571618902
7792	Unknown	100505885	2.94E-06	0.652464355
7793	Unknown	100505903	0.001414959	0.486514323
7794	Unknown	100505928	1.24E-05	0.621881648
7795	Unknown	100505935	1.38E-10	0.805552722
7796	Unknown	100505956	0.001734576	0.478927877
7797	Unknown	100505960	0.0076713	-0.417442534
7798	Unknown	100505967	3.70E-09	0.761783854
7799	Unknown	100505986	3.41E-05	0.598107926
7800	Unknown	100506012	1.49E-11	0.84033382
7801	Unknown	100506014	6.60E-05	0.581322157
7802	Unknown	100506030	2.89E-07	0.695357989
7803	Unknown	100506037	4.50E-06	0.643740181
7804	Unknown	100506047	4.22E-08	0.726555357
7805	Unknown	100506051	1.66E-06	0.663648808
7806	Unknown	100506082	0.002251868	0.469054607
7807	Unknown	100506088	9.54E-07	0.674012413
7808	Unknown	100506100	0.00064862	0.514155428
7809	Unknown	100506114	0.00075484	0.509002314
7810	Unknown	100506125	1.28E-05	0.621218785
7811	Unknown	100506172	1.90E-06	0.660987839
7812	Unknown	100506216	9.21E-08	0.714260847
7813	Unknown	100506234	0.000148081	0.559553638
7814	Unknown	100506237	0.000157089	0.557901871
7815	Unknown	100506238	4.91E-08	0.724198357
7816	Unknown	100506245	5.64E-05	0.585447993
7817	Unknown	100506259	9.55E-05	0.571662425
7818	Unknown	100506266	0.000381694	-0.531394276
7819	Unknown	100506267	0.00046186	0.525382143
7820	Unknown	100506288	2.32E-10	0.79829831
7821	Unknown	100506348	1.83E-05	0.613027446
7822	Unknown	100506354	4.49E-07	0.687627513
7823	Unknown	100506379	1.68E-05	0.615014351
7824	Unknown	100506380	3.34E-08	0.730009765

7825	Unknown	100506418	0.008944518	-0.410151576
7826	Unknown	100506430	0.001030358	0.497980836
7827	Unknown	100506436	6.68E-09	0.753578212
7828	Unknown	100506457	3.23E-09	0.763538506
7829	Unknown	100506470	0.00014773	0.559632783
7830	Unknown	100506480	0.007141037	0.420826163
7831	Unknown	100506521	0.001711688	0.479463397
7832	Unknown	100506530	1.06E-06	0.672024995
7833	Unknown	100506555	0.007884411	0.416186066
7834	Unknown	100506558	0.000772722	0.508155855
7835	Unknown	100506652	0.007431381	0.418927161
7836	Unknown	100506676	4.64E-08	0.725099333
7837	Unknown	100506677	7.40E-08	0.717730139
7838	Unknown	100506696	0.000520679	0.521479739
7839	Unknown	100506714	4.60E-10	0.789228137
7840	Unknown	100506727	0.001347258	-0.488418957
7841	Unknown	100506733	0.004681252	0.439321289
7842	Unknown	100506757	0.003887339	0.447106289
7843	Unknown	100506762	8.89E-11	0.811403414
7844	Unknown	100506767	0.000232011	0.546640953
7845	Unknown	100506797	2.68E-08	0.733434211
7846	Unknown	100506816	6.77E-05	0.580683025
7847	Unknown	100506834	1.99E-10	0.800525743
7848	Unknown	100506843	0.000528395	0.520974029
7849	Unknown	100506851	1.95E-07	0.702067261
7850	Unknown	100506858	5.03E-11	0.82036628
7851	Unknown	100506860	3.75E-09	0.761597035
7852	Unknown	100506866	2.29E-05	0.607684694
7853	Unknown	100506880	3.05E-05	0.600778796
7854	Unknown	100506907	8.44E-07	0.676268231
7855	Unknown	100506958	0.001188925	0.49288343
7856	Unknown	100506992	0.000965391	0.500285792
7857	Unknown	100506995	1.11E-09	0.777793933
7858	Unknown	100506997	2.41E-10	0.797749448
7859	Unknown	100507009	4.80E-07	0.686463082
7860	Unknown	100507028	6.57E-05	0.581447565
7861	Unknown	100507049	0.003202436	0.455152298
7862	Unknown	100507054	0.005490423	0.432407142
7863	Unknown	100507063	3.36E-08	0.72994048
7864	Unknown	100507077	7.91E-09	0.751174427
7865	Unknown	100507098	0.000376703	0.531791324
7866	Unknown	100507109	4.82E-06	0.642342498
7867	Unknown	100507123	2.90E-11	0.828186109
7868	Unknown	100507156	6.19E-11	0.816486709
7869	Unknown	100507164	0.000100825	0.570229432

7870	Unknown	100507177	3.20E-06	0.650678424
7871	Unknown	100507193	3.83E-05	0.595196335
7872	Unknown	100507194	1.73E-10	0.802329485
7873	Unknown	100507215	3.89E-05	0.594779744
7874	Unknown	100507224	0.00028189	0.540795961
7875	Unknown	100507226	1.56E-08	0.741577282
7876	Unknown	100507250	0.004417445	-0.441723542
7877	Unknown	100507291	8.86E-08	0.714901029
7878	Unknown	100507303	0.002108035	-0.471586763
7879	Unknown	100507307	8.01E-11	0.812839245
7880	Unknown	100507362	3.63E-08	0.72878658
7881	Unknown	100507390	2.77E-11	0.829358262
7882	Unknown	100507408	9.77E-10	0.779499179
7883	Unknown	100507424	0.005251664	-0.434362127
7884	Unknown	100507443	3.80E-05	0.595410144
7885	Unknown	100507454	0.002204878	0.469869333
7886	Unknown	100507461	3.11E-05	0.600263668
7887	Unknown	100507466	0.003539423	0.451056555
7888	Unknown	100507492	5.93E-08	0.721258152
7889	Unknown	100507525	2.74E-07	0.696219178
7890	Unknown	100507531	2.56E-09	0.766523941
7891	Unknown	100507540	0.000596761	0.517001076
7892	Unknown	100507560	0.000494146	0.523170144
7893	Unknown	100507572	2.04E-05	0.610448748
7894	Unknown	100507596	2.27E-06	0.657569445
7895	Unknown	100507620	0.001523368	0.483804833
7896	Unknown	100507625	3.55E-08	0.729122375
7897	Unknown	100507652	0.002989023	0.457936778
7898	Unknown	100507675	3.74E-11	0.824641267
7899	Unknown	100507741	1.66E-10	0.802989496
7900	Unknown	100507745	2.66E-09	0.766038705
7901	Unknown	100507904	1.60E-07	0.705416117
7902	Unknown	100507928	4.68E-09	0.75848583
7903	Unknown	100508095	2.09E-08	0.73736027
7904	Unknown	100508124	8.29E-05	0.575431145
7905	Unknown	100508176	0.0055769	-0.431768871
7906	Unknown	100508307	4.08E-07	0.689334665
7907	Unknown	100508341	5.31E-05	0.586971652
7908	Unknown	100508368	1.18E-06	0.670020268
7909	Unknown	100508428	0.004807943	0.438169368
7910	Unknown	100508528	0.001924199	0.47497827
7911	Unknown	100508567	2.67E-07	0.696693385
7912	Unknown	100508624	8.41E-09	0.75030075
7913	Unknown	100508657	3.08E-09	0.764201221
7914	Unknown	100508709	0.00026456	0.542707351

7915	Unknown	100508787	2.12E-08	0.737082154
7916	Unknown	100508889	4.86E-05	0.589153468
7917	Unknown	100508951	0.00135838	0.488093875
7918	Unknown	100508968	8.82E-06	0.629458716
7919	Unknown	100509020	0.001881354	-0.475805661
7920	Unknown	100509030	5.20E-11	0.81985408
7921	Unknown	100509093	5.20E-08	0.723311046
7922	Unknown	100509139	4.08E-06	0.645781699
7923	Unknown	100509212	2.08E-06	0.659276698
7924	Unknown	100509388	2.58E-06	0.655037626
7925	Unknown	100509550	1.18E-07	0.710311815
7926	Unknown	100509621	0.006596915	0.424396982
7927	Unknown	100509639	2.61E-11	0.830560027
7928	Unknown	100509662	3.35E-07	0.692781307
7929	Unknown	100509761	0.000770368	0.508278802
7930	Unknown	100509762	0.000904266	0.502695685
7931	Unknown	100509893	3.51E-06	0.648835364
7932	Unknown	100510080	1.26E-09	0.775976797
7933	Unknown	100510453	0.001635941	0.481158177

Gene IDs did not match any gene symbols were defined as "Unknown".
